# Genetically-encoded discovery of proteolytically stable bicyclic inhibitors for morphogen NODAL†

**DOI:** 10.1039/d1sc01916c

**Published:** 2021-06-17

**Authors:** Jeffrey Y.-K. Wong, Raja Mukherjee, Jiayuan Miao, Olena Bilyk, Vivian Triana, Mark Miskolzie, Antoine Henninot, John J. Dwyer, Serhii Kharchenko, Anna Iampolska, Dmitriy M. Volochnyuk, Yu-Shan Lin, Lynne-Marie Postovit, Ratmir Derda

**Affiliations:** Department of Chemistry, University of Alberta Edmonton AB T6G 2G2 Canada ratmir@ualberta.ca; Department of Chemistry, Tufts University Medford MA 02155 USA; Department of Experimental Oncology, University of Alberta Edmonton AB T6G 2G2 Canada; Ferring Research Institute San Diego California 92121 USA; Enamine Ltd. Chervonotkatska Street 78 Kyiv 02094 Ukraine

## Abstract

In this manuscript, we developed a two-fold symmetric linchpin (**TSL**) that converts readily available phage-displayed peptides libraries made of 20 common amino acids to genetically-encoded libraries of bicyclic peptides displayed on phage. **TSL** combines an aldehyde-reactive group and two thiol-reactive groups; it bridges two side chains of cysteine [C] with an N-terminal aldehyde group derived from the N-terminal serine [S], yielding a novel bicyclic topology that lacks a free N-terminus. Phage display libraries of SX_1_CX_2_X_3_X_4_X_5_X_6_X_7_C sequences, where X is any amino acid but Cys, were converted to a library of bicyclic **TSL**-[S]X_1_[C]X_2_X_3_X_4_X_5_X_6_X_7_[C] peptides in 45 ± 15% yield. Using this library and protein morphogen NODAL as a target, we discovered bicyclic macrocycles that specifically antagonize NODAL-induced signaling in cancer cells. At a 10 μM concentration, two discovered bicyclic peptides completely suppressed NODAL-induced phosphorylation of SMAD2 in P19 embryonic carcinoma cells. The **TSL**-[S]Y[C]KRAHKN[C] bicycle inhibited NODAL-induced proliferation of NODAL-TYK-nu ovarian carcinoma cells with apparent IC_50_ of 1 μM. The same bicycle at 10 μM concentration did not affect the growth of the control TYK-nu cells. **TSL**-bicycles remained stable over the course of the 72 hour-long assays in a serum-rich cell-culture medium. We further observed general stability in mouse serum and in a mixture of proteases (Pronase™) for 21 diverse bicyclic macrocycles of different ring sizes, amino acid sequences, and cross-linker geometries. **TSL**-constrained peptides to expand the previously reported repertoire of phage-displayed bicyclic architectures formed by cross-linking Cys side chains. We anticipate that it will aid the discovery of proteolytically stable bicyclic inhibitors for a variety of protein targets.

## Abstract 2

A two-fold symmetric linchpin (**TSL**) converts readily available phage-displayed disulfide peptide libraries to proteolytically stable bicyclic peptides. The bicyclic phage library was screened to discover an antagonist of NODAL morphogen.

## Graphical abstract

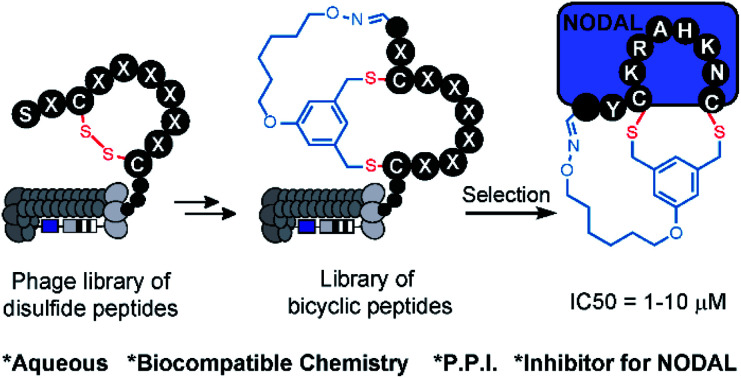

## Introduction

Peptide macrocycles constitute a significant fraction of approved peptide therapeutics: around 30 out of 80 peptide drugs on the global market; macrocyclic topologies are prevalent among 150 peptides in clinical development and in 400–600 peptides undergoing preclinical studies.^1–4^ Macrocyclization of peptides increases binding affinity, improves permeability through the cell membrane, and increases stability towards enzymatic hydrolysis compared to linear peptides.^5–9^ The large surface area of macrocycles has been critical for identifying molecules that bind extended protein surfaces and inhibit protein–protein interactions.^10^ Introduction of a bridgehead into macrocyclic topologies to form so-called bicyclic peptides could further decrease conformational flexibility and increase stability or binding potency.^5,11^ Bioactive bicyclic peptides that have been reported thus far originate from natural products,^12^ computational approaches,^13–15^ cyclization of known bioactive peptides,^16–19^ or screening of combinatorial libraries.^20–22^ To fuel the last method, synthesis on the solid support can yield libraries of 10^2^–10^5^ diversity,^20–22^ whereas late-stage chemical diversification of biosynthesized peptides displayed on mRNA^11,23–25^ or phage^26,27^ can give rise to bicyclic libraries with 10^9^–10^12^ diversity. DNA-encoded libraries (DELs) have been used extensively to synthesize mono-cyclic libraries of 10^4^–10^8^ members^28–31^ and recently 10^12^ members;^32^ although there are no published examples of bicyclic DELs, the late-stage chemical diversification used in phage and mRNA display can be applied to DELs to generate such libraries.^33^ Development of new approaches for late-stage chemical diversification of encoded libraries^34–36^ make it possible to screen and discover new macrocyclic and bicyclic topologies with value-added properties.

**Fig. 1:**
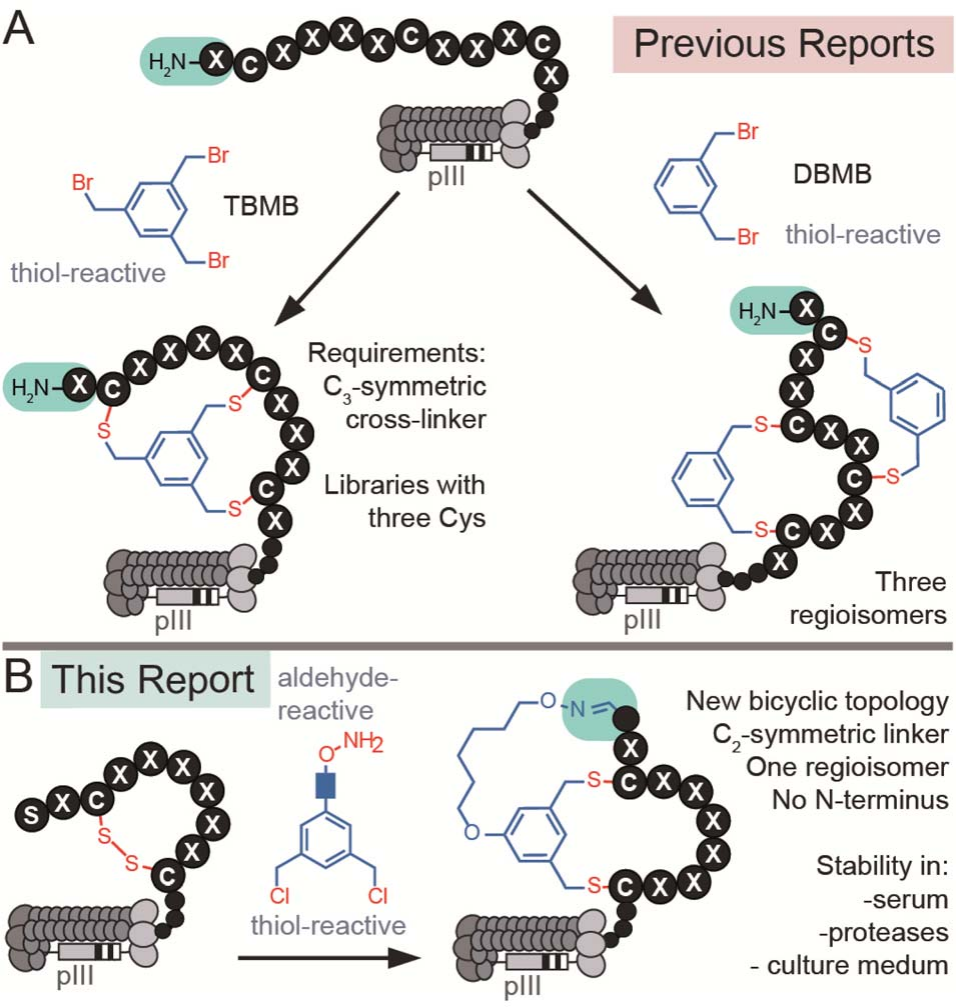
(A) Previous reports of synthesis of bicyclic phage-displayed peptide libraries. (B) Synthesis of bicyclic phage-displayed peptide libraries described in this report.

There are currently two strategies for synthesizing chemically-modified phage-displayed bicyclic libraries. Both employ crosslinking of Cys side chains with electrophiles (Fig. 1A). The first approach pioneered by Winter and Heinis cross-links three Cys residues with a *C*_3_-symmetric electrophile to yield bicycles displayed on phage (Fig. 1A).^26^ This approach was developed extensively by Heinis group^37,38^ and researchers at Bicyclic Therapeutics^39,40^ and employed recently by Slavoff and co-workers^41^ and Wales, Balasubramanian and co-workers.^42^ The second approach published recently by Heinis group employs cross-linking of four Cys residues with *C*_2_-symmetric electrophiles to yield a mixture of three regioisomeric bicycles displayed on phage (Fig. 1A).^43,44^ Bicyclic libraries have also been synthesized in mRNA display libraries using the strategy (i),^24^*via* incorporation of two pairs of orthogonal reactive unnatural amino acids (UAAs) into mRNA display libraries,^25^ or *via* a combination of the two approaches.^11,45^ Incorporation of UAAs into phage-displayed peptide libraries is possible,^46,47^ and UAAs have been used to generate phage-displayed macrocyclic libraries.^48,49^ In this manuscript, we sought to devise the modification approach that uses peptide libraries made of 20 natural amino acids: bypassing the complexity of UAA incorporation avoids biases that might result from the incorporation of such UAAs in the phage library.^50^ We combined modifications of N-terminal Ser and Cys-side chains to generate a novel genetically-encoded bicyclic topology (Fig. 1B). Contrast to previous topologies (Fig. 1A), this topology does not display a free N-terminus and unlike strategies that modify four Cys residues,^43,44^ this cyclization strategy yields a single regioisomer (Fig. 1B).

Aldehyde is a versatile bio-orthogonal handle. In proteins, aldehydes can be incorporated by periodate oxidation of N-terminal Ser.^51,52^ This method has been used for PEGylation of clinically relevant growth factors,^53^ for improving the stability of cytokines in preclinical studies,^54^ and for the synthesis of antibody-drug conjugates.^55^ Libraries with N-terminal Ser have been previously converted to peptide-aldehydes and modified by oximes and hydrazines,^56^ benzamidoxime,^57^ or Wittig reaction,^58^ and used for the selection of diverse chemically-modified peptide ligands.^59–63^ Our group has previously demonstrated that the bicyclic topology akin to the one described in Fig. 1B can be introduced into synthetic peptides using *C*_2_-symmetric azobenzene linkers with an aldehyde reactive oxime functionality and two thiol-reactive chlorobenzyl functionalities.^64^ We demonstrated the feasibility of such bicyclization in several unprotected synthetic peptides with N-terminal Ser and two Cys residues in aqueous and organic solvents.^64^ In this report, we extend the previously published concept other classes of linkers biocompatible aqueous environment. We then provide the first example of using this technology for bicyclization of bacteriophage-displayed libraries with N-terminal aldehyde residues (Fig. 1B). To demonstrate the value of such a library in discovering new bioactive bicycles, we employed this library to discover inhibitors of protein NODAL and antagonists of NODAL-induced signaling.

The extracellular embryonic morphogen NODAL belongs to the transforming growth factor-beta (TGF-β) superfamily.^65^ It is a stem-cell-associated factor that has emerged as a putative target for the treatment of cancer.^66,67^ NODAL is normally restricted to embryogenesis, wherein it maintains pluripotency in the epiblast and governs the formation of the body axis and left-right asymmetry.^65^ After development, NODAL is relatively restricted to reproductive cell types and is not detectable in most normal adult tissues.^68^ However, NODAL expression re-emerges in a large number of divergent cancers.^69–79^ It also supports self-renewal in pancreatic and breast cancer stem cells and is enriched in melanoma and colon cancer cells with stem cell properties.^69–71^ In almost every cancer studied thus far, the acquisition of NODAL expression is associated with increased tumorigenesis, invasion, and metastasis. NODAL exerts its function by binding to and activating the cell surface receptors ALK4 and ALK7 in cooperation with the co-receptors Cripto-1 (FDGF1) or Cryptic (CFC1) to form a ligand–receptor complex that leads to the phosphorylation of Smad2/3 and the transcription of target genes, including NODAL itself.^73^ The only available inhibitor of NODAL to date, monoclonal anti-NODAL antibody 3D1,^80,81^ has demonstrated success in preclinical models of melanoma and is currently undergoing further preclinical evaluation. Recently, Mandomenico and co-workers designed a bicyclic peptide that inhibited the interaction between ALK4 and Cripto-1.^14^ No small-molecule or macrocyclic inhibitors of NODAL are presently available. In this manuscript, we employed bicyclic phage libraries to discover the first-in-class bicyclic ligands for NODAL protein. These ligands antagonize NODAL-induced signaling and specifically suppress NODAL-promoted proliferation of cancer cells. Evaluation of these antagonists benefited from the unique topology of the macro-bicycles that masked the N-terminus and equipped these macro-bicycles with multi-day stability in serum-rich cell culture media.

## Results and discussion

### Optimization of bicyclization on unprotected synthetic peptides

**Fig. 2:**
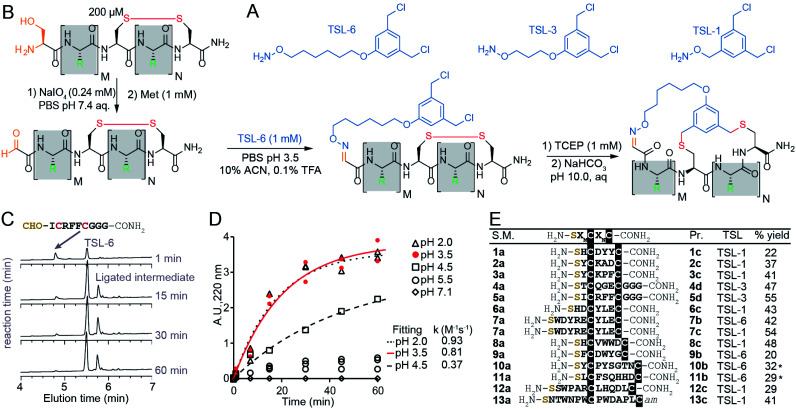
Macrocyclization reaction of bicycles with model peptides. (A) Chemical structure of **TSL**s. (B) Ligation of disulfide peptides with **TSL-6** at pH 3.5 and further macrocyclization into bicyclic peptides at pH 10. (C) Liquid chromatography traces at 200 μM for the reaction between oxidated **5a** and **TSL-6**. The reaction reaches 95% completion in 1 hour. (D) Kinetic traces of the reaction between oxidated **5a** and **TSL-6** at different pH. Reaction rates at pH 2.0, pH 3.5, and pH 4.5 were fit to pseudo first order kinetic equation to determine *k* values. (E) Isolated yields of bicyclic peptides with various sequences and different **TSL**s. The bicycles modified with **TSL-6**, **TSL-1** and **TSL-3** were denoted as #**b**, #**c** and #**d** respectively (*see ESI pages S20–S21† for details of the modification protocol).

The chemical linkers **TSL-1**, **TSL-3**, and **TSL-6** containing aminooxy and benzyl chloride functional groups (Fig. 2A) were synthesized (Scheme S1†) and tested for their ability to modify a series of unprotected peptides of structure SX_*n*_CX_*m*_C where X is any amino acids except Cys and *n* + *m* ranges from 4 to 11. To mimic the conditions that would be suitable for modification of phage-display library of peptides, we used model peptides at a micromolar concentration in aqueous buffers and treated them with super-stoichiometric reagents (Fig. 2B). Fig. 2C and D describe monitoring of the oxime formation progress. A representative model peptide SICRFFCGGG (200 μM) and NaIO_4_ (2.4 mM) reacted to form the N-terminal oxoaldehyde. Quenching the excess of NaIO_4_ with an excess of methionine, and addition of 1 mM **TSL-6** while decreasing the pH, led to the formation of the oxime (Fig. 2B). At pH ranging from 2.0 to 3.5, the rate constant of this ligation was *k* = 0.81–0.93 M^−1^ s^−1^ (Fig. 2C and D). In these conditions, oxime ligation went to completion within 1 hour. Increasing the pH to 4.5 decreased the rate (*k* = 0.37 M^−1^ s^−1^) and led to partial completion in 1 hour (Fig. 2D). Little to no oxime was formed at a pH higher than 5.5 (Fig. 2D). We note that aniline can catalyze oxime reactions;^56,82^ however, we avoided aniline and other nucleophilic catalysts to prevent the formation of byproducts with **TSL**s.^64^ The addition of 1 mM TCEP to the ligated product reduced the disulfide linkage. Raising the pH to 10 led to bicyclization of peptides in 3 hours. We note that this specific sequence of reactions—oxidation and aldehyde ligation followed by bicylization *via* an Sn2 reaction between thiols and chlorobenzyl—was based on previously optimized route to bicyclic peptides.^64^ Switching the order of steps is possible but it should be done with caution: when oxidation of N-terminal Ser to aldehyde is performed after formation of thioether the oxidation of relatively electron rich benzyl thioethers to sulfoxides may take place.^64,83^ We also observed sluggish linker- and sequence-dependent bicyclization when oxime ligation was used in place of thioether formation as the last ring-closing step.^64^

The reaction sequence described in Fig. 2B successfully produced 14 unique bicycles of different spacing between the Ser and Cys residues with an average isolated yield of 40% (Fig. 2E). Monitoring of the step-by-step synthesis for these and other bicycles are available in ESI (Schemes S2–S35†) and are summarized in Table S1.† We note that bicyclization of peptides can proceed at pH 10 (Schemes S7–S9 and S21–S31†), pH 8.5 (Schemes S3–S6, S10–S18 and S32–S35†) as well as pH 8.0 (Schemes S19–S20†). The model peptides were either chosen at random (**1a–3a**, **6a–7a**) or selected from the phage-displayed peptide library (**4a–5a**, and **10a–11a**). Two peptides (**12a–13a**) were adapted from a previous publication.^40^ Table S2† further highlights the various physicochemical properties of these peptides. We compared the yields of this reaction to modification of peptides with other reagents such as pentafluorophenyl sulfide (**PFS**),^84^ 1,3,5-tris(bromomethyl)benzene (**TBMB**)^26^ and α,α′-dibromo-*m*-xylene (**DBMB**).^17^**PFS** cyclized peptides had an average yield of 35.5% (Scheme S36 and Table S3†). **TBMB** cyclized peptides had an average yield of 35% (Schemes S37, S38 and Table S3†). **DBMB** cyclized peptides had an average yield of 31% (Table S3†). In conclusion, the aqueous biocompatible modification of peptides with **TSL** effectively produced bicyclic peptides with comparable yields with other reagents used in peptide cyclization or bicyclization. Although oxime linker is known to be reversible, we observed these bicycles to be stable in aqueous ammonium acetate (pH 4.7), in PBS (pH 7.4), and in Tris buffers (pH 8.5) for a month at room temperature (Fig. S1†).

### Modification of phage display libraries

**Fig. 3:**
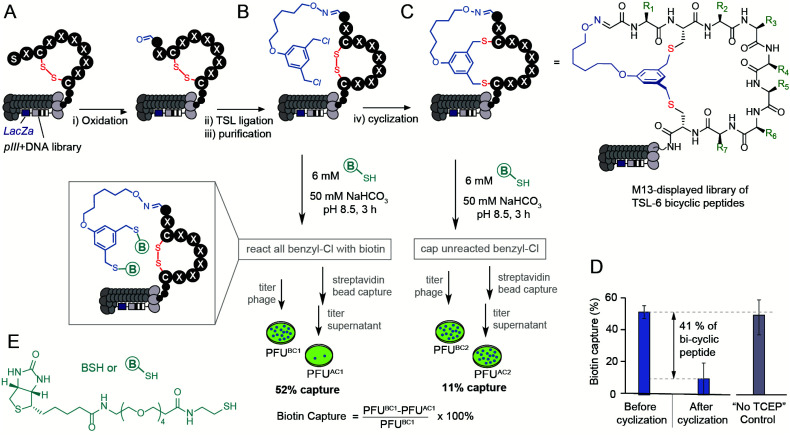
Modification of the library with a diversity of ∼10^9^ peptides displayed on phage by the **TSL-6**. (A and B) M13 phage-displayed disulfide library was oxidated and ligated with **TSL-6**. (C) The **TSL-6** ligated peptides were further converted into bicyclic peptides. Reaction conditions: (i) 0.06 mM NaIO_4_, pH 7.9, 9 min, ice. 0.5 mM Met, 20 min, r.t. (ii) 1 mM **TSL-6**, 10% MeCN, 0.1% TFA, 1 h, r.t. (iii) Zeba™ column, elute with 10 mM NaAc buffer, pH 4.6 (iv) 1 mM TCEP in 10 mM NaAc buffer, pH 4.6, 30 min. Increase the pH to 10 by adding 1 M NaHCO_3_ and incubate for 3 h, r.t. (D) Quantification of the phage with thiol-reactive groups before and after cyclization. Control incubation of **TSL-6**-ligated phages in pH 10 buffer for 3 h did not lead to a significant decrease of thiol-reactive group content. (E) Chemical structure of the biotin-thiol (BSH) probe.

The bicyclization approach described above was compatible with the modification of the phage-displayed peptide libraries. To quantify the efficiency of bicyclization reaction in phage libraries, we employed biotinylation and phage capture steps with similar approaches in previous publications (Fig. 3).^56–60^ Previously, the formation and reactivity of aldehyde in phage libraries were quantified by exposing the library to an aldehyde-reactive aminooxybiotin (AOB) and counting the number of biotinylated particles captured by streptavidin paramagnetic particles (“AOB capture,” Fig. S2C†).^56^ Using the reported oxidation conditions, we exposed a phage displaying SX_1_CX_2_X_3_X_4_X_5_X_6_X_7_C library with a diversity of ∼10^9^ peptides to an ice-cold solution of NaIO_4_ (60 μM in PBS) for 9 min, quenched the oxidation by 0.5 mM methionine for 20 min and used AOB capture to confirm that 93 ± 11% of the library was converted to aldehyde. Reacting with 1 mM solution of **TSL-6** at pH 3.5 for 1 hour consumed most of the aldehyde functionalities (Fig. S2E†). After removing excess **TSL-6** by size exclusion spin column, we exposed the phage to a biotin-thiol reagent (BSH, Fig. 3E) and captured the biotinylated clones by streptavidin paramagnetic particles. This “BSH capture” confirmed that 52 ± 4% of the library contained thiol-reactive benzyl chloride groups (Fig. 3B). Exposure of phage to TCEP and then pH 10 buffer completed bicyclization as evidenced by the decrease in BSH capture. In the control condition, incubation of the **TSL-6** ligated library at pH 10 in the absence of TCEP did not lead to any decrease in BSH capture, indicating that the number of benzyl chloride groups on phage remained unchanged in the absence of TCEP. We estimated 41 ± 13% of the library to be converted to **TSL-6**-bicyclic library (Fig. 3D). Detailed calculation of the conversion percentage can be found in Fig. S3.† Similar monitoring the modification of the SX_1_CX_2_X_3_X_4_X_5_X_6_X_7_C library with **TSL-1** and **TSL-3** (Fig. S4†) and the SX_1_CX_2_X_3_X_4_C phage with **TSL-6** (Fig. S5†) demonstrated a generality of this approach. Although modification of synthetic peptide proceeds effectively in pH 8.0–10.0 range, we observed that modification of libraries at pH 10 was more reliable. The ligation condition showed minor effects on the infectivity of the phage (Fig. S3F†). To confirm the chemical modification did not compromise the integrity of the phage DNA; we performed PCR (Fig. S6 and 7†) of the library and deep sequenced the PCR amplicons to monitor the sequence diversity of the library before and after chemical modification.

If chemical modification significantly damaged the DNA, we anticipated observing a change in the library composition. As the composition of the library before and after the modification remained the same (Fig. S8–S10†), we concluded that the modification did not impact the diversity of the phage library and did not impact the integrity of the phage DNA. These studies collectively demonstrate the construction of a bicyclic library that offers the potential for discovering bicyclic ligands for any target using canonical selection approaches.

### Selection of bicycles that bind to NODAL

**Fig. 4:**
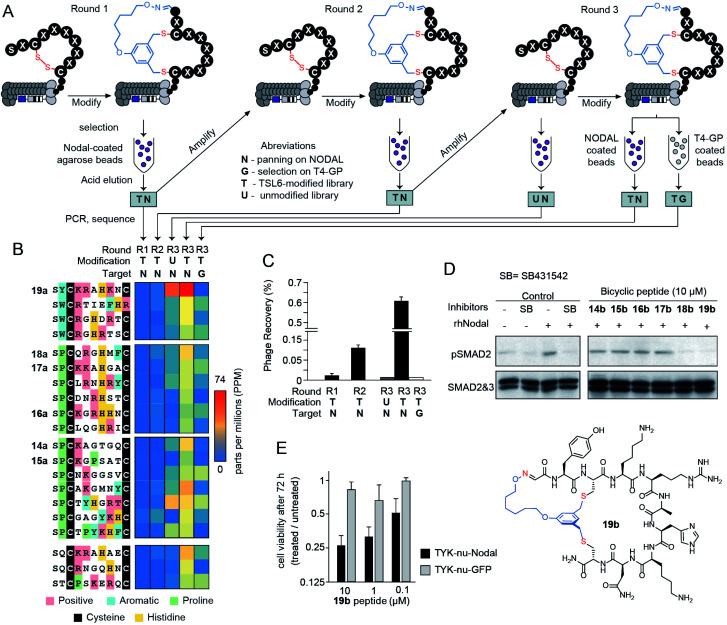
Bio-panning against the NODAL protein. (A) A scheme of three-rounds panning against NODAL and negative controls. (B) The top 20 sequences from the deep sequencing results were clustered into 4 groups and 6 of them were chemically synthesized. (C) Percentage of the phage recovery after each round of bio-panning. (D) Western blot validating with p-SMAD2 in response to treatment with rhNODAL and inhibitors in P19 cells. Total SMAD used as control (E) cell viability assay of TYK-nu cell line transfected with rhNODAL or GFP and treated with **19b** at various peptide concentrations for 72 hours.

We applied a **TSL-6**-modified phage-displayed SX_1_CX_2_X_3_X_4_X_5_X_6_X_7_C library to discover a ligand for the morphogen NODAL. We performed three rounds of phage selection using His_6_-tagged NODAL protein as bait. In between rounds of selection, we raised the stringency by increasing the number of washes and reducing the amount of immobilized NODAL protein (Fig. 4A). In round 3, we also performed two control selections; in the first control, we panned the unmodified R3 library against the NODAL protein (R3-UN) and in the second control, we panned the **TSL-6**-modified R3 library against unrelated His_6_-tagged protein (R3-TG). Phage recovery increased by 4-fold in R3 when compared to R1 and R2. This recovery was ablated by 20-fold when the unmodified round 3 library was panned against NODAL (R3-UN) and when the **TSL-6**-modified library was panned against an unrelated protein (R3-TG) (Fig. 4C). Deep sequencing the output of all selection rounds and the control experiments identified families of sequences that exhibited high normalized abundance in R3 and low normalized abundance in R1, R2, and the control experiments R3-UN, and R3-TG (Fig. 4B and S11B†). From these families, we selected six representative sequences for further validation (**14a–19a**; Fig. 4B and S11B†).

### Validation of NODAL bicyclic inhibitors

The bicycles **14b–19b** were chemically synthesized and tested for their ability to antagonize NODAL-induced signalling in the P19 cells: a model cell line known to respond to NODAL.^85^ Stimulations of the P19 cells with rhNODAL at 100 ng mL^−1^ for 1 hour led to the phosphorylation of SMAD2 (Fig. 4D, column 3). This phosphorylation was inhibited by ALK4/7 kinase inhibitor SB431542 (Fig. 4D, column 4), as previously reported.^85^ Bicyclic peptides **14b–19b** at 100 μM were able to inhibit rhNODAL-induced phosphorylation of SMAD2 (Fig. S12A†). At the concentration of 10 μM, bicyclic peptides **18b** and **19b** inhibited phosphorylation of SMAD2 (Fig. 4D, columns 9 and 10), whereas bicyclic peptides **14b–17b** exhibited no inhibition (Fig. 4D, columns 5–8 and Fig. S12B†). As **19b** exhibited robust and reproducible inhibition of phosphorylation (Fig. S12B†), we further tested the ability of **19b** to suppress the NODAL-induced proliferation of ovarian cancer cells. We transfected ovarian cancer cells (TYK-nu) with a plasmid vector containing human NODAL and used a GFP transfected TYK-nu cell line as an isotype control. TYK-nu-NODAL and TYK-nu-GFP cell lines were cultured in the presence and absence of **19b** for 72 hours (Fig. S13†). Treatment of TYK-nu-GFP cell with **19b** at 10 μM had no effect on the proliferation, whereas the viability of TYK-nu-NODAL cells was reduced to 23% compared to untreated TYK-nu-NODAL cells (Fig. 4E). The response to **19b** was dose-dependent with apparent IC_50_ between 0.1 and 1 μM **19b** (Fig. 4E and S14B†). The discovery of **19b** served as a promising starting point for developing more potent NODAL antagonists.

### Proteolytic stability of bicycles

**Fig. 5:**
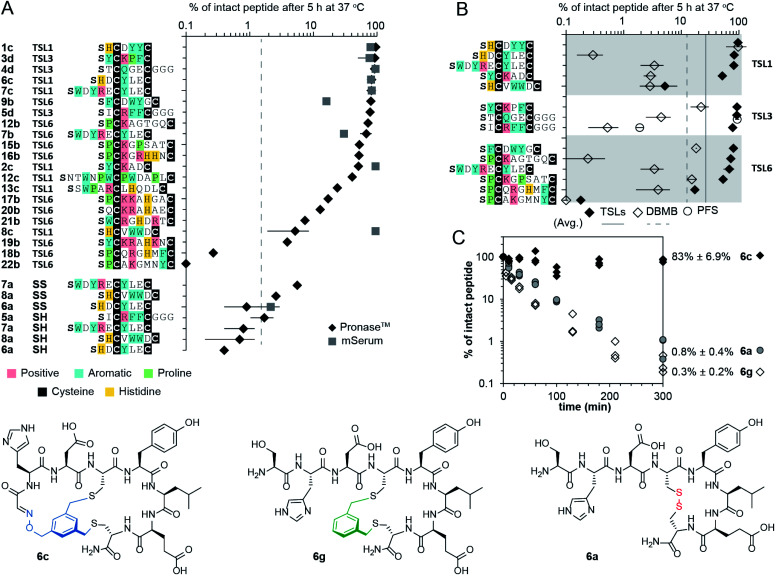
Proteolytic stability of bicycles and controls. (A) Stability of **TSL**s bicycles, disulfide constrained peptides, and linear peptides in the presence of Pronase™ and mouse serum for 5 hours at 37 °C. (B) Stability of peptides modified with **TSL**s, **DBMB** and **PFS** in the presence of Pronase™ for 5 hours at 37 °C. (C) Stability of **6a** (disulfide-bonded), **6c** (bicycled with **TSL-1**), and **6g** (macro-cyclized with **DBMB**) in the presence of Pronase™.

Intrinsic proteolytic stability of the bicyclic scaffold was critical to the evaluation of the NODAL antagonist in the aforementioned cell-based assays. Specifically, we found that 53% of the bicyclic peptide antagonist **19b** remained intact after 72 hours of incubation at 37 °C in a serum-rich culture medium (Fig. S15†). We followed up on this observation and tested the stability of a panel of bicyclic scaffolds in two proteolytic degradation conditions (Fig. 5). In the first condition, we highlighted that 25–90% of the bicycles remained intact after 5 hours of exposure to Pronase™ (Fig. 5A and S16–S22†). In these conditions, all the tested linear and monocyclic disulfides degraded to <1%. In the second condition, nine of these bicycles were exposed to fresh mouse serum at 37 °C. On average, 72% of the starting peptide amount was intact after 5 hours (Fig. 5A, S23 and S24†). Monocyclic peptides formed by modifying peptides with **DBMB**,^17,86^ which have the same topology as one of the rings in **TSL**-modified bicycles. We observed that on average, 13% of the **DBMB** macrocycles remained intact after 5 hour treatment by Pronase™, compared to 62% from the **TSL**s-modified set (Fig. 5B and S25–S28,† the values represent average from the set of *n* = 14 sequences modified by both **DBMB** and **TSL**s). Fig. 5C represents an example of bicycle **6c** that remained 83 ± 6.9% intact after 5 h of incubation in Pronase™; the **DBMB** macrocycle **6g** and the disulfide precursor **6a** degraded to <1% under the same conditions. We tested the stability of two sequences modified with the **PFS** cross-linker developed by the Pentelute Lab.^84^ In Pronase™, macrocycle **PFS**-STCQGECGGG and bicycle **TSL-3**-STCQGECGGG exhibited similar stabilities, whereas macrocycle **PFS**-SICRFFCGGG exhibited lower stability than bicycle **TSL-3**-SICRFFCGGG (Fig. 5B, S29 and S30†). Due to differences in the shape of the cross-linkers resulting in different conformations of peptides, the results were difficult to interpret, and we did not expand on this comparison further. In general, it is not trivial to quantify the advantages of a peptide cross-linkers in comparison to the other available cross-linkers to-date; however, a comparison of a set, *n* = 14, peptides modified with closely related **DBMB** and **TSL** linkers indeed suggests that the bicyclization yields a significant improvement in stability.

### Molecular dynamics simulation of bicycle structures

In testing the stability of a large, diverse set of bicycles, we observed preliminary linker-dependent and sequence dependent trends in degradation. For example, Pronase™ degradation of peptide SWDYRECYLEC modified with **TSL-1**, or **TSL6** linker yielded minor but statistically significant differences: 82 ± 13% and 68 ± 14% intact bicycles after 5 hours (Fig. 5A). To explore these differences, we employed molecular dynamics (MD) simulation of the conformational ensemble of these bicycles. The penultimate amino acids in **TSL-1**-SWDYRECYLEC and **TSL-6**-SWDYRECYLEC bicycles yielded different Ramachandran plots describing the dihedral angles for –WDYR– sequences in the first ring. On the other hand, the dihedral angle populations for the –CYLEC– sequence in the second ring were similar (Fig. S31†). The MD simulation suggested that conformations of two rings are decoupled from one another. Thus, differences in degradation for two bicycles, might originate from the enhanced flexibility in one of the rings. Similar decoupling was observed in **TSL-1**-SHCVWWDC and **TSL-6**-SHCVWWDC bicycles. The penultimate amino acid, His, exhibited different clustering of the dihedral angles. On the other hand, –VWWD– sequence in the second ring had similar backbone conformations in both bicycles (Fig. S31†). These studies provide an important starting point for understanding the ground-state conformational ensemble of these molecules.

## Conclusions

In conclusion, two-fold symmetric tridentate linchpins that contain aldehyde and two thiol-reactive groups enable a robust one-pot bicyclization of peptides SX_*n*_CX_*m*_C. Such libraries can be used to discover productive antagonists of protein–protein interactions. The bicycles show good stability in digestive conditions. Although the 21 bicyclic peptide sequences tested do not exhaustively sample all possible combinations, the tested peptides included all the potentially problematic amino acids (Lys, Arg, His, Tyr, Trp, Asp/Glu, Ser/Thr). Proteolytic stability of bicyclic architecture sans a free N-terminus is significantly improved when compared to closely-related **DBMB**-cross-linked monocycles. As the strategy is compatible with phage display libraries containing the SX_*n*_CX_*m*_C motif, we anticipate that other peptide libraries that contain this motif will be amenable to such late-stage functionalization. We noted that many genetically-encoded libraries do not contain N-terminal Ser and instead have an N-terminal Met or Met analogs encoded by AUC starting codon. However, it is possible to introduce an N-terminal Ser into these systems by expressing a library with N-terminal TEV-cleavable sequence: H-MENLYFQ\S (where\denoted as the cleavage site). A conceptually similar approach has been recently demonstrated by Jianmin Gao and co-workers who expressed ENLYFQ\C in phage-displayed peptide libraries and used TEV cleavage to expose the N-terminal Cys.^87^ Finally, the lower symmetry of the **TSL**-style linkers allows their diversification with any chemotype of *C*_2_-symmetry.^64^ It offers a significant expansion of the bicyclization repertoire beyond traditional architectures produced from three-fold symmetric cross-linkers.

## Data availability

The datasets supporting this article have been uploaded as part of the supplementary material. All the kinetic data and MATLAB scripts are available at Data.zip/Kinetic. The deep sequencing data with DNA reads, raw counts, were uploaded to http://48hd.cloud/ server with an unique alphanumeric name (*e.g.*, 20181108-16TSooPA-YW) and an unique static URL can be found in the ESI section 3.6.

## Author contributions

R. D. conceived and designed the project. R. D., L-M. P., Y-S. L. A. H. and J. D. supervised the project. J. W. and R. M. synthesized bicycles and preformed proteolytic stabilities studies. S. K., V. I., and D. V. optimized the scale up reactions. M. M. preformed NMR studies and analysis. J. W. and V. T. preformed chemical modification of phage-displayed peptide libraries. J. W. screened and validated NODAL ligands. O. B. provided TYK-nu cell. J. M. and Y-S. L. preformed molecular dynamics studies. J. W. and R. D. wrote the manuscript with editing from all authors.

## Conflicts of interest

Patent application describing this invention was filed by TEC Edmonton in July 2018. R. D. is a shareholder of 48Hour Discovery Inc., the company licensing the technology.

## Supplementary Material

SC-012-D1SC01916C-s001

SC-012-D1SC01916C-s002

SC-012-D1SC01916C-s003

SC-012-D1SC01916C-s004

SC-012-D1SC01916C-s005

SC-012-D1SC01916C-s006

SC-012-D1SC01916C-s007

SC-012-D1SC01916C-s008

SC-012-D1SC01916C-s009

SC-012-D1SC01916C-s010

SC-012-D1SC01916C-s011

SC-012-D1SC01916C-s012

SC-012-D1SC01916C-s013

SC-012-D1SC01916C-s014
