# Supplementary figures and images for: Genetically-encoded discovery of proteolytically stable bicyclic inhibitors for morphogen NODAL
Source: Chem Sci. 2021 Jun 17;12(28):9694–703. doi: 10.1039/d1sc01916c (PMC8294009; doi:10.1039/d1sc01916c)

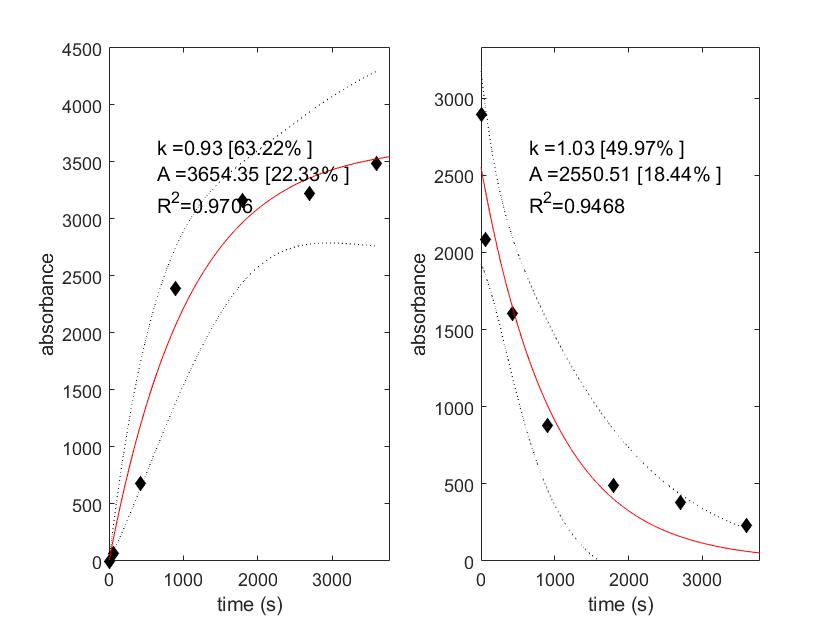

Supplement: SC-012-D1SC01916C-s001 [file SC-012-D1SC01916C-s001.jpg]

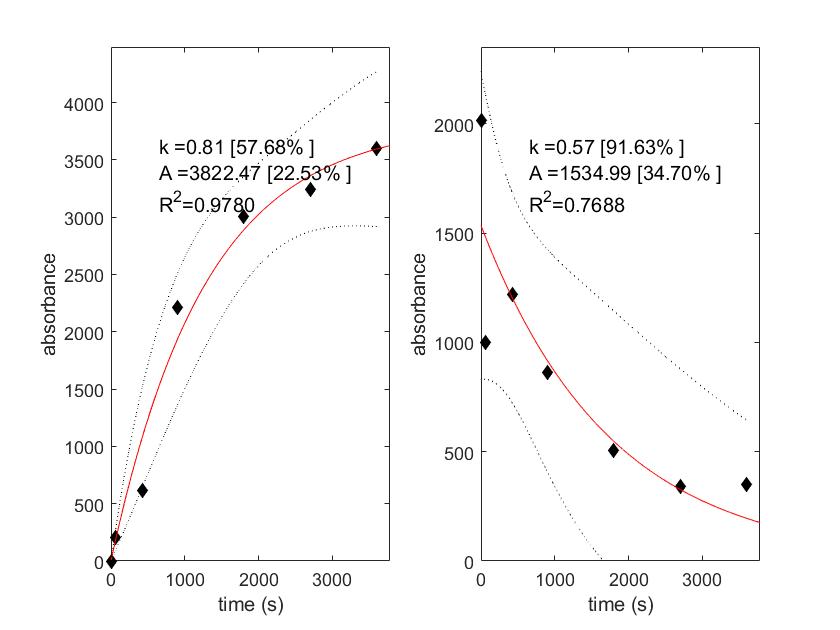

Supplement: SC-012-D1SC01916C-s002 [file SC-012-D1SC01916C-s002.jpg]

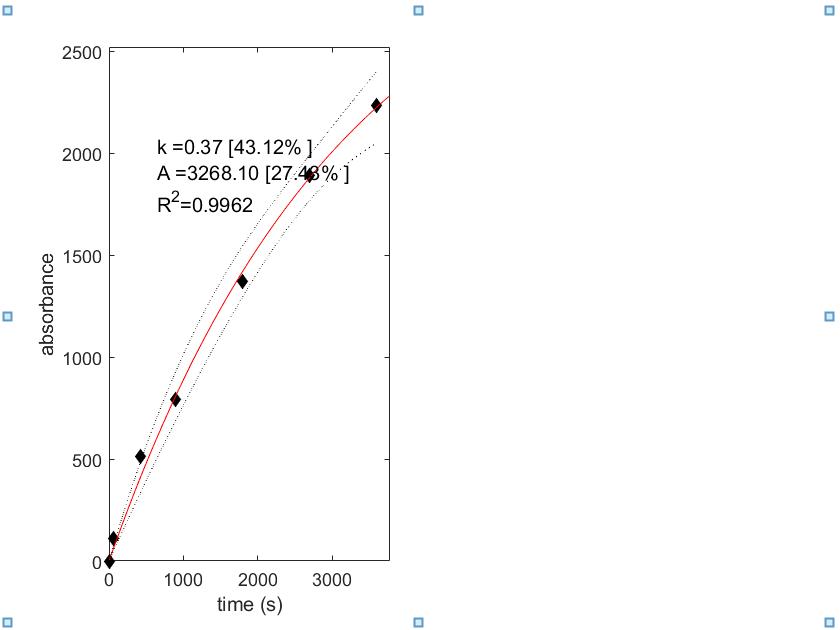

Supplement: SC-012-D1SC01916C-s003 [file SC-012-D1SC01916C-s003.jpg]
