# Supplementary material for: Genetically-encoded discovery of proteolytically stable bicyclic inhibitors for morphogen NODAL
Source: Chem Sci. 2021 Jun 17;12(28):9694–703. doi: 10.1039/d1sc01916c (PMC8294009; doi:10.1039/d1sc01916c)
Supplement: SC-012-D1SC01916C-s014 [file SC-012-D1SC01916C-s014.pdf]

Electronic Supplementary Material (ESI) for Chemical Science.
This journal is © The Royal Society of Chemistry 2021

Supporting Information for:

Genetically-Encoded Discovery of Proteolytically Stable Bicyclic Inhibitors for Morphogen NODAL

Jeffrey Y.-K. Wong,[a] Raja Mukherjee,[a] Jiayuan Miao,[b] Olena Bilyk,[c] Vivian Triana,[a] Mark Miskolzie,[a] Antoine Henninot,[d] John J. Dwyer,[d] Serhii Kharchenko,[e] Anna Iampolska,[e] Dmitriy M. Volochnyuk,[e] Yu-Shan Lin,[b] Lynne-Marie Postovit[c] and Ratmir Derda[a*]

a. Department of Chemistry, University of Alberta, Edmonton, AB T6G 2G2, Canada
b. Department of Chemistry, Tufts University, Medford, MA 02155, USA
c. Department of Experimental Oncology, University of Alberta, Edmonton, AB T6G 2G2, Canada
d. Ferring Research Institute, San Diego, California 92121, USA
e. Enamine Ltd., Chervonotkatska Street 78, Kyiv 02094, Ukraine

*Corresponding author: ratmir@ualberta.ca

Table of Contents

List of abbreviations 6

1. Chemistry Methods 7

1.1. *General Chemistry information* 7
Scheme S1: Synthetic procedures for the linchpins **TSL-1**, **TSL-3** and **TSL-6**: 8
1.2. *Synthetic procedures for the linchpins **TSL-1**, **TSL-3** and **TSL-6**:* 9
1.3. *General procedure for peptide synthesis* 15
1.4. *Protocol 1: Bicyclization of Peptides SX_mCX_nC with **TSL** using C18 spin column.* 16
1.5. *Protocol 2: Bicyclization of Peptides SX_mCX_nC with **TSL** using methionine as quencher* 17
1.6. *General procedure for one-pot bicyclization on semi-preparative scale* 18
1.7. *General Protocol for bicyclization with **TBMB*** 18
1.8. *General Protocol for cyclization with perflurodiphenylsufide (**PFS**)* 18
1.9. *General Protocol for cyclization with α,α'-Dibromo-m-xylene (**DBMB**)* 18
1.10. *General bicyclization analytical procedure for **10b** and **11b**:* 19
1.11. *Protocol for **10b** scale up synthesis:* 20
1.12. *Protocol for **11b** scale up synthesis:* 21
Scheme S2: One-pot bicyclization of **5a** (0.2 mmol) with **TSL-6** 22
Scheme S3: One-pot bicyclization of **1a** (0.5 mM) with **TSL-1**. 23
Scheme S4: One-pot bicyclization of **2a** (0.5 mM) with **TSL-1**. 24
Scheme S5: One-pot bicyclization of **3a** (0.5 mM) with **TSL-1**. 25
Scheme S6: One-pot bicyclization of **3a** (0.5 mM) with **TSL-3**. 26
Scheme S7: One-pot bicyclization of **4a** with **TSL-3**: 27
Scheme S8: One-pot bicyclization of **5a** with **TSL-1**: 28
Scheme S9: One-pot bicyclization of **5a** with **TSL-3**: 29
Scheme S10: One pot bicyclization of **6a** (0.5 mM) with **TSL-6**. 30

Scheme S11: One-pot bicyclization of **6a** (0.5 mM) with **TSL-1**.. 31
Scheme S12: One-pot bicyclization of **6a** (0.5 mM) with **TSL-6**.. 32
Scheme S13: One-pot bicyclization of **7a** (0.5 mM) with **TSL-1**.. 33
Scheme S14: One-pot bicyclization of **7a** (0.5 mM) with **TSL-1**.. 34
Scheme S15: One-pot bicyclization of **8a** (0.5 mM) with **TSL-6**.. 35
Scheme S16: One-pot bicyclization of **8a** (0.5 mM) with **TSL-3**.. 36
Scheme S17: One-pot bicyclization of **8a** (0.5 mM) with **TSL-1**.. 37
Scheme S18: One-pot bicyclization of **9a** (0.5 mM) with **TSL-1**.. 38
Scheme S19: Bicyclization of **10a** (10 mg, 84 nmol) with **TSL-6** 39
Scheme S20: Bicyclization of **11a** (10 mg, 66 nmol) with **TSL-6** 40
Scheme S21: One-pot bicyclization of **12a** with **TSL-1**... 41
Scheme S22: One-pot bicyclization of **13a** with **TSL-1**... 42
Scheme S23: Bicyclization of **14a** with **TSL-6** 43
Scheme S24: Bicyclization of **15a** with **TSL-6** 44
Scheme S25: Bicyclization of **16a** with **TSL-6** 45
Scheme S26: Bicyclization of **17a** with **TSL-6** 46
Scheme S27: Bicyclization of **18a** with **TSL-6** 47
Scheme S28: Bicyclization of **19a** with **TSL-6** 48
Scheme S29: Bicyclization of **20a** with **TSL-6** 49
Scheme S30: Bicyclization of **21a** with **TSL-6** 50
Scheme S31: Bicyclization of **22a** with **TSL-6** 51
Scheme S32: One-pot bicyclization of **23a** (0.5 mM) with **TSL-6**.. 52
Scheme S33: One-pot bicyclization of **24a** (0.5 mM) with **TSL-6**.. 53
Scheme S34: One-pot bicyclization of **25a** (0.5 mM) with **TSL-6**.. 54
Scheme S35: One-pot bicyclization of **26a** (0.5 mM) with **TSL-3**.. 55
Scheme S36: Cyclization of **4a** and **5a** with **PFS** 56
Scheme S37: Bicyclization of **23a** with **TBMB** 57
Scheme S38: Bicyclization of **24a** with **TBMB**: 58
Scheme S39: Comparison between bicyclization of **8a** with **TSL-1** 59
Table S1: List of peptide sequences, **TSL**s and resulting bicyclic products 60
Table S2: Peptide sequence used in the study and their properties. 61
Table S3: Modifiers other than **TSL** and resulting bicyclic/monocyclic product 62
Figure S1: Stability test of bicyclic peptide **TSL-6-**SHCDYYC over 30 days in buffers of different pH.. 63

2. Phage Modification Methods 64

2.1. *Preparation of SXCX$_6$C phage libraries* 64
2.2. *General protocol for modification of SXCX$_6$C phage library:* 64
Figure S2: Modification of the library of 10^8 peptides displayed on phage by the **TSL-6.**. 66
Figure S3: Composition of SXCX$_6$C library during modification with **TSL-6** 67
Figure S4: Modification of the SXCX$_6$C library by the **TSL-1** and **TSL-3** 68

Figure S5: Modification of monoclonal phage displaying SICNQFC with **TSL-6**. 69
3. General Selection and Validation Methods 70
3.1. *General setting for panning on Kingfisher instrument* 70
3.2. *Bio panning of NODAL protein* 70
3.3. *General PCR amplification protocol for Illumina deep sequencing* 72
3.4. *Illumina sequencing of samples before and after panning* 72
3.5. *General data processing methods* 72
3.6. *Processing of Illumina data* 73
3.7. *General protocol for protein extraction* 73
3.8. *Western blotting protocol for detecting pSMAD2 protein level* 73
3.9. *General protocol P19 Cell Culture* 74
3.10. *Inhibition of pSMAD assay with P19 Cell* 74
3.11. *Transfect TYK-nu cell with constitutive NODAL and GFP* 74
3.12. *Cell Viability assay with TYK-nu-NODAL and TYK-nu-GFP* 74
Figure S6: DNA sequences of PCR amplification protocol for Illumina deep sequencing . 71
Figure S7: PCR product of **TSL-6** modification and 3 rounds of the NODAL panning. 75
Figure S8: 20 × 20 plot comparison before and after **TSL-6** modification in input library. 76
Figure S9: 20 × 20 plot comparison before and after **TSL-6** modification after R1 selection. 77
Figure S10: 20 × 20 plot comparison before and after **TSL-6** modification after R2 selection 78
Figure S11: Scheme of selection of NODAL bicycles and post-selection analysis of selection samples 79
Figure S12: Western blot analysis of p-SMAD2 80
Figure S13: CellTiter-Glo® Luminescent Cell Viability 600 cells/well 81
Figure S14: CellTiter-Glo® Luminescent Cell Viability 6000 cells/well 81
4. Proteolytic Stability Methods 82
4.1. *Protocol for measurement of proteolytic stability in cell assay:* 82
4.2. *Protocol for measurement of proteolytic stability in Pronase™ :* 82
4.3. *Protocol for measurement of proteolytic stability in fresh mouse serum:* 82
Figure S15: Peptide stability in active P19 cell culture for 72 hours of **19b** 82
Figure S16: Proteolytic stability of **7a**, **7b** and **7c** in Pronase™: 83
Figure S17: Proteolytic stability of **8a**, and **8c** in Pronase™ 84
Figure S18: Proteolytic stability of **6a** and **6c** in Pronase™. 84
Figure S19: Proteolytic stability of **9b**, **1c**, **2c** and **3c** in Pronase™. 85
Figure S20: Proteolytic stability of **5a**, **14b**, **15b**, and **16b** in Pronase™. 86
Figure S21: Proteolytic stability of **17b**, **18b**, **19b**, and **20b** in Pronase™. 87
Figure S22: Proteolytic stability of **21b** and **22b** in Pronase™. 88
Figure S23: Proteolytic stability of **1c, 2c, 3c**, **4d** and **5d** in fresh mouse serum 89
Figure S24: Proteolytic stability of **5d**, **6c**, **7b**, **7c**, **8c** and **8a** in fresh mouse serum 90

Figure S25: Proteolytic stability of **1g**, **2g**, **3g**, and **4g** in Pronase^{TM}. 91
Figure S26: Proteolytic stability of **5g**, **6g**, **7g**, and **8g** in Pronase^{TM}. 92
Figure S27: Proteolytic stability of **9g**, **13g**, **14g**, and **16g** in Pronase^{TM}. 93
Figure S28: Proteolytic stability of **22g** in Pronase^{TM}... 94
Figure S29: Proteolytic stability of **4d** and **4e** in Pronase^{TM}. 95
Figure S30: Proteolytic stability of **5d** and **5e** in Pronase^{TM}. 95

5. Molecular Dynamics Simulation 96

Table S4: Populations of the top 10 clusters of **8c**, **8b**, **7c**, and **7b** using the torsional angles in cycle 1 in the cluster analysis. 97
Table S5: Populations of the top 10 clusters of **8c**, **8b**, **7c**, and **7b** using the torsional angles in cycle 2 in the cluster analysis. 97
Figure S31: Ramachandran plot of the cyclic peptide backbone for **8c**, **8b**, **7c** and **7b**....... 98

6. Summary of synthesis 99

6.1. *Summary of* ***TSL****s Peptides Synthesis* 99
Figure S32: Summary for **1c** synthesis.. 99
Figure S33: Summary for **2c** synthesis.. 100
Figure S34: Summary for **3c** synthesis.. 101
Figure S35: Summary for **4d** synthesis 102
Figure S36: Summary for **5d** synthesis 103
Figure S37: Summary for **7c** synthesis.. 104
Figure S38: Summary for **7b** synthesis 105
Figure S39: Summary for **8c** synthesis.. 106
Figure S40: Summary for **9b** synthesis 107
Figure S41: Summary for **13c** synthesis.. 108
Figure S42: Summary for **12c** synthesis.. 109
Figure S43: Summary for **15d** synthesis 110
Figure S44: Summary for **16b** synthesis 111
Figure S45: Summary for **18b** synthesis 112
Figure S46: Summary for **19b** synthesis 113
Figure S47: Summary for **21b** synthesis 114
Figure S48: Summary for **22b** synthesis 115

6.2. *Summary of* ***PFS*** *Peptides Synthesis* 116
Figure S49: Summary for **4e** synthesis.. 116
Figure S50: Summary for **5e** synthesis.. 117

6.3. Summary of **DBMB** Peptides Synthesis.. 118
Figure S51: Summary for **1g** synthesis 118
Figure S52: Summary for **2g** synthesis 119
Figure S53: Summary for **3g** synthesis 120
Figure S54: Summary for **4g** synthesis 121
Figure S55: Summary for **5g** synthesis 122

Figure S56: Summary for **6g** synthesis 123
Figure S57: Summary for **7g** synthesis 124
Figure S58: Summary for **8g** synthesis 125
Figure S59: Summary for **9g** synthesis 126
Figure S60: Summary for **12g** synthesis 127
Figure S61: Summary for **14g** synthesis 128
Figure S62: Summary for **15g** synthesis 129
Figure S63: Summary for **16g** synthesis 130
Figure S64: Summary for **19g** synthesis 131
Figure S65: Summary for **20g** synthesis 132
Figure S66: Summary for **22g** synthesis 133
7. NMR spectra 134
7.1. *NMR spectra for **TSL-1**, **TSL-3** and **TSL-6*** 134
7.2. *Proton NMR assignment and corresponding NMR spectra of 7**c*** 147
7.3. *Proton NMR assignment and corresponding NMR spectra of **3c*** 161
References: 171

List of abbreviations:

AOB	aminooxy-biotin
BIA	biotin-PEG2-iodoacetamide
Boc	*tert*-butyloxycarbonyl
BSH	biotin-thiol
Calc	calculated
COSY	correlation spectroscopy
Da.	daltons(s)
DBU	1,8-Diaza-bicyclo (5.4.0) undec-7-en
DCM	dichloromethane
DMF	N, N-Dimethylformamide
ESI	electrospray ionization
eq.	equivalent(s)
EDT	1,2-ethanedithiol
h	hour(s)
HPLC	high performance liquid chromatography
HRMS	high-resolution mass spectrometry
LCMS	liquid chromatography mass spectrometry
MHz	megahertz
MsCl	methanesulfonyl chloride
NMR	nuclear magnetic resonance
NOESY	nuclear overhauser effect spectroscopy
DBMB	α,α′-Dibromo-m-xylene
mL	milliliter(s)
mM	millimolar
min	minute(s)
mmol	millimoles
PBS	phosphate buffered saline
PFS	pentafluorophenyldisulfide
ppm	parts per million
ROESY	rotating frame overhauser effect correlation spectroscopy
rt	room temperature
TBMB	1,3,5-Tris(bromomethyl)benzene
TBS	tris-buffered saline
TBST	tris-buffered saline, w. 0.1% Tween 20
TCEP	tris(2-carboxyethyl)phosphine)
TIPS	triisopropylsilane
TFA	trifluoroacetic acid
TLC	thin layer chromatography
Tris	tris(hydroxymethyl)aminomethane
TOCSY	total correlation spectroscopy
TSL	two fold symmetric tridentate lincpin
v/v	volume/volume

1. Chemistry Methods

1.1. *General Chemistry information*

Chemical reagents and solvents were purchased from Sigma-Aldrich or Fisher Scientific unless noted otherwise. 5-hydroxydimethyl isophthalate, 1,6-dibromohexane and 1,3-dibromopropane were purchased from TCI America. 1,3,5-Trisbromomethyl benzene was purchased from Synthonix. TCEP was purchased from Soltech Ventures. Pronase was purchased from Roche Diagnostics GmbH. Reagents for peptide synthesis were purchased from ChemPep. Reactions were monitored by TLC which was carried out on silica gel 60 F_{254} (Merck) plates and visualized by UV-light (λ = 254 nm) and/or by spraying potassium permanganate, anisaldehyde followed by heating. Flash column chromatography was performed using silica gel 60 (40-63 μm). The subsequent evaporation of solvents *in vacuo* was performed using IKA RV10 rotary evaporator. Analytical and preparative HPLC was conducted using Waters 1525 Binary pump equipped with a Waters Symmetry prep 19×50 mm C18 Columns and Waters 2489 UV detector. Removal of aqueous solvents was performed using Labconco Freezone 2.5w system.

Proton (^{1}H NMR) and Carbon (^{13}C NMR) nuclear magnetic resonance spectra were recorded on an Agilent/Varian VNMRS two channel 500 MHz or Agilent/Varian Inova two-channel 400 MHz spectrometer. The chemical shifts are given in part per million (ppm) on the delta scale. The solvent peak was used as reference values. For ^{1}H NMR: $CDCl_3$= 7.26 ppm and for ^{13}C NMR: $CDCl_3$= 77.16 ppm. The following abbreviations have been used: s, singlet; d, doublet; t, triplet; m, multiplet.

LCMS analysis of peptide modifications was obtained on Agilent Technologies 6130 LCMS. A gradient of solvent A (MQ water) and solvent B ($MeCN/H_2O$ 95/5) was run at a flow rate of 0.5 mL/min (0-4.0 min 5% B; 4.0-5.0 min 5%→60% B; 5.0-5.5 min 60%→100% B; 5.5-7.5 100% B, 7.5-11 min 100%→5% B).

LCMS studies of stability of peptides in proteases and serum were performed in Hewlett Packard 1100 series instrument using a Phenomenex Jupiter C4 protein column (300A, 2×50 mm, 0.3 mL/min, A: 0.1% formic acid in water, B: 0.1% formic acid in acetonitrile (0 min 2% B, 0→10 min 2%→70% B, 10→15 min 70% B, 15→20 min 70%→2% B). The amount of peptide remaining was calculated with the area under the curve of SIM (Selected Ion Monitoring) peak in LCMS.

All the kinetic data and MATLAB fitting script are available at Data.zip/ kinetic.

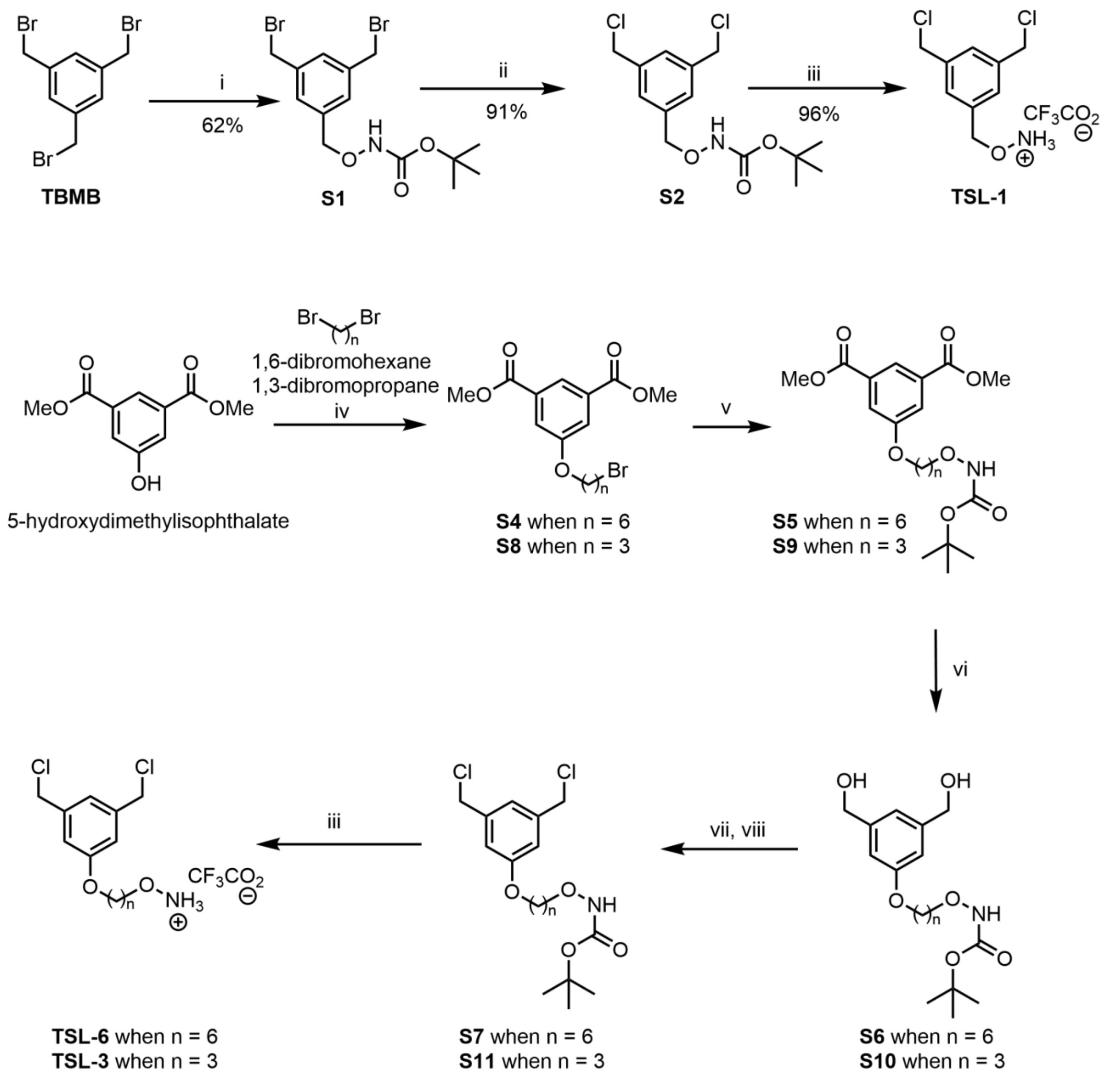

Scheme S1: Synthetic procedures for the linchpins **TSL-1**, **TSL-3** and **TSL-6**: Reagents and conditions: i) BocNHOH, DBU, DCM, 3 h; ii) LiCl, DMF, 10 h; iii) TFA, DCM; iv) 1,6-dibromohexane or 1,3-dibromopropan, K_2CO_3, CH_3CN, reflux, 72 h; v) BocNHOH, DBU, DCM, 5 h; vi) $LiAlH_4$, THF, 0 °C to rt, 1 h; vii) MsCl, Et_3N, 0 °C to rt, 5 min; viii) LiCl, DMF, 10 h.

*Synthetic procedures for the linchpins **TSL-1**, **TSL-3** and **TSL-6**:*

tert-Butyl ((3,5-bis(bromomethyl)benzyl)oxy)carbamate **S1**

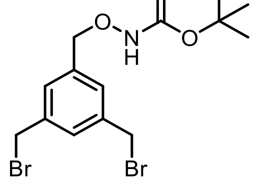

To a solution of 1,3,5-Trisbromobenzylbenzene (**TBMB**) (2.5 g, 7 mmol) in DCM (30 mL), an equimolar solution of N-Boc-hydroxylamine (306 mg, 2.3 eq.) and DBU (0.3 mL, 2.3 eq.) in DCM (5 mL) was added over the course of 30 min and the resulting solution was stirred for 3 h. The solvent was removed on a rotary evaporator and the crude residue was purified over silica gel chromatography using ethyl acetate-hexanes (1:4) as eluent producing the title compound **S1** as a white solid (707 mg, 25%): ^{1}H NMR (500 MHz, $CDCl_3$) δ = 7.37 (s, 1 H), 7.34 (s, 1 H), 7.33 (s, 2 H), 4.82 (s, 2 H), 4.44 (s, 4 H), 1.47 (s, 9 H). ^{13}C NMR (125 MHz, $CDCl_3$) δ = 156.73, 138.65, 137.18, 129.59, 129.38, 81.89, 77.58, 32.48, 28.20.HRMS (ESI) calculated for $C_{14}H_{19}Br_2NO_3Na$ $[M+Na]^+$ m/z=429.9629, found 429.9226.

tert-Butyl ((3,5-bis(chloromethyl)benzyl)oxy)carbamate **S2**

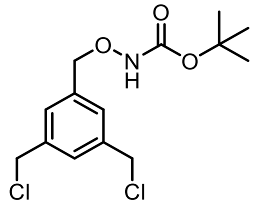

Lithium chloride (196 mg, 3 eq.) was added to a solution of **S1** (707 mg, 1.72 mmol) in DMF (10 mL) and the solution was stirred for 10 h. The reaction mixture was partitioned between ethyl acetate and water. The combined organic layers were washed with water and brine. The organic layer was dried over anhydrous sodium sulfate. After removing the solvent on a rotary evaporator, the crude residue was purified over silica gel chromatography using ethyl acetate-hexanes (1:4) as eluent producing the title compound **S2** as colorless oil (457 mg, 83%). ^{1}H NMR (400 MHz, $CDCl_3$) δ = 7.46 (s, 1 H), 7.37 (s, 1 H), 7.36 (s, 2 H), 4.82 (s, 2 H), 4.53 (s, 4 H), 1.46 (s, 9 H). ^{13}C NMR (100 MHz, $CDCl_3$) δ = 156.99, 138.51, 137.29, 129.14, 128.86, 82.04, 77.85, 45.70, 28.39. HRMS (ESI) calculated for $C_{14}H_{19}Cl_2NO_3Na$ $[M+Na]^+$ m/z=342.0640, found 342.0632.

O-(3,5-bis(chloromethyl)benzyl)hydroxylammonium 2,2,2-trifluoroacetate **TSL-1**

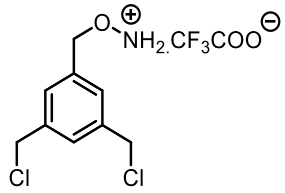

To a solution of **S2** (450 mg, 1.4 mmol) in DCM (10 mL), TFA (0.5 mL, 5 eq.) was added and the mixture was stirred for 1 h. The volatiles were removed on a rotary evaporator. Residual TFA was azeotropically removed by repeatedly dissolving the resulting oil in toluene and evaporation on a rotary evaporator to produce the title compound **TSL-1** as white viscous liquid (416 mg, 89%). To obtain product of higher purity 300 mg of crude **TSL-1** was purified by semi preparative RP-HPLC and lyophilized to yield **TSL-1** as light-yellow powder (212 mg, 71%). ^{1}H NMR (400 MHz, CD_3OD) δ = 7.56 (s, 1 H), 7.49 (d, 2 H, *J* = 1.6 Hz), 5.06 (s, 2 H), 4.70 (s, 4 H). ^{13}C NMR (100 MHz, CD_3OD) δ = 141.5, 136.7, 131.7, 131.1, 78.3, 46.49. HRMS (ESI) calculated for $C_9H_{12}Cl_2NO$ $[M+H]^+$ m/z=220.0290, found 220.0289.

Dimethyl 5-((6-bromohexyl)oxy)isophthalate **S4**

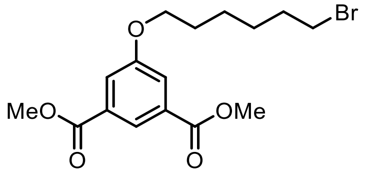

To a solution of 5-hydroxydimethylisophthalate (Cat#) (4.2 g, 20 mmol) and 1,6-dibromohexane (Cat#) (9.2 mL, 3 eq.) in CH_3CN (50 mL), potassium carbonate (8.3 g, 3 eq.) was added and the mixture was refluxed for 12 h. The reaction mixture was cooled to room temperature, diluted with water (50 mL) and extracted with ethyl acetate (3×20 mL). The combined organic layer was washed with water (50 mL) and brine (50 mL). Ethyl acetate was removed by rotary evaporator. Chromatography of the residue on silica gel using ethyl acetate-hexanes (7:1) as eluent produced the title compound **S4** as white solid (5.3 g, 70%): ^{1}H NMR (500 MHz, $CDCl_3$) δ = 8.23 (s, 1 H), 7.71 (s, 2 H), 4.02 (t, 2 H, *J* = 6.5 Hz), 3.92 (s, 6 H), 3.40 (t, 2 H, *J* = 6.5 Hz), 1.89-1.80 (m, 4 H), 1.51-1.48 (m, 4 H). ^{13}C NMR (125 MHz, $CDCl_3$) δ = 166.43, 159.40, 132.00, 123.09, 120.07, 68.60, 33.95, 32.91, 29.18, 28.13, 25.48. HRMS (ESI) calculated for $C_{16}H_{21}BrO_5Na$ $[M+Na]^+$ m/z=395.0465, found 395.0472.

Dimethyl 5-((6-(((tert-butoxycarbonyl)amino)oxy)hexyl)oxy)isophthalate **S5**

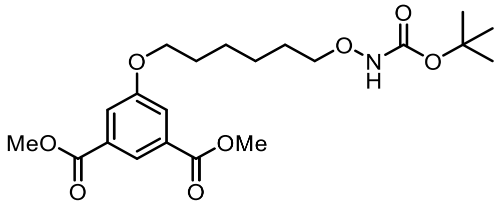

To a mixture of **S4** (5.3 g, 14 mmol) and N-Boc hydroxylamine (1.8 g, 1.2 eq.) in DCM (30 mL), DBU (1.7 mL, 1.2 eq.) was added drop wise and the solution stirred for 5 h. DCM was evaporated on a rotary evaporator and the crude residue was subjected to chromatography over silica gel with ethyl acetate-hexanes (4:1) produced the title compound **S5** as colorless oil (2.6 g, 43%). ^{1}H NMR (500 MHz, $CDCl_3$) δ = 8.24 (s, 1 H), 7.71 (s, 2 H), 7.15 (s, 1 H), 4.02 (t, 2 H, *J* = 6.5 Hz), 3.92 (s, 6 H), 3.85 (t, 2 H, *J* = 6.5 Hz), 1.80-1.64 (m, 4 H), 1.50-1.44 (m, 17 H). ^{13}C NMR (125 MHz, $CDCl_3$) δ = 166.50, 159.49, 157.22, 132.07, 123.11, 120.13, 81.86, 68.75, 29.27, 28.53, 28.49, 28.26, 26.13, 25.97. HRMS (ESI) calculated for $C_{21}H_{31}NO_8Na$ $[M+Na]^+$ m/z=448.1942, found 448.1940.

tert-Butyl ((6-(3,5-bis(hydroxymethyl)phenoxy)hexyl)oxy)carbamate **S6**

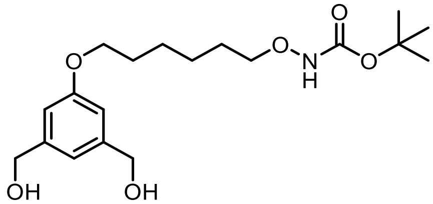

A solution of lithium aluminum hydride (713 mg, 18.3 mmol) in THF (10 mL) was added to an ice cold solution of **S7** (2.6 g, 6.1 mmol) in THF (25 mL) drop wise via cannula and the mixture was stirred for 3 h. Water was added very carefully until the evolution of hydrogen ceased. The white precipitate was filtered off and the solution was partitioned between ethyl acetate (3×30 mL) and water. The combined organic layers were washed with water, brine and dried over anhydrous sodium sulfate. Ethyl acetate was evaporated on a rotary evaporator and chromatography over silica gel of the crude residue with ethyl acetate-hexanes (1:1) produced the title compound **S6** as a colorless gum (1.5 g, 66 %): ^{1}H NMR (500 MHz, $CDCl_3$) δ = 7.15 (s, 1 H), 6.92 (s, 1 H), 6.84 (s, 1 H), 4.65 (s, 4 H), 3.98 (t, 2 H, *J* = 6.5 Hz), 3.86 (t, 2 H, *J* = 6.5 Hz), 2.03 (bs, 2 H), 1.82-1.76 (m, 2 H), 1.69-1.64 (m, 2 H), 1.52-1.43 (m, 13 H). ^{13}C NMR (125 MHz, $CDCl_3$) δ = 159.61, 156.96, 142.81, 117.36, 112.17, 81.63, 76.70, 67.87, 65.13, 29.05, 28.23, 27.92, 25.83, 25.61. HRMS (ESI) calculated for $C_{19}H_{31}NO_6Na$ $[M+Na]^+$ m/z=392.2044, found 392.2046.

tert-Butyl ((6-(3,5-bis(chloromethyl)phenoxy)hexyl)oxy)carbamate **S7**

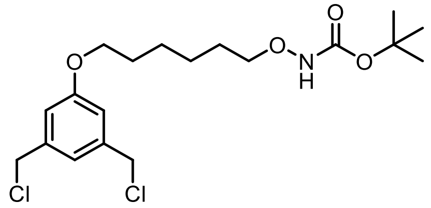

To an ice cold solution of **S6** (1.5 g, 4 mmol) and triethylamine (1.7 mL, 3 eq.) in DCM (20 mL), methane sulfonyl chloride (0.8 mL, 2.5 eq.) was added dropwise and the solution was stirred for 30 min. Without further purification THF (10 mL) and lithium chloride (500 mg, 3 eq.) was added subsequently. The ice bath was removed, and the reaction stirred for 12 h. The solvent was removed in a rotary evaporator and the crude residue was purified by chromatography over silica gel using ethyl acetate-hexanes (7:1) as eluent to produce the title compound **S7** was as colorless oil (1.2 g, 74%). ^{1}H NMR (500 MHz, $CDCl_3$) δ = 7.15 (s, 1 H), 6.97 (s, 1 H), 6.87 (s, 2 H), 4.53 (s, 4 H), 3.96 (t, 2 H, *J* = 6.5 Hz), 3.86 (t, 2 H, *J* = 6.5 Hz), 3.92 (s, 6 H), 3.40 (t, 2 H, *J* = 7.0 Hz), 1.89-1.63 (m, 4 H), 1.51-1.48 (m, 4 H). ^{13}C NMR (125 MHz, $CDCl_3$) δ = 159.61, 156.91, 139.29, 120.69, 114.72, 81.60, 68.04, 52.57, 45.86, 29.02, 28.25, 27.97, 25.88, 25.69. HRMS (ESI) calculated for $C_{19}H_{29}Cl_2NO_4Na$ $[M+Na]^+$ m/z=428.1366, found 428.1372.

O-(6-(3,5-bis(chloromethyl)phenoxy)hexyl)hydroxylammonium 2,2,2-trifluoroacetate **TSL-6**

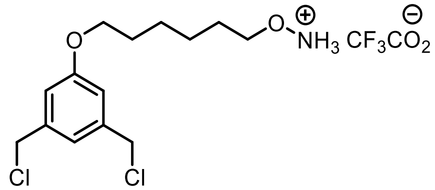

TFA (1.1 mL, 5 eq.) was added to a solution of **S7** (1.2 g, 2.9 mmol) in DCM (15 mL) and stirred for 1 h. TFA and DCM was removed on a rotary evaporator. Residual TFA was azeotropically removed by repeatedly dissolving the resulting oil in toluene and evaporation on the rotary evaporator to produce the title compound **TSL-6** as white viscous liquid (1.1 g, 89%). To yield a product of higher purity 100 mg of this compound was purified by RP-HPLC and lyophilized to produce the title compound **TSL-6** as white powder (62 mg, 62%). ^{1}H NMR (500 MHz, CD_3OD) δ = 7.05 (s, 1 H), 6.95 (s, 2 H), 4.61 (s, 4 H), 4.08-4.01 (m, 4 H), 1.82-1.68 (m, 4 H), 1.57-1.46 (m, 4 H). ^{13}C NMR (125 MHz, CD_3OD) δ = 161.81, 141.94, 122.82, 116.44, 77.12, 69.81, 47.29, 30.94, 29.48, 27.56, 27.23. HRMS (ESI) calculated for $C_{14}H_{22}Cl_2NO_2$ $[M+H]^+$ m/z=306.1028, found 306.1026.

Dimethyl 5-(3-bromopropoxy)isophthalate **S8**

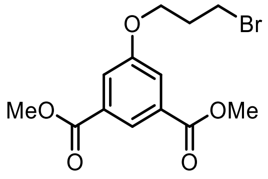

To a solution of 5-hydroxydimethylisophthalate (900 mg, 2.3 mmol) and 1,3-dibromopropane (0.31 mL, 1.5 eq.) in CH_3CN (20 mL), potassium carbonate was added and the mixture was refluxed for 12 h. The reaction mixture was allowed to cool down to room temperature, diluted with water (60 mL) and extracted with ethyl acetate (3 × 20 mL). The combined organic layer was washed with brine (50 mL). Ethyl acetate was removed on a rotary evaporator. Chromatography of the residue on silica gel in ethyl acetate-hexanes (7:1) produced the title compound **S8** as white solid (980 mg, 85%): ^{1}H NMR (500 MHz, $CDCl_3$) δ = 8.27 (s, 1 H), 7.74 (s, 2 H), 4.18 (t, 2 H, *J* = 6.0 Hz), 3.92 (s, 6 H), 3.60 (t, 2 H, *J* = 6.0 Hz), 2.33 (p, 2 H, *J* = 6.0 Hz). ^{13}C NMR (125 MHz, $CDCl_3$) δ = 166.43, 159.13, 132.20, 123.56, 120.17, 66.23, 52.78, 32.48, 29.96. HRMS (ESI) calculated for $C_{13}H_{15}BrO_5Na$ $[M+Na]^+$ m/z=353.9995, found 353.0002.

Dimethyl 5-(3-(((tert-butoxycarbonyl)amino)oxy)propoxy)isophthalate **S9**

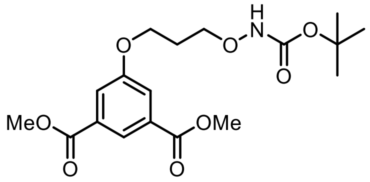

To a mixture of **S8** (3 g, 9 mmol) and N-Boc hydroxylamine (1.4 g, 1.2 eq.) in DCM (20 mL), DBU (1.6 mL, 1.2 eq.) was added drop wise and the solution stirred for 5 h. DCM was evaporated on a rotary evaporator and the crude was subjected to chromatography over silica gel with ethyl acetate-hexanes (4:1) produced the title compound **S9** as colorless oil (1.25 g, 36%). ^{1}H NMR (500 MHz, $CDCl_3$) δ = 8.26 (s, 1 H), 7.74 (s, 2 H), 7.21 (s, 1 H) 4.18 (t, 2 H, *J* = 6.0 Hz), 4.04 (t, 2 H, *J* = 6.0 Hz), 3.93 (s, 6 H), 2.14 (p, 2 H, *J* = 6.0 Hz), 1.47 (s, 9 H). ^{13}C NMR (125 MHz, $CDCl_3$) δ = 166.19, 159.03, 157.04, 131.81, 123.06, 119.91, 81.87, 73.14, 65.29, 52.44, 28.25, 28.05. HRMS (ESI) calculated for $C_{18}H_{25}NO_8Na$ $[M+Na]^+$ m/z=406.1472, found 406.1468.

tert-Butyl (3-(3,5-bis(hydroxymethyl)phenoxy)propoxy)carbamate **S10**

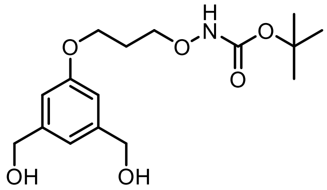

A solution lithium aluminium hydride (129 mg, 3 eq.) in THF (2 mL) was added to an ice cold solution of **S9** (1.25 g, 3.2 mmol) in THF (10 mL) via cannula drop wise and the mixture was stirred for 3 h. Water was added carefully until the evolution of hydrogen ceased. The white precipitate was filtered off and the filtrate was extracted with ethyl acetate (3 × 30 mL). The combined organic layer was washed with water, brine and dried over anhydrous sodium sulfate. Ethyl acetate was evaporated on a rotary evaporator and purification of the crude residue by chromatography over silica gel with ethyl acetate-hexanes (1:1) as eluent produced the title compound **S10** as a colorless gum (1.23 g, 86 %). ^{1}H NMR (500 MHz, $CDCl_3$) δ = 7.39 (s, 1 H), 6.87 (s, 1 H), 4.59 (s, 4 H), 4.18 (t, 2 H, *J* = 6.0 Hz), 4.04 (t, 2 H, *J* = 6.0 Hz), 3.93 (s, 6 H), 2.14 (t, 2 H, *J* = 6.5 Hz), 1.47 (s, 9 H). ^{13}C NMR (125 MHz, $CDCl_3$) δ = 159.63, 157.36, 143.14, 117.89, 112.48, 82.10, 73.61, 65.28, 65.01, 28.54, 28.42. HRMS (ESI) calculated for $C_{16}H_{25}NO_6Na$ $[M+Na]^+$ m/z=350.1574, found 350.1569.

tert-Butyl (3-(3,5-bis(chloromethyl)phenoxy)propoxy)carbamate **S11**

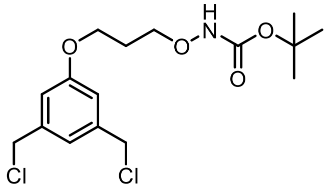

To an ice-cold solution of **S10** (1.2 g, 3.7 mmol) and trimethylamine (1.5 mL, 3 eq.) in DCM (15 mL), methane sulfonyl chloride (0.7 mL, 2.5 eq.) was added dropwise and the solution was stirred for 30 minutes. Without further purification THF (5 mL) and lithium chloride (421 mg, 3 eq.) was added subsequently. The ice bath was removed and the reaction mixture was stirred for 12 h. The volatiles were evaporated on a rotary evaporator and the crude residue was subjected to chromatography over silica gel using ethyl acetate-hexanes (1:6) as eluent produced the title compound **S11** as colorless oil (900 mg, 65%). ^{1}H NMR (500 MHz, $CDCl_3$) δ = 6.99 (s, 1 H), 6.90 (s, 2 H), 4.54 (s, 4 H), 4.31 (t, 2 H, *J* = 6.0 Hz), 4.13 (t, 2 H, *J* = 6.0 Hz), 2.15 (p, 2 H, *J* = 6.0 Hz), 1.48 (s, 9 H). ^{13}C NMR (125 MHz, $CDCl_3$) δ = 159.49, 151.69, 139.51, 121.14, 114.92, 86.19, 76.23, 45.97, 38.76, 28.27. HRMS (ESI) calculated for $C_{16}H_{23}Cl_2NO_4Na$ $[M+Na]^+$ m/z=386.0896, found 386.0902.

O-(3-(3,5-bis(chloromethyl)phenoxy)propyl)hydroxylammonium 2,2,2-trifluoroacetate
TSL-3

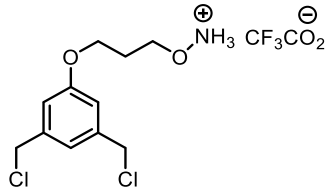

TFA (0.9 mL, 5 eq.) was added to a solution of **S11** (900 mg, 2.4 mmol) in DCM (10 mL) and stirred for 1 h. TFA and DCM was removed on a rotary evaporator. Residual TFA was azeotropically removed by repeatedly dissolving the resulting oil in toluene and evaporation, which produced the title compound **TSL-3** as white gummy liquid (725 mg, 80%). To obtain a product of higher purity, 300 mg of the title compound was purified in RP-HPLC and lyophilized to get a white powder (202 mg, 67%). ^{1}H NMR (400 MHz, CD_3OD) δ = 7.08 (s, 1 H), 6.98 (s, 2 H), 4.63 (s, 4 H), 4.27 (t, 2 H, *J* = 6.0 Hz), 4.14 (t, 2 H, *J* = 6.0 Hz), 2.20 (p, 2 H, *J* = 6.0 Hz).^{13}C NMR (100 MHz, CD_3OD) δ = 161.3, 142.1, 123.3, 116.5, 73.9, 65.9, 29.7. HRMS (ESI) calculated for $C_{11}H_{16}C_{l2}NO_2$ $[M+Na]^+$ m/z=264.0553, found 264.0551.

1.3. *General procedure for peptide synthesis*

Peptides were synthesized on an automated peptide synthesizer (Prelude®X; GYROS PROTEIN Technology) using standard solid phase amide coupling. After synthesis the resin was transferred to a Poly-Prep column (BIORAD) and washed with DCM (10 mL) and dried in vacuum. The resin was then treated with a cleavage cocktail (7 mL) containing TFA/H_2O/TIPS/EDT, 90/2.5/5/2.5 (v/v/v/v) for the global deprotection and cleavage of the peptide from the resin. After 4 h the flow through from the column was collected and the resin was rinsed with TFA (1 mL). The combined cleavage mixture reduced in volume to 2 mL by means of gently bubbling nitrogen through it and was added drop-wise to cold diethyl ether (10 mL) in a 15 mL polypropylene centrifuge tube (Falcon, Thermo Fisher). The precipitate formed was separated by centrifugation (5 min, 3000 rpm). Supernatant was decanted and the precipitates were washed with cold diethyl ether (10 mL). The centrifugation and washing steps were repeated for two more cycles. The precipitates were air-dried. For HPLC purification, crude peptide powder was dissolved in MeCN and water; addition of acetic acid was necessary in some cases to dissolve the peptide. The solution was injected into a semi preparative RP-HPLC system. The fractions corresponding to the main peak were collected. CH_3CN was removed in Speed Vac (Savant SPD111V). The aqueous solution was lyophilized to yield the peptide as white powder.

1.4. *Protocol 1: Bicyclization of Peptides SX_mCX_nC with **TSL** using C18 spin column.*
Materials:

- Solution of 5 mM peptide (SX_mCX_nC) in water
- 30 mM stock of **TSL** in water : acetonitrile (1:1)
- 30 mM stock of $NaIO_4$ solution in water
- 125 µM stock solution of TCEP in water
- C18-desalting spin column (#89870 Pierce C-18 spin column from Thermo Scientific)
- MiliQ (mQ) water and HPLC grade acetonitrile
- 500 mM Tris, pH 8.5
- 5% TFA solution in mQ water.
- PBS (50 mM K_2HPO_4, 150 mM NaCl, pH 7.4)
- 0.6 mL Eppendorf tubes, pipettes and tips
- LCMS instrument and auto-sampler vials for LCMS

Procedure (analytical scale: 25 nanomole or 25 µg of 1000 Da peptide):	Vol. (µL):
1. In a 0.6 mL Eppendorf tube, combine peptide (5 µL from 5 mM stock) and 45 µL PBS to a final concentration of peptide 0.5 mM.	45+5=50
2. Take 1 µL out to check the purity of the starting material and serve as reference (mix 1 µL with 9 µL 0.1% TFA and inject 5 µL in the LCMS)	50-1=49
3. Add sodium periodate (1.2 eq., 0.6 mM, 1 µL from 30 mM stock) and incubate for 5 min in the dark.	
4. Load the resulting solution onto an equilibrated C18 desalting spin column. Wash the column with 2 × 50 µL of 20% acetonitrile containing 0.1% TFA and elute the peptide with 2 × 20 µL of 70% acetonitrile. A typical volume collected at this step is 40 µL	49+1=50<br>40
5. Remove excess of acetonitrile in the speed-vac. A typical volume after this step is 12 µL. Then add 28 µL mQ water.	12+28=40
6. To solution from 5, add (in this order!): 8 µL acetonitrile, then 1 µL of 5% TFA (final TFA concentration = 0.1%) and then 1 µL of 30 mM stock solution of **TSL** (1.2 eq., final concentration 0.6 mM). Incubate for 1 h.	40+1+8+1=50
7. If necessary, monitor the progress of the reaction by withdrawing 1 µL and quenching is with 9 µL of 0.1% TFA and injecting 5 µL in the LCMS.	50-1=49
8. To the resulting oxime, add TCEP (5 eq., 1 µL from 125 mM stock solution, final concentration 2.5 mM) and incubate for 30 minutes.	
9. Add 30 µL mQ water followed by addition of 20 µL 500 mM Tris of pH 8.5 (final Tris concentration 100 mM) and incubate for an hour.	49+1=50
10. To confirm the formation of the product, withdraw 1 µL of reaction mixture, quenching with 9 µL 0.1% TFA and injecting 5 µL in the LCMS	50+30+20=100
	100-1=99

1.5. *Protocol 2: Bicyclization of Peptides SX_mCX_nC with* ***TSL*** *using methionine as quencher*

Materials:

- Solution of 25 mM peptide (SX_mCX_nC) in water
- 25 mM stock of $NaIO_4$ solution in water
- 125 mM methionine in water
- 30 mM stock of **TSL** in water : acetonitrile (1:1)
- 125 µM stock solution of TCEP in water
- MiliQ (mQ) water and HPLC grade acetonitrile
- 1000 mM TRIS of pH 8.5
- 5% TFA solution in mQ water.
- 1X PBS, (50 mM phosphates, 150 mM NaCl, pH 7.4)
- 0.6 mL epi tubes, pipettes and tips
- LCMS instrument and auto-sampler vials for LCMS

Procedure (analytical scale: 25 nanomole or 25 µg of 1000 Da peptide):	Vol. (µL):
1. In a 0.6 mL epi, combine peptide (1 µL from 25 mM stock) 39 µL PBS pH 7.4 and 10 µL acetonitrile to a final concentration of 0.5 mM.	1+39+10=50
2. Take 1 µL out to check the purity of the starting material and serve as reference point (mix 1 µL with 9 µL 0.1% TFA and injecting 5 µL in the LCMS)	50-1=49
3. Add sodium periodate (1.0 eq., 0.5 mM, 1 µL from 25 mM stock) and incubate for 5 min in the dark.	49+1=50
4. Take 1 µL out to check LCMS	50-1 = 49
5. To the resulting solution add methionine (5.0 eq., 2.5 mM, from 125 mM stock) and incubate for 15 min	49+1 = 50
6. To the solution then add 1 µL of 5% TFA (final TFA concentration = 0.1%) and 1 µL of 30 mM stock solution of **TSL** (1.2 eq., final concentration 0.6 mM). Incubate for 1 h.	50+2 = 52
7. Monitor the progress of the reaction by withdrawing 2 µL in 18 µL of 0.1% TFA and injecting 5 µL in the LCMS.	52-2 = 50
8. To the resulting oxime, add TCEP (5 eq., 1 µL from 125 mM stock solution, final concentration 2.5 mM) and incubate for 30 minutes.	50+1=51
9. Add 10 µL acetonitrile, 29 µL mQ water, followed by addition of 10 µL 1000 mM Tris of pH 8.5 (final Tris concentration 100 mM) and incubate for an hour.	51+10+29+10 =100
10. To confirm the formation of the product, withdraw 1 µL of reaction mixture, quenching with 9 µL 0.1% TFA and injecting 5 µL in the LCMS.	100-1=99

1.6. *General procedure for one-pot bicyclization on semi-preparative scale*
In a 50 mL poly-propylene falcon tube, 10 mg of peptide (NH_2-SYCKPFC-$CONH_2$, M.W = 846 Da, 12 µmol) was dissolved in 20.8 mL PBS (pH 7.4) containing 2.36 mL of acetonitrile. To the resulting solution, sodium periodate in water (1.2 eq., 236 µL from 500 mM stock) was added and mixed on a rocker for 5 minutes in the dark. A solution of methionine in water (5 eq., 9 mg, 0.06 mmol) was added to quench the residual oxidizing agent (periodate/iodate). After 15 minutes, neat TFA was added to the reaction (23.6 µL to a final concentration of 0.1%) followed by the addition of **TSL-1** in acetonitrile (2 eq., 26.6 µL from 1 M stock). As oxo-aldehyde and formaldehyde are generated simultaneously, an excess of **TSL** was needed in this step (Scheme S2). After incubation for 1 h, solution of TCEP in water (5 eq., 15 mg, 0.06 mmol) was added and rocked for 30 minutes to reduce the disulfide bond. The reaction mixture was diluted by 16.5 mL of water and 2.36 mL of acetonitrile followed by the addition of sodium bicarbonate at pH 10 (4.7 mL from 1 M stock to a final concentration of 100 mM) and rocked for 3 h. Completion of bicyclization can be confirmed by sampling an aliquot and analyzing it by LCMS. The reaction was purified in semi preparative RP-HPLC to yield a bicyclic peptide **TSL-1**-SYCKPFC (5 µmol, 4.6 mg, 42%).

1.7. *General Protocol for bicyclization with **TBMB***
Peptide **12a** (10 mg, 5.4 µmol) was dissolved in 5.4 mL bicarbonate buffer (100 mM, pH 10) containing 10% acetonitrile. A solution of TCEP (2.5 eq, 27 µL of 500 mM stock, to a final concentration 2.5 mM) was added, follow with a solution of TBMB was added (1 eq, 11 µL of 500 mM in acetonitrile) and the reaction mixture was mixed on a rocker for 20 h. Upon consumption of all the starting material (as confirmed by LCMS) the reaction mixture was directly purified on RP-HPLC and freeze-dried to yield **12f** as light yellow powder (4.3 mg, 41%).

1.8. *General Protocol for cyclization with perflurodiphenylsufide (**PFS**)*
Peptide **5a** (10 mg, 10 µmol) was dissolved in 5.0 mL DMF in a glass vial and a solution of perflurodiphenylsulfide (4 eq, 14 mg, 40 µmol) was added to this solution. 560 µL of 50 mM Tris base (final concentration of Tris is 5 mM/DMF) were added into the vial. The mixture was vortexed for 30 sec and incubated at rt for 1 h. After 1 h, the reaction was quenched by diluting 10 times with 50% aq. acetonitrile containing 0.1% TFA. The product was purified with RP-HPLC, freeze dried to obtain **5e** as white powder (5 mg, 40%). [1]

1.9. *General Protocol for cyclization with α,α'-Dibromo-m-xylene (**DBMB**)*
Peptide **5a** (10 mg, 10 µmol) was dissolved in 5.0 mL H_2O/ACN 50% in a glass vial and a solution of α,α′-Dibromo-m-xylene in acetonitrile (1.2 eq) was added to this solution. 500 µL of 500 mM Tris-HCl buffer at pH 8.5 (final concentration of Tris-HCl buffer 50 mM) were added into the vial. The mixture was vortexed for 30 sec and incubated at rt for 1 hour. After 1 hour, the reaction was purified with RP-HPLC, freeze dried to obtain **5g** as white powder (5.1 mg, 46%).

1.10. *General bicyclization analytical procedure for **10b** and **11b***:

Materials:

- Solution of 5 mM peptide (SXmCXnC) in **TSL** in water: acetonitrile (9:1)
- 30 mM stock of **TSL** in water: acetonitrile (1:2)
- 30 mM stock of $NaIO_4$ solution in water
- 125 µM stock solution of TCEP in water
- C18-desalting spin column (#89870 Pierce C-18 spin column from Thermo Scientific)
- MiliQ (mQ) water and HPLC grade acetonitrile
- 500 mM $KHCO_3$, pH 8.0
- 5% TFA solution in mQ water.
- PBS (50 mM K_2HPO_4, 150 mM NaCl, pH 7.4)
- 0.6 mL Eppendorf tubes, pipettes and tips
- LCMS instrument and auto-sampler vials for LCMS

**Procedure (analytical scale: 25 nanomole or 25 µg of 1000 Da peptide):**	**Vol. (µL):**
1. In a 0.6 mL Eppendorf tube, combine peptide (5 µL from 5 mM stock) and 45 µL PBS to a final concentration of peptide 0.5 mM.	45+5=50
2. Take 1 µL out to check the purity of the starting material and serve as reference (mix 1 µL with 9 µL 0.1% TFA and inject 5 µL in the LCMS)	50-1=49
3. Add sodium periodate (1.2 eq., 0.6 mM, 1 µL from 30 mM stock) and incubate for 5 min in the dark. Add methionine (12 eq., 6 mM, 1 µL from 300 mM stock) and incubate for an hour.	49+1=50
4. Load the resulting solution onto an equilibrated C18 desalting spin column. Wash the column with 2 × 50 µL of 20% acetonitrile containing 0.1% TFA and elute the peptide with 2 × 20 µL of 70% acetonitrile. A typical volume collected at this step is 40 µL	40
5. Remove excess of acetonitrile in the speed-vac. A typical volume after this step is 12 µL. Then add 28 µL miliQ water.	12+28=40
6. To solution from 5, add (in this order): 8 µL acetonitrile, then 1 µL of 5% TFA (final TFA concentration = 0.1%) and then 1 µL of 30 mM stock solution of TSL (1.2 eq., final concentration 0.6 mM). Incubated for 1 h.	40+1+8+1=50
7. If necessary, monitor the progress of the reaction by withdrawing 1 µL and quenching is with 9 µL of 0.1% TFA and injecting 5 µL in the LCMS.	50-1=49
8. To the resulting oxime, add TCEP (5 eq., 1 µL from 125 mM stock solution, final concentration 2.5 mM) and incubate for 30 minutes.	49+1=50
9. Add 30 µL mQ water followed by addition of 20 µL 500 mM $KHCO_3$ of pH 8.0 (final $KHCO_3$ concentration 100 mM) and incubate for an hour.	50+30+20 =100
10. To confirm the formation of the product, withdraw 1 µL of reaction mixture, quenching with 9 µL 0.1% TFA and injecting 5 µL in the LCMS	100-1=99

1.11. *Protocol for **10b** scale up synthesis:*

Peptide **10a** (10 mg, 0.0084 mmol) was dissolved in water:acetonitrile (1.67 mL, v/v 7:3) and buffered with PBS (14.98 mL, 50 mM K_2HPO_4, 150 mM NaCl, pH 7.4). 1 µL of the solution was sampled for LCMS (mixed 1 µL with 9 µL of 0.1% TFA and injected 5 µL in the LCMS). A solution of $NaIO_4$ in water (336 µL, 1.2 eq, 2.16 mg, 0.01 mmol) was added to the reaction and incubated at 20 °C in the dark for 5 min. To quench the oxidation, a solution of methionine in water (336 µL, 12 eq, 14.86 mg, 0.01 mmol) was added to the reaction and incubated for 1 h. The resulting solution was loaded onto an equilibrated C18 desalting spin column (pre-washed the column with 2 × 2.5 mL of 20% acetonitrile containing 0.1% TFA) and eluted the peptide with 2 × 500 µL of 70% acetonitrile without TFA. A typical volume collected at this step is 13.4 mL. The excess acetonitrile was removed in the speed-vac and the typical volume after this step was ~4 mL. MiliQ water was added to a final volume of 13.4 mL and1 µL of the solution was sampled to check the purity of the eluent to serve as a reference (mix 1 µL with 9 µL 0.1% TFA and inject 5 µL in the LCMS). To the eluent, we added acetonitrile in water:acetonitrile v/v 1:1 (2.67mL), 5% TFA (336 µL) and then a solution of **TSL-6** (336 µL, 1.2 eq, 4.2 mg, 0.01 mmol,) was added. The reaction mixture stirred for 2 h at 30 °C. The progresses of the reaction were monitored by withdrawing 1 µL, quenching with 9 µL of 0.1% TFA and injecting 5 µL in the LCMS. When the reaction was completed, a solution of TCEP in water (336 µL, 5 eq, 12.02 mg, 0.043 mmol) was added to the reaction and stirred for1 h (1 µL of the reaction was sampled, mixed with 9 µL of 0.1% TFA and injected 5 µL in the LCMS as a reference). The reaction mixture was then supplemented with mQ water (10.05 mL), adjusted the $KHCO_3$ buffer to a final concentration of 100 mM (6.6 mL from 500 mM $KHCO_3$ of pH 8.0 stock) and incubated for 3 h. The progress of the reaction was monitored by withdrawing 1 µL, quenching with 9 µL of 0.1% TFA and injecting 5 µL in the LCMS. Then, the reaction mixture was concentrated by lyophilization and was purified by LCMS. The yield of the bicyclization is 3.5 mg, 32% from 10 mg starting material.

1.12. *Protocol for **11b** scale up synthesis:*

Peptide **11a** (10mg, 0.0066 mmol) was dissolved in water:acetonitrile (1.32 mL, v/v 7:3) and buffered with PBS (11.88 mL, 50 mM K_2HPO_4, 150 mM NaCl, pH 7.4). 1 µL of the solution was sampled for LCMS (mixed 1 µL with 9 µL of 0.1% TFA and injected 5 µL in the LCMS). A solution of $NaIO_4$ in water (264 µL, 1.2 eq, 1.7 mg, 0.0079 mmol) was added and was incubated at 20 °C in the dark for 5 min. To quench the oxidation, a solution of methionine in water (264 µL, 12 eq, 11.7 mg, 0.079 mmol) was added and incubated for 1 h. The resulting solution was loaded onto an equilibrated C18 desalting spin column (pre-washed the column with 2 × 2.5 mL of 20% acetonitrile containing 0.1% TFA) and elute the peptide with 2 × 500 µL of 70% acetonitrile. A typical volume collected at this step is 10.56 mL. The excess acetonitrile was removed in the speed-vac and the typical volume after this step was ~ 3 mL. MiliQ water was added to a final volume of 10.56 mL. 1 µL of the solution was sampled to check the purity of the eluent and to serve as a reference (mixed 1 µL with 9 µL of 0.1% TFA and injected 5 µL in the LCMS). To the reaction mixture, we added acetonitrile (2.1 mL), 5% TFA (336 µL) and then a solution of **TSL-6** in water:acetonitrile v/v 1:1 (264 µL, 1.2 eq, 3.18 mg, 0.0079 mmol,) was added to the reaction. The reaction mixture was stirred for 2 h at 30 °C. The progresses of the reaction were monitored by withdrawing 1 µL, quenching with 9 µL of 0.1% TFA and injecting 5 µL in the LCMS. When the reaction was completed, a solution of TCEP in water (264 µL, 5 eq, 9.47 mg, 0.0339 mmol) was added to the solution, and stirred for 1 h (1 µL of the reaction was sampled, mixed with 9 µL of 0.1% TFA and injected 5 µL in the LCMS as a reference) Reaction mixture was then supplemented with miliQ water (7.92 mL), adjusted the $KHCO_3$ buffer to a final concentration of 100 mM (5.2 mL from 500 mM $KHCO_3$ of pH 8.0 stock) and incubated for 3 h. The progress of the reaction was monitored by withdrawing 1 µL, quenching with 9 µL of 0.1% TFA and injecting 5 µL in the LCMS. Then, the reaction mixture was concentrated by lyophilization and was purified by LCMS. The yield of the bicyclization was 2.9 mg, 28% from 10 mg starting material.

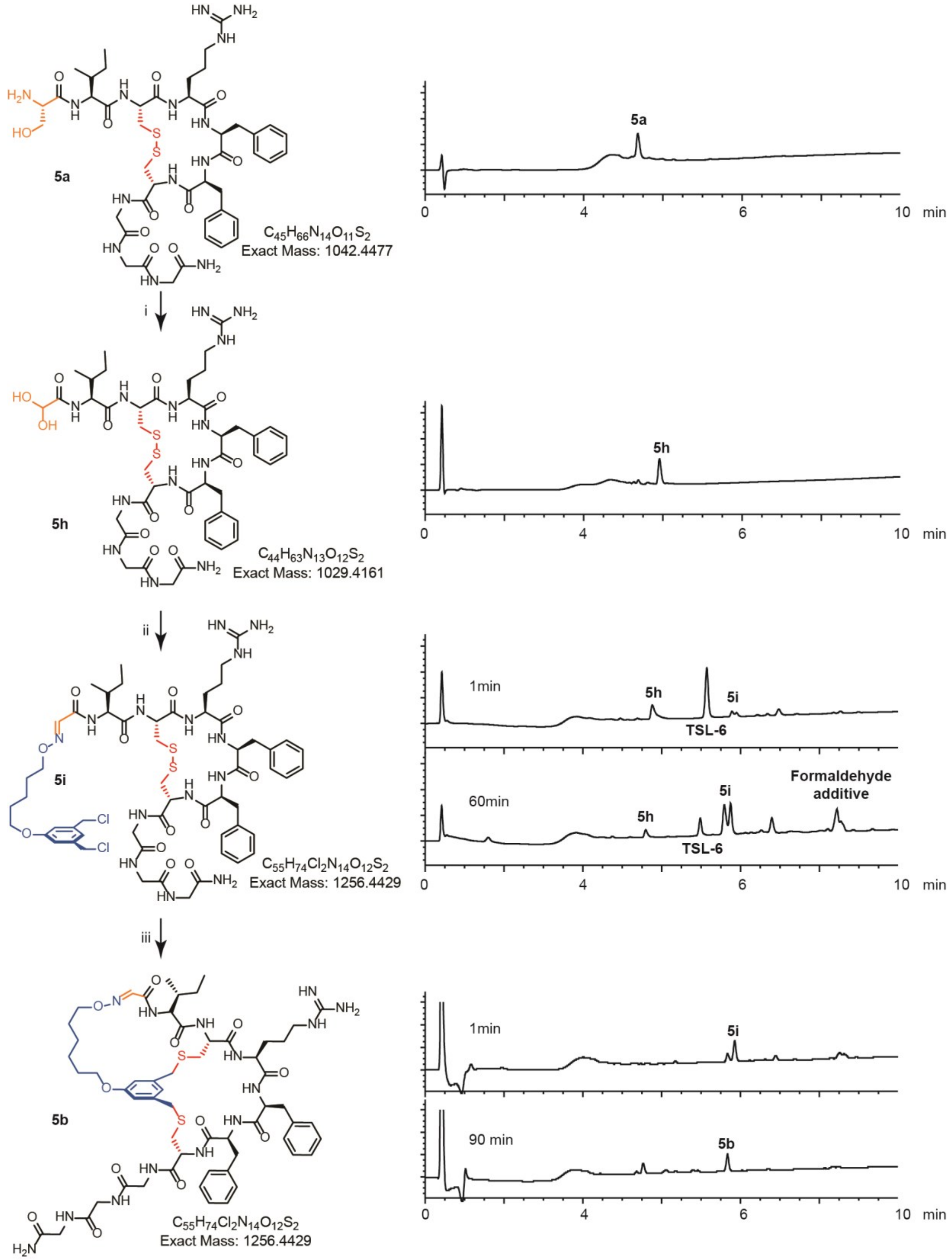

Scheme S2: One-pot bicyclization of **5a** (0.2 mmol) with **TSL-6**: Reagents and conditions: (i) 2.4 mM $NaIO_4$, PBS (pH 7.4), 5 min, 1 mM methionine, 20 min (ii) 0.1% TFA, 1 mM TSL-6, 1 h; 1 mM TCEP, 30 min; (iii) 150 mM bicarbonate buffer (pH 10), 90 mins;

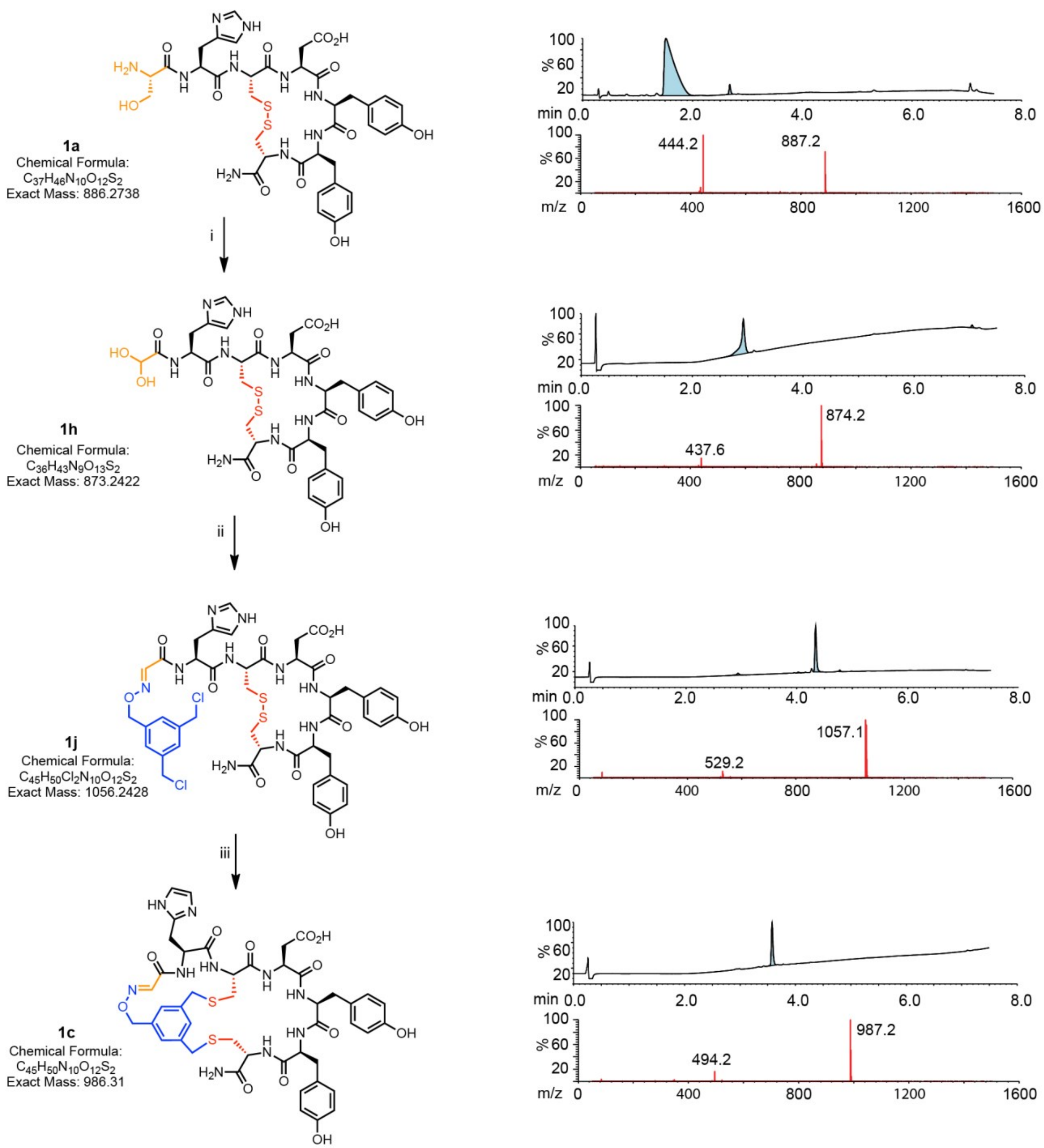

Scheme S3: One-pot bicyclization of **1a** (0.5 mM) with **TSL-1**. Reagents and conditions: (i) 0.6 mM $NaIO_4$, PBS (pH 7.4), 5 min. (ii) 0.1% TFA, 0.6 mM **TSL-1** (pH 4), 1 h; (iii) 2.5 mM TCEP, 30 min; 100 mM TRIS (pH 8.5), 1 h.

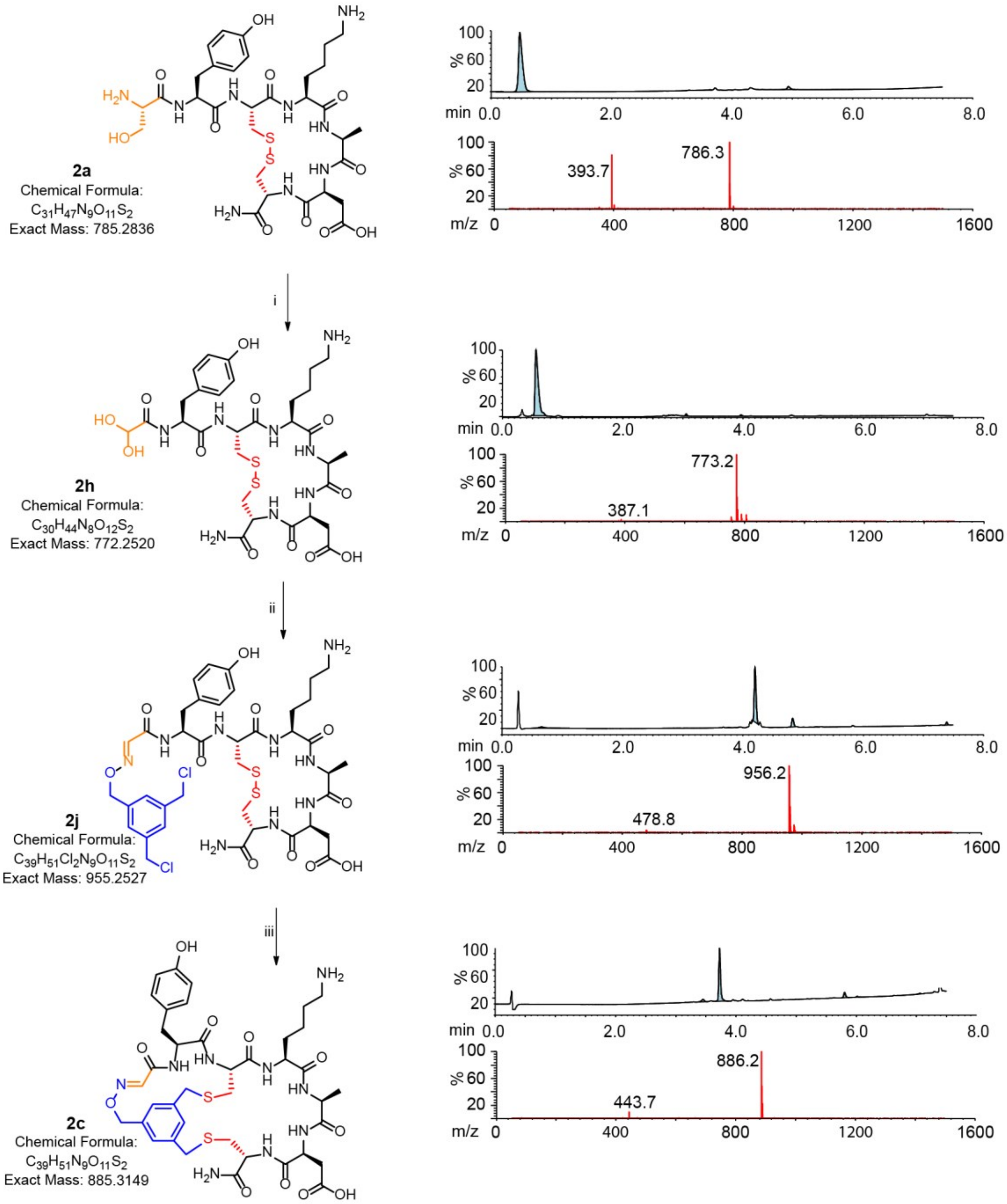

Scheme S4: One-pot bicyclization of **2a** (0.5 mM) with **TSL-1**. Reagents and conditions: (i) 0.6 mM $NaIO_4$, PBS (pH 7.4), 5 min. (ii) 0.1% TFA, 0.6 mM **TSL-1** (pH 4), 1 h; (iii) 2.5 mM TCEP, 30 min; 100 mM TRIS (pH 8.5), 1 h.

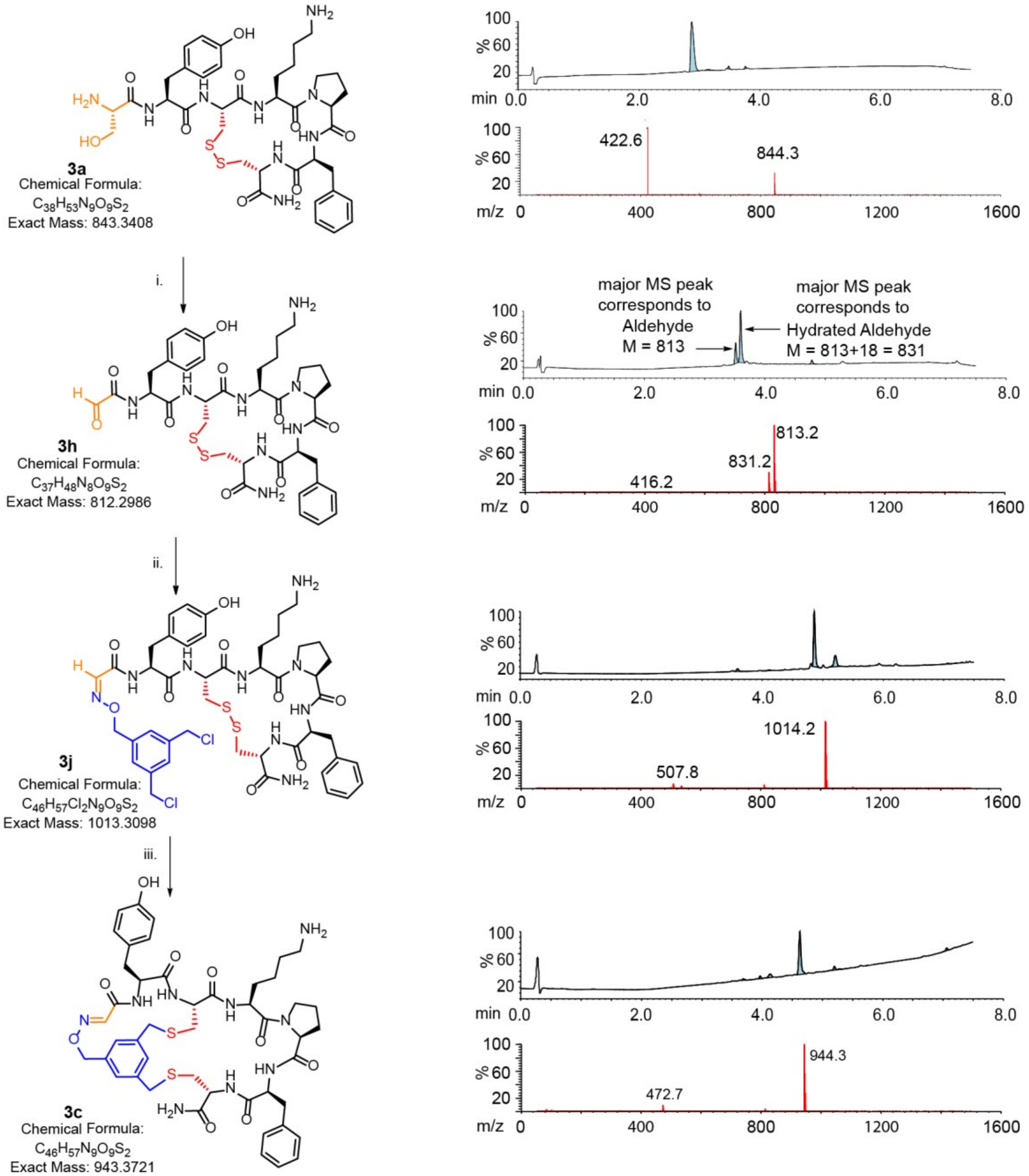

Scheme S5: One-pot bicyclization of **3a** (0.5 mM) with **TSL-1**. Reagents and conditions: (i) 0.6 mM $NaIO_4$, PBS (pH 7.4), 5 min, 1 mM methionine, 15 min (ii) 0.1% TFA, 0.6 mM **TSL-1** (pH 4), 1 h; (iii) 2.5 mM TCEP, 30 min; 100 mM TRIS (pH 8.5), 1 h.

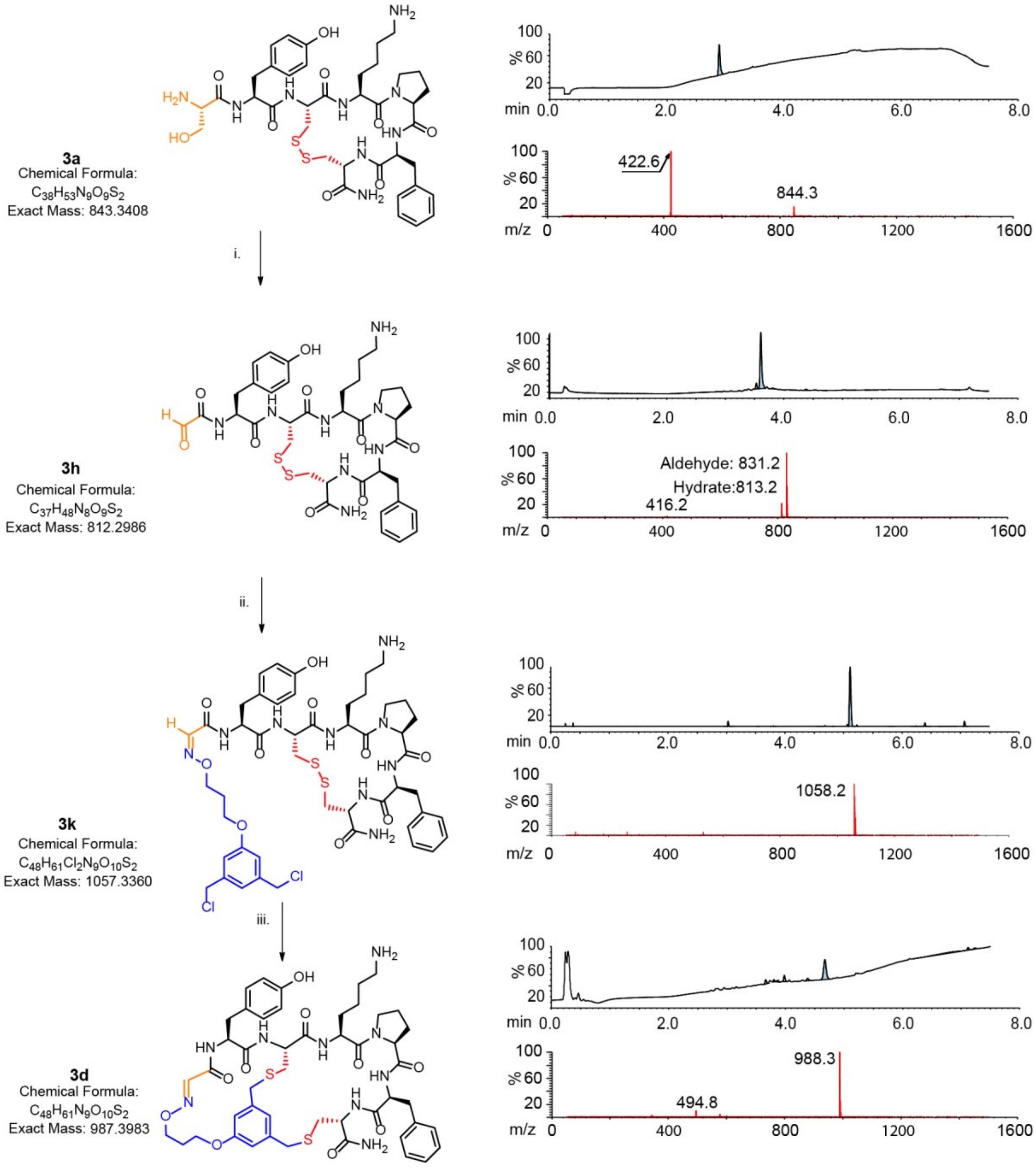

Scheme S6: One-pot bicyclization of **3a** (0.5 mM) with **TSL-3**. Reagents and conditions: (i) 0.6 mM $NaIO_4$, PBS (pH 7.4), 5 min, 1 mM methionine, 15 min (ii) 0.1% TFA, 0.6 mM **TSL-3** (pH 4), 1 h; (iii) 2.5 mM TCEP, 30 min; 100 mM TRIS (pH 8.5), 1 h.

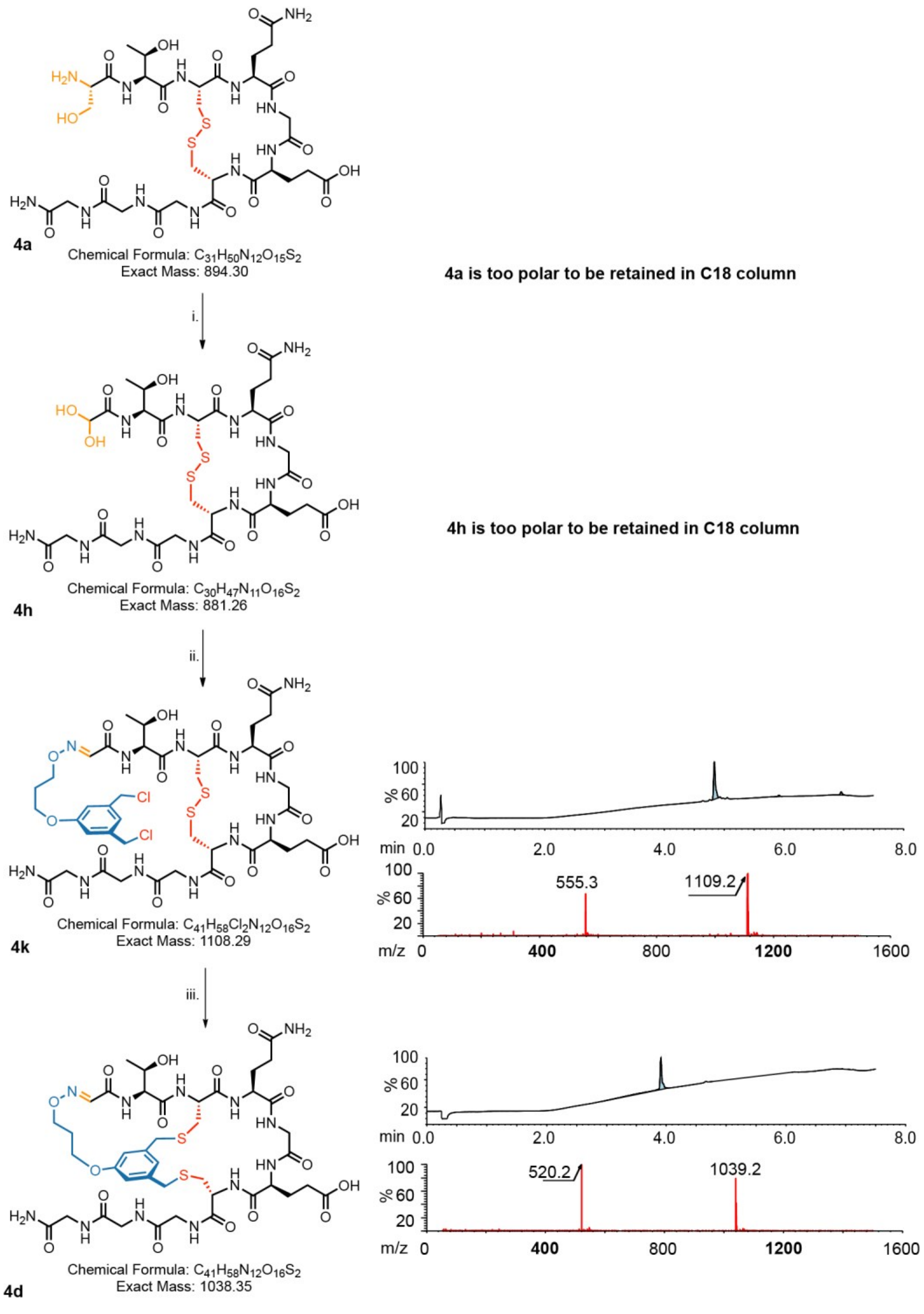

Scheme S7: One-pot bicyclization of **4a** with **TSL-3**: Reagents and conditions: (i) 0.6 mM $NaIO_4$, PBS (pH 7.4), 5 min, 1 mM methionine, 15 min (ii) 0.1% TFA, 0.6 mM **TSL-3** (pH 4), 1 h; (iii) 2.5 mM TCEP, 30 min; 100 mM bicarbonate buffer (pH 10), 30 min.

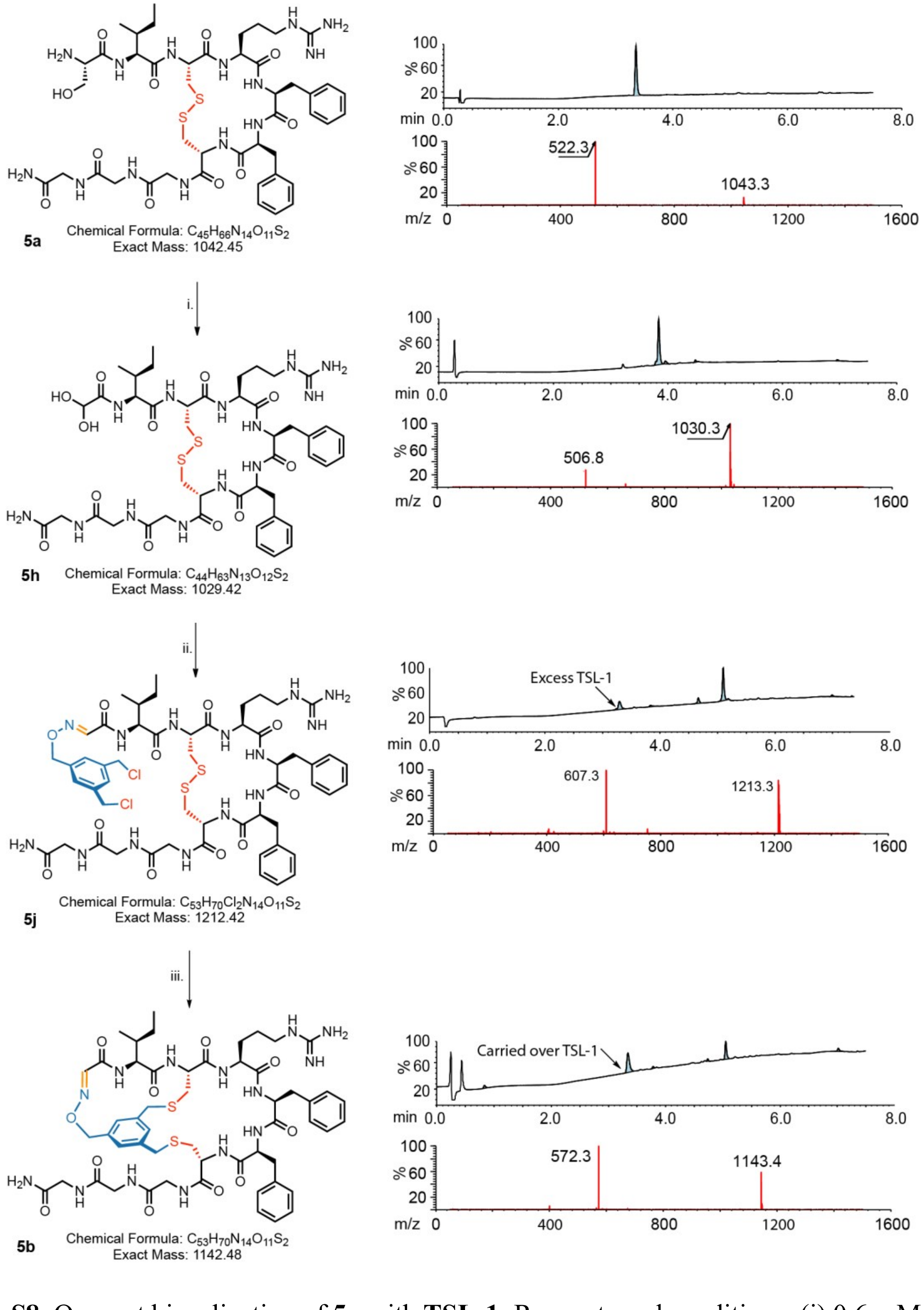

Scheme S8: One-pot bicyclization of **5a** with **TSL-1**: Reagents and conditions: (i) 0.6 mM $NaIO_4$, PBS (pH 7.4), 5 min, 1 mM methionine, 15 min (ii) 0.1% TFA, 0.6 mM **TSL-1** (pH 4), 1 h; (iii) 2.5 mM TCEP, 30 min; 100 mM bicarbonate buffer (pH 10), 30 min.

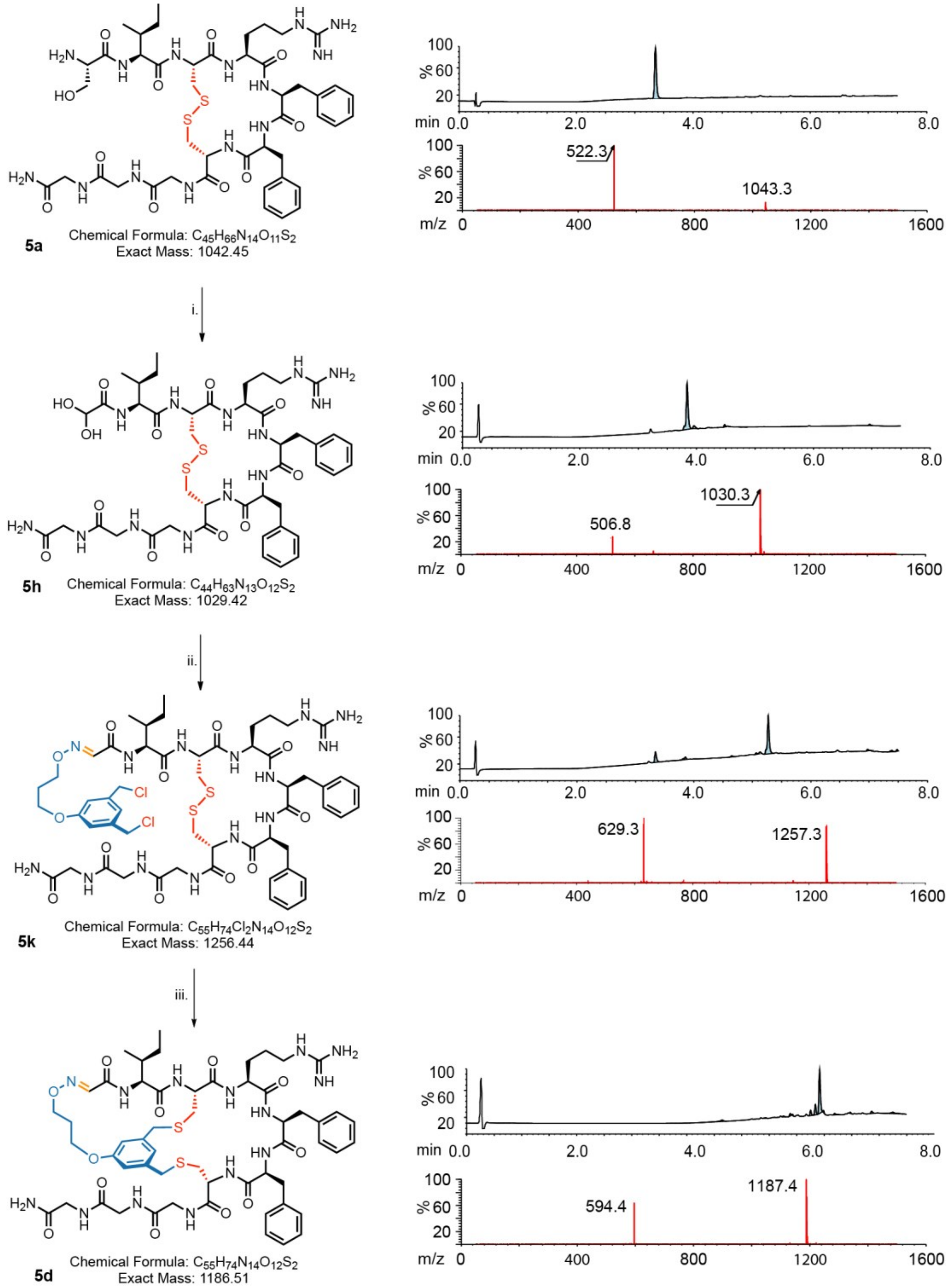

Scheme S9: One-pot bicyclization of **5a** with **TSL-3**: Reagents and conditions: (i) 0.6 mM $NaIO_4$, PBS (pH 7.4), 5 min, 1 mM methionine, 15 min (ii) 0.1% TFA, 0.6 mM **TSL-3** (pH 4), 1 h; (iii) 2.5 mM TCEP, 30 min; 100 mM bicarbonate buffer (pH 10), 30 min.

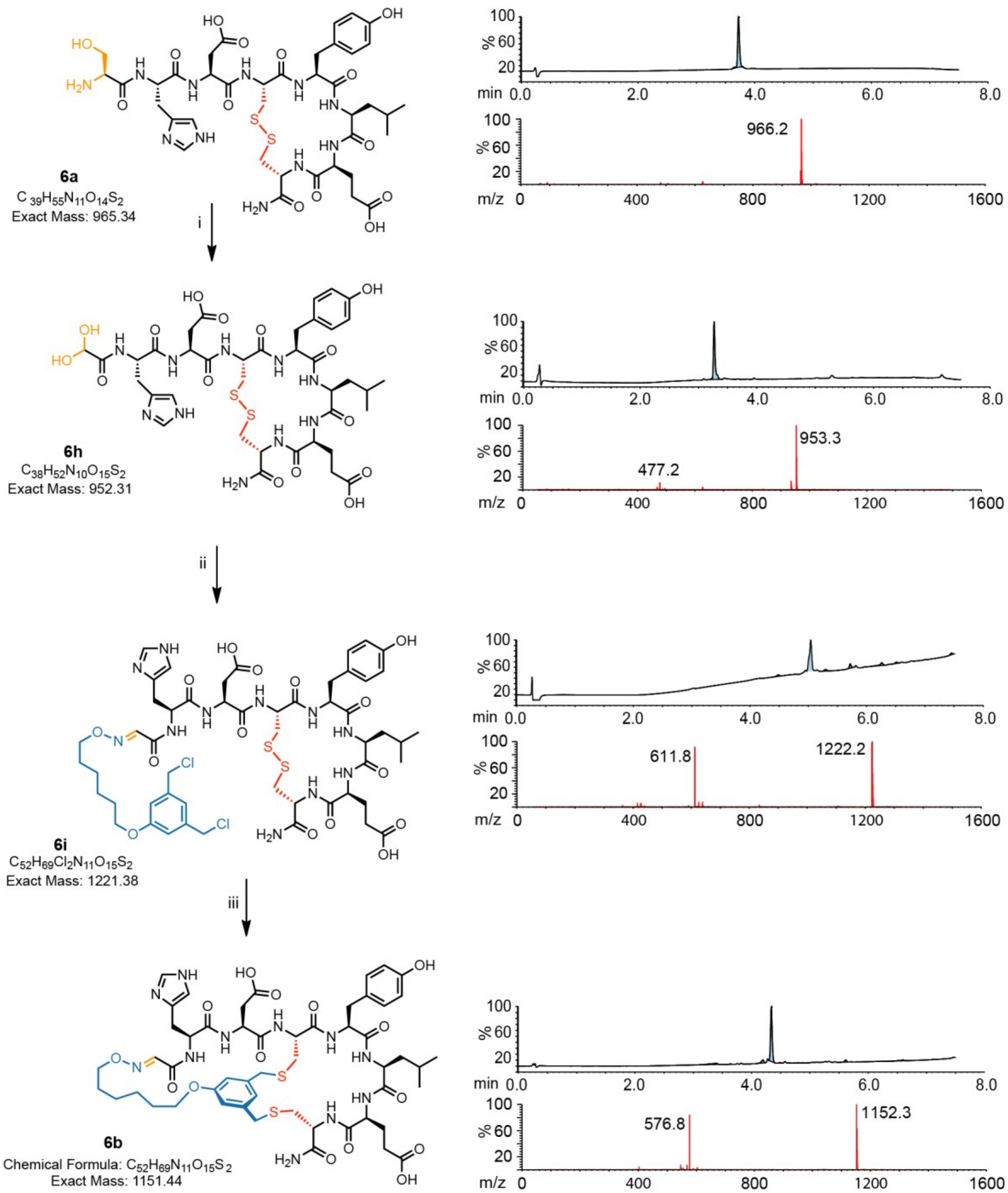

Scheme S10: One pot bicyclization of **6a** (0.5 mM) with **TSL-6**. Reagents and conditions: (i) 0.6 mM $NaIO_4$, PBS (pH 7.4), 5 min. (ii) 0.1% TFA, 0.6 mM **TSL-6** (pH 4), 1 h; (iii) 2.5 mM TCEP, 30 min; 100 mM TRIS (pH 8.5), 1 h.

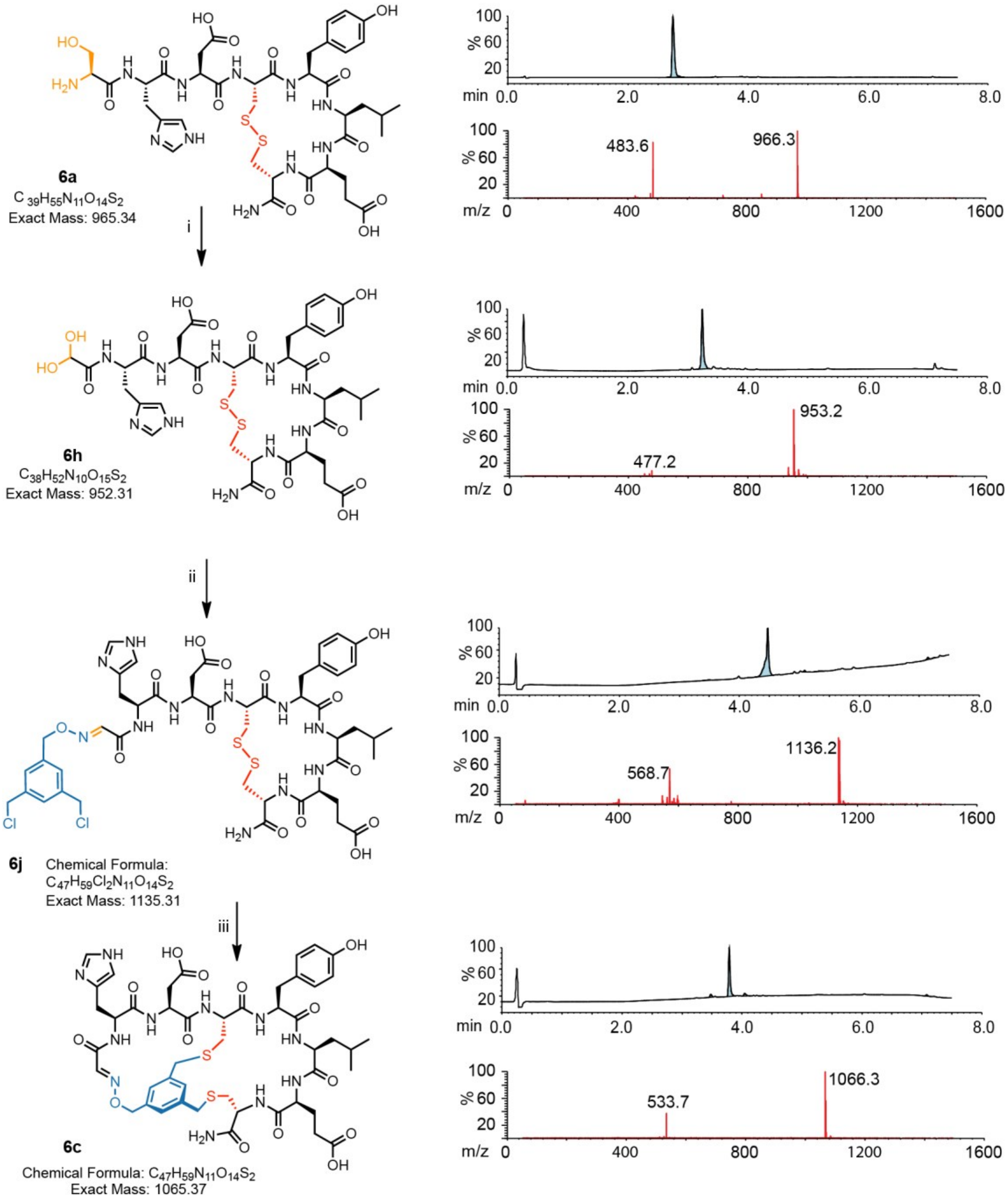

Scheme S11: One-pot bicyclization of **6a** (0.5 mM) with **TSL-1**. Reagents and conditions: (i) 0.6 mM NaIO4, PBS (pH 7.4), 5 min. (ii) 0.1% TFA, 0.6 mM **TSL-1** (pH 4), 1 h; (iii) 2.5 mM TCEP, 30 min; 100 mM TRIS (pH 8.5), 1 h.

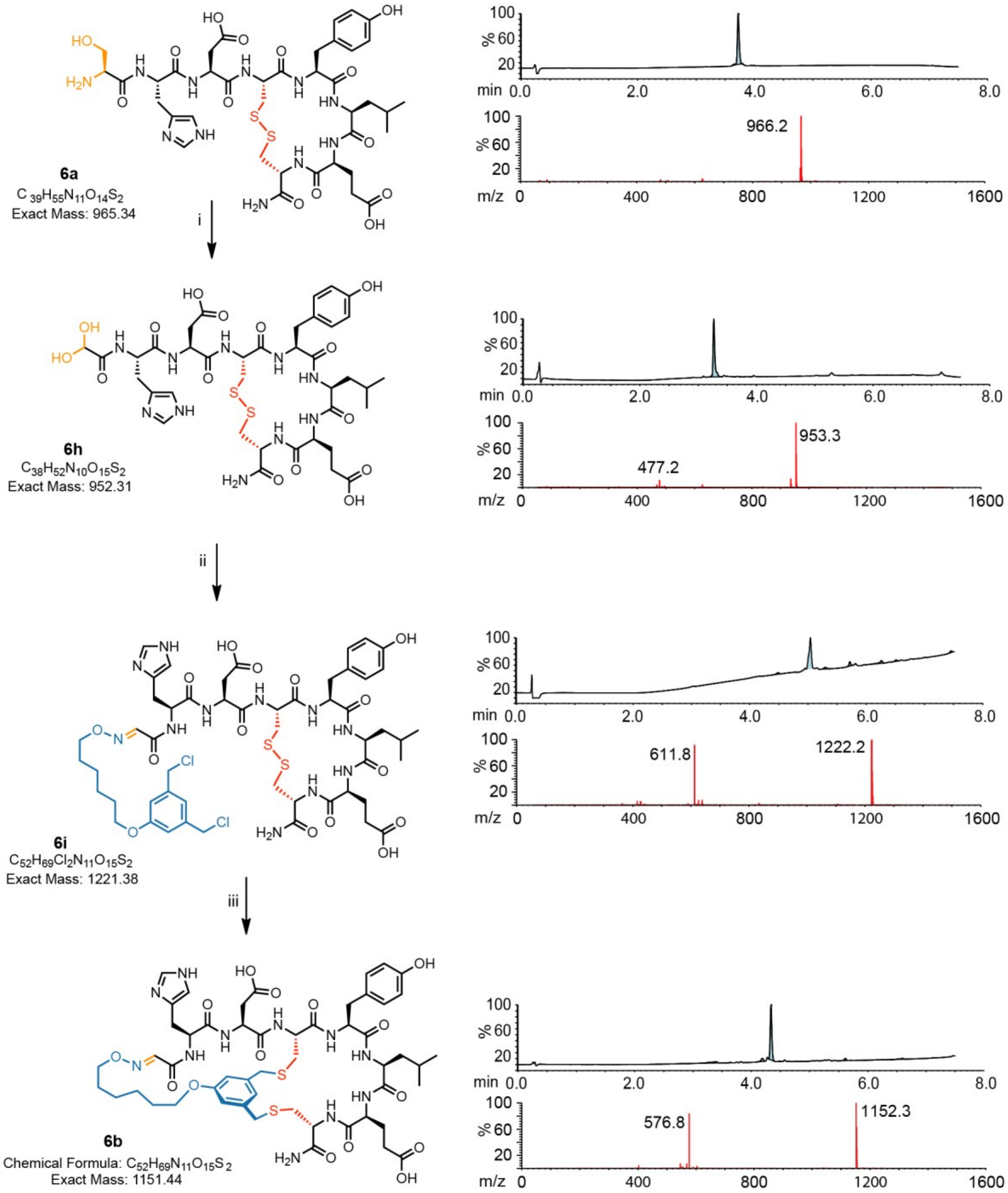

Scheme S12: One-pot bicyclization of **6a** (0.5 mM) with **TSL-6**. Reagents and conditions: (i) 0.6 mM $NaIO_4$, PBS (pH 7.4), 5 min, 1 mM methionine, 15 min (ii) 0.1% TFA, 0.6 mM **TSL-6** (pH 4), 1 h; (iii) 2.5 mM TCEP, 30 min; 100 mM TRIS (pH 8.5), 1 h.

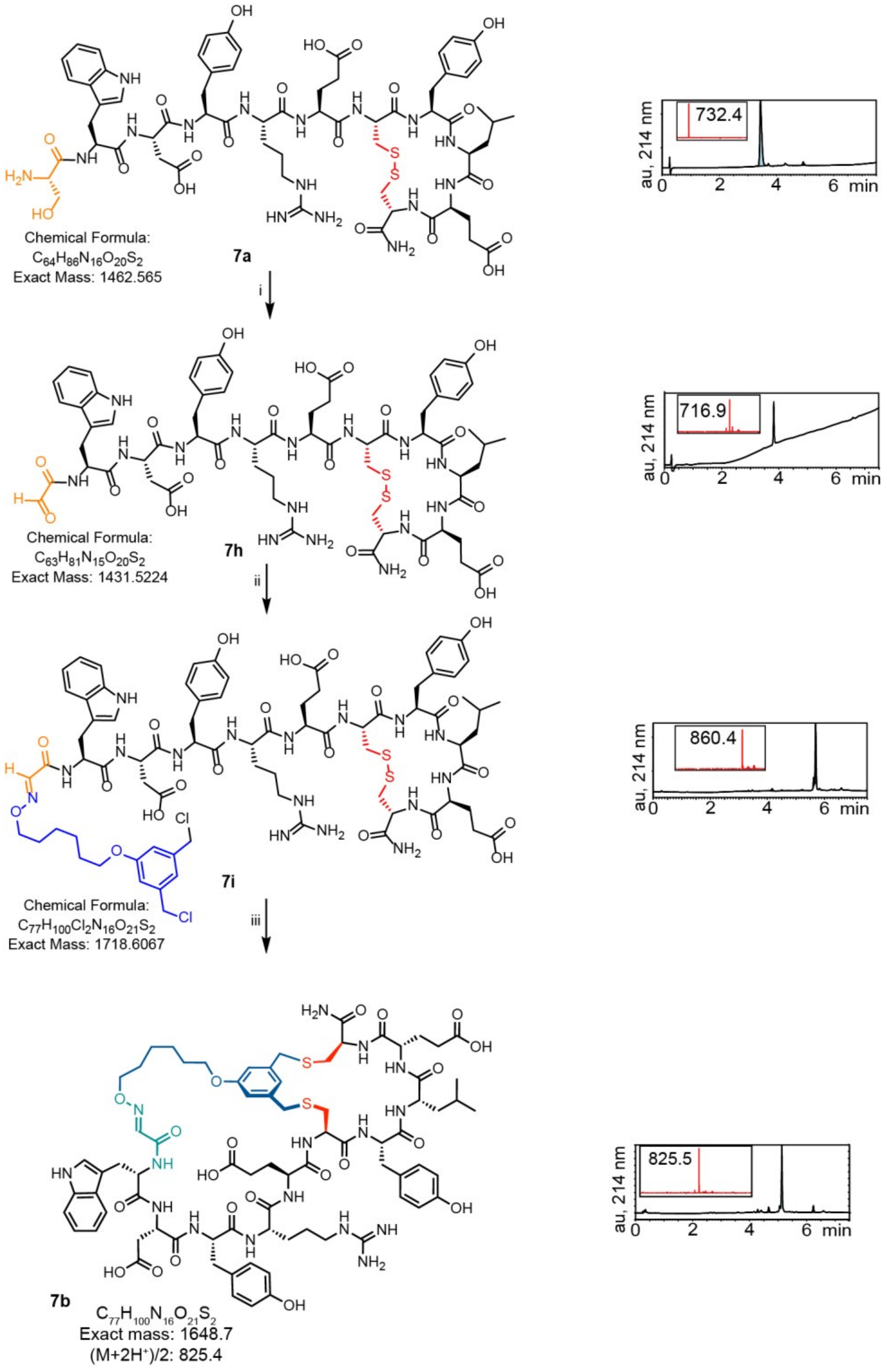

Scheme S13: One-pot bicyclization of **7a** (0.5 mM) with **TSL-1**.Reagents and conditions: (i) 0.6 mM $NaIO_4$, PBS (pH 7.4), 5 min. (ii) 0.1% TFA, 0.6 mM **TSL-1** (pH 4), 1 h; (iii) 2.5 mM TCEP, 30 min; 100 mM TRIS (pH 8.5), 1 h.

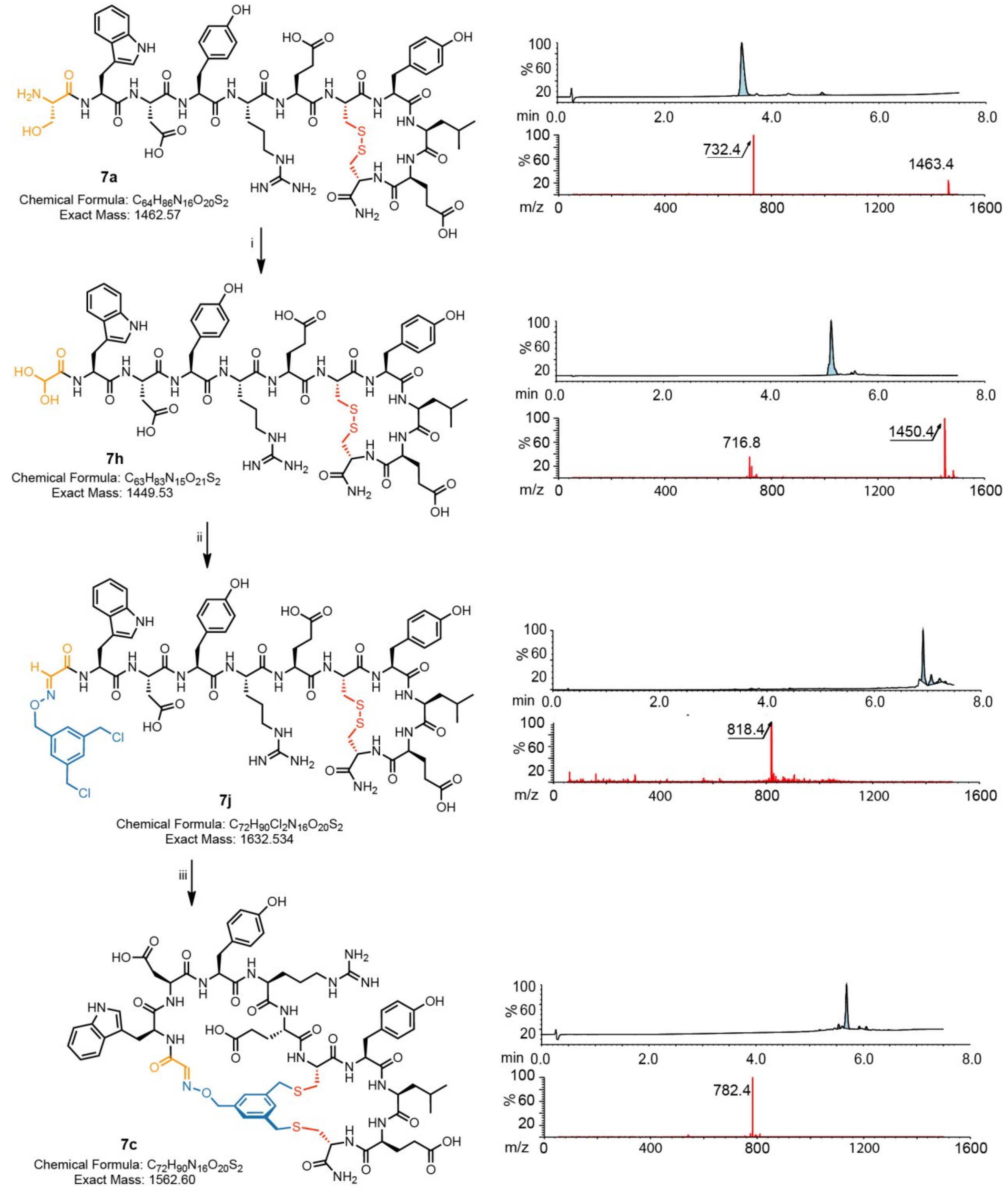

Scheme S14: One-pot bicyclization of **7a** (0.5 mM) with **TSL-1**.Reagents and conditions: (i) 0.6 mM $NaIO_4$, PBS (pH 7.4), 5 min. (ii) 0.1% TFA, 0.6 mM **TSL-1** (pH 4), 1 h; (iii) 2.5 mM TCEP, 30 min; 100 mM TRIS (pH 8.5), 1 h.

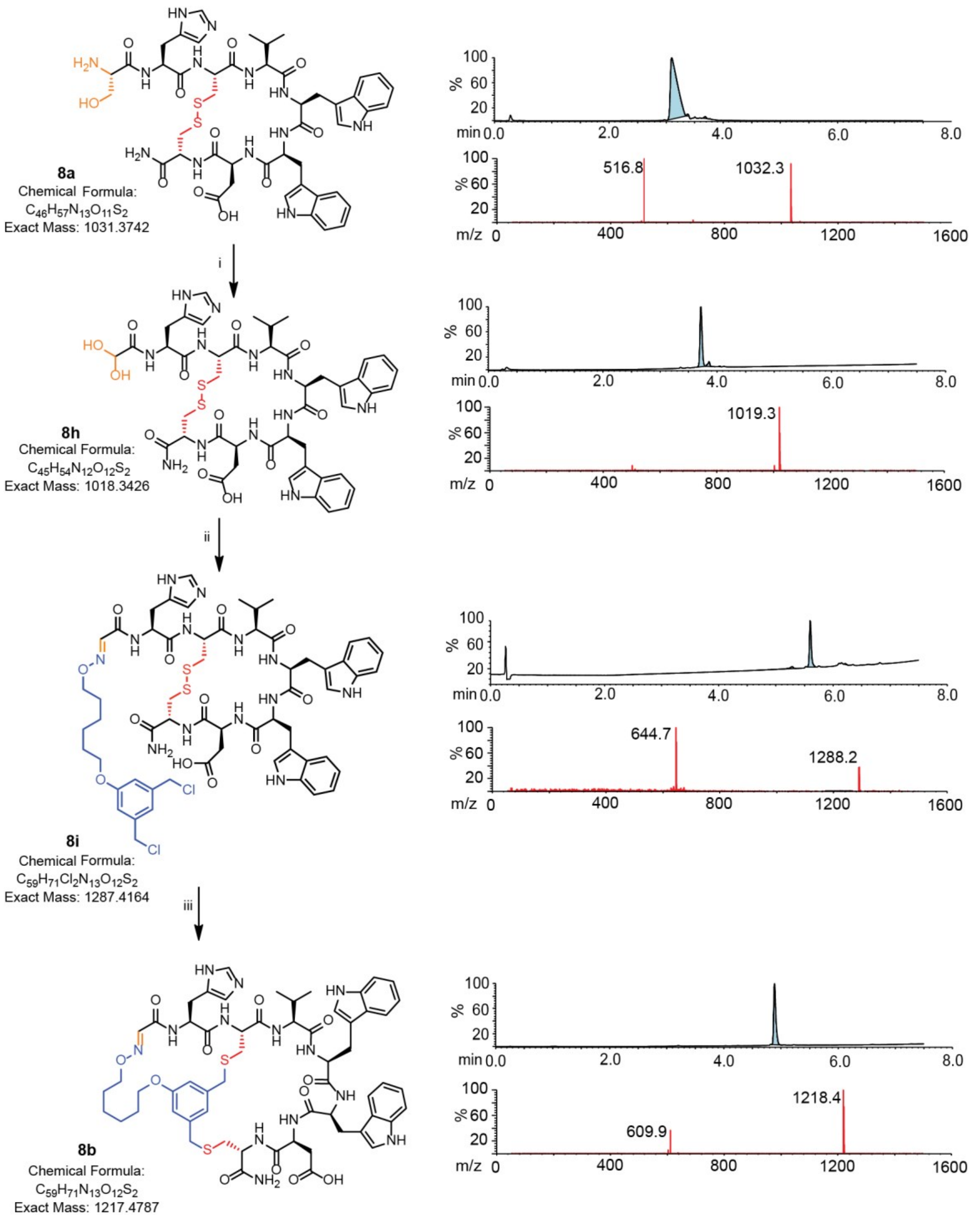

Scheme S15: One-pot bicyclization of **8a** (0.5 mM) with **TSL-6**.Reagents and conditions: (i) 0.6 mM $NaIO_4$, PBS (pH 7.4), 5 min. (ii) 0.1% TFA, 0.6 mM **TSL-6** (pH 4), 1 h; (iii) 2.5 mM TCEP, 30 min; 100 mM TRIS (pH 8.5), 1 h.

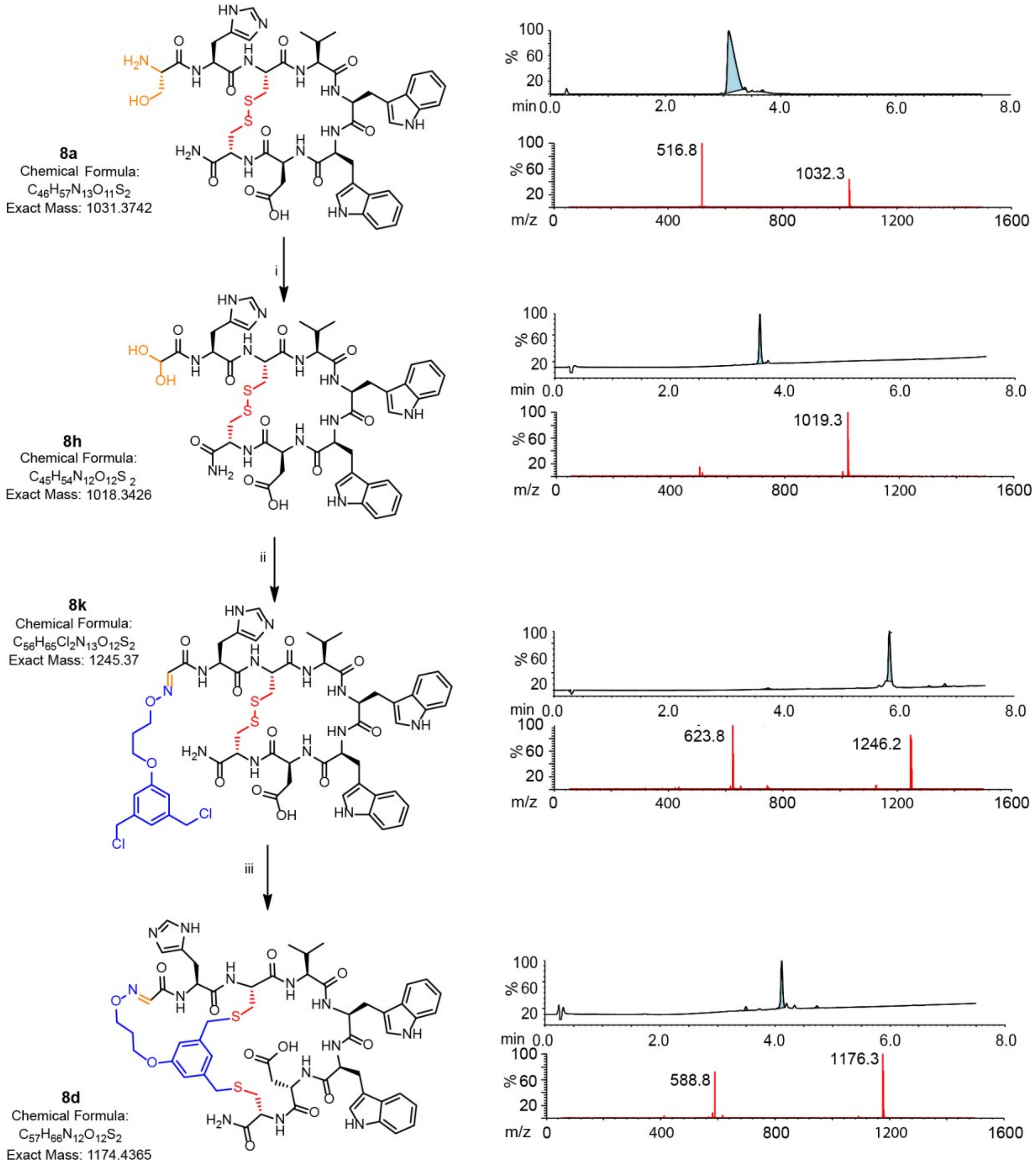

Scheme S16: One-pot bicyclization of **8a** (0.5 mM) with **TSL-3**. Reagents and conditions: (i) 0.6 mM $NaIO_4$, PBS (pH 7.4), 5 min. (ii) 0.1% TFA, 0.6 mM **TSL-3** (pH 4), 1 h; (iii) 2.5 mM TCEP, 30 min; 100 mM TRIS (pH 8.5), 1 h.

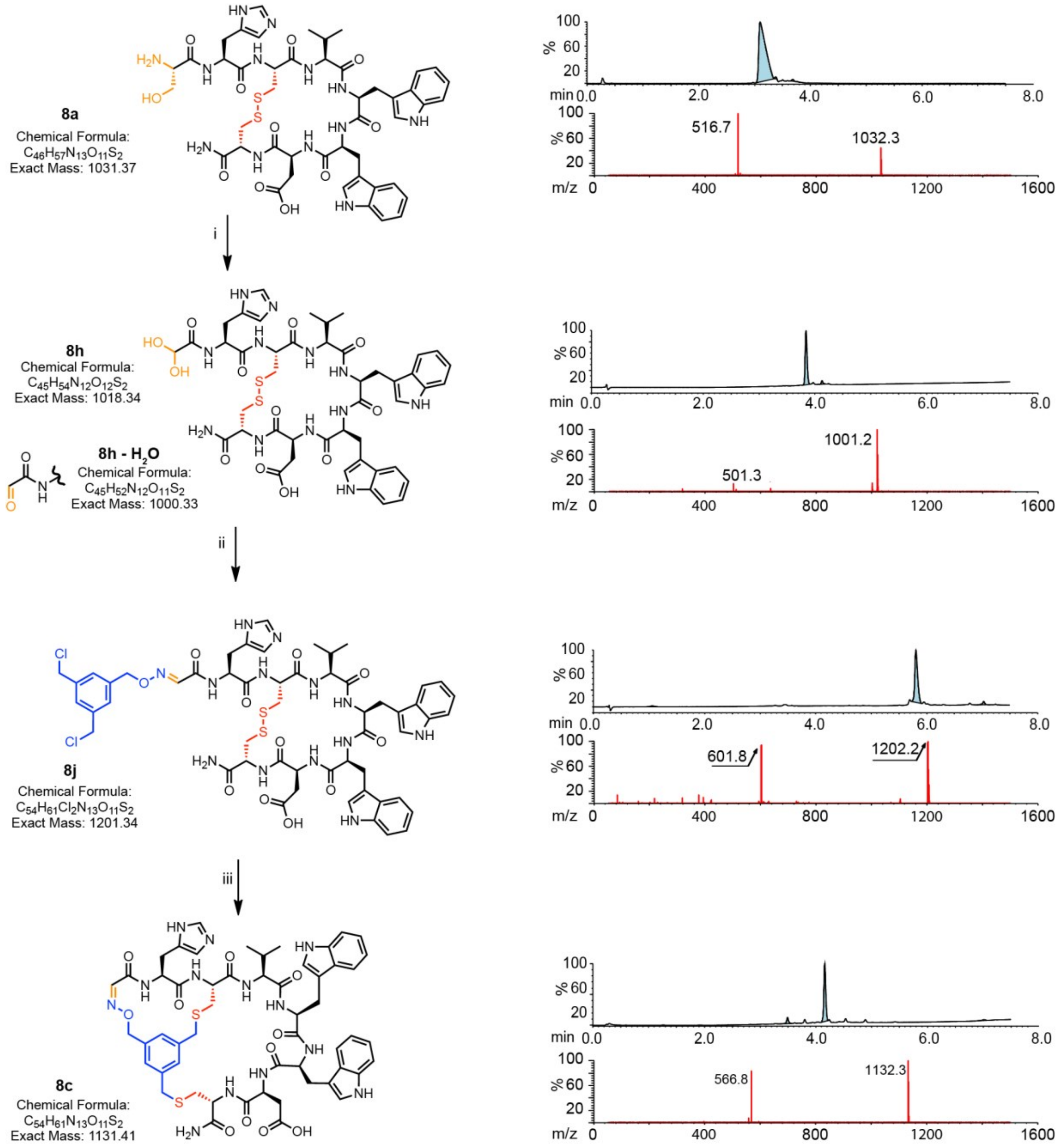

Scheme S17: One-pot bicyclization of **8a** (0.5 mM) with **TSL-1**. Reagents and conditions: (i) 0.6 mM $NaIO_4$, PBS (pH 7.4), 5 min. (ii) 0.1% TFA, 0.6 mM **TSL-1** (pH 4), 1 h; (iii) 2.5 mM TCEP, 30 min; 100 mM TRIS (pH 8.5), 1 h.

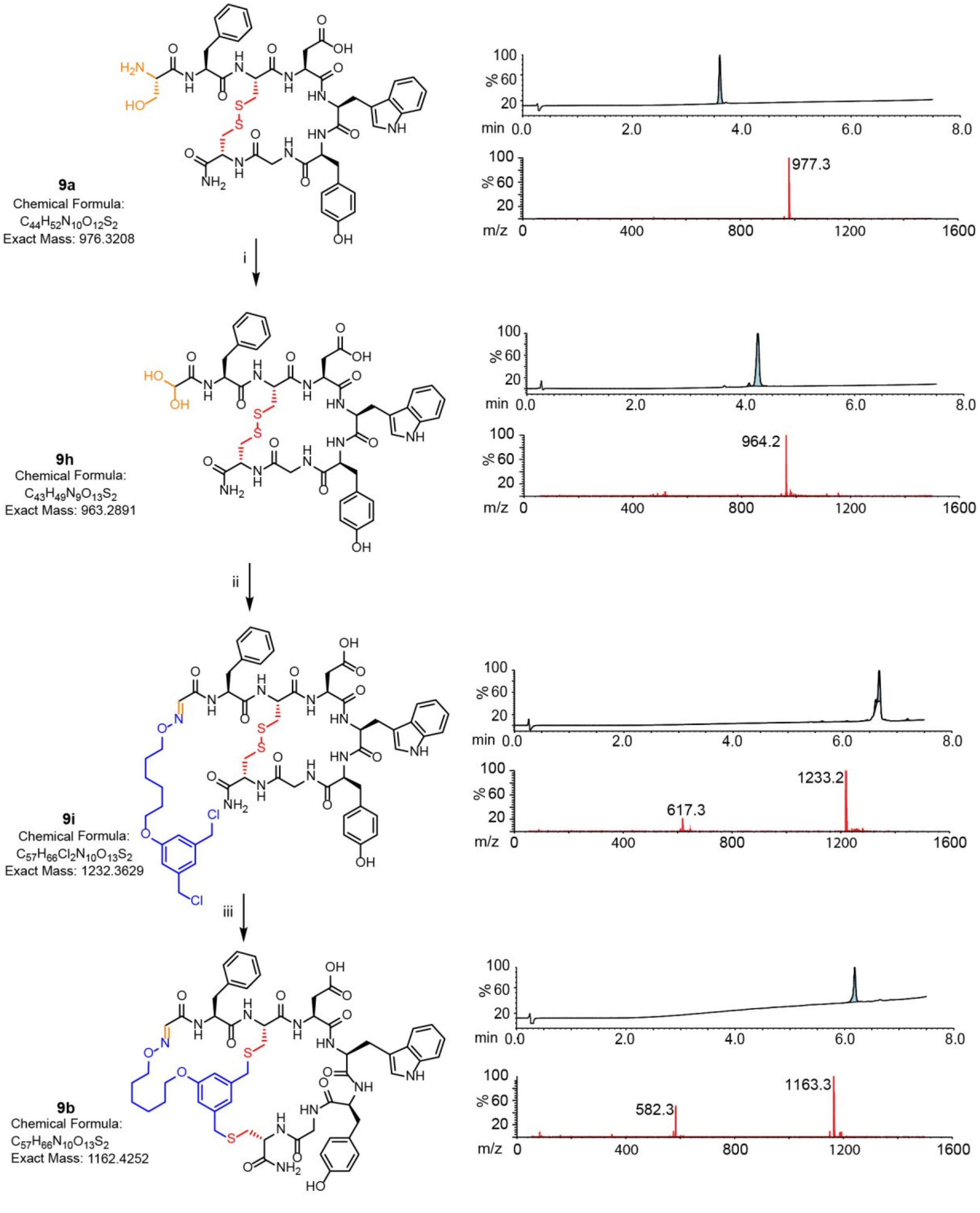

Scheme S18: One-pot bicyclization of **9a** (0.5 mM) with **TSL-1**. Reagents and conditions: (i) 0.6 mM $NaIO_4$, PBS (pH 7.4), 5 min. (ii) 0.1% TFA, 0.6 mM **TSL-1** (pH 4), 1 h; (iii) 2.5 mM TCEP, 30 min; 100 mM TRIS (pH 8.5), 1 h.

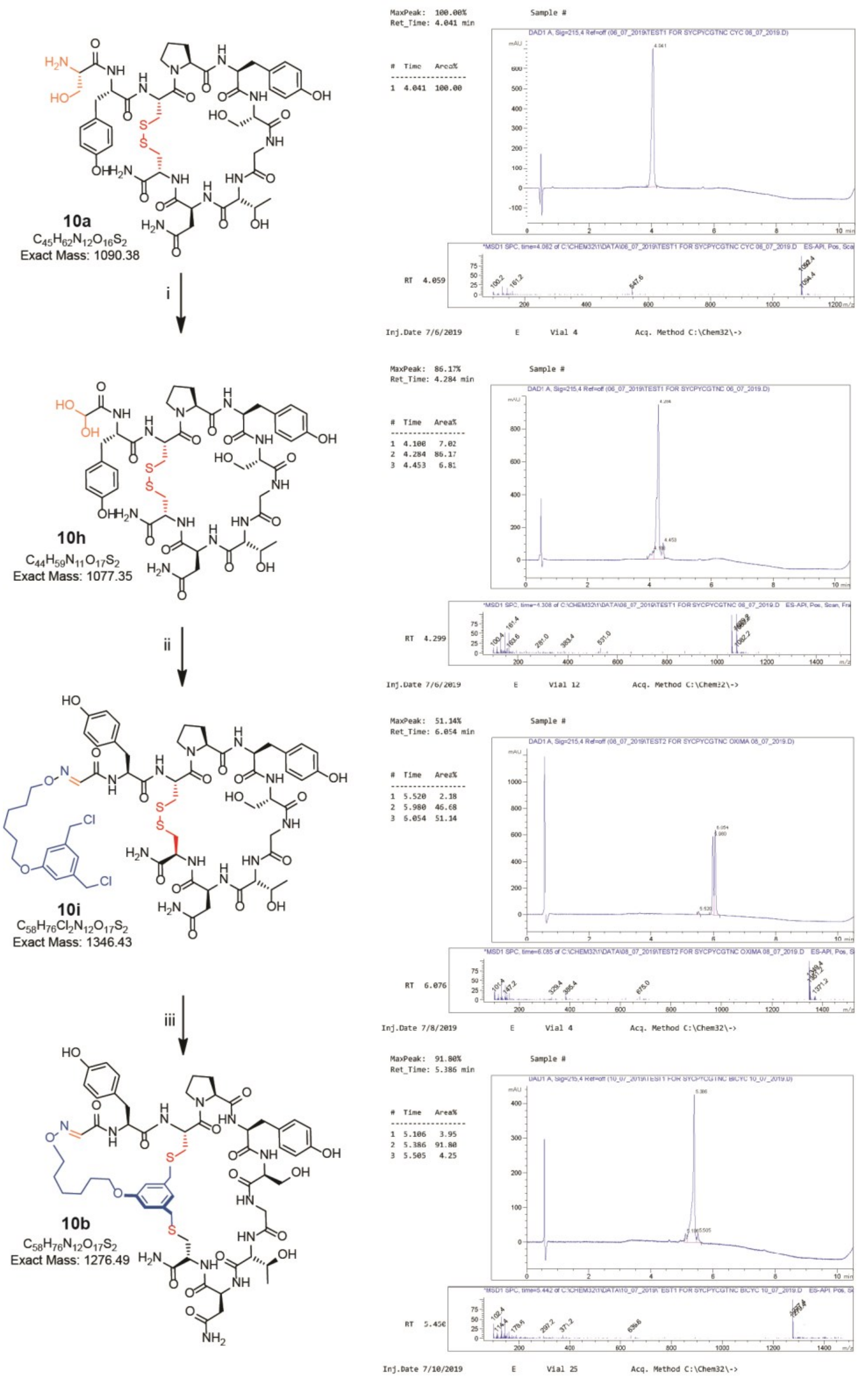

Scheme S19: Bicyclization of **10a** (10 mg, 84 nmol) with **TSL-6*:*** Reagents and conditions: (i) $NaIO_4$ (1.2 eq.), PBS (pH 7.4), 5 min. Methionine (12 eq.), 1 h. Desalting with C18 spin column (ii) 0.1% TFA, **TSL-6** (1.2 eq.), 2 h at 30 °C; (iii) TCEP (5 eq.), 1 h; 100 mM $KHCO_3$ buffer (pH 8.0), 3h; Purify w/ RP-HPLC.

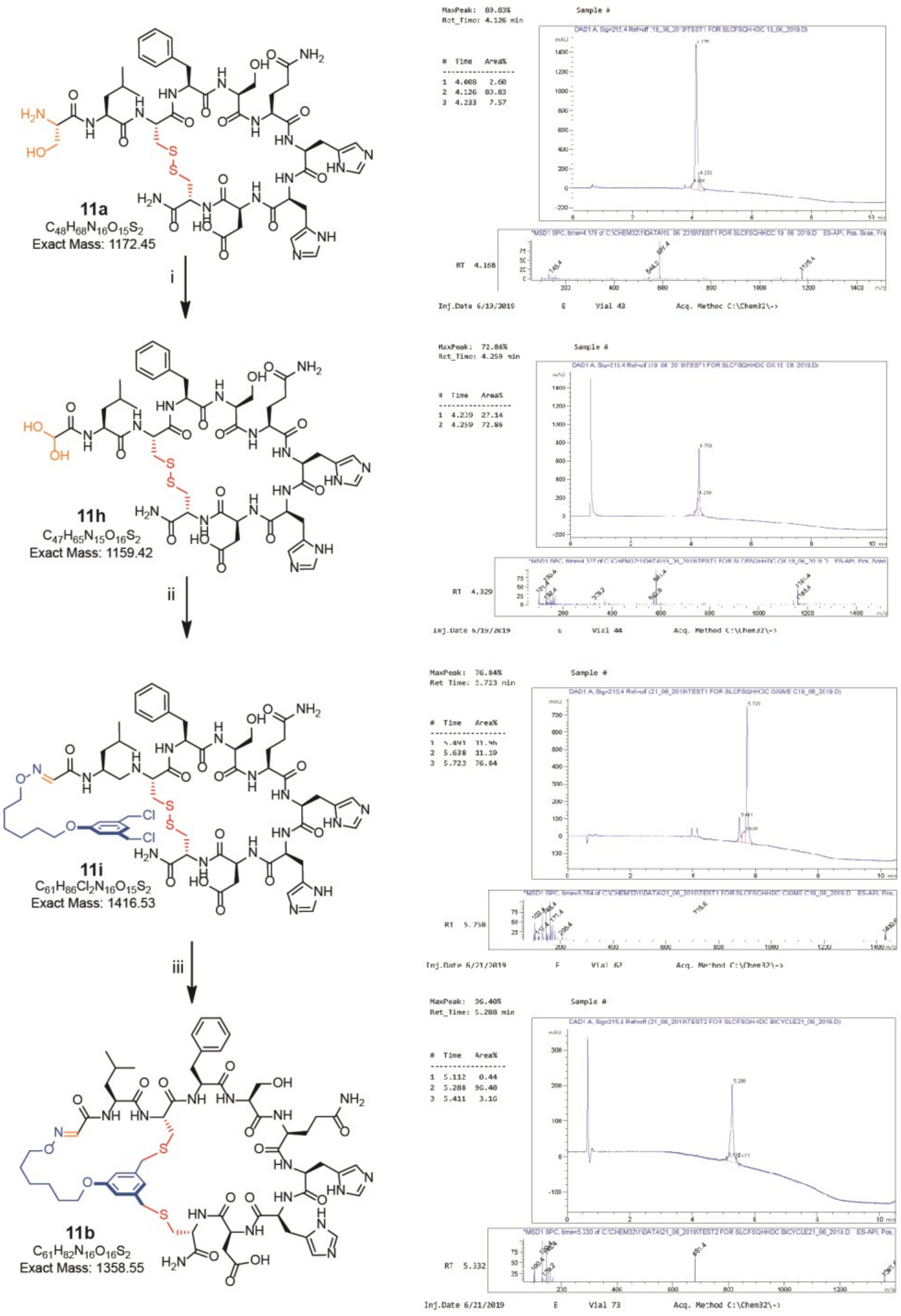

Scheme S20: Bicyclization of **11a** (10 mg, 66 nmol) with **TSL-6**: Reagents and conditions: (i) $NaIO_4$ (1.2 eq.), PBS (pH 7.4), 5 min, dark. Methionine (12 eq.), 1 h. Desalting with C18 spin column (ii) 0.1% TFA, **TSL-6** (1.2 eq.), 2 h at 30 °C; (iii) TCEP (5 eq.), 1 h; 100 mM $KHCO_3$ buffer (pH 8.0), 3h; Purify w/ RP-HPLC.

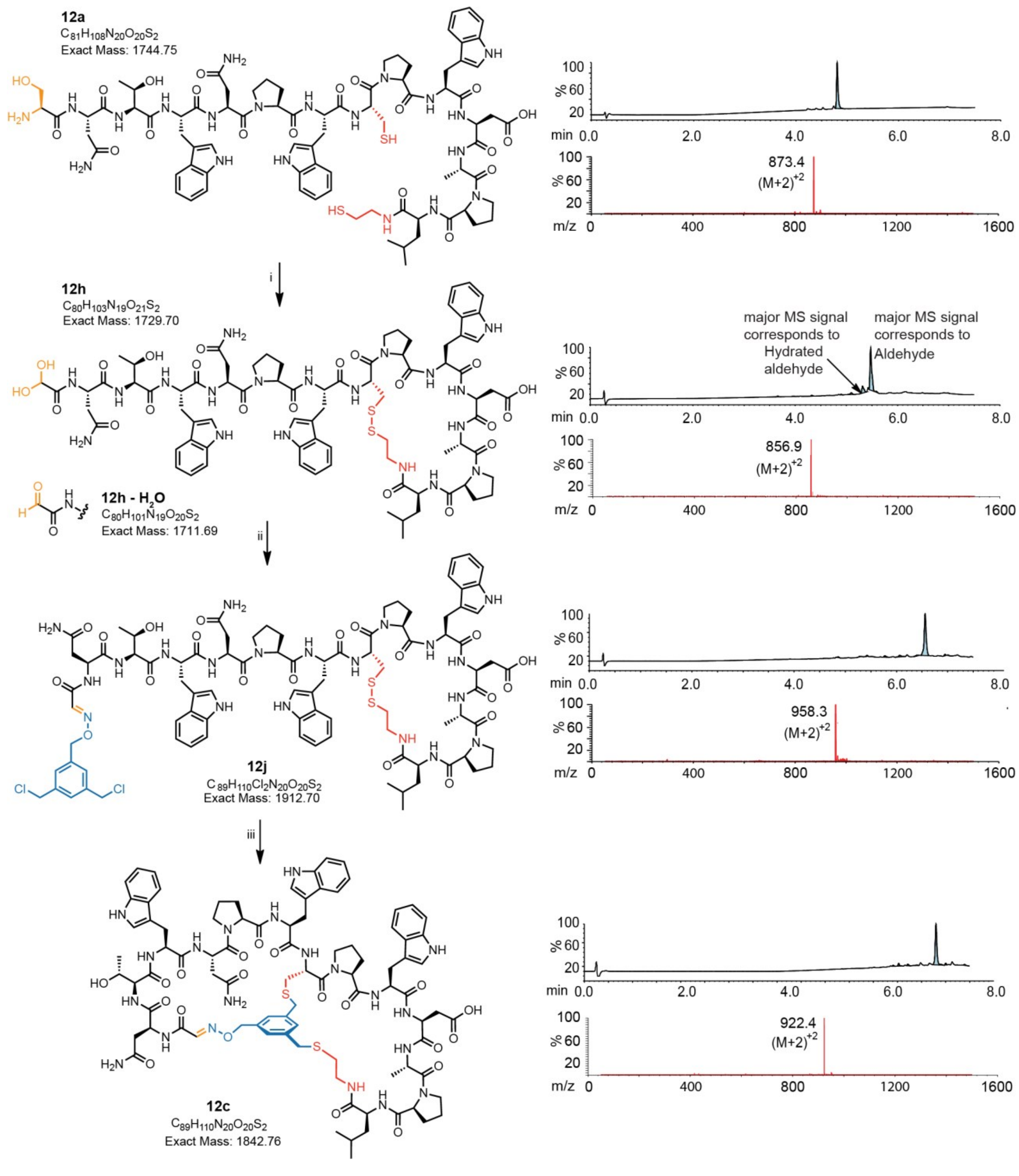

Scheme S21: One-pot bicyclization of **12a** with **TSL-1**: Reagents and conditions: (i) 0.6 mM $NaIO_4$, PBS (pH 7.4), 5 min, 1 mM methionine, 15 min (ii) 0.1% TFA, 0.6 mM **TSL-1** (pH 4), 1 h; (iii) 2.5 mM TCEP, 30 min; 100 mM bicarbonate buffer (pH 10), 30 min.

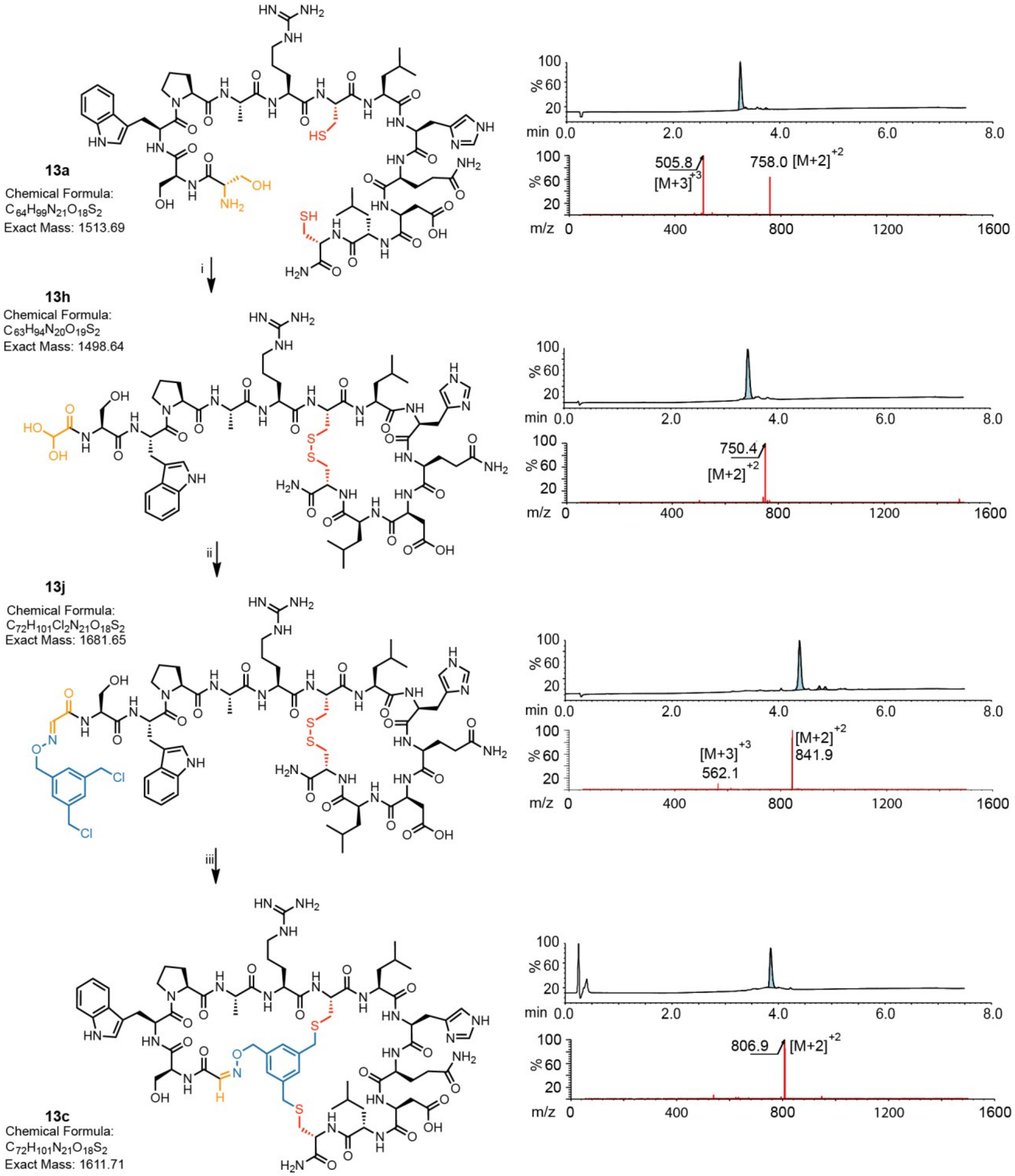

Scheme S22: One-pot bicyclization of **13a** with **TSL-1**: Reagents and conditions: (i) 0.6 mM $NaIO_4$, PBS (pH 7.4), 5 min, 1 mM methionine, 15 min (ii) 0.1% TFA, 0.6 mM **TSL-1** (pH 4), 1 h; (iii) 2.5 mM TCEP, 30 min; 100 mM bicarbonate buffer (pH 10), 1 h.

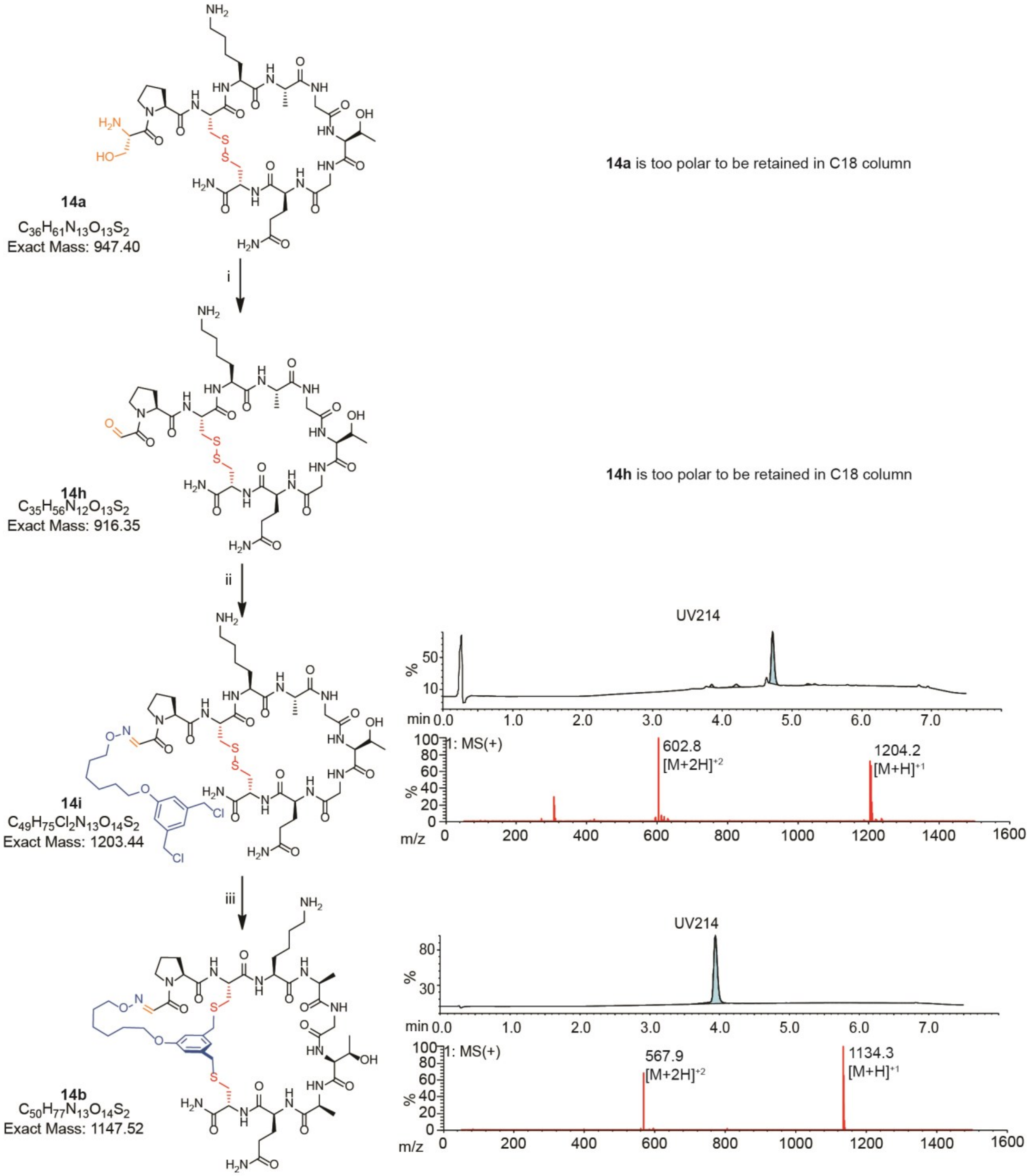

Scheme S23: Bicyclization of **14a** with **TSL-6** *:* Reagents and conditions: (i) 2.4 mM $NaIO_4$, PBS (pH 7.4), 5 min, 1 mM methionine, 15 min (ii) 0.1% TFA, 2.4 mM **TSL-6**, 1 h; (iii) 2.5 mM TCEP, 30 min; 100 mM bicarbonate buffer (pH 10), O/N; Purify w/ RP-HPLC.

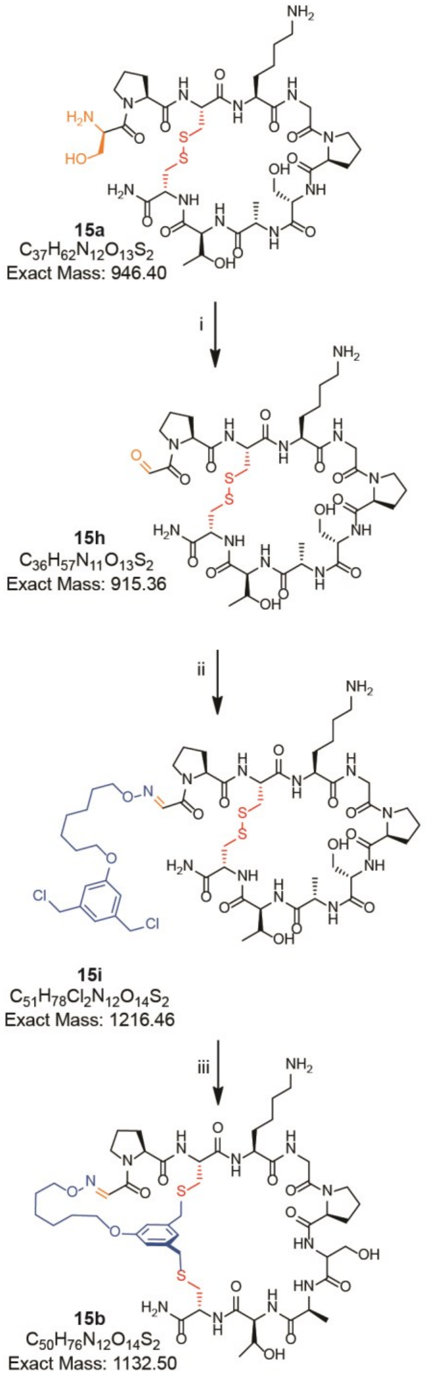

15a is too polar to be retained in C18 column

15h is too polar to be retained in C18 column

15i is too diluted to be detected

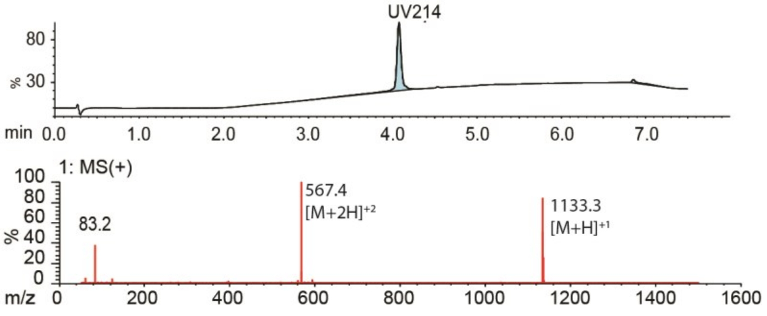

Scheme S24: Bicyclization of **15a** with **TSL-6***:* Reagents and conditions: (i) 2.4 mM $NaIO_4$, PBS (pH 7.4), 10 sec on ice, 1 mM methionine, 15 min (ii) 0.1% TFA, 2.4 mM **TSL-6**, 1 h; (iii) 2.5 mM TCEP, 30 min; 100 mM bicarbonate buffer (pH 10), O/N; Purify w/ RP-HPLC.

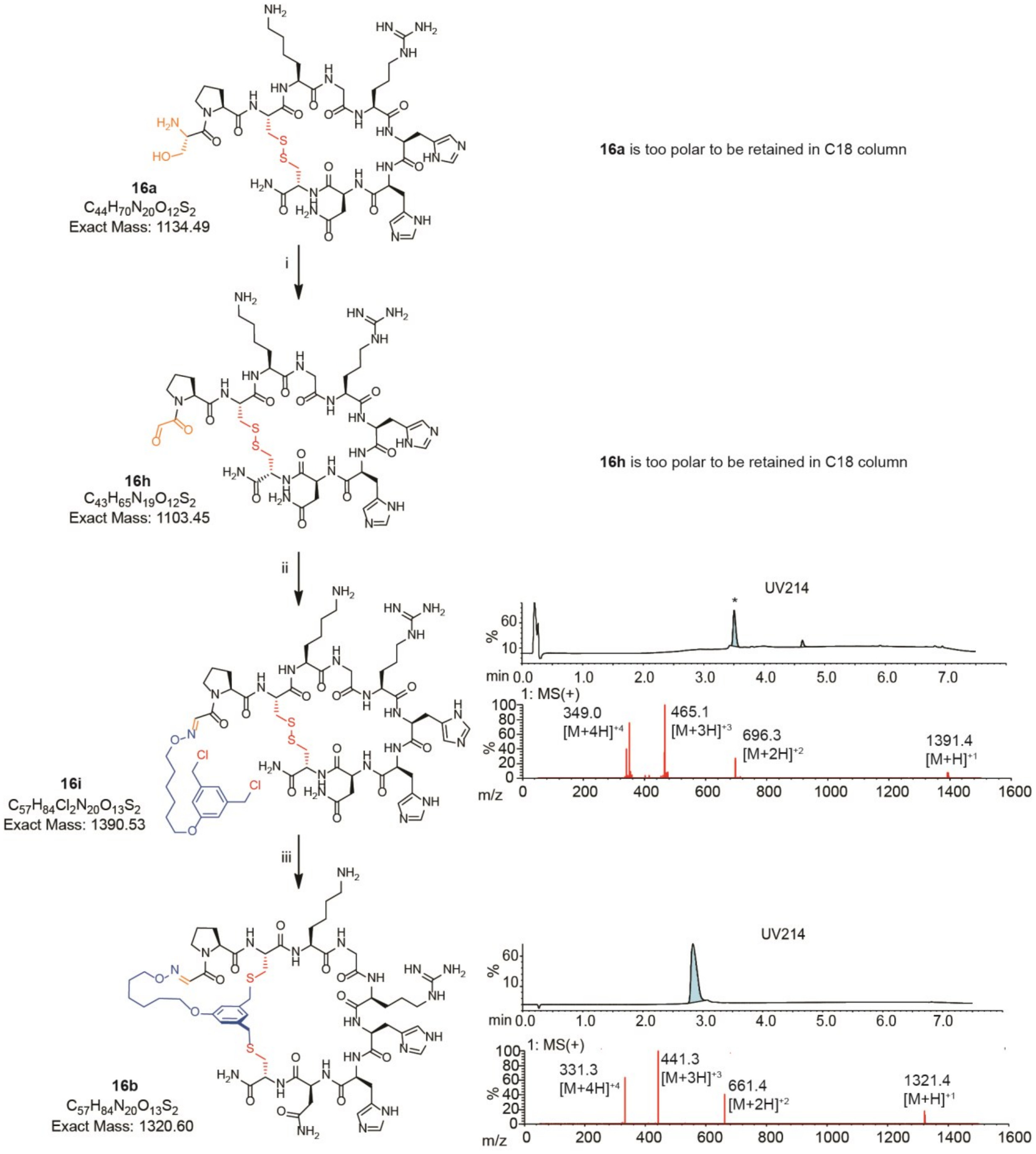

Scheme S25: Bicyclization of **16a** with **TSL-6** *:* Reagents and conditions: (i) 2.4 mM $NaIO_4$, PBS (pH 7.4), 5 min, 1 mM methionine, 15 min (ii) 0.1% TFA, 2.4 mM **TSL-6**, 1 h; (iii) 2.5 mM TCEP, 30 min; 100 mM bicarbonate buffer (pH 10), O/N; Purify w/ RP-HPLC.

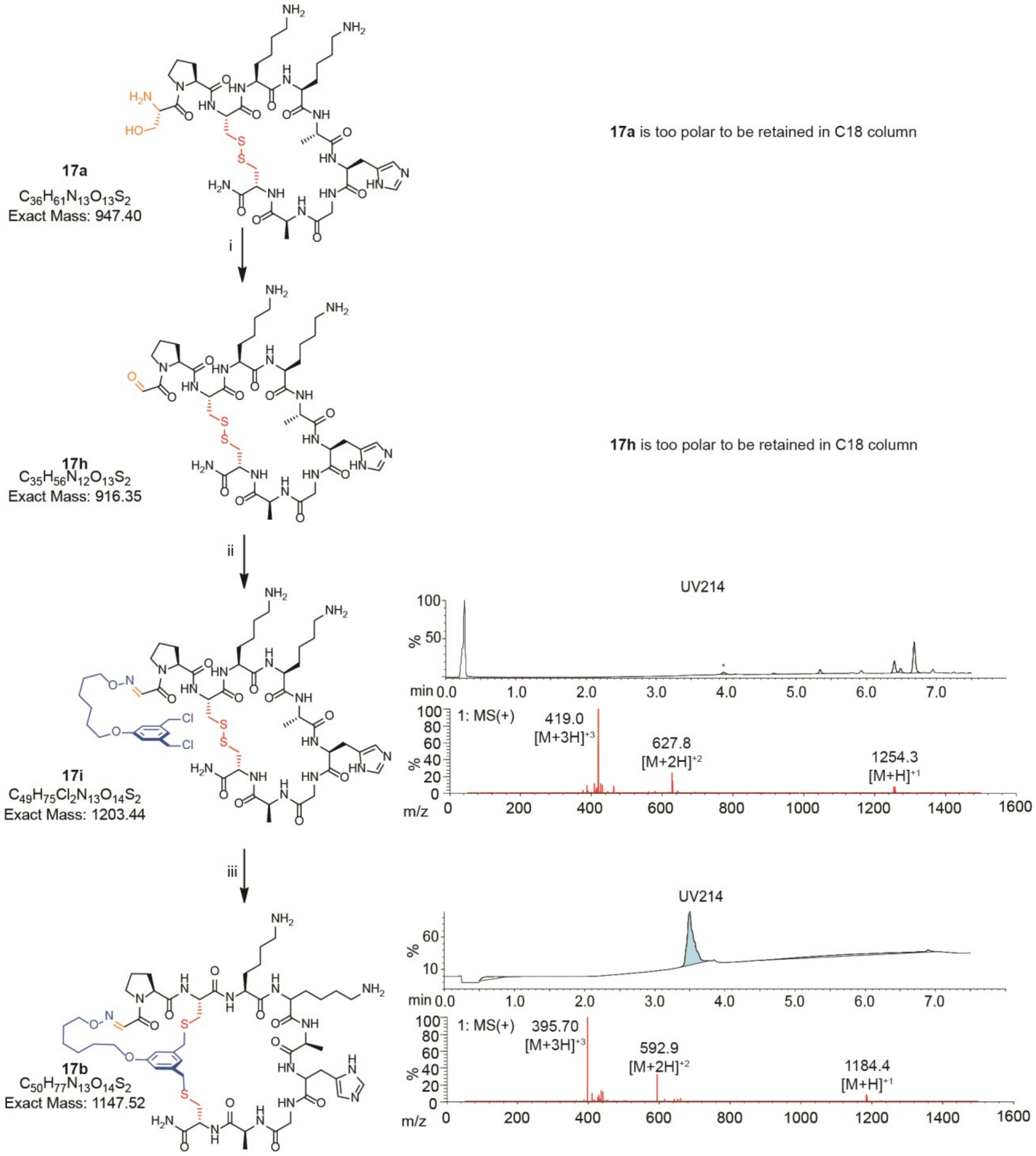

Scheme S26: Bicyclization of **17a** with **TSL-6** : Reagents and conditions: (i) 2.4 mM $NaIO_4$, PBS (pH 7.4), 5 min, 1 mM methionine, 15 min (ii) 0.1% TFA, 2.4 mM **TSL-6**, 1 h; (iii) 2.5 mM TCEP, 30 min; 100 mM bicarbonate buffer (pH 10), O/N; Purify w/ RP-HPLC.

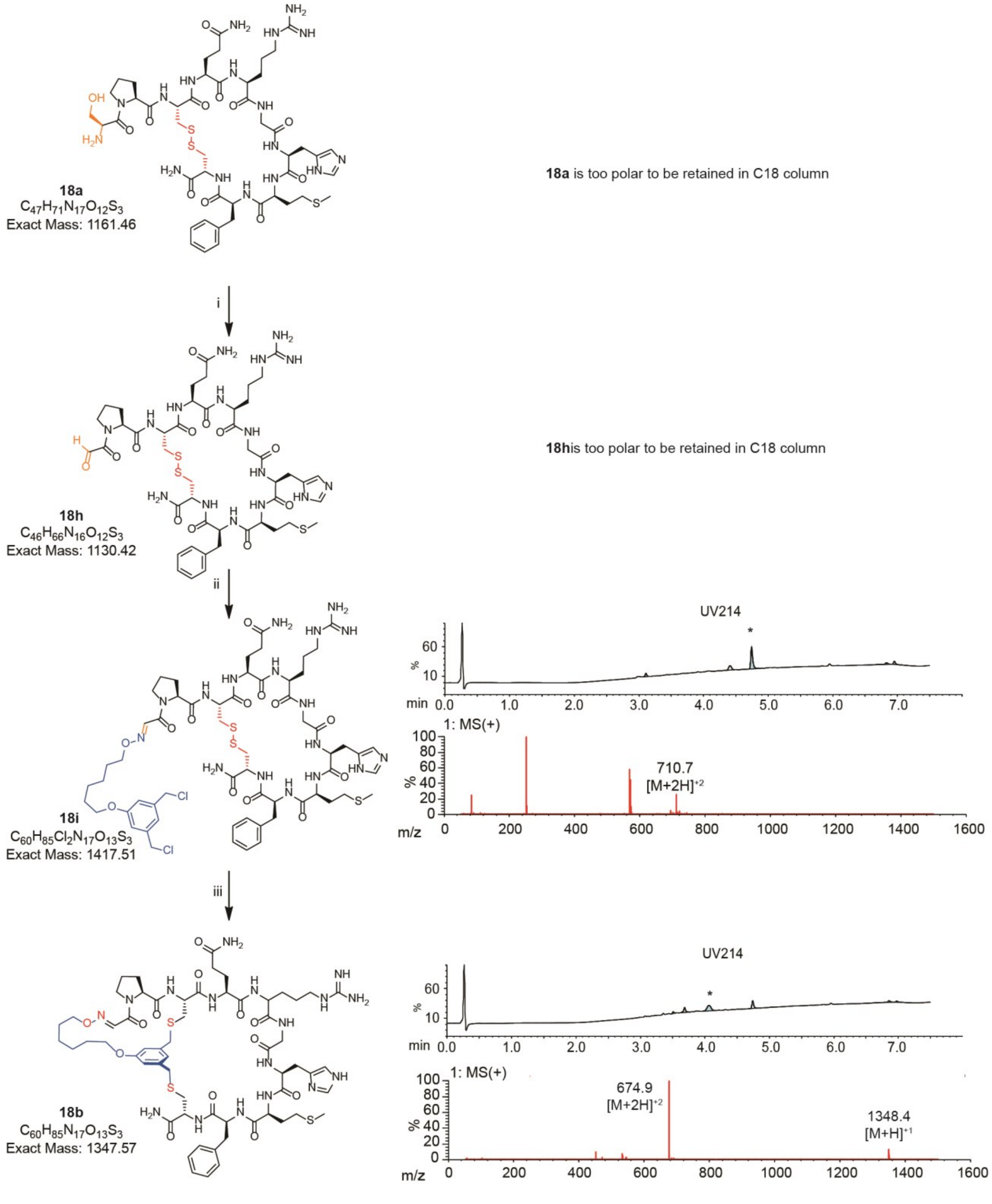

Scheme S27: Bicyclization of **18a** with **TSL-6*:*** Reagents and conditions: (i) 2.4 mM $NaIO_4$, PBS (pH 7.4), 10 sec on ice, 1 mM methionine, 15 min (ii) 0.1% TFA, 2.4 mM **TSL-6**, 1 h; (iii) 2.5 mM TCEP, 30 min; 100 mM bicarbonate buffer (pH 10), O/N; Purify w/ RP-HPLC.

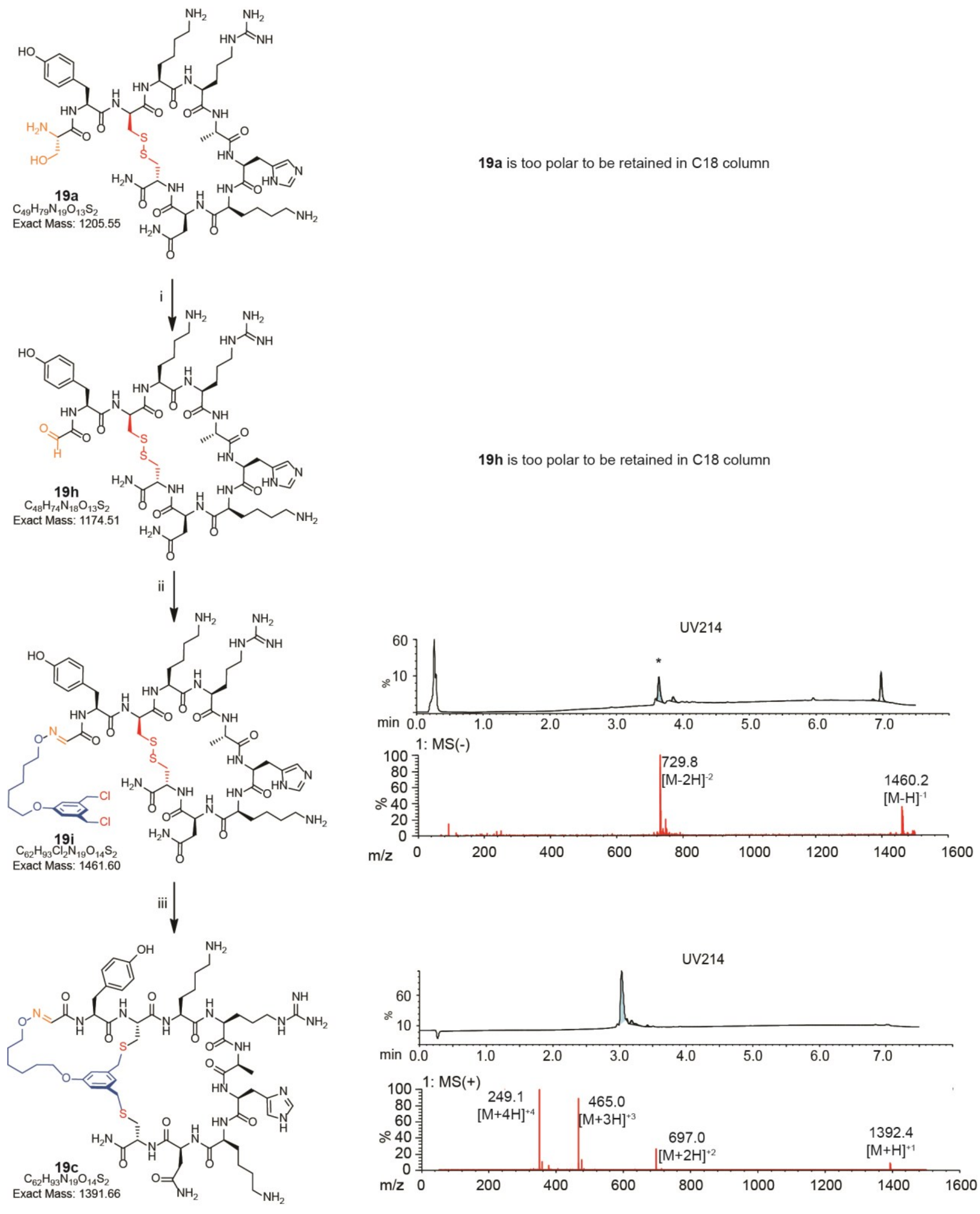

Scheme S28: Bicyclization of **19a** with **TSL-6***:* Reagents and conditions: (i) 2.4 mM $NaIO_4$, PBS (pH 7.4), 5 min, 1 mM methionine, 15 min (ii) 0.1% TFA, 2.4 mM **TSL-6**, 1 h; (iii) 2.5 mM TCEP, 30 min; 100 mM bicarbonate buffer (pH 10), O/N; Purify w/ RP-HPLC.

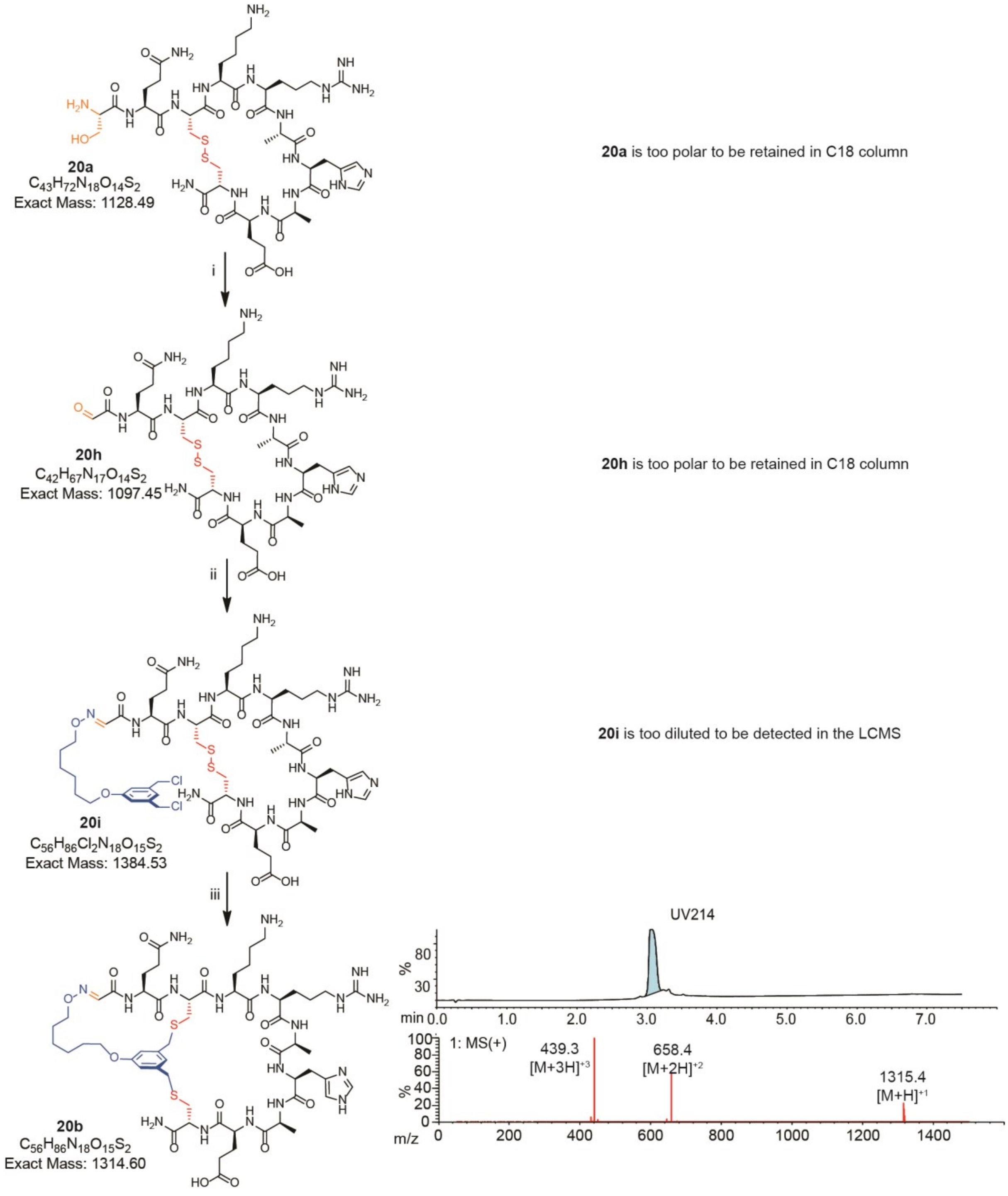

Scheme S29: Bicyclization of **20a** with **TSL-6***:* Reagents and conditions: (i) 2.4 mM $NaIO_4$, PBS (pH 7.4), 5 min, 1 mM methionine, 15 min (ii) 0.1% TFA, 2.4 mM **TSL-6**, 1 h; (iii) 2.5 mM TCEP, 30 min; 100 mM bicarbonate buffer (pH 10), O/N; Purify w/ RP-HPLC.

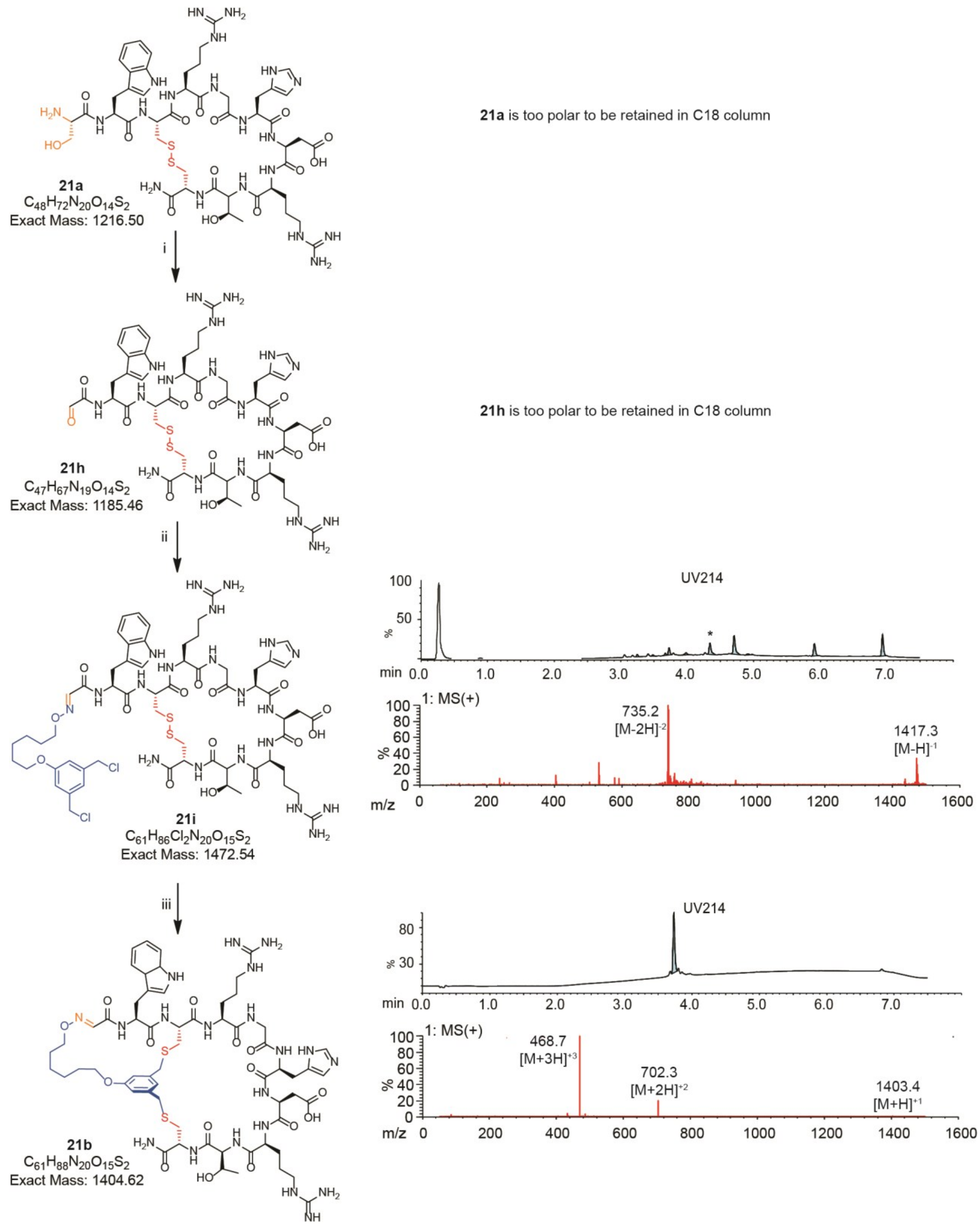

Scheme S30: Bicyclization of **21a** with **TSL-6***:* Reagents and conditions: (i) 2.4 mM $NaIO_4$, PBS (pH 7.4), 5 min, 1 mM methionine, 15 min (ii) 0.1% TFA, 2.4 mM **TSL-6**, 1 h; (iii) 2.5 mM TCEP, 30 min; 100 mM bicarbonate buffer (pH 10), O/N; Purify w/ RP-HPLC.

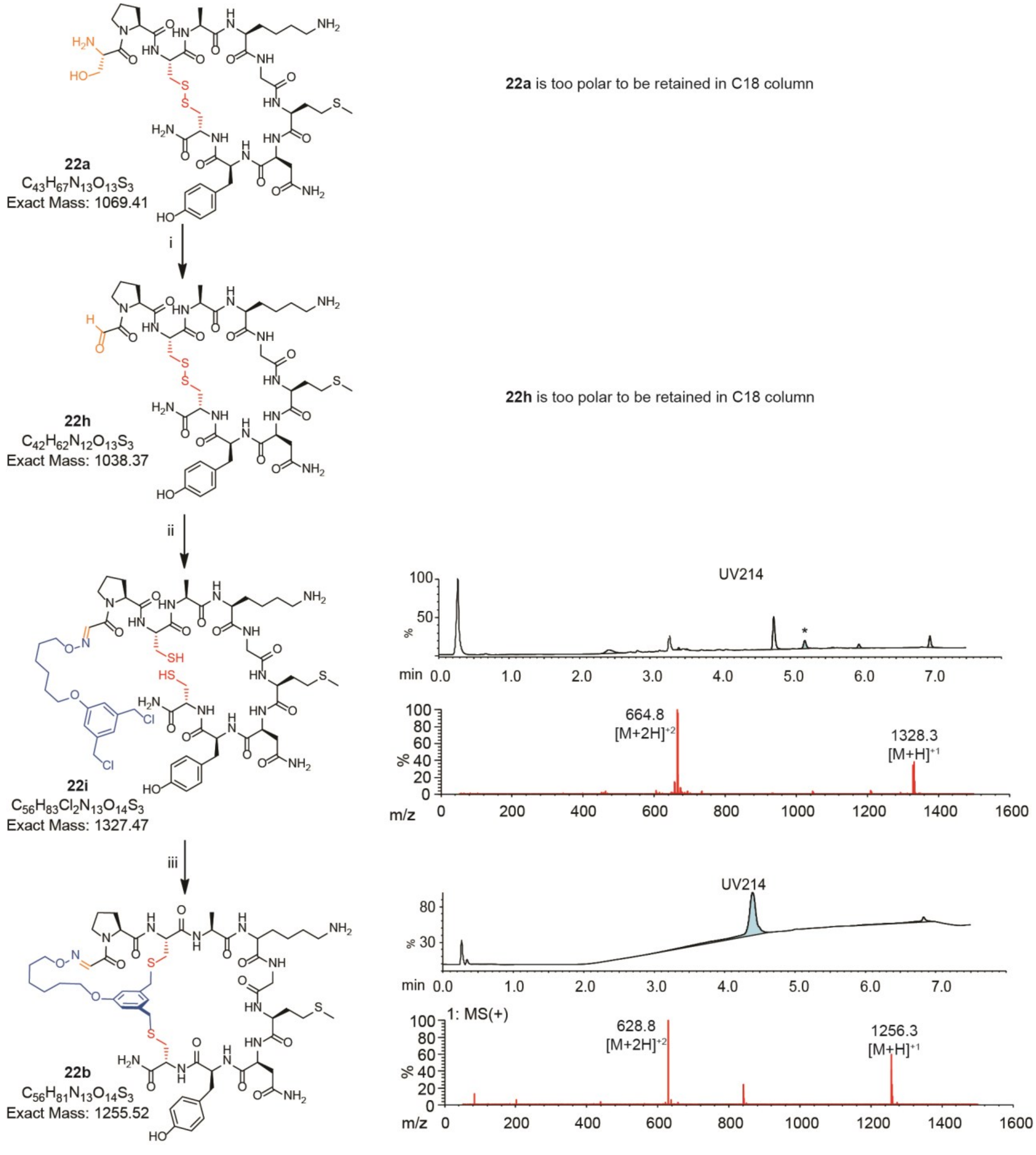

Scheme S31: Bicyclization of **22a** with **TSL-6**: Reagents and conditions: (i) 2.4 mM $NaIO_4$, PBS (pH 7.4), 5 min, 1 mM methionine, 15 min (ii) 0.1% TFA, 2.4 mM **TSL-6**, 1 h; 2.5 mM TCEP, 30 min; (iii) 100 mM bicarbonate buffer (pH 10), O/N; Purify w/ RP-HPLC

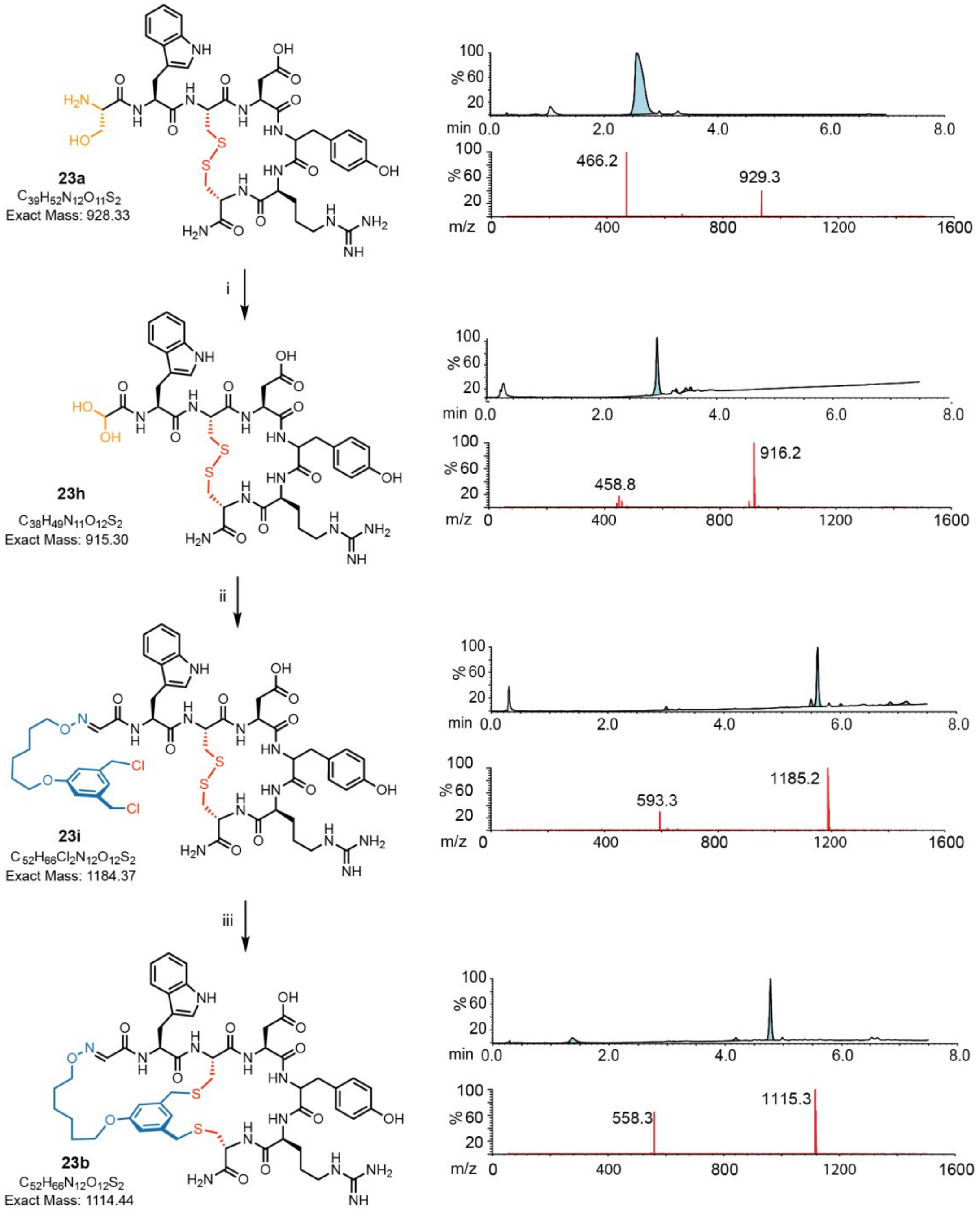

Scheme S32: One-pot bicyclization of **23a** (0.5 mM) with **TSL-6**. Reagents and conditions: (i) 0.6 mM $NaIO_4$, PBS (pH 7.4), 5 min. (ii) 0.1% TFA, 0.6 mM **TSL-6** (pH 4), 1 h; (iii) 2.5 mM TCEP, 30 min; 100 mM TRIS (pH 8.5), 1 h.

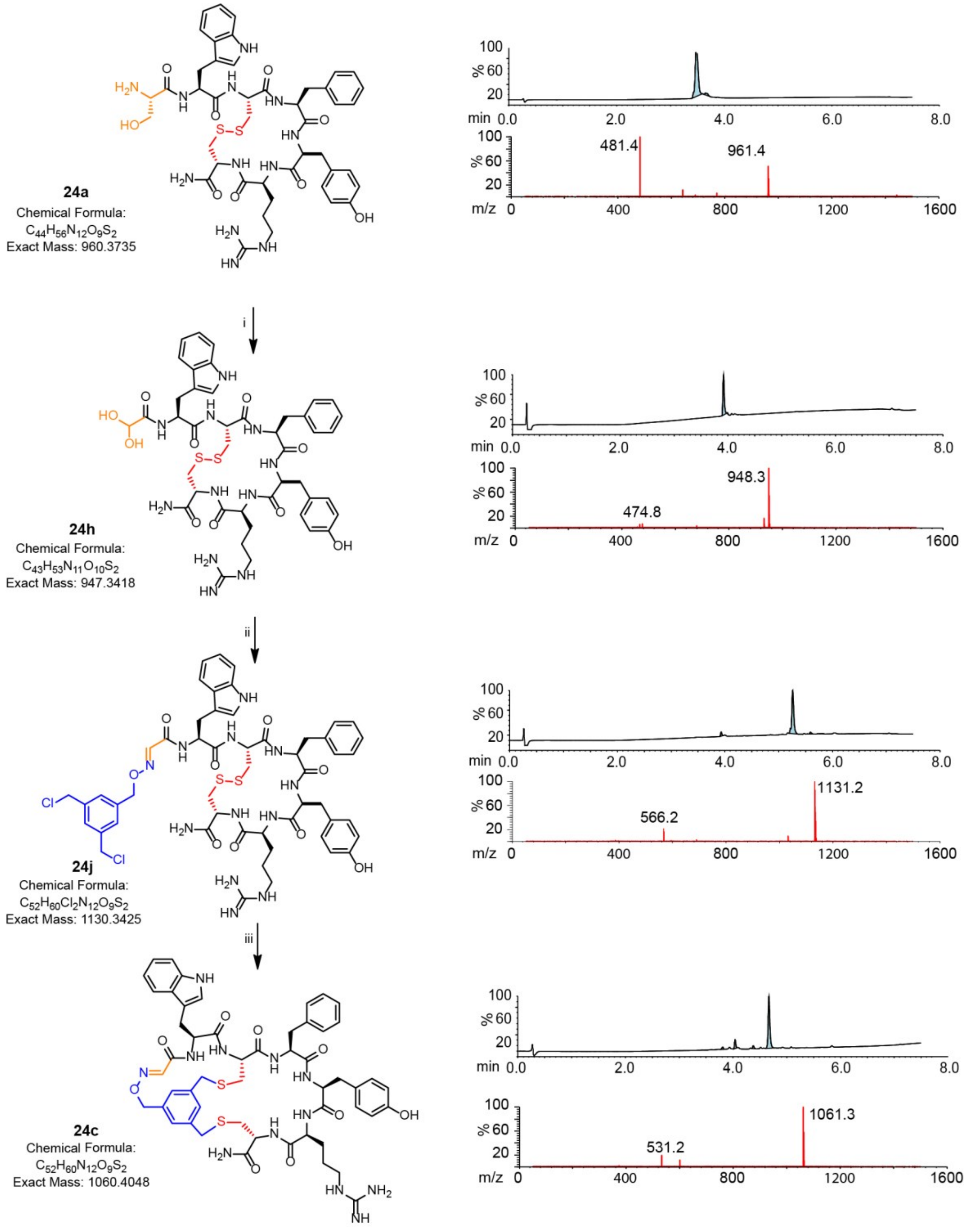

Scheme S33: One-pot bicyclization of **24a** (0.5 mM) with **TSL-6**. Reagents and conditions: (i) 0.6 mM $NaIO_4$, PBS (pH 7.4), 5 min. (ii) 0.1% TFA, 0.6 mM **TSL-6** (pH 4), 1 h; (iii) 2.5 mM TCEP, 30 min; 100 mM TRIS (pH 8.5), 1 h.

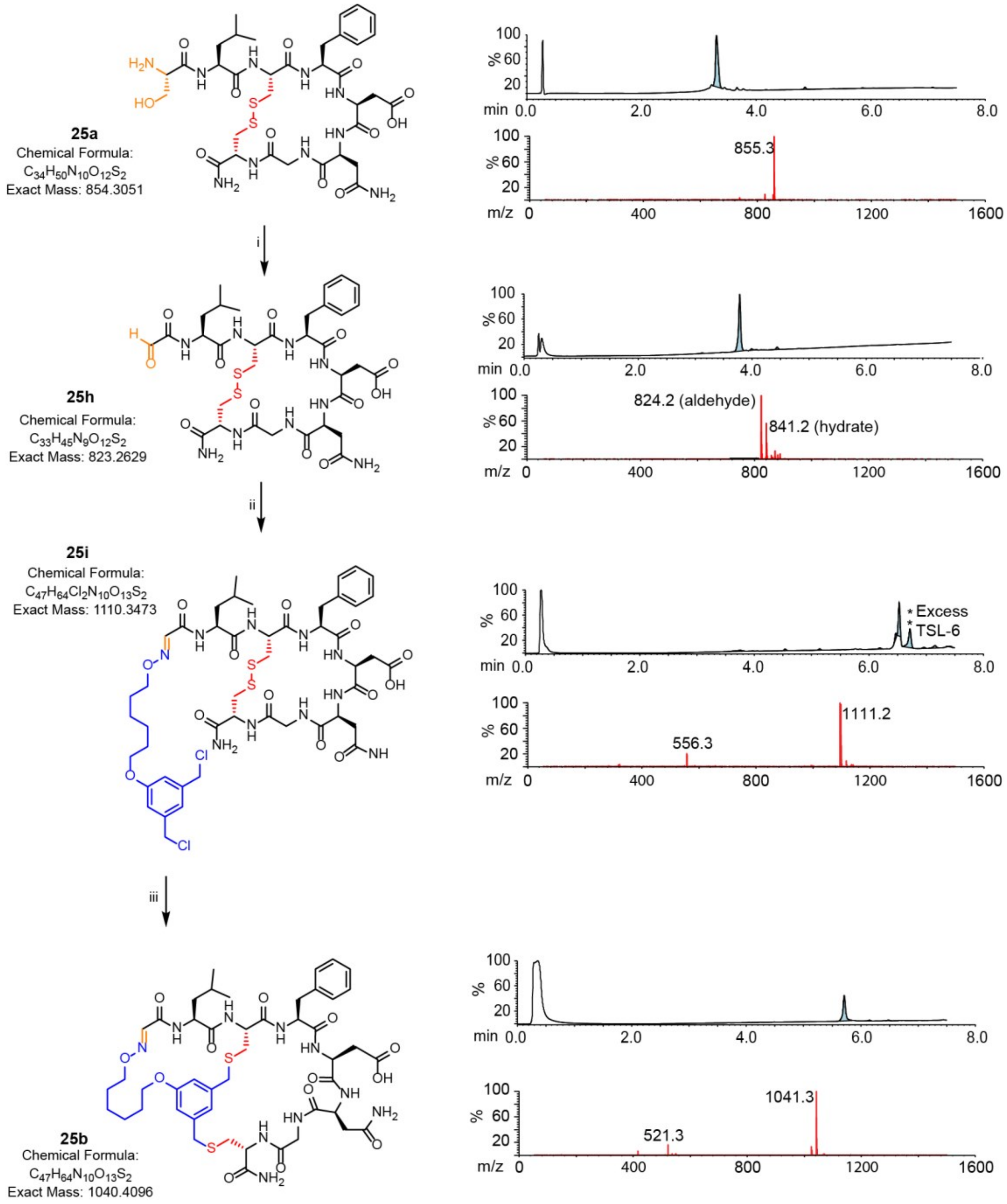

Scheme S34: One-pot bicyclization of **25a** (0.5 mM) with **TSL-6**. Reagents and conditions: (i) 0.6 mM $NaIO_4$, PBS (pH 7.4), 5 min. (ii) 0.1% TFA, 0.6 mM **TSL-6** (pH 4), 1 h; (iii) 2.5 mM TCEP, 30 min; 100 mM TRIS (pH 8.5), 1 h.

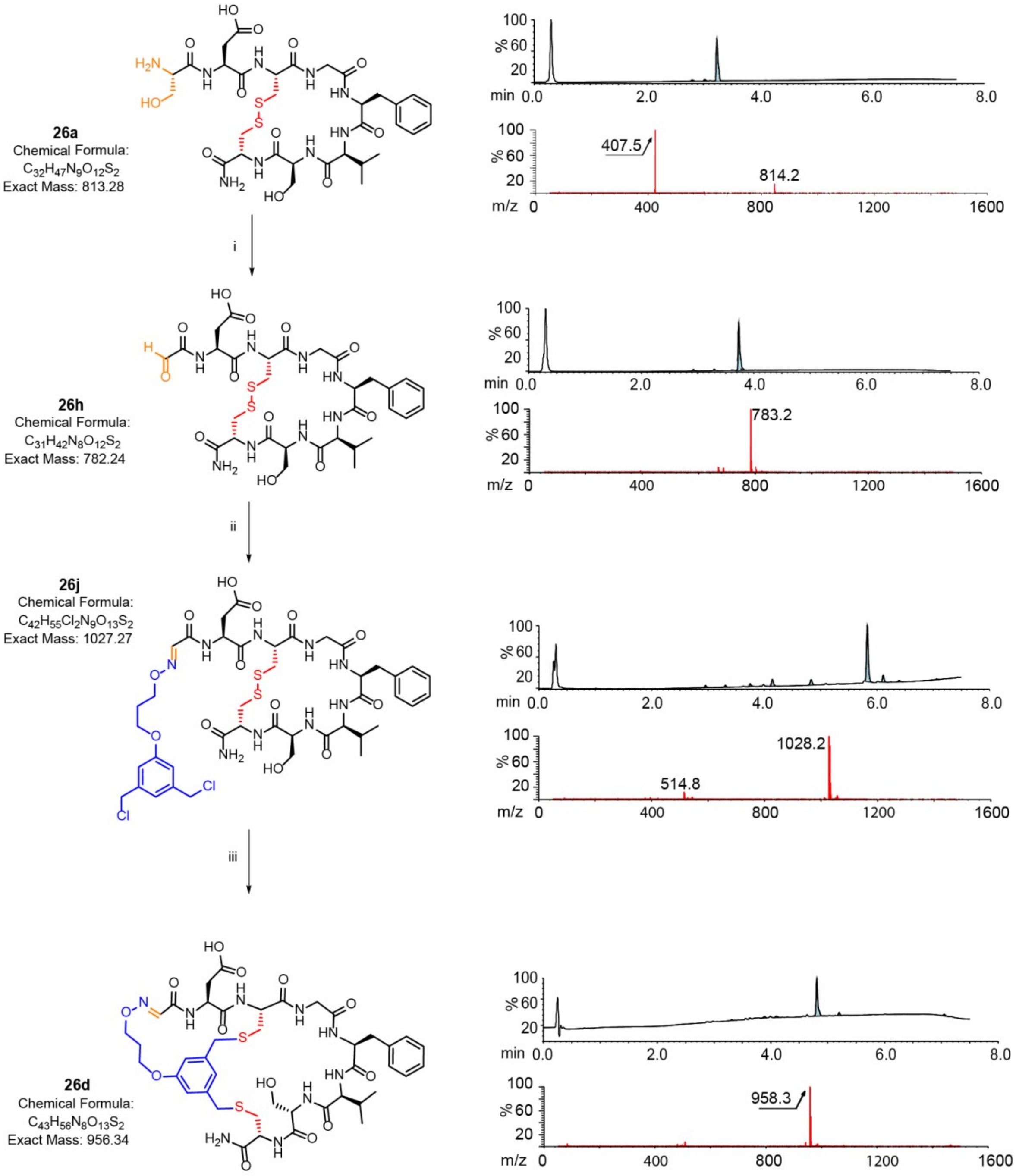

Scheme S35: One-pot bicyclization of **26a** (0.5 mM) with **TSL-3**. Reagents and conditions: (i) 0.6 mM $NaIO_4$, PBS (pH 7.4), 5 min. (ii) 0.1% TFA, 0.6 mM **TSL-3** (pH 4), 1 h; (iii) 2.5 mM TCEP, 30 min; 100 mM TRIS (pH 8.5), 1 h.

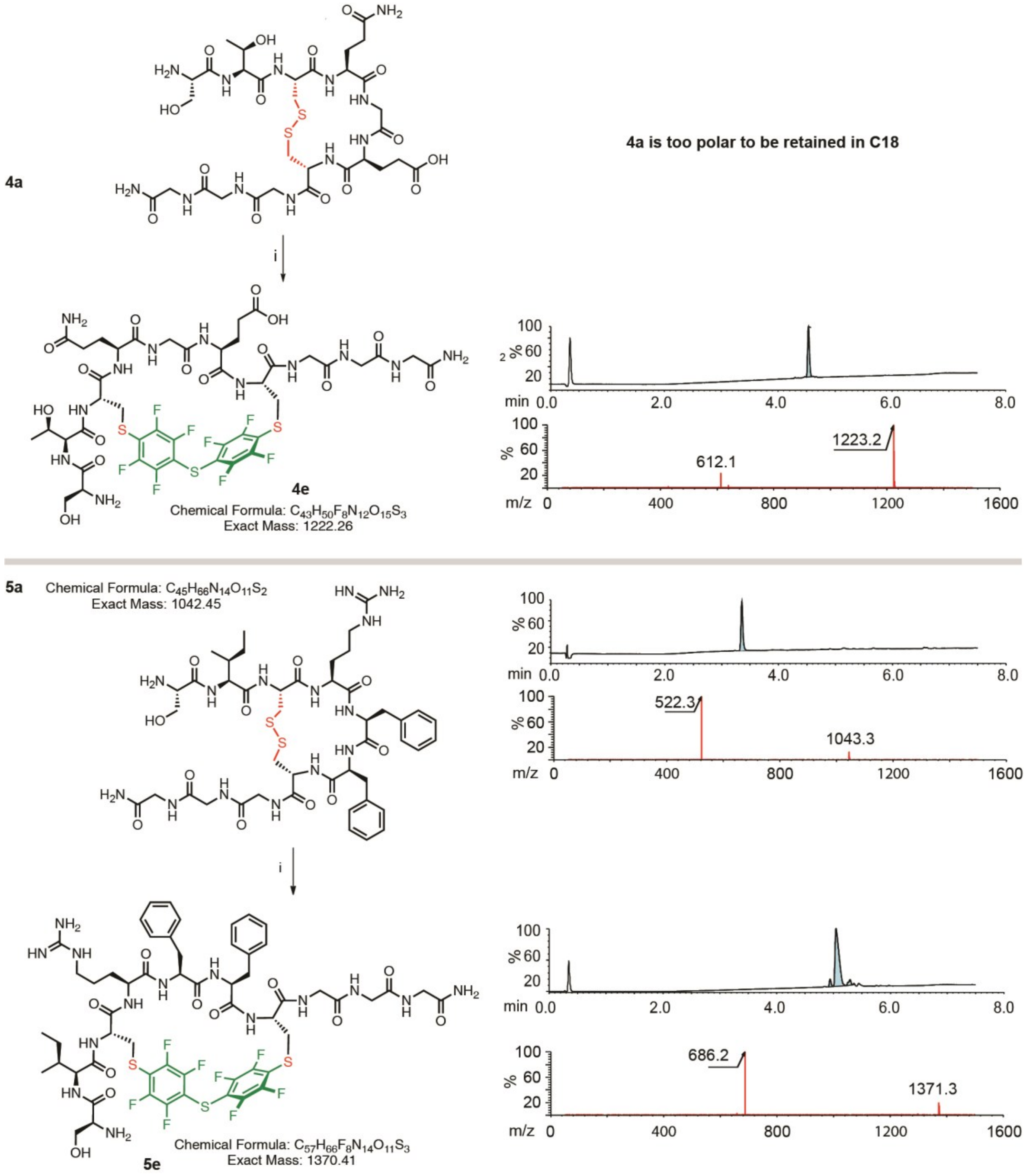

Scheme S36: Cyclization of **4a** and **5a** with **PFS** : For reagents and conditions see General Protocol for cyclization with perflurodiphenylsufide (**PFS**).

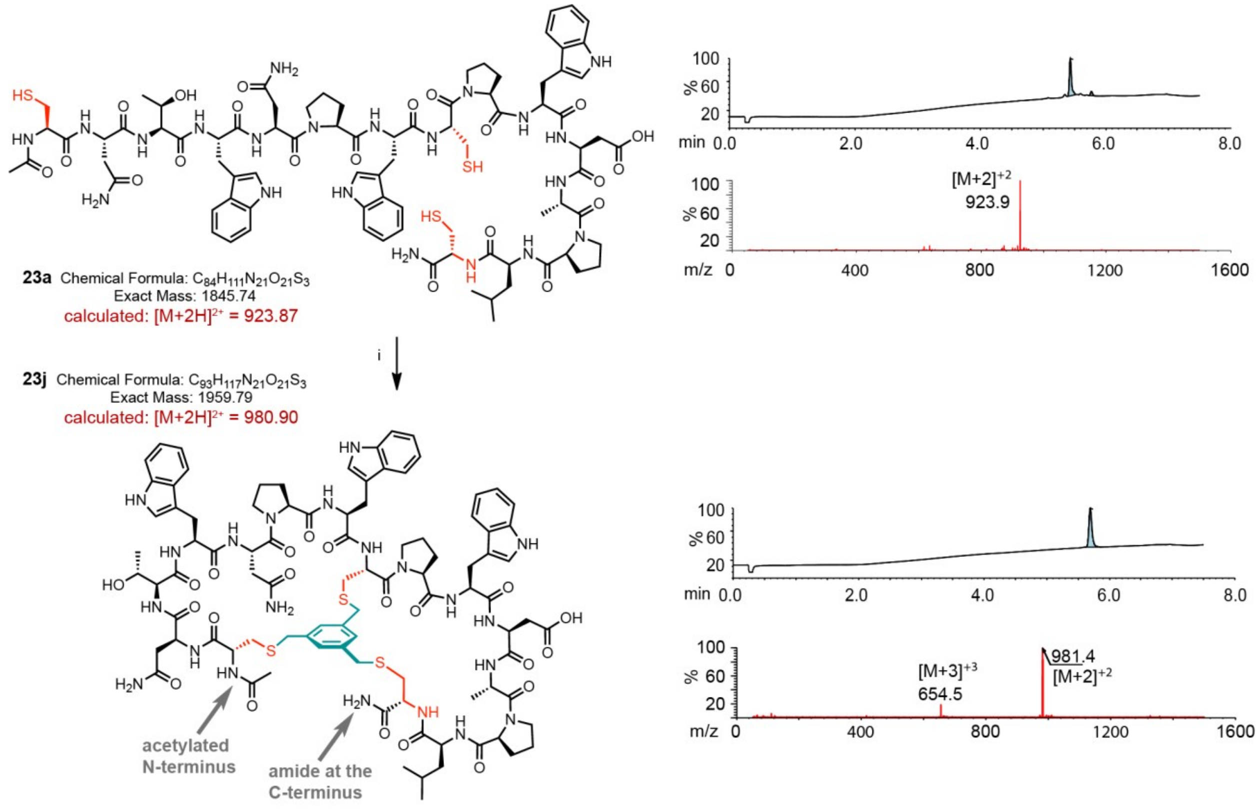

Scheme S37: Bicyclization of **23a** with **TBMB**: Reagents and conditions: 2.5 mM TCEP, 100 mM bicarbonate buffer (pH 10), 20 h. For details, see: General Protocol for bicyclization with **TBMB**

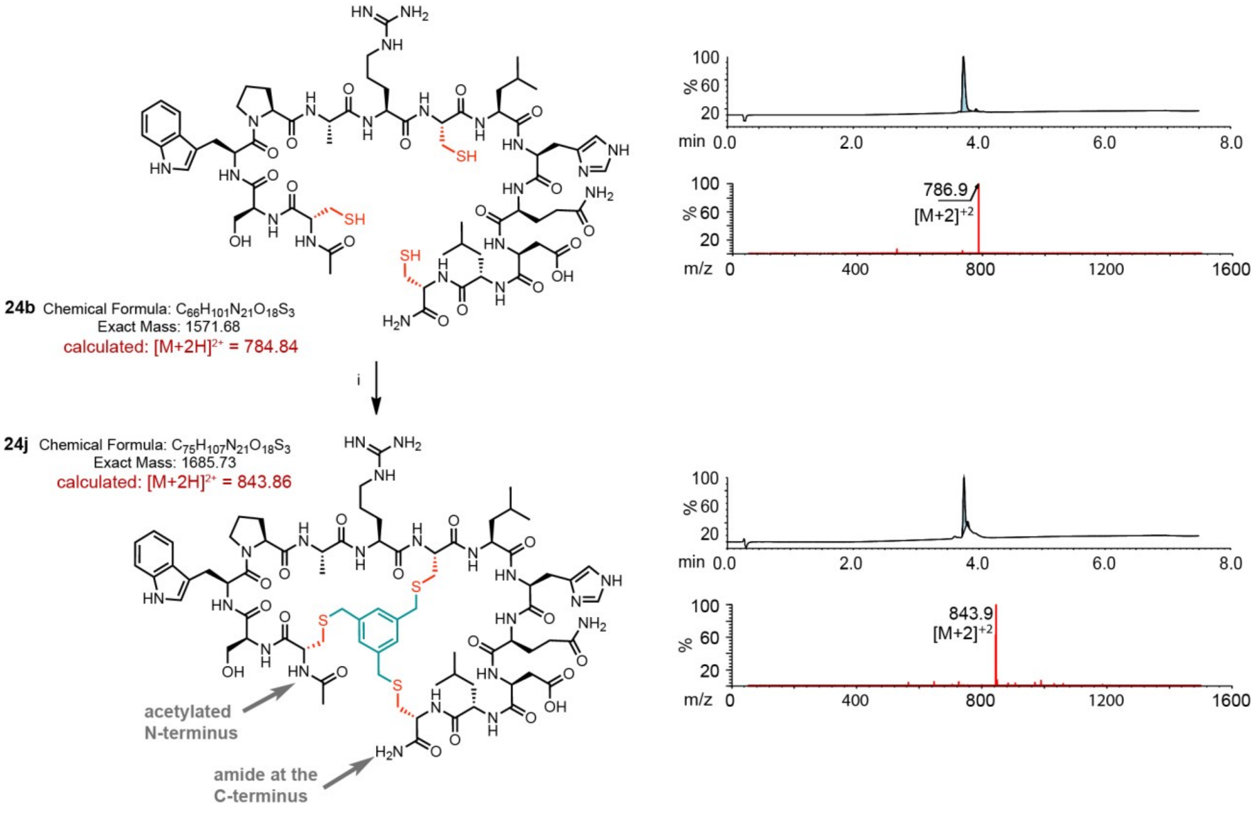

Scheme S38: Bicyclization of **24a** with **TBMB**: Reagents and conditions: 2.5 mM TCEP, 100 mM bicarbonate buffer (pH 10), 20 h. For details, see: General Protocol for bicyclization with **TBMB**

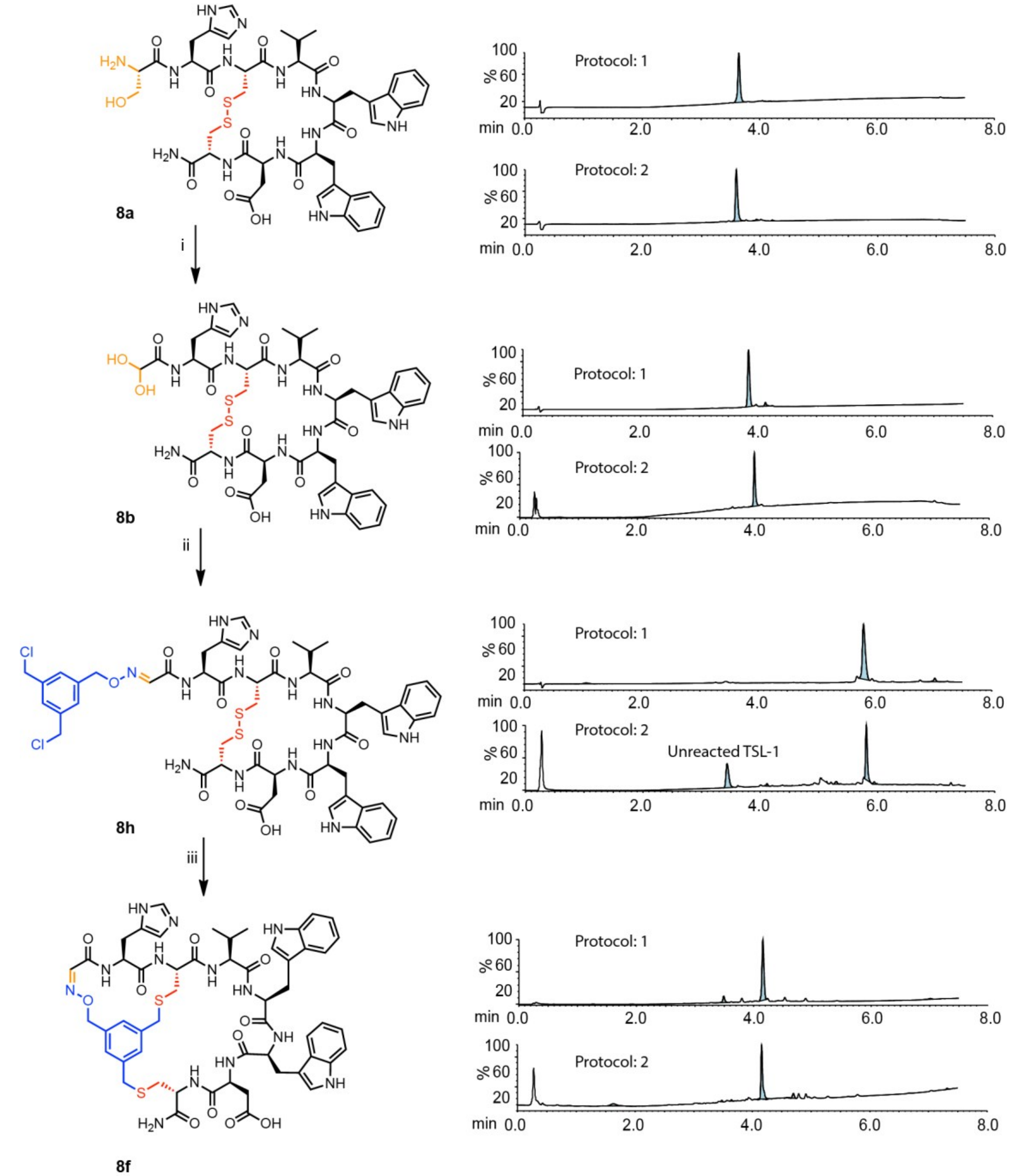

Scheme S39: Comparison between bicyclization of **8a** with **TSL-1** in two different protocols (*Protocol 1: Bicyclization of Peptides* SX_mCX_nC *with **TSL** using C18 spin column.and Protocol 2: Bicyclization of Peptides* SX_mCX_nC *with **TSL** using methionine as quencher*)

Table S1: List of peptide sequences, **TSL**s and resulting bicyclic products

S.M.	Pr.	Sequence	% yield	TSL	#. of residues	m,n	Scheme
**1a**	**1c**	H-SHCDYYC-NH2	22%	TSL-1	7	1, 3	Scheme S3
**2a**	**2c**	H-SYCKADC-NH2	37%	TSL-1	7	1, 3	Scheme S4
**3a**	**3d**	H-SYCKPFC-NH2	N.D.	TSL-3	7	1, 3	Scheme S5
**3a**	**3c**	H-SYCKPFC-NH2	41%	TSL-1	7	1, 3	Scheme S6
**4a**	**4d**	H-STCQGECGGG-NH2	47%	TSL-3	10	1, 3	Scheme S7
**5a**	**5b**	H-SICRFFCGGG-NH2	N.D.	TSL-6	10	1, 3	Scheme S2
**5a**	**5c**	H-SICRFFCGGG-NH2	N.D.	TSL-1	10	1, 3	Scheme S8
**5a**	**5d**	H-SICRFFCGGG-NH2	55%	TSL-3	10	1, 3	Scheme S9
**6a**	**6b**	H-SHDCYLEC-NH2	N.D.	TSL-6	8	2, 3	Scheme S10
**6a**	**6c**	H-SHDCYLEC-NH2	43%	TSL-1	8	2, 3	Scheme S11
**6a**	**6d**	H-SHDCYLEC-NH2	N.D.	TSL-3	8	2, 3	Scheme S12
**7a**	**7b**	H-SWDYRECYLEC-NH2	42%	TSL-6	11	5, 3	Scheme S13
**7a**	**7c**	H-SWDYRECYLEC-NH2	54%	TSL-1	8	5, 3	Scheme S14
**8a**	**8b**	H-SHCVWWDC-NH2	N.D.	TSL-6	8	1, 4	Scheme S15
**8a**	**8d**	H-SHCVWWDC-NH2	N.D.	TSL-3	8	1, 4	Scheme S16
**8a**	**8c**	H-SHCVWWDC-NH2	48%	TSL-1	8	1, 4	Scheme S17
**9a**	**9b**	H-SFCDWYGC-NH2	20%	TSL-6	8	1, 4	Scheme S18
**10a**	**10b**	H-SYCPYSGTNC-NH2	32%	TSL-6	10	1, 6	Scheme S19
**11a**	**11b**	H-SLCFSQHHDC-NH2	28%	TSL-6	10	1, 6	Scheme S20
**12a**	**12c**	H-SSWPARCLHQDLC-NH2	29%	TSL-1	13	5, 5	Scheme S21
**13a**	**13c**	H-SNTWNPWCPWDAPL-cam	41%	TSL-1	14	6, 5	Scheme S22
**14a**	**14b**	H-SPCKAGTGQC-NH2	30%	TSL-6	10	1, 6	Scheme S23
**15a**	**15b**	H-SPCKGPSATC-NH2	9%	TSL-6	10	1, 6	Scheme S24
**16a**	**16b**	H-SPCKGRHHNC-NH2	51%	TSL-6	10	1, 6	Scheme S25
**17a**	**17b**	H-SPCKKAHGAC-NH2	9%	TSL-6	10	1, 6	Scheme S26
**18a**	**18b**	H-SPCQRGHMFC-NH2	8.6%	TSL-6	10	1, 6	Scheme S27
**19a**	**19b**	H-SYCKRAHKNC-NH2	14%	TSL-6	10	1, 6	Scheme S28
**20a**	**20b**	H-SQCKRAHAEC-NH2	31%	TSL-6	10	1, 6	Scheme S29
**21a**	**21b**	H-SWCRGHDRTC-NH2	6%	TSL-6	10	1, 6	Scheme S30
**22a**	**22b**	H-SPCAKGMNYC-NH2	5.9%	TSL-6	10	1, 6	Scheme S31
**23a**	**23b**	H-SWCDYRC-NH2	N.D.	TSL-6	7	1, 3	Scheme S32
**24a**	**24c**	H-SWCFYRC-NH2	N.D.	TSL-1	7	1, 3	Scheme S33
**25a**	**25b**	H-SLCFDNGC-NH2	N.D.	TSL-6	8	1, 3	Scheme S34
**26a**	**26d**	H-SDCGFVSC-NH2	N.D.	TSL-3	8	1, 4	Scheme S35

(N.D) = Not determined or reaction were carried out in analytical scale

Table S2: Peptide sequence used in the study and their properties.

	Sequence (SX_mCX_nC)	#. of residue	m, n	Charge (pH 7)	GRAVY Hydro-phobicity[2]	Boman Index[2]	2nd structure prediction[3-5]
**1a**	H-SHCDYYC-NH_2	7	1, 3	-0.75	-0.72	2.07	loop
**2a**	H-SYCKADC-NH_2	7	1, 3	0	-0.39	1.92	loop
**3a**	H-SYCKPFC-NH_2	7	1, 3	1	0.03	0.5	loop
**4a**	H-STCQGECGGG-NH_2	10	1, 3	-1	-0.51	2.11	loop
**5a**	H-SICRFFCGGG-NH_2	10	1, 3	1	0.86	0.69	α-helix
**6a**	H-SHDCYLEC-NH_2	8	2, 3	-1.75	-0.43	2.03	loop
**7a**	H-SWDYRECYLEC-NH_2	11	5, 3	-2	-0.95	2.83	α-helix
**8a**	H-SHCVWWDC-NH_2	8	1, 4	-0.75	-0.01	0.69	Loop
**9a**	H-SFCDWYGC-NH_2	8	1, 4	-1	0.11	0.43	loop
**10a**	H-SYCPYSGTNC-NH_2	10	1,6	0	-0.54	1.27	β-sheet
**11a**	H-SLCFSQHHDC-NH_2	10	1,6	-0.5	-0.34	1.99	α-helix
**12a**	H-SSWPARCLHQDLC-NH_2	13	5, 5	1	-0.341	1.85	α-helix
**13a**	H-SNTWNPWCPWDAPL-*cam*	15	6, 5	-1	-0.81	0.92	loop
**14a**	H-SPCKAGTGQC-NH_2	10	1, 6	1	-0.45	1.08	loop
**15a**	H-SPCKGPSATC-NH_2	10	1, 6	1	-0.3	0.96	loop
**16a**	H-SPCKGRHHNC-NH_2	10	1, 6	2.5	-1.61	3.63	loop
**17a**	H-SPCKKAHGAC-NH_2	10	1, 6	2.25	-0.52	1.2	α-helix
**18a**	H-SPCQRGHMFC-NH_2	10	1, 6	1.25	-0.43	1.96	loop
**19a**	H-SYCKRAHKNC-NH_2	10	1, 6	3.25	-1.43	3.64	α-helix
**20a**	H-SQCKRAHAEC-NH_2	10	1, 6	1.25	-1.08	3.47	α-helix
**21a**	H-SWCRGHDRTC-NH_2	10	1, 6	1.25	-1.35	4.33	loop
**22a**	H-SPCAKGMNYC-NH_2	10	1, 6	1	-0.28	0.8	α-helix
**23a**	Ac-CNTWNPWCPWDAPLC-NH_2	15	6, 5	0.25	-0.37	0.46	loop
**24a**	Ac-CSWPARCLHQDLC-NH_2	13	5, 5	0.25	-0.08	1.49	α-helix
**25a**	H-SWCDYRC-NH_2	7	1, 3	0	-0.85	3.18	α-helix
**26a**	H-SWCFYRC-NH_2	7	1, 3	1	0.04	1.51	α-helix
**27a**	H-SLCFDNGC-NH_2	8	1, 4	-1	0.42	0.92	loop
**28a**	H-SDCGFVSC-NH_2	8	1, 4	-1	0.81	0.62	loop

Table S3: Modifiers other than **TSL** and resulting bicyclic/monocyclic product

S.M.	Pr.	Sequence	% yield	Linker	#. of residues	m,n	Ref.
**4a**	**4e**	H-STCQGECGGG-NH2	31%	PFS	10	1, 3	Scheme S36
**5a**	**5e**	H-SICRFFCGGG-NH2	40%	PFS	10	1, 3	Scheme S36
**23a**	**23f**	Ac-CNTWNPWCPWDAPL*Cam*-NH2	38%	TBMB	14	6, 5	Scheme S37
**24a**	**24f**	Ac-CSWPARCLHQDLC-NH2	33%	TBMB	13	5, 5	Scheme S38
**1a**	**1g**	H-SHCDYYC-NH2	47%	DBMB	7	1, 3	Figure S50
**2a**	**2g**	H-SYCKADC-NH2	47%	DBMB	7	1, 3	Figure S51
**3a**	**3g**	H-SYCKPFC-NH2	62%	DBMB	7	1, 3	Figure S52
**4a**	**4g**	H-STCQGECGGG-NH2	19%	DBMB	7	1, 3	Figure S53
**5a**	**5g**	H-SICRFFCGGG-NH2	46%	DBMB	7	1, 3	Figure S54
**6a**	**6g**	H-SHDCYLEC-NH2	27%	DBMB	8	2, 3	Figure S55
**7a**	**7g**	H-SWDYRECYLEC-NH2	12%	DBMB	11	5, 3	Figure S56
**8a**	**8g**	H-SHCVWWDC-NH2	16%	DBMB	8	1, 4	Figure S57
**9a**	**9g**	H-SFCDWYGC-NH2	10%	DBMB	8	1, 4	Figure S58
**12a**	**12g**	H-SSWPARCLHQDLC-NH2	46%	DBMB	14	6, 5	Figure S59
**14a**	**14g**	H-SPCKAGTGQC-NH2	12%	DBMB	10	1, 6	Figure S60
**15a**	**15g**	H-SPCKGPSATC-NH2	10%	DBMB	10	1, 6	Figure S61
**16a**	**16g**	H-SPCKGRHHNC-NH2	63%	DBMB	10	1, 6	Figure S62
**19a**	**19g**	H-SYCKRAHKNC-NH2	32%	DBMB	10	1, 6	Figure S63
**20a**	**20g**	H-SQCKRAHAEC-NH2	10%	DBMB	10	1, 6	Figure S64
**22a**	**22g**	H-SPCAKGMNYC-NH2	25%	DBMB	10	1, 6	Figure S65

(N.D) = Not determined or reaction were carried out in analytical scale

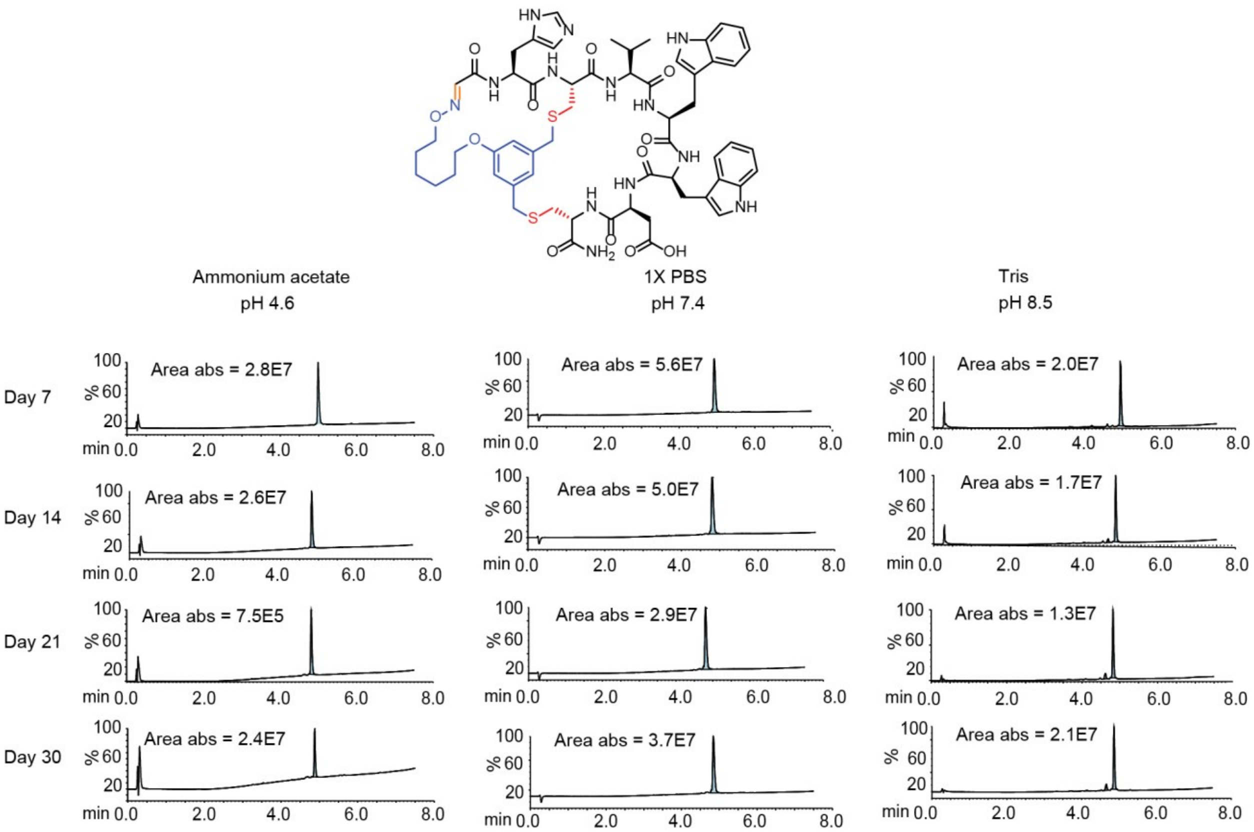

Figure S1: Stability test of bicyclic peptide **TSL-6-**SHCDYYC over 30 days in buffers of different pH

2. Phage Modification Methods

2.1. *Preparation of SXCX$_6$C phage libraries*
The procedures have been adopted and modified from previously described in two publications that produced the M13-displayed SXCXXXC library[6] and M13-SDB vector[7]. In short, the vector SB4 QFT*LHQ was digested with Kpn I HF (NEB cat# R3142S) and Eag I HF (NEB cat# R3505S). A primer/template pair consisting of primer 5' - CAT GGC GCC CGG CCG AAC CTC CAC C - 3' and template 5' CC CGG GTA CCT TTC TAT TCT CAC TCT TCT X TGT XXXXXX TGT GGT GGA GGT TCG GCC GGG CGC TTG ATT - 3' with the 'X' representing a trinucleotide was formed by annealing. The primer/template was then extended using Klenow DNA polymerase (NEB) according to the manufacturer's instructions. The insert fragment was then digested with Kpn1 HF and Eag1 HF, gel purified and ligated into the cut vector. The ligation products were then transformed into electrocompetent *E.coli* cells and the transformants were grown overnight on *E.coli* TG1 to allow for phage production. Phage cultures were then centrifuged to remove cells and debris and then the phage was precipitated by PEG precipitation (5% PEG 0.5 M NaCl). Other SD vectors have been processed identically. We sequenced the naïve libraries by Illumina sequencing and the naïve library of SB4-SXCXXXXXXC composition are publicly available at the following links: https://48hd.cloud/file/1470

2.2. *General protocol for modification of SXCX$_6$C phage library:*
SXCX$_6$C phage-displayed peptide library was cloned using trinucleotide codon libraries and purified by PEG precipitation as described in **2.1**. We observed that the further cleanup of phage-associate lipopolysaccharide (LPS) improved the chemical modification. To remove the LPS, the phage solution (10^{13} PFU/mL) was combined with Triton X-100 to 10% final amount and incubated for 1 hour at room temperature. The phage was then re-purified using PEG-NaCl precipitation and resuspended to original volume with PBS (50 mM, pH 7.4). The resuspended phage then dialyzed at 4 ºC against 4 L of PBS (50 mM, pH 7.4) for 12 hours using 10K MWCO membrane. All the incubation in the chemical modification were performed by gentle agitation with a rotator, as prolonged vortex-shaking of phage is detrimental to infectivity of phage.[8]

Oxime Ligation: To a cleaned phage library (100 µL, ~3×10^{13} pfu/mL), we added sodium periodate (1 µL of 6 mM $NaIO_4$ in water to a final concentration of 60 µM) and incubated on ice in the dark for 9 min. The oxidation was quenched with methionine (1 µL of 500 mM methionine in water to a final concentration of 0.5 mM) and incubated for 20 minutes at rt. To the oxidized library, we added **TSL-6** linchpin (100 µL of 2 mM **TSL-6** in 20% aq. CH_3CN containing 0.2% TFA; final concentrations: 2 mM of **TSL-6**, 10% CH_3CN, 0.1% TFA) and incubated for 1 h at rt. To monitor the oxidation and oxime ligation reactions, we used previously described biotin capture assay.[9] Briefly, 5 µL of the oxidized or 5 µL of the oxime-ligated phage solutions were combined with 1 mM (5 µL of 2 mM AOB in 200 mM anilinum acetate buffer, pH 4.6) for 1 h. AOB modified phage was diluted 10^6 fold, captured with streptavidin magnetic beads; supernatant was tittered before and after capture.

Reduction and bicyclization: The **TSL**-ligated library was purified using Zeba™ Spin Desalting Columns (7K MWCO, 0.5 mL, cat# 89882) using sodium acetate (50 mM NaAc, pH 5) as eluent. To 100 µL of the purified library, we added TCEP (2 µL of 50 mM TECP in water, final

concentration 0.5 mM) and incubated for 30 mins. Increase of the pH to 10 by addition of bicarbonate buffer (25 μL of 1 M bicarbonate buffer, pH 10) and incubation for 3 h led to cyclization. The modified library supplemented with PBS (20 μL of 500 mM PBS, pH 7.4) and purified using Zeba column prior to storage or panning. To monitor the cyclization reaction, 5 μL of the reaction mixture was sampled at various steps (before and after addition of TCEP, control experiments with TCEP) and combined thiol-biotin (BSH) at pH 8.5 (2 μL of 4 mM BSH in MiliQ water), supplemented with 5 μL of 500 mM Tris-HCl pH 8.5 and 38 μL water and incubated for 3 hours. The phage treated with BSH was captured with biotin-capture assay as described above. Typically, over 40% of the phage library was successfully bi-cyclized.

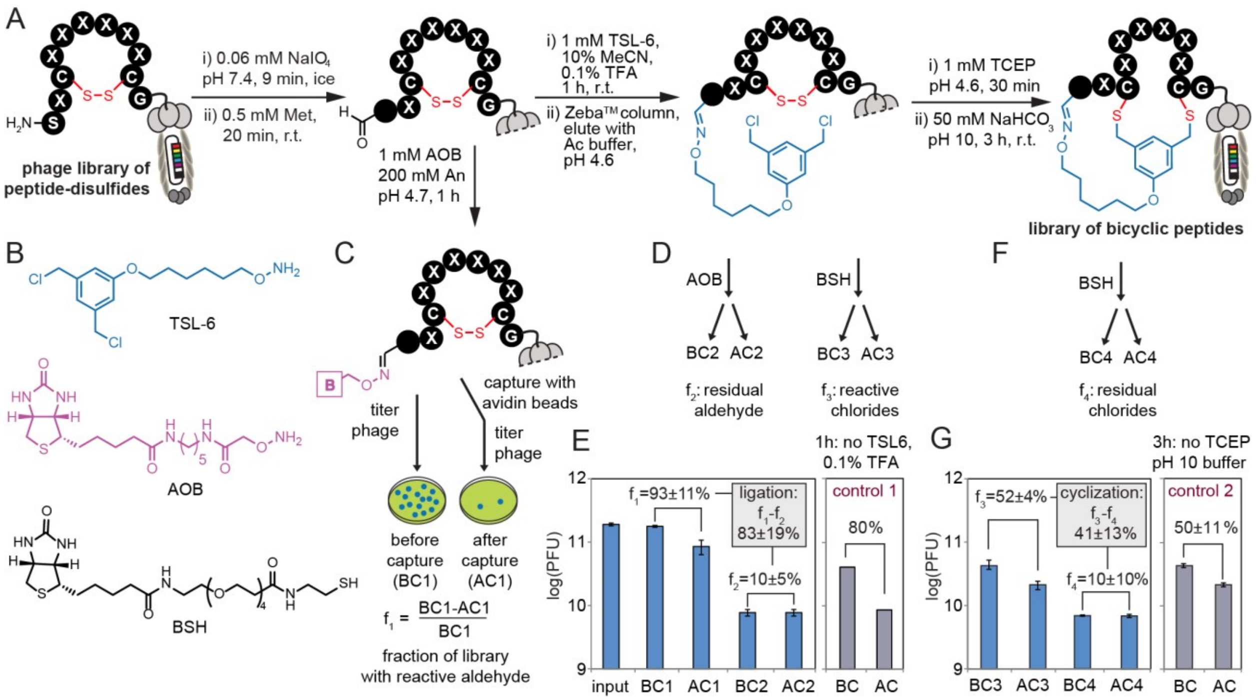

Figure S2: Modification of the library of 10^8 peptides displayed on phage by the **TSL-6.** (A) Scheme of the modification. (B) Reagents for synthesis or monitoring of the reactions. (C-D) Exposure of the aldehyde-peptide library to AOB and counting the number of phage particles before and after the capture with streptavidin-agarose measured the fraction of library with aldehyde. (E) Reaction of aldehyde-phage with **TSL-6** decreased the fraction of library with aldehyde from 73±11 to 10±5%. Control incubation in TFA in the absence of **TSL-6** did not decrease the fraction of aldehydes. (F) Ligation of **TSL-6** introduced thiol-reactive chlorobenzyl groups on phage that were detected by BSH. (G) Reduction with TCEP at pH 4.6 and increase of the pH to 10 induced bicyclization and decreased the fraction of library with thiol reactive groups from 52±4 to 10±10%. Control incubation of **TSL-6**-modified phage in pH 10 buffer for 3 h in the absence of TCEP did not lead to a significant decrease of thiol-reactive groups: phage remained reactive to BSH (E-F)

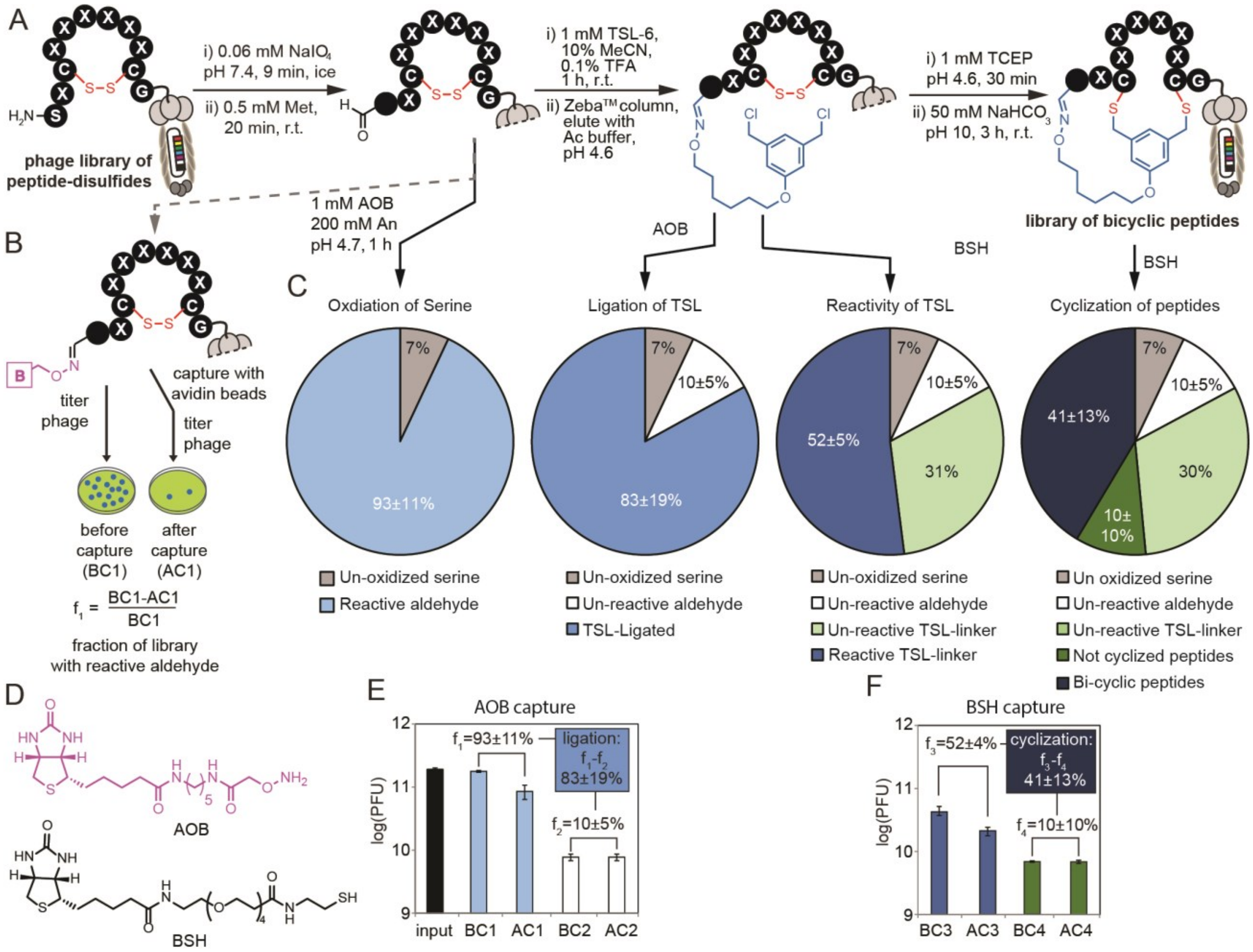

Figure S3: Composition of SXCX$_6$C library during modification with **TSL-6** (A) Overall step-by-step modification of SXCX$_6$C displyed peptide library by **TSL-6**. (B) The efficiency of oxidation was measured by exposure of the phage to aminooxybiotin (AOB) and measuring the biotinylation by counting the number of phage particles before and after the capture of the modified phage with streptavidin paramagnetic particles. (C) The percentage of different chemical species in the different steps of the modification of SXCX$_6$C displyed peptide library. (D) Thiol and aldehyde reactive compound for generating bicyclic phage (**TSL-6**). Biotinylating compounds to monitor reaction progress for oxime ligation (AOB) and cyclization (BSH). (E-F)

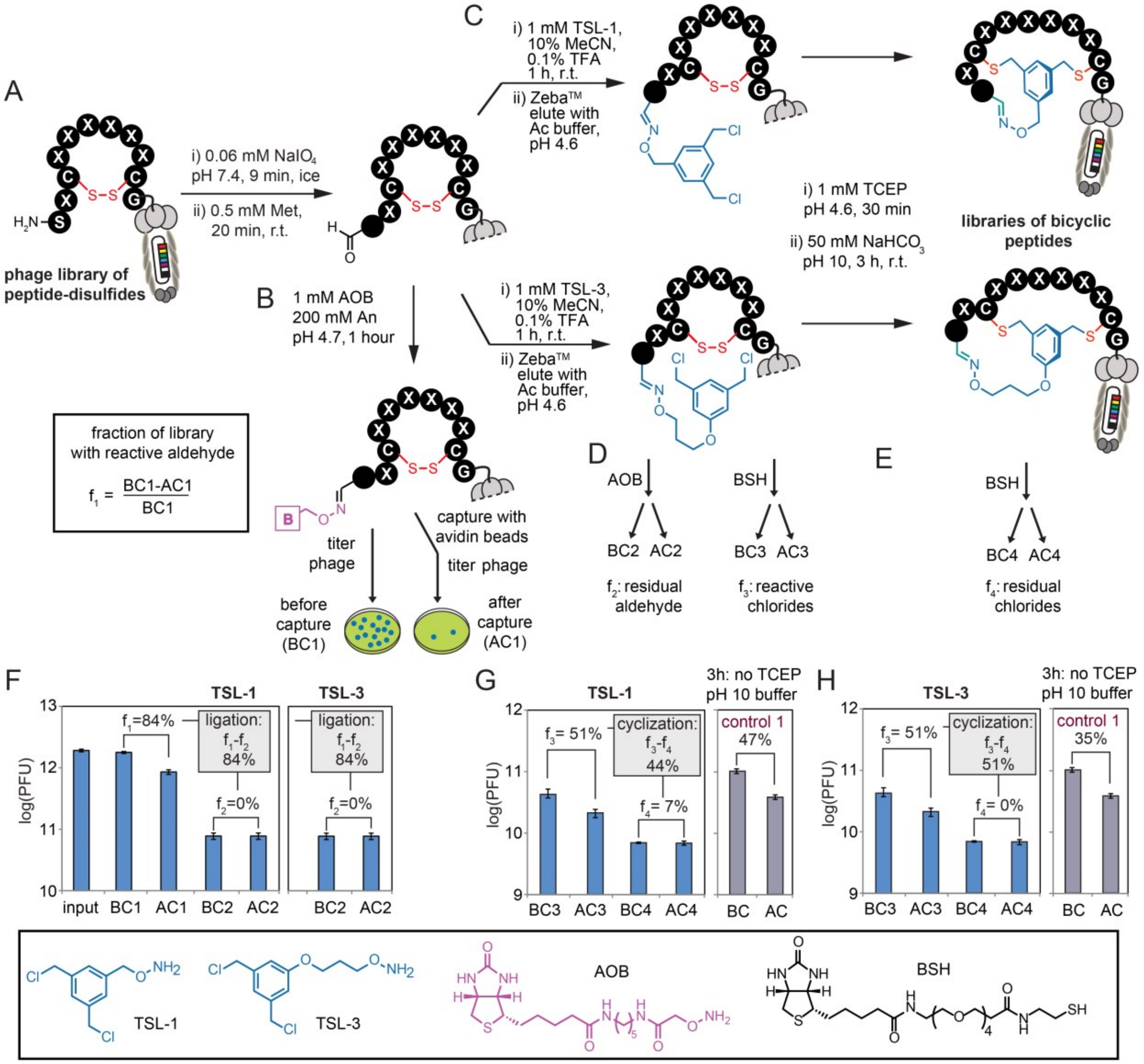

Figure S4: Modification of the $SXCX_6C$ library by the **TSL-1** and **TSL-3**. (A) The process starts from oxidation of N-Ser by $NaIO_4$. (B) The efficiency of oxidation was measured by exposure of the library to aminooxybiotin (AOB) and measuring the biotinylation by counting the number of phage particles before and after the capture of the library with streptavidin paramagnetic particles. (C) Two aliquots of aldehyde library were exposed to **TSL-1** or **3** in 0.1% TFA for 1 h. After purification by Zeba^{TM} column and elution with acetate buffer (pH 4.6), exposure to TCEP at pH 4.6 for 30 min reduced the disulfides and increase of the pH to 10 induced bicyclization. (D) "AOB-capture" after ligation of **TSL** detects disappearance of aldehydes; similar "BSH-capture" detects concurrent appearance of thiol-reactive chlorobenzyl groups and (E) their disappearance after bicyclization. (F) "AOB capture" shows that 84% of library was oxidized, and **TSL-1** or **3** consumed all aldehyde groups. (G-H) BSH-capture confirms appearance thiol-reactive groups on phage and their disappearance after bicyclization. In control conditions, incubation of **TSL-1** or **3** ligated phage in pH 10 buffer for 3 h in the absence of TCEP did not lead to a significant decrease of chlorobenzyl groups: phage remained reactive to BSH.

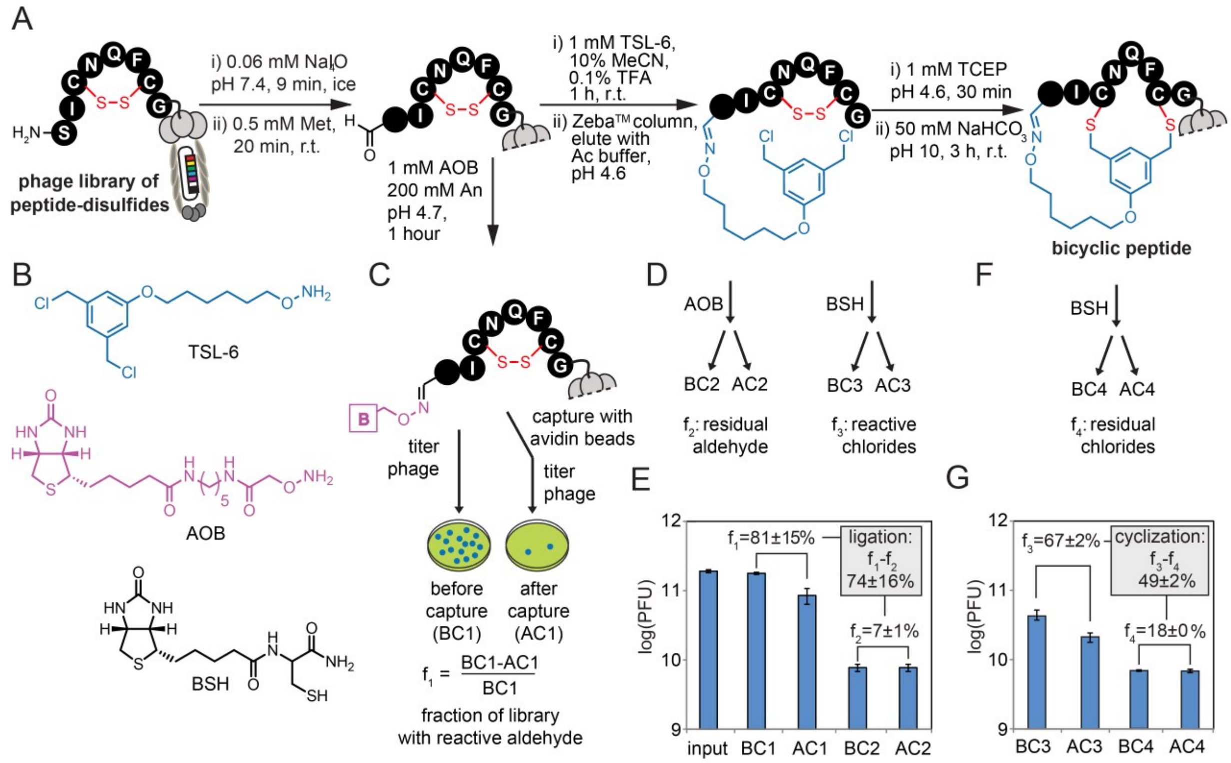

Figure S5: Modification of monoclonal phage displaying SICNQFC with **TSL-6**.. (A) Overall step-by-step modification of the peptide, SICNQFC displayed on M13KE phage by the linchpin **TSL-6**. (B) Thiol and aldehyde reactive compound for generating bicyclic phage (**TSL-6**). Biotinylating compounds to monitor reaction progress for oxime ligation (AOB) and cyclization (BSH). (C) The efficiency of oxidation was measured by exposure of the phage to aminooxybiotin (AOB) and measuring the biotinylation by counting the number of phage particles before and after the capture of the modified phage with streptavidin paramagnetic particles. (D-E) Reaction with **TSL-6** in 0.1% TFA for 1 hour led to the disappearance of aldehyde functionality and loss of biotinylation after exposure to AOB and concurrent appearance of thiol-reactive chlorobenzyl groups: their presence was detected by exposure of phage to biotin-thiol (BSH). After purification by size-exclusion Zeba™ column, to remove excess of the linchpin **TSL-6**, and elution with acetate buffer (pH 4.6), exposure to TCEP at pH 4.6 for 30 minutes for reducing the disulfides. The increase of the pH to 10 induced bicyclization. Exposure of the bicyclized product to BSH did not produce visible biotinylating, indicating the disappearance of reactive thiol groups

3. General Selection and Validation Methods

3.1. *General setting for panning on Kingfisher instrument*

The protein immobilized beads suspension and other reagents were added to a 96 Deepwell Plate (Thermo Fisher, #95040450) as follows:

Row A: Protein coated magnetic beads (1mL in in PBS Buffer)
Row B: Reserved for 12-tip Deepwell magnetic comb (Thermo Fisher, #97003500)
Row C: Wash Buffer (1 mL, PBS buffer)
Row D: Blocking Buffer (1 mL, 2% BSA (w/v) in PBS Buffer)
Row E: Solution of TSL-6 $SXCX_6C$ libraries (1 mL, 10^9 PFU/mL in PBS Buffer)
Row F: Wash Buffer (1 mL, 0.1 % Tween-20 (v/v) in PBS Buffer)
Row G: Wash Buffer (1 mL, 0.1 % Tween-20 (v/v) in PBS Buffer)
Row H: Wash Buffer (1 mL, 0.1 % Tween-20 (v/v) in PBS Buffer)

Following steps were performed using a KingFisher^{TM} Duo Prime Purification System with a magnetic comb to transfer the beads. The program is as follows: a) collect comb from row B b) Collect beads from row A on comb, c) Wash beads in row C – 30 s, d) Block in row D – 1 h, e) Phage binding in row E – 1.5 h, f) Wash beads in row F – 1 min, g) Wash beads in row G – 1 min, h) Wash beads in row H – 1 min. At the end of the program, the protein coated beads with phage bound were in wells in the Row H. The content of each well from row H was transferred to individual Eppendorf^{TM} tube, and process for next round panning described in **3.2** and for Illumina deep sequencing described in **3.3**.

3.2. *Bio panning of NODAL protein*

All his-tagged NODAL protein were purchased from Proteintech (cat # Ag21882) and Ni-NTA magnetic beads were purchased from Thermo Fisher Scientific (cat # 10104D)

<u>First round of selection:</u> (Denoted as R1-NT) In a 1.7 mL centrifuge tube, 20 µL of Ni-NTA magnetic beads were incubated with 5 µg of His-tagged NODAL overnight in 100 µL of 1× PBS at 4 °C. In parallel, **TSL-6** modified library was incubated with 20 µL of empty Ni-NTA magnetic beads over at 4 °C to remove beads specific binding. After immobilizing, the beads were wash with 1×PBS 3 times and blocked with blocking solution (1 % BSA in 1× PBS) at rt for 1 hour. In parallel, **TSL-6** modified library was incubated with 20 µL of empty Ni-NTA magnetic beads in the present of blocking solution (1 % BSA in 1 × PBS) at rt for 1 hour. After blocking the NODAL immobilized beads, pre-selected **TSL-6** modified library was incubated with NODAL immobilized beads for 2 hours at rt. The beads were captured with magnetic rack and washed once with 1×PBS with 0.1%(v/v) Tween-20 to remove unbound phage. Phage remaining on the beads were eluted with 200 µL of glycine elution buffer (Glycine-HCl pH 2.2, 0.1% BSA) for 9 min. The elution buffer was transferred into a new 1.7 mL microcentrifuge tube and neutralized with 20 µL of 1 M Tris-HCl (pH 9.1). The recovered phage solution was amplified for next round of bio panning and for deep sequencing.

<u>Second round of selection:</u>(R2-NT) Amplified phage recovered from R1-NT was modified with **TSL-6**. In a 1.7 mL centrifuge tube, 20 µL of Ni-NTA magnetic beads were incubated with 5 µg of His-tagged NODAL overnight in 100 µL of 1× PBS at 4 °C. In parallel, **TSL-6** modified library was incubated with 20 µL of empty Ni-NTA magnetic beads over at 4 °C to remove beads-specific binders. The blocking, panning and washing were performed in Kingfisher Instrument described in **3.1**. The panning solution after Kingfisher Instrument were transfer into 1.7 mL centrifuge tube.

Phage remaining on the beads were eluted with 200 µL of glycine elution buffer (Glycine-HCl pH 2.2, 0.1% BSA) for 9 min. The elution buffer was transferred into a new 1.7 mL microcentrifuge tube and neutralized with 20 µL of 1 M Tris-HCl (pH 9.1). The recovered phage solution was amplified for next round of bio panning and was amplified with PCR for Illumina deep sequencing.

<u>Third round of selection:</u> Amplified phage recovered from R2-NT was modified with **TSL-6**. In each 1.7 mL centrifuge tube, 20 µL of Ni-NTA magnetic beads were incubated with 2.5 µg of His-tagged NODAL and His-tagged T4-GP overnight in 100 µL of 1×PBS at 4 °C. In parallel, of second round selected **TSL-6** modified library and second round selected un-modified library was incubated with 20 µL of empty Ni-NTA magnetic beads over at 4 °C to remove beads specific binding. The panning against NODAL were performed in Kingfisher Instrument described in **3.1**. (R3-NT) In control panning, **TSL-6** library against T4-GP (R3-TG) and unmodified library that amplified phage from R2-NT against NODAL (R3-UN) were also performed in parallel. The proteins immobilized beads, phage library, blocking buffer and washing buffer were added into King Fisher Plate in the corresponding well. The panning solution after Kingfisher Instrument were transfer into 1.7 mL centrifuge tube. Phage remaining on the beads were eluted with 200 µL of glycine elution buffer (Glycine-HCl pH 2.2, 0.1% BSA) for 9 min. The elution buffer was transferred into a new 1.7 mL microcentrifuge tube and neutralized with 20 µL of 1 M Tris-HCl (pH 9.1). The recovered phage solution was amplified for next round of bio panning and was amplified with PCR for deep sequencing.

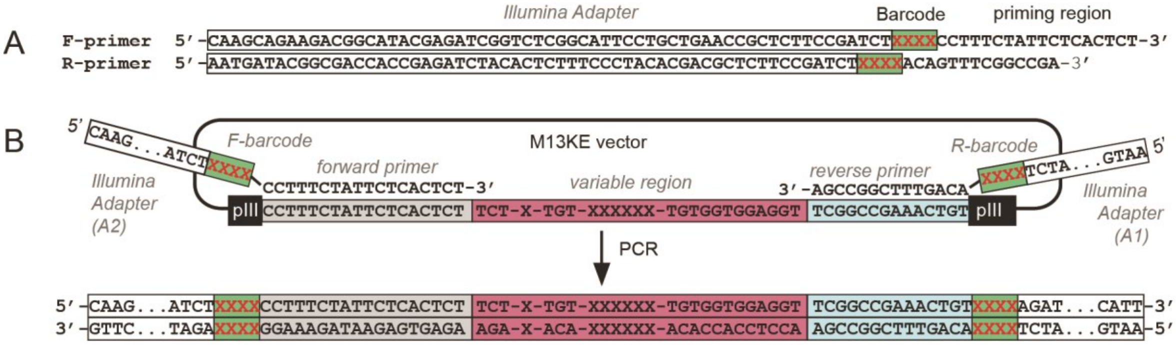

Figure S6: DNA sequences of PCR amplification protocol for Illumina deep sequencing (A) Primers used for amplifying ligated or naïve oligonucleotide DNA. XXXX denotes 4-nucleotide-long barcodes used to trace multiple samples in an Illumina sequencing experiment. (B) Generation of PCR product. Alignment of forward and reverse primers to 18-bp and 14-bp sequences flanking the variable region at the N-terminus of the pIII gene in M13KE vector, respectively.

3.3. *General PCR amplification protocol for Illumina deep sequencing*

Take 25 µL of eluted or amplified phage solution was used as a template for PCR with total volume of 50 µL. (Figure S6:)

A Typical 50 µL reaction mixture contained:

1. 5x Phusion buffer 10 µL
2. 10 mM dNTPs 1 µL
3. Phusion® High-Fidelity DNA Polymerase (NEB, cat#M0530S) 0.5 µL
4. Forward primer (3'-CAAGCAGAAGACGGCATACGAGATCGGTCTCGGCATTCCTGCTGAACCGCTCTTCCGATCTXXXXCCTTTCTATTCTCACTCT-5', 10 µM) 2.5 µL
5. Reverse primer (3'-AATGATACGGCGACCACCGAGATCTACACTCTTTCCCTACACGACGCTCTTCCGATCTXXXXACAGTTTCGGCCGA-5', 10 µM) 2.5 µL
6. Template solution 25 µL
7. Nuclease free water 8.5 µL

Thermocycler was preformed using the following setting:

a) 95 °C for 30 sec
b) 95 °C for 30 sec
c) 60.5 °C for 15 sec
d) 72 °C for 30 sec
e) Repeat step b) to d) 25 times
f) 72 °C for 5 min
g) hold at 4°C

3.4. *Illumina sequencing of samples before and after panning*

The PCR products were produced by PCR as described in Section **3.3** with one exception: in amplification of libraries before panning (input), volume of template (phage solution) was 2 µL. All products were quantified by 2% (w/v) agarose gel in Tris-Borate-EDTA buffer at 100 volts for ~35 min using a low molecular weight DNA ladder as standard (NEB, #N3233S). PCR products that contain different indexing barcodes were pooled allowing 10 ng of each product in the mixture. The mixture was purified by eGel, quantified by quBit and sequenced using the Illumina NextSeq paired-end 500/550 High Output Kit v2.5 (2x75 Cycles). Data was automatically uploaded to BaseSpace™ Sequence Hub. Processing of the data is described in section "**3.6** Processing of Illumina data".

3.5. *General data processing methods*

Data analysis of Illumina data at Figure S11 was performed in Microsoft Excel. All the 20 × 20 plots were generated on the 48 Hour Discovery cloud: https://48hd.cloud/. Linear regression analysis of Figure S13 and S14 were performed in Studio R.

3.6. *Processing of Illumina data*

The Gzip compressed FASTQ files were downloaded from BaseSpace™ Sequence Hub. The files were converted into tables of DNA sequences and their counts per experiment. Briefly, FASTQ files were parsed based on unique multiplexing barcodes within the reads discarding any reads that contained a low-quality score. Mapping the forward (F) and reverse (F) barcoding regions, mapping of F and R priming regions allowing no more than one base substitution each and F-R read alignment allowing no mismatches between F and R reads yielded DNA sequences located between the priming regions. The files with DNA reads, raw counts, and mapped peptide modifications were uploaded to http://48hd.cloud/ server. Each experiment has a unique alphanumeric name (e.g., 20181108-16TSooPA-YW) and unique static URL:

	R1	R2	R3
Un-Modified	http://48hd.cloud/file/2363	https://48hd.cloud/file/2326	https://48hd.cloud/file/2600
TSL-6 Modified	https://48hd.cloud/file/2320	https://48hd.cloud/file/2602	https://48hd.cloud/file/2609
Elution	https://48hd.cloud/file/2322	https://48hd.cloud/file/2601	https://48hd.cloud/file/2608
Amplification	https://48hd.cloud/file/2326	https://48hd.cloud/file/2600	https://48hd.cloud/file/2607

3.7. *General protocol for protein extraction*

All samples in the protein extraction protocol were done on ice. All cell samples were scrapped and treated with M-PER™ Mammalian Protein Extraction Reagent (Thermo Scientific, Cat. # 78501). Then, the treated sample sonicated for 4 sec and centrifuging with ~15,000×g for 10 mins at 4° C to remove cell debris. The supernatant then transferred to a new tube and store at -20° C for further analysis

3.8. *Western blotting protocol for detecting pSMAD2 protein level*

All cell lysate samples were mixed with 4× Laemmli sample buffer (Biorad, Cat. # 1610747) and 5% (v/v) 2-Mercaptoethanol (Sigma-Aldrich, cat #M6250). All sample were boiled for five minutes at 95 °C. SDS-PAGE were run with 10 % Acrylamide gels with 4 % staking layer. Proteins were transfer to nitrocellulose membrane, 0.45 µm (Biorad, Cat. # 1620115) with setting of 80 V for 75 mins in 4 ºC After western blot transfer, all the membranes were blocked with 6% milk in 1×TBS with 0.1% Tween 20 in room temperature for 1 h. All membranes were incubated with primary antibodies in 1×TBS, 0.1% Tween 20 and 3% BSA at 4 °C O/N. For detecting pSMAD2, rabbit anti-smad2 (phospho S423 + S425) antibody (Cell signaling Technology, Cat. #3108) was used at the dilution of 1/1000. For detecting SMAD2/3, Anti-Smad2 + Smad3 antibody (Cell signaling Technology, Cat. #8685) was used at the dilution of 1/1000. For detecting Nodal, Human Nodal Antibody (R&D system, cat# MAB3218) was used at the dilution of 1/1000. After O/N primary antibody incubation, all membranes were washed 3 times with 1×TBS and 0.1% Tween 20 in room temperature for 5 mins. All the membranes then incubated with corresponding seconding antibody anti-Mouse or anti-Rabbit that conjugate with HRP at the dilution of 1:7000. For imaging, the membranes were treated with Clarity™ Western ECL Substrate (Biorad, Cat. # 1705060) for 1 min and then exposed to X-ray film (Fuji Super RX) accordingly.

3.9. *General protocol P19 Cell Culture*
P19 Cell were obtained from ATCC cell bank and culture in Alpha Minimum Essential Medium with ribonucleotide and deoxyribonuclease with 7.5% bovine calf serum and 2.5% fetal bovine serum at 37 °C with 5% CO_2 supplementation.

3.10. *Inhibition of pSMAD assay with P19 Cell*
P19 Cells were seeded in 6 wells plate with 200,000 cells/well and were grew in full media contain 10 µM of SB341542 to suppress pSMAD signals O/N. Then, the cells were washed with warm serum free Alpha Minimum Essential medium 3 times and were co-treated the cells with peptides at 10 µM and rhNODAL 100 ng/mL (R&D system, Cat. # 3218-ND/CF) in serum free Alpha Minimum Essential medium for 1 hour at 37 °C with 5% CO_2 supplementation**.** After 1 hour of treatment, cells washed and lysed (**3.2**. General protocol for protein extraction). All the samples were stored at -20 °C for further western blotting analysis (**3.3.** Western blotting protocol for detecting pSMAD2 protein level).

3.11. *Transfect TYK-nu cell with constitutive NODAL and GFP*
TYK-nu ovarian cancer cells were obtained from JCRB cell bank and cultured in Eagle's minimal essential medium with 10 % fetal calf serum (Gibco/Thermo Fisher; Waltham, Massachusetts, USA) at 37 °C with 5% CO_2 supplementation. To express the constitutive NODAL, a plasmid vector for human NODAL open reading frame (not including the stop codon) was cloned into pCMV6-Entry vector in frame with a tandem MYCDYK (FLAG) tag (Origene Cat. # RC211302). The pCMV6 plasmid containing a GFP insert was used as a negative control. TYK-nu cells were transfected with desired plasmids using GeneIn (GlobalStem) following the manufacturer's protocol. Cells were stably selected with G418 (Thermo Fisher) at 250 µg/mL starting 48 hours after transfection for 10 days, and then maintained at 100 µg/mL. Nodal overexpression in TYK-nu cells was confirmed by Western Blot.

3.12. *Cell Viability assay with TYK-nu-NODAL and TYK-nu-GFP*
Cells were seeded in three 96 wells plates with 600 cells/well or 6000 cells/well and grow in full media with G418 at 100 µg/mL O/N. The next day the media was changed to contain **19d** peptide (10 µM, 1 µM and 0.1 µM) and without G418. Cells viability was measured every 24 hours with CellTiter-Glo® Luminescent Cell Viability Assay (Promega Cat. # G7572) over the course of 72 h.

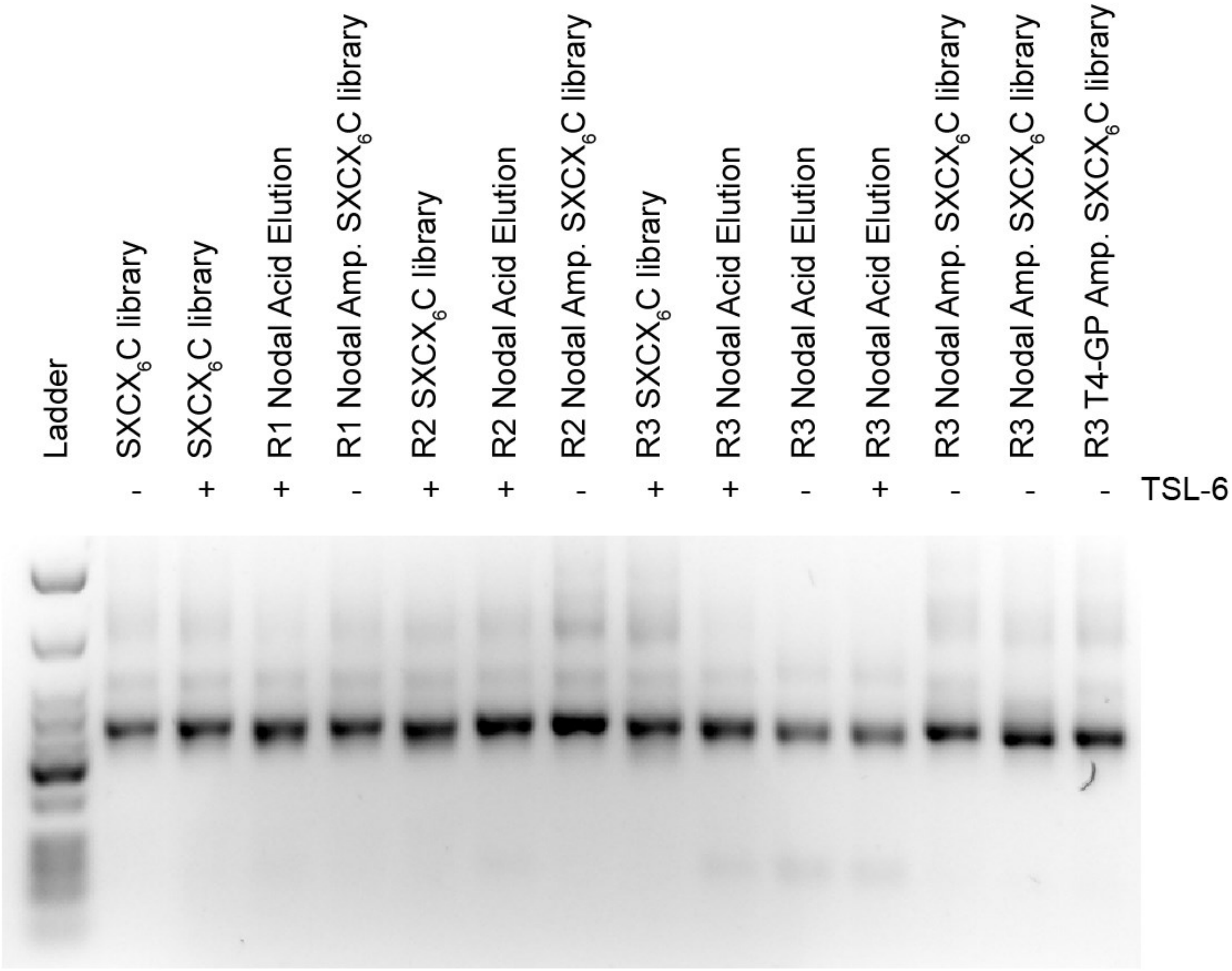

Figure S7: PCR product of **TSL-6** modification and 3 rounds of the NODAL panning.

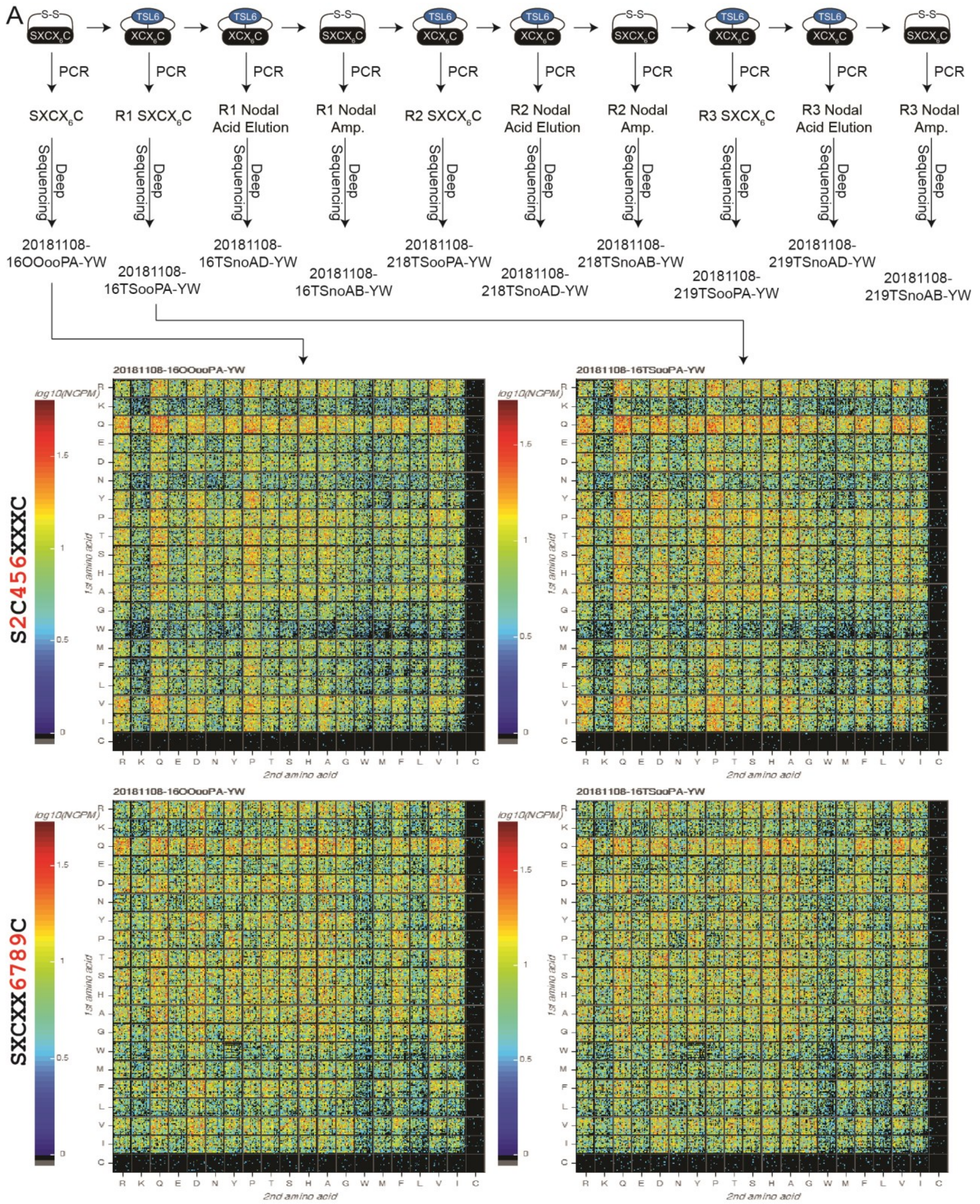

Figure S8: 20 × 20 plot comparison before and after **TSL-6** modification in input library. (20181108-16OO00PA-YW) example of names from deep sequencing files.) 20x20 plot are produce as previous publications.

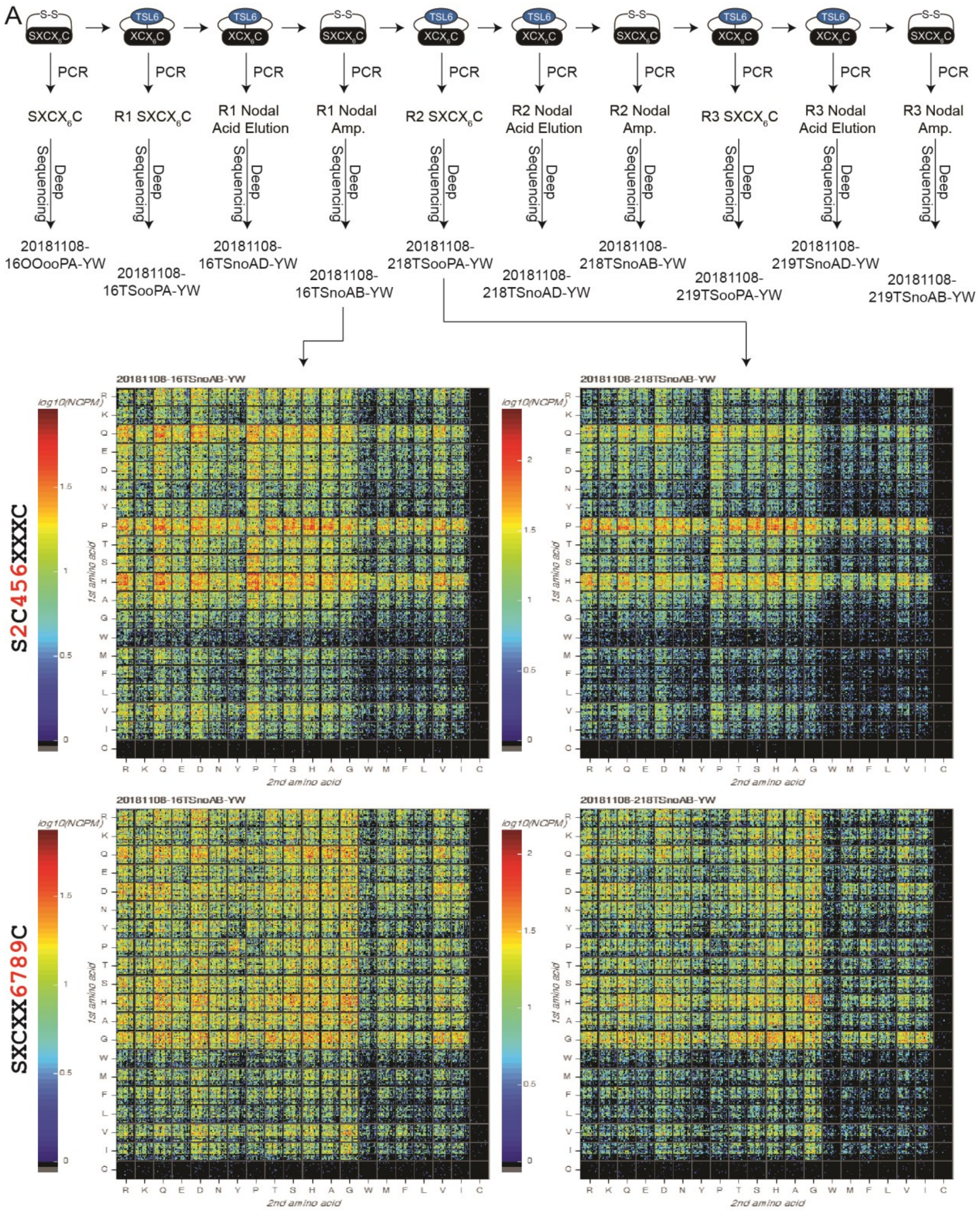

Figure S9: 20 × 20 plot comparison before and after **TSL-6** modification after R1 selection.

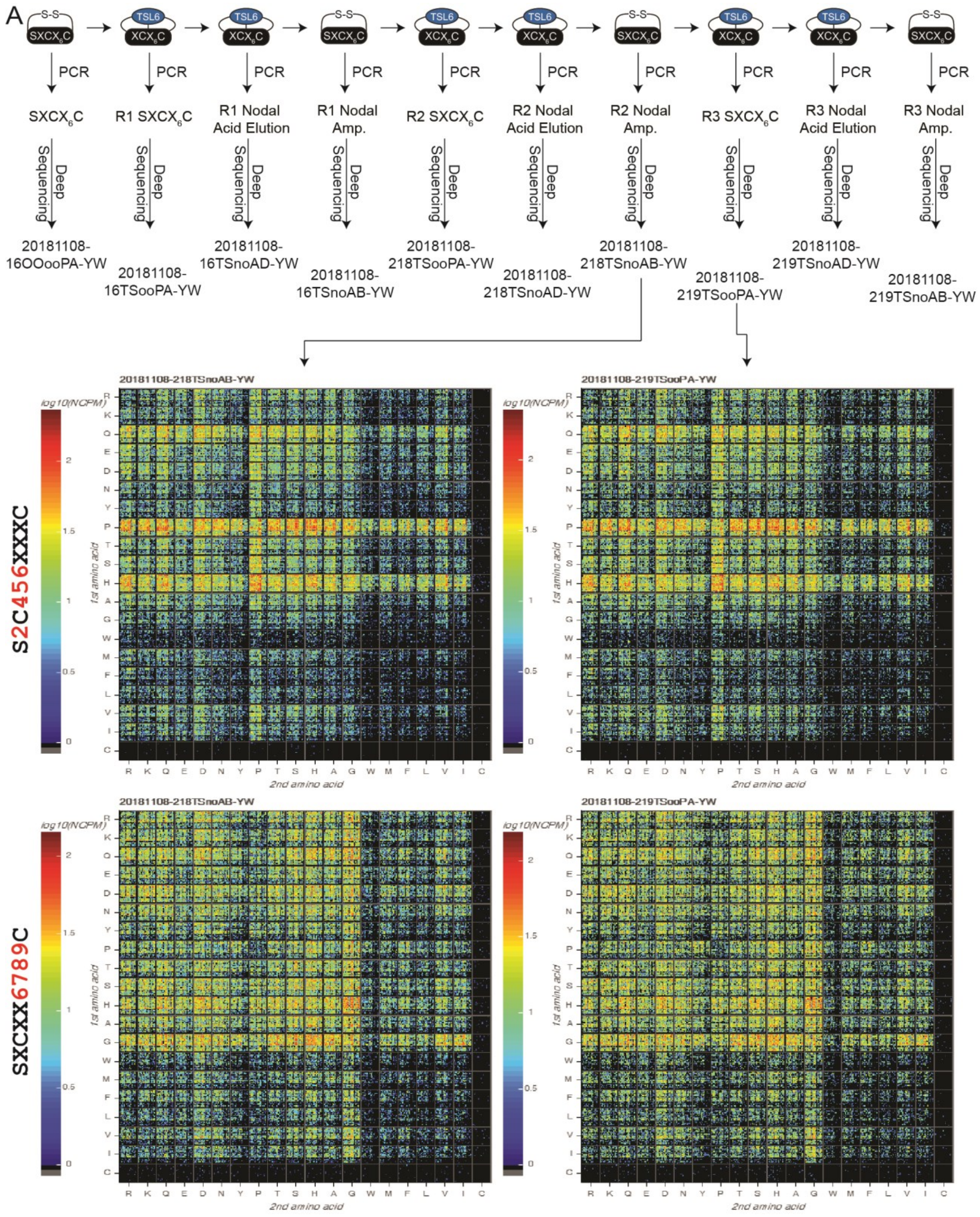

Figure S10: 20 × 20 plot comparison before and after **TSL-6** modification after R2 selection

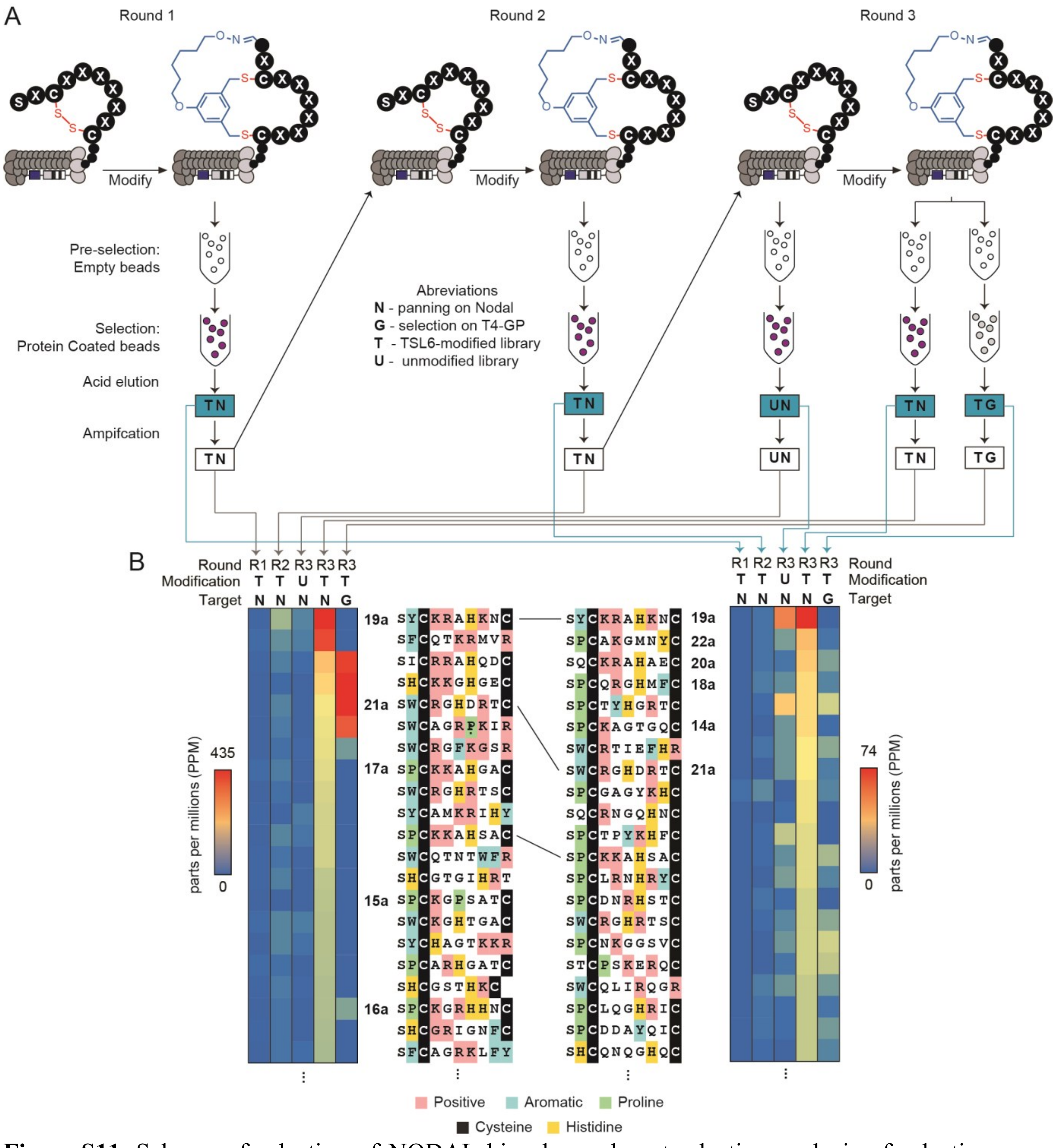

Figure S11: Scheme of selection of NODAL bicycles and post-selection analysis of selection samples . (A) A detail flow chat of 3 rounds of panning against Nodal protein. (B) Heat map of top 22 sequences after amplification (right) and acid elution. (left) Sequences were rank from high to low in the TN.

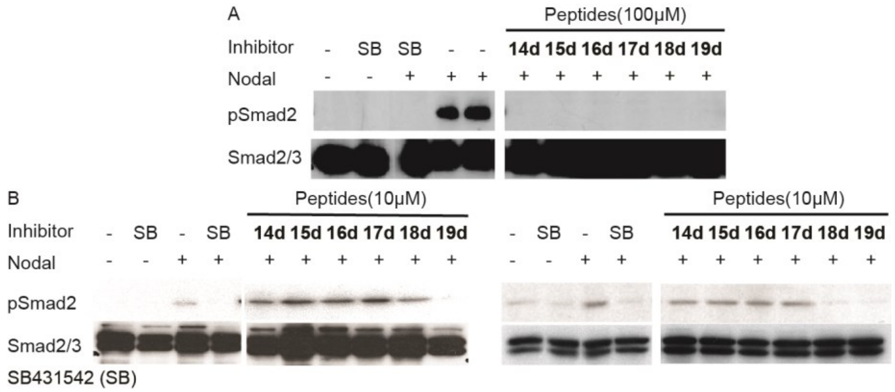

Figure S12: Western blot analysis of p-SMAD2 in response to treatment with rhNODAL and bicycles inhibitors at 100 µM (A) and 10 µM (B) in P19 cells. Total SMAD2/3 used as controls.

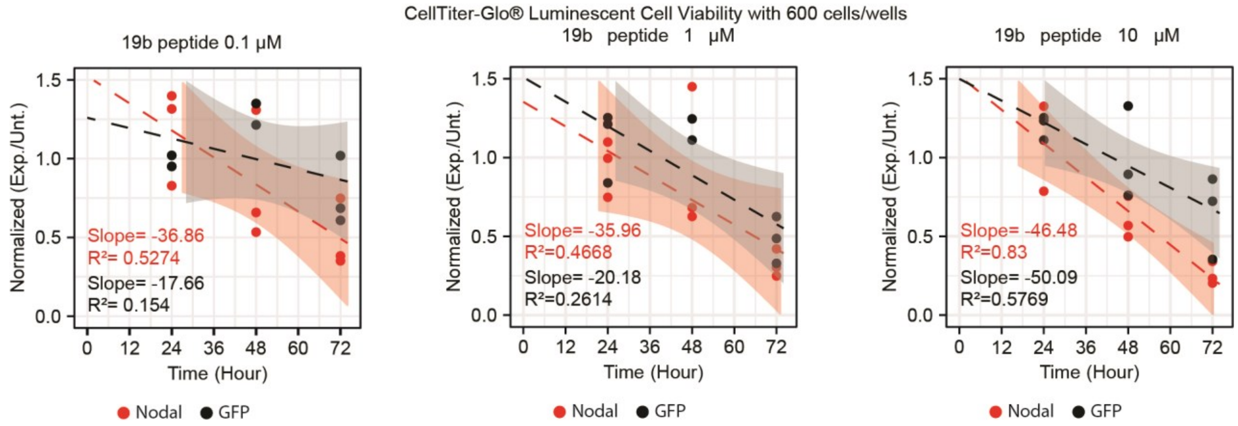

Figure S13: CellTiter-Glo® Luminescent Cell Viability 600 cells/wellAssay with TYK-nu-Nodal and TYK-nu-GFP treated with **19b** peptide inhibitor at 10 µM, 1 µM and 0.1 µM over 72 hours.

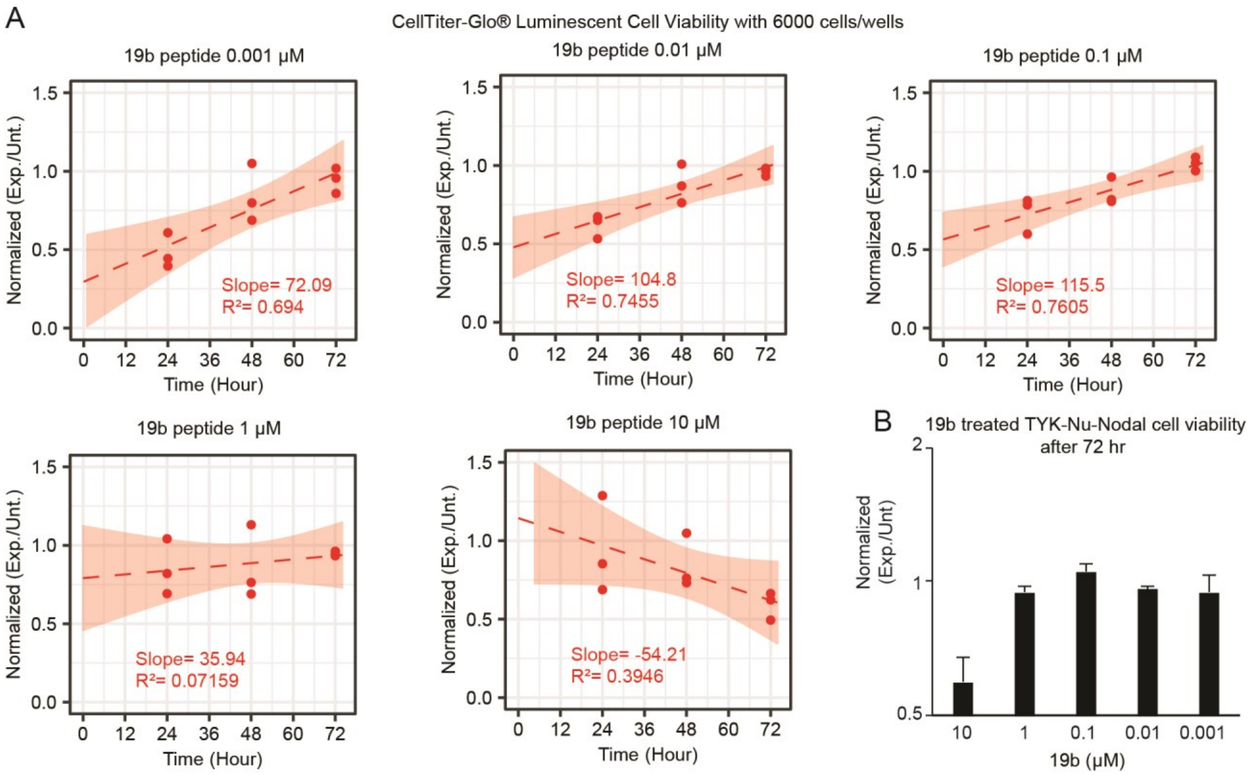

Figure S14: CellTiter-Glo® Luminescent Cell Viability 6000 cells/wellAssay with TYK-nu-Nodal treated with **19b** peptides inhibitors at 10 µM, 1 µM, 0.1 µM, 0.01 µM and 0.001 µM over 72 hours.

4. Proteolytic Stability Methods

4.1. *Protocol for measurement of proteolytic stability in cell assay:*
10 µL of the solution was sampled from the cell media and quenched with 190 µL of 50% aq. CH_3CN. The mixture was vortexed and centrifuged in a bench top centrifuge at 14000 RPM to precipitate any proteins. The supernatant was maintained at 4 °C until analysis by LCMS.

4.2. *Protocol for measurement of proteolytic stability in Pronase^{TM} :*
In a 600 µL Eppendorf tube, we combined 196 µL PBS (pH 7.4), 2 µL of corresponding peptide solution (from 10 mM stock) and 2 µL of 0.1 mg/mL Pronase^{TM}. The mixture was vortexed and incubated at 37 °C. At indicated time points, 10 µL of the solution was sampled, quenched with 190 µL of 50% aq. CH_3CN and maintained at 4 °C until analysis by LCMS.

4.3. *Protocol for measurement of proteolytic stability in fresh mouse serum:*
In a 600 µL Eppendorf tube, we combined 198 µL fresh mouse serum, 2 µL of corresponding peptide solution (from 10 mM stock). The mixture was vortexed and incubated at 37 °C. At indicated time points, 10 µL of the solution was sampled, quenched with 190 µL of 50% aq. CH_3CN. The mixture was vortexed and centrifuged in a bench top centrifuge at 14000 RPM to precipitate the serum protein. The supernatant was maintained at 4 °C until analysis by LCMS.

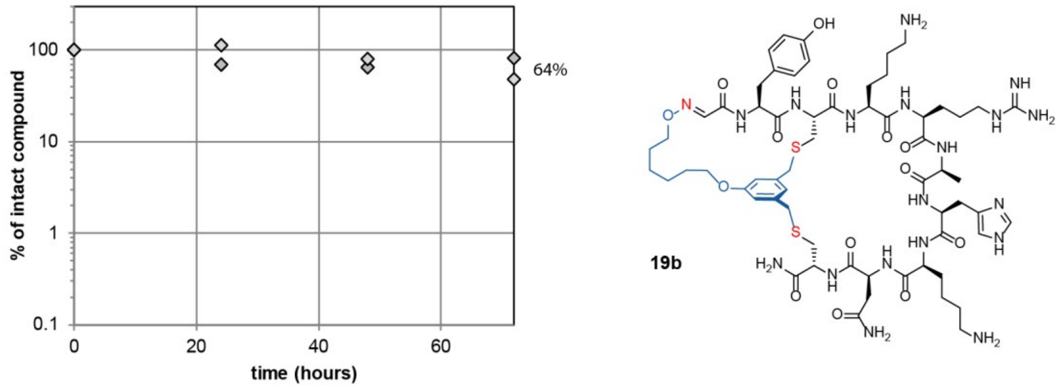

Figure S15: Peptide stability in active P19 cell culture for 72 hours of **19b**.

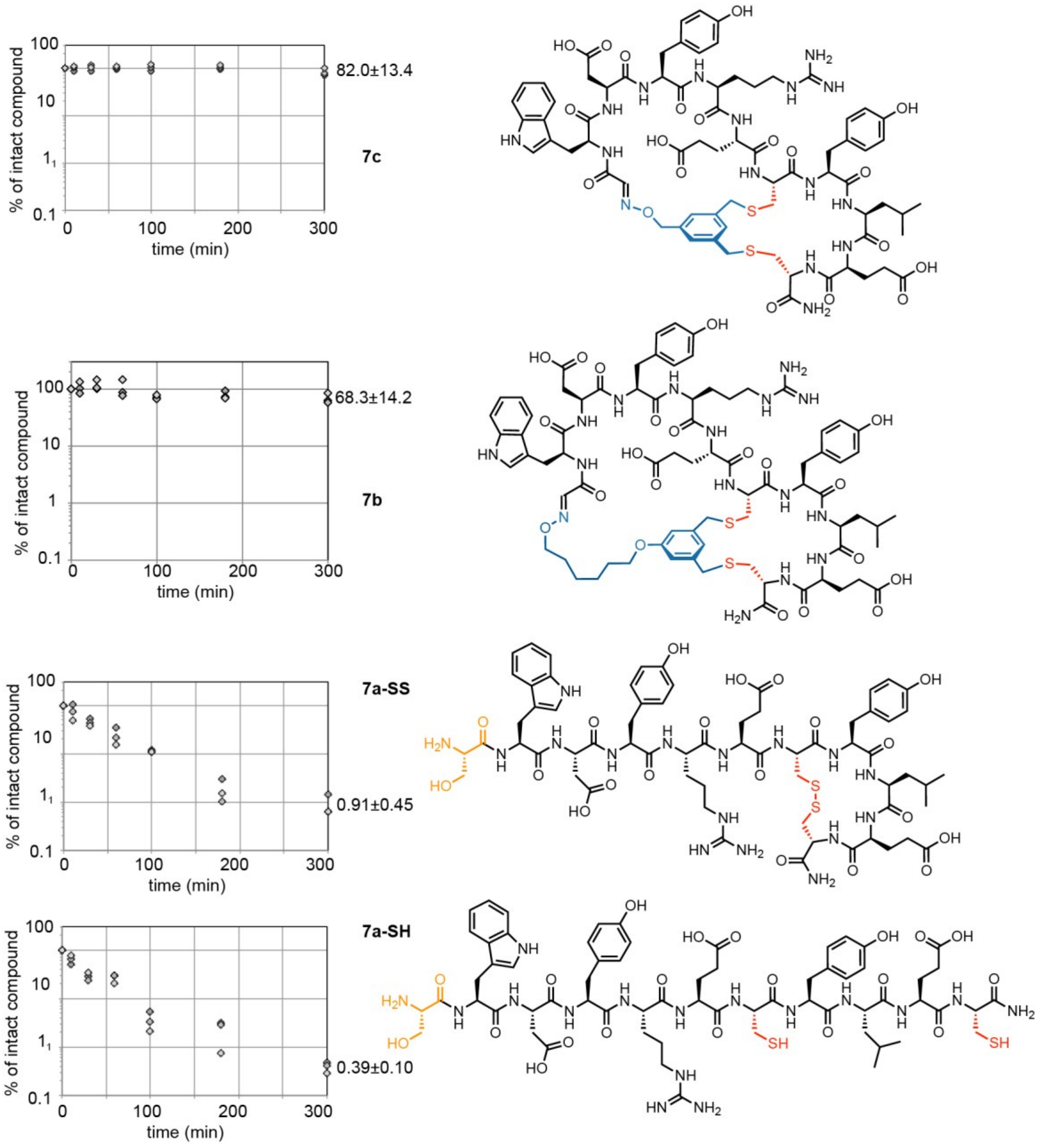

Figure S16: Proteolytic stability of **7a**, **7b** and **7c** in Pronase^{TM}: **7a** disulfide-peptide and **7a** linear peptide.

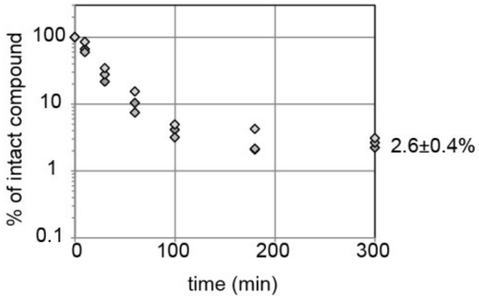

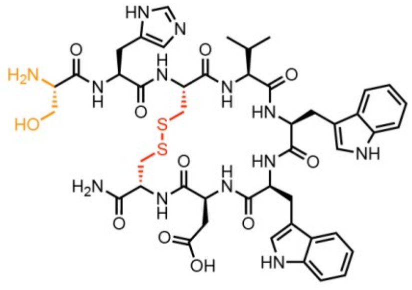

8a-SS

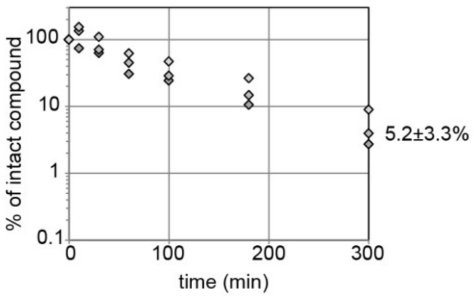

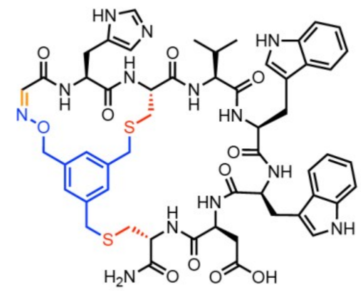

8c

Figure S17: Proteolytic stability of **8a**, and **8c** in Pronase^{TM}

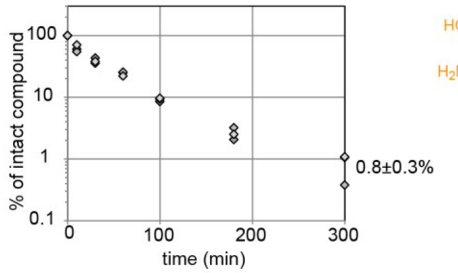

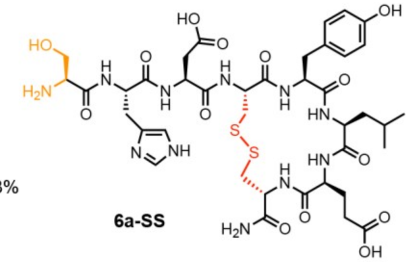

6a-SS

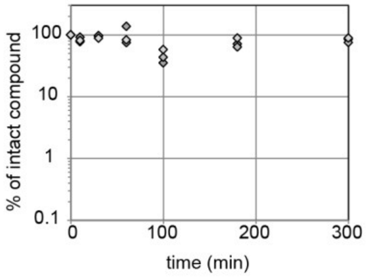

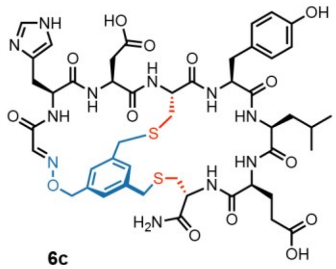

6c

Figure S18: Proteolytic stability of **6a** and **6c** in Pronase^{TM}.

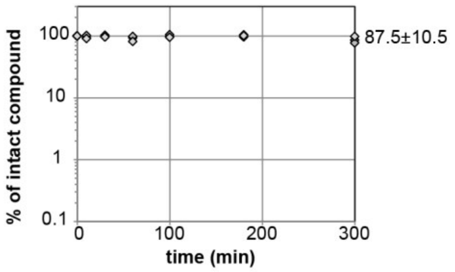

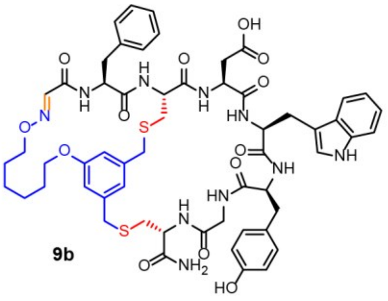

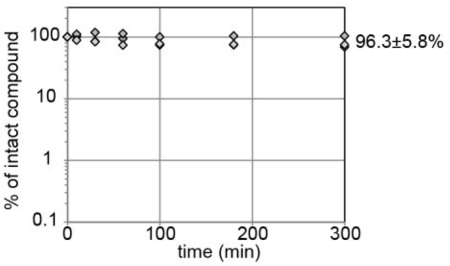

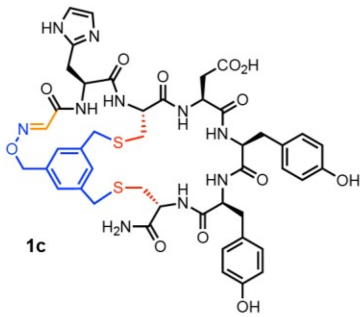

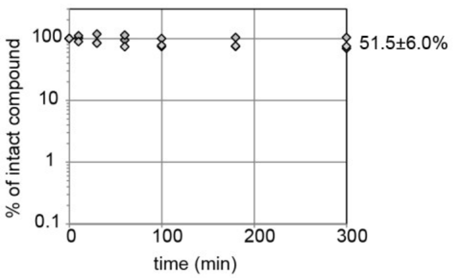

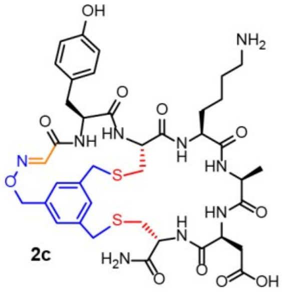

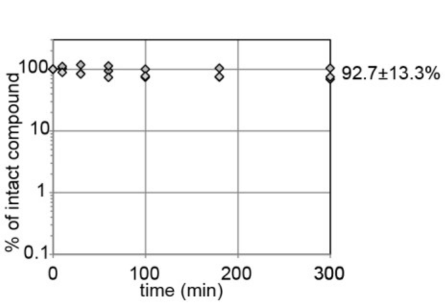

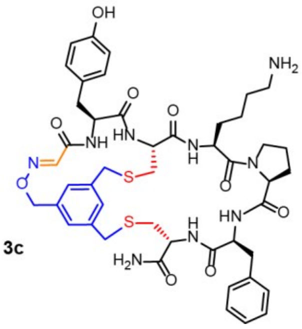

Figure S19: Proteolytic stability of **9b**, **1c**, **2c** and **3c** in Pronase^{TM}.

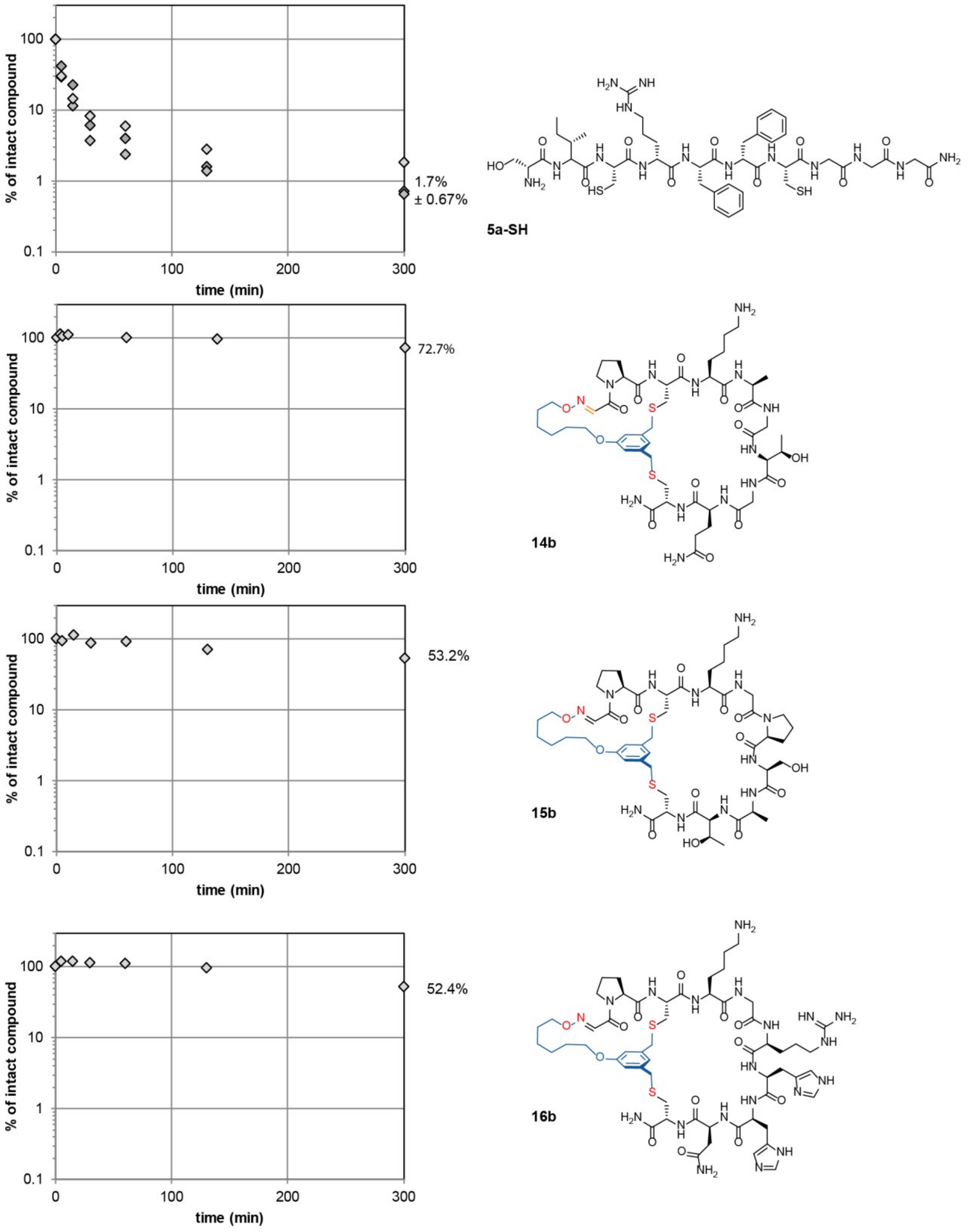

Figure S20: Proteolytic stability of **5a**, **14b**, **15b**, and **16b** in Pronase^{TM}.

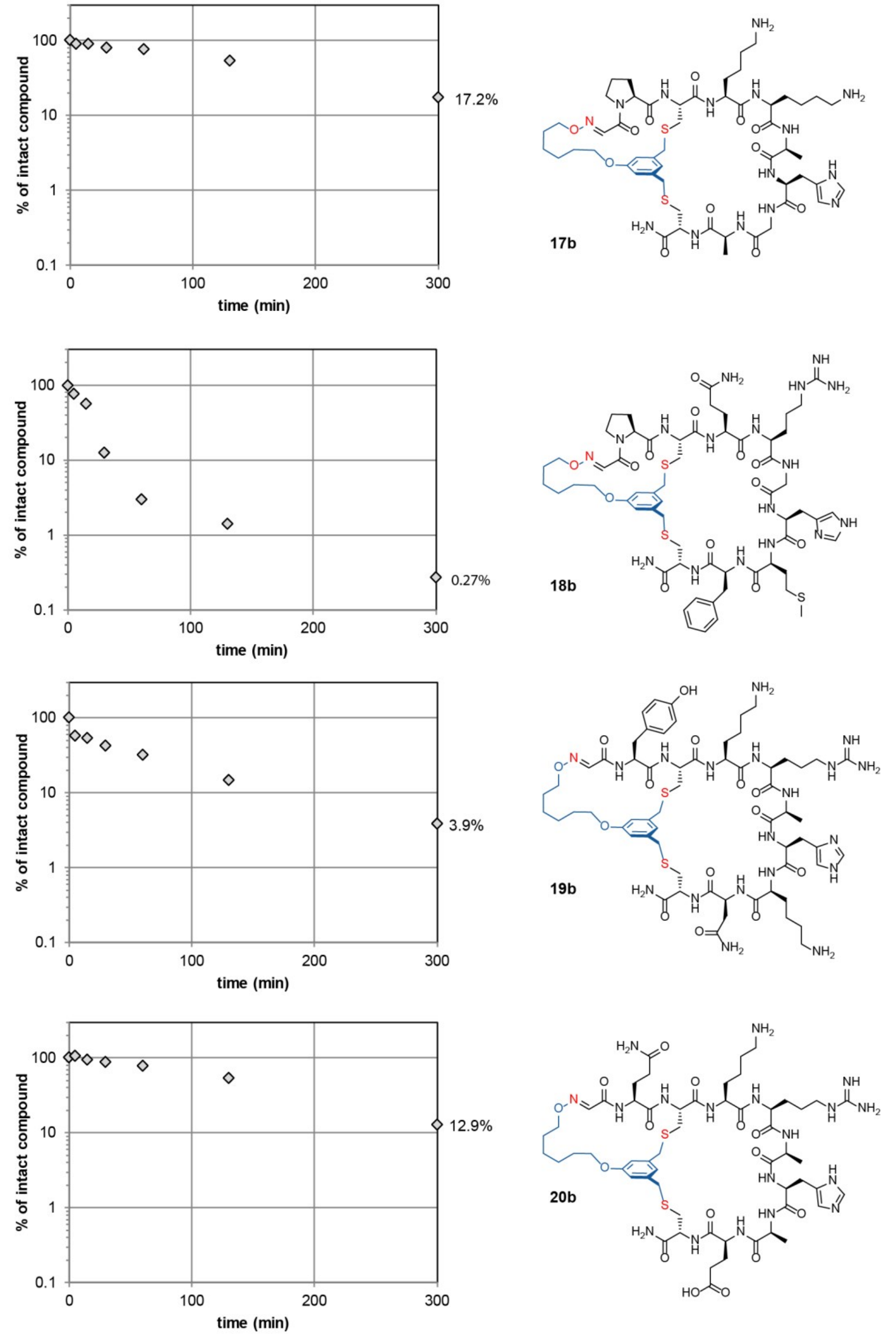

Figure S21: Proteolytic stability of **17b**, **18b**, **19b**, and **20b** in Pronase™.

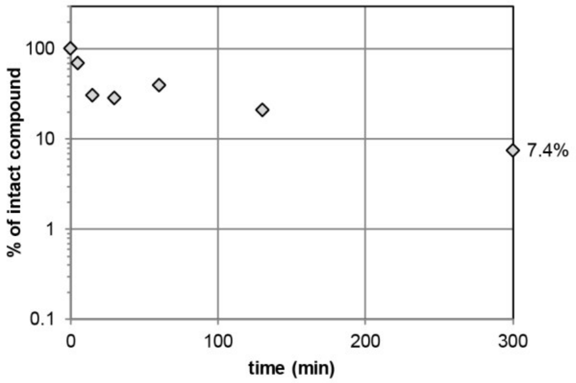

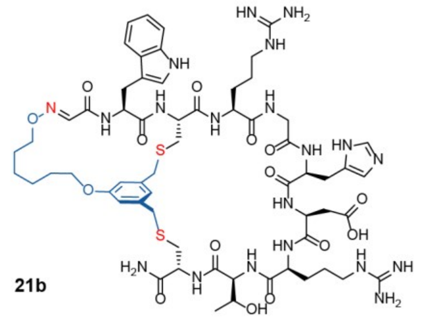

21b

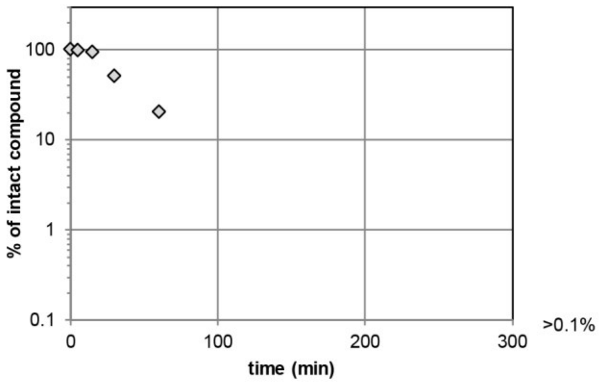

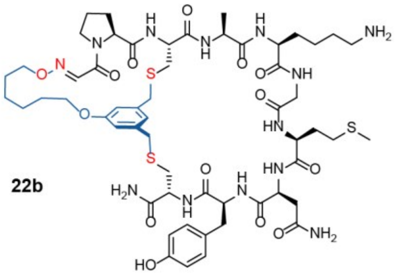

22b

Figure S22: Proteolytic stability of **21b** and **22b** in Pronase^{TM}.

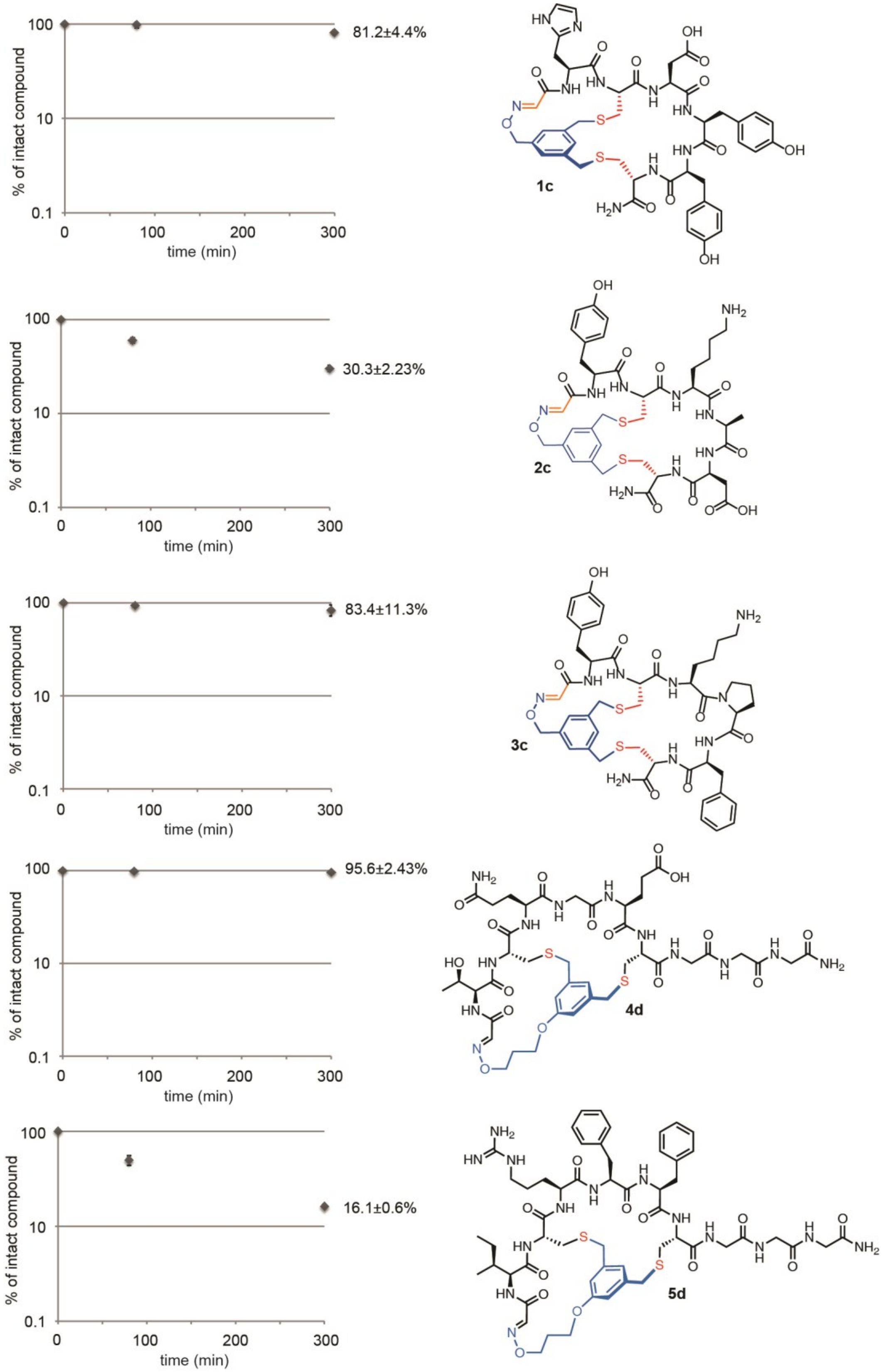

Figure S23: Proteolytic stability of **1c, 2c, 3c**, **4d** and **5d** in fresh mouse serum.

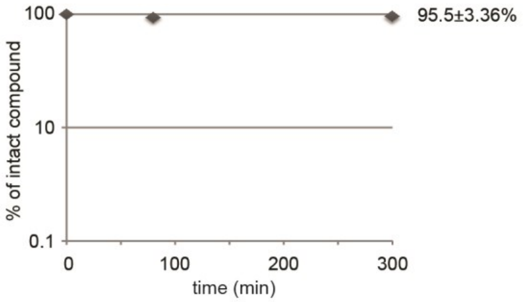

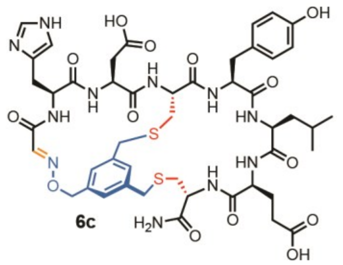

6c

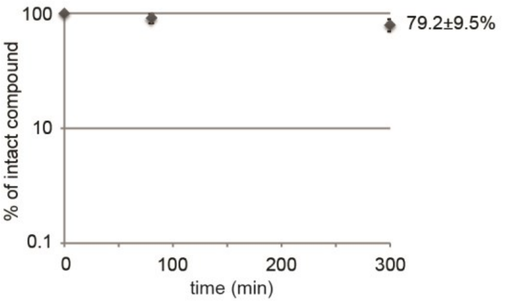

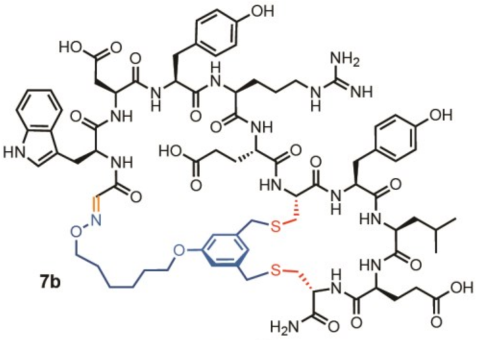

7b

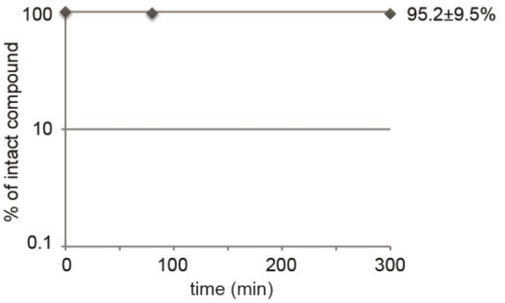

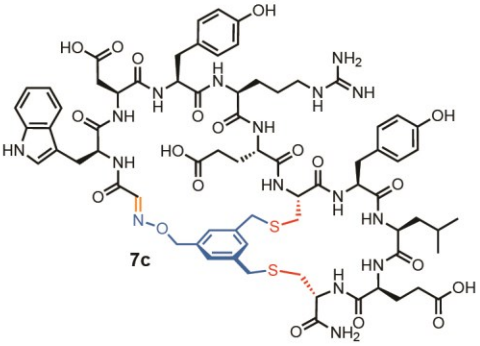

7c

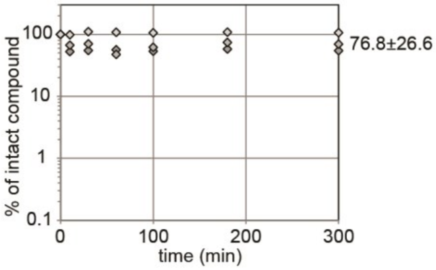

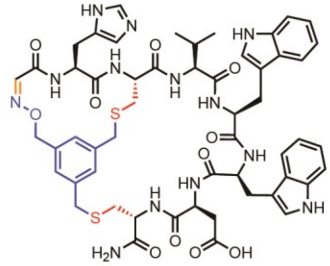

8c

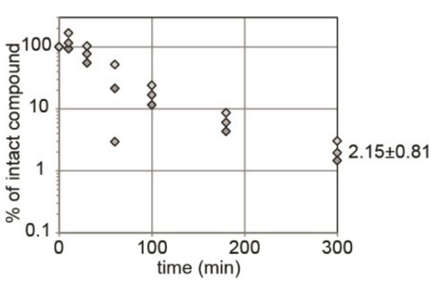

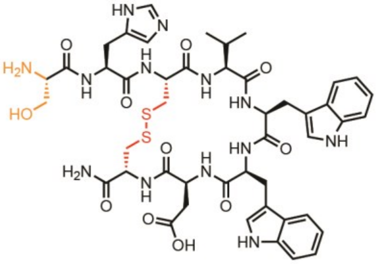

8a-SS

Figure S24: Proteolytic stability of **5d**, **6c**, **7b**, **7c**, **8c** and **8a** in fresh mouse serum.

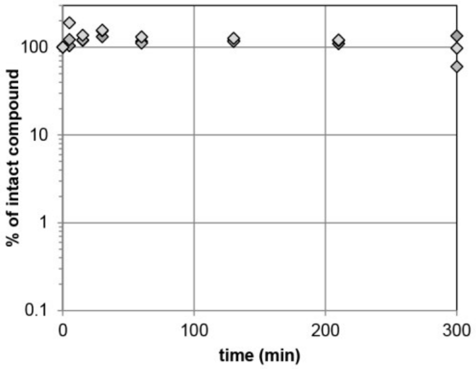

97.5%
± 37.3%

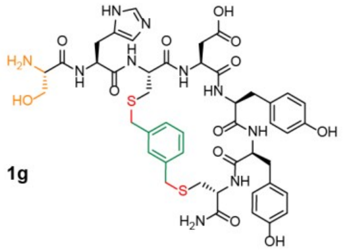

1g

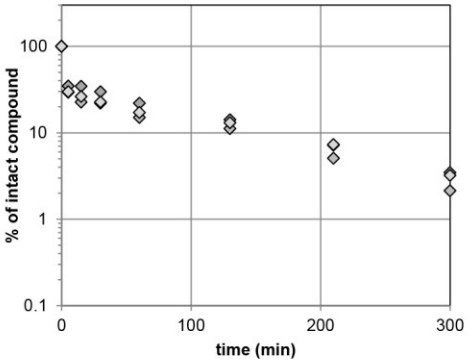

2.94%
± 0.7%

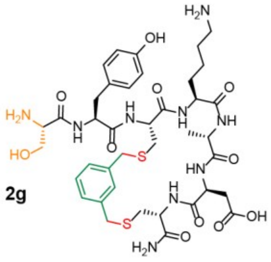

2g

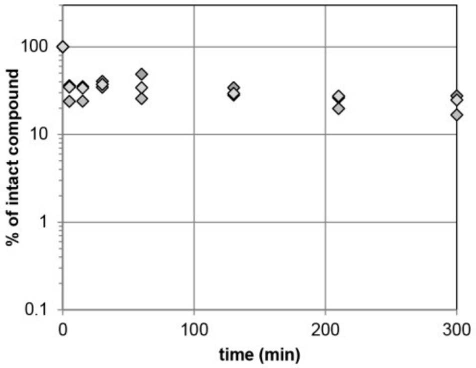

22.97%
± 5.61%

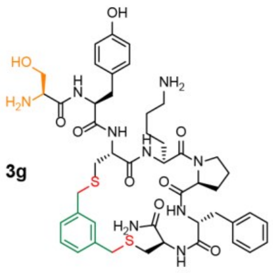

3g

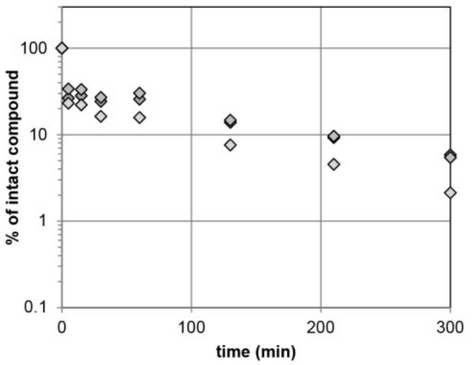

4.47%
±2.04%

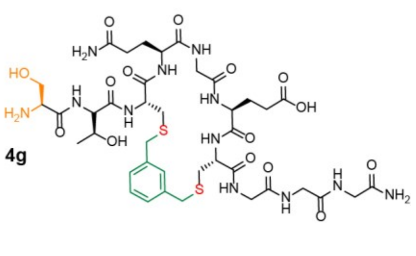

4g

Figure S25: Proteolytic stability of **1g**, **2g**, **3g**, and **4g** in Pronase™.

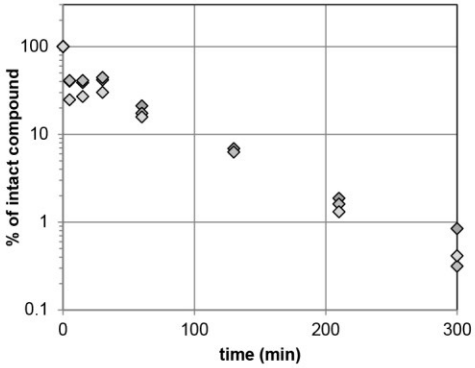

0.52%
± 0.28%

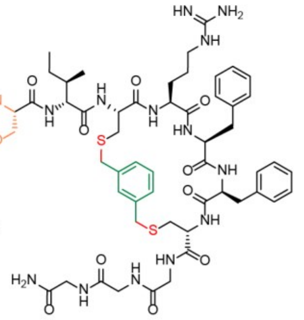

5g

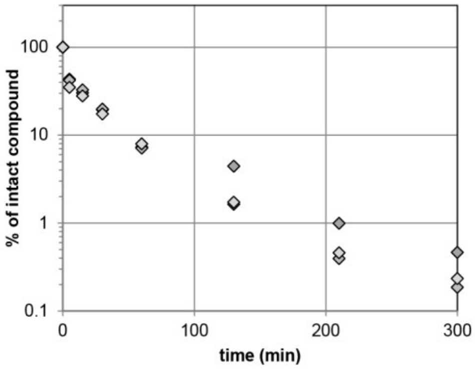

0.29%
± 0.15%

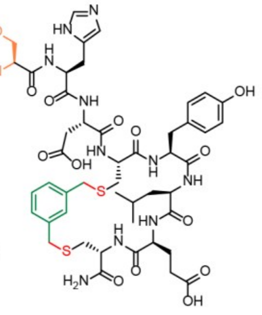

6g

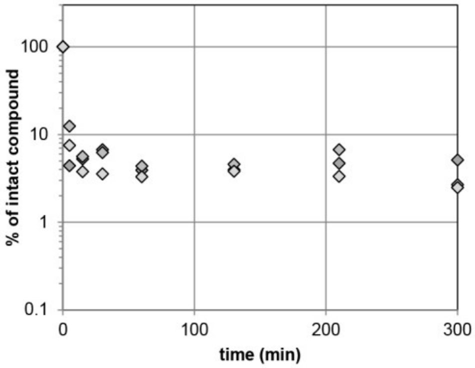

3.42%
± 1.47%

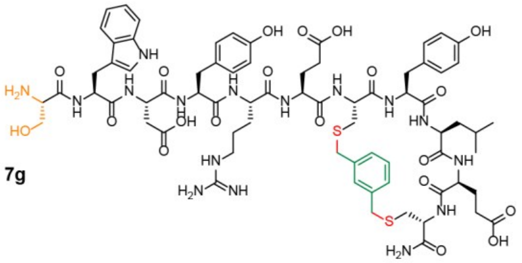

7g

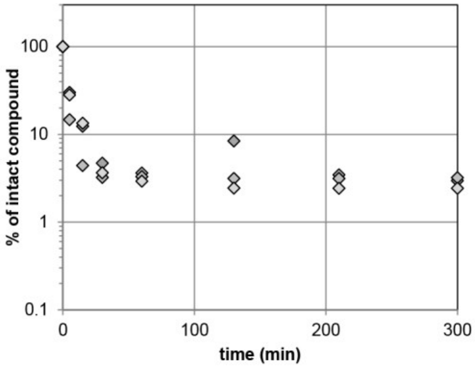

2.87%
±0.41%

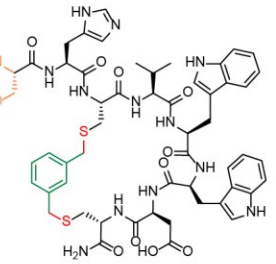

8g

Figure S26: Proteolytic stability of **5g**, **6g**, **7g**, and **8g** in Pronase^{TM}.

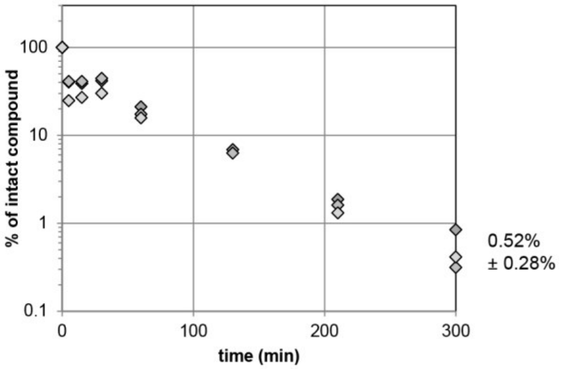

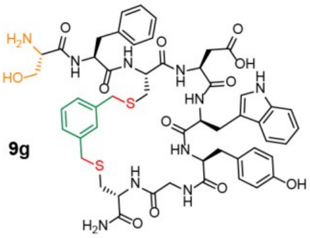

9g

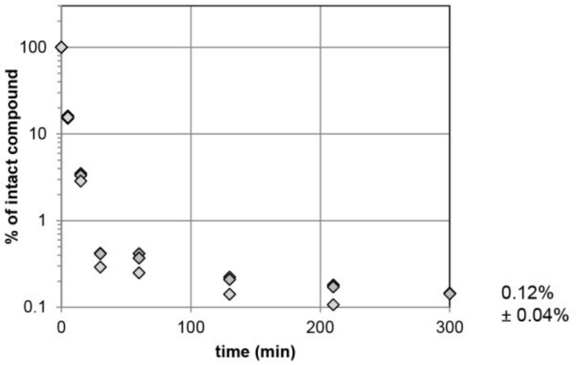

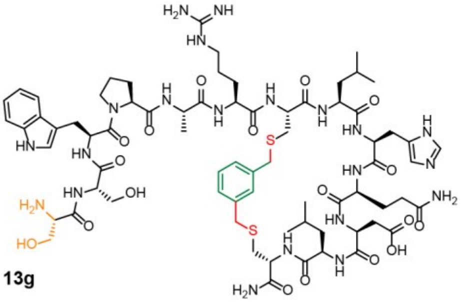

13g

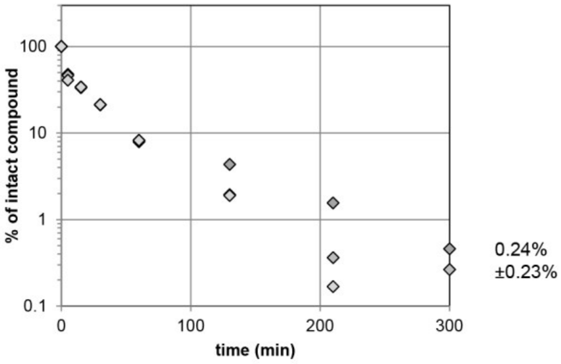

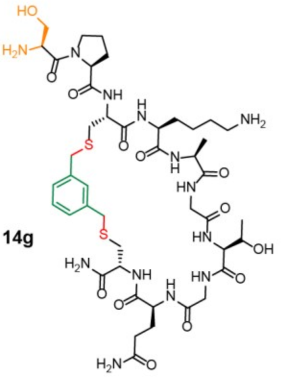

14g

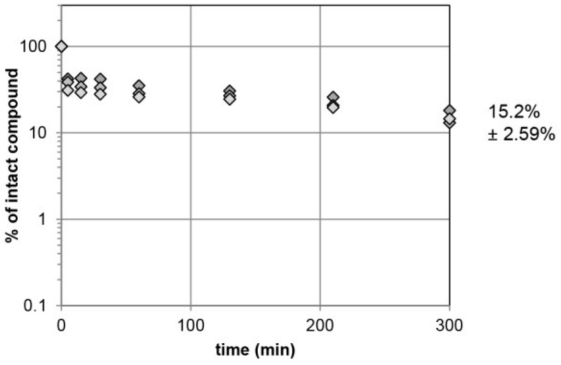

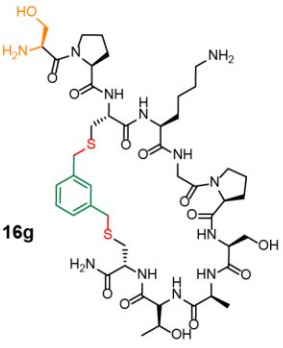

16g

Figure S27: Proteolytic stability of **9g**, **13g**, **14g**, and **16g** in Pronase^{TM}.

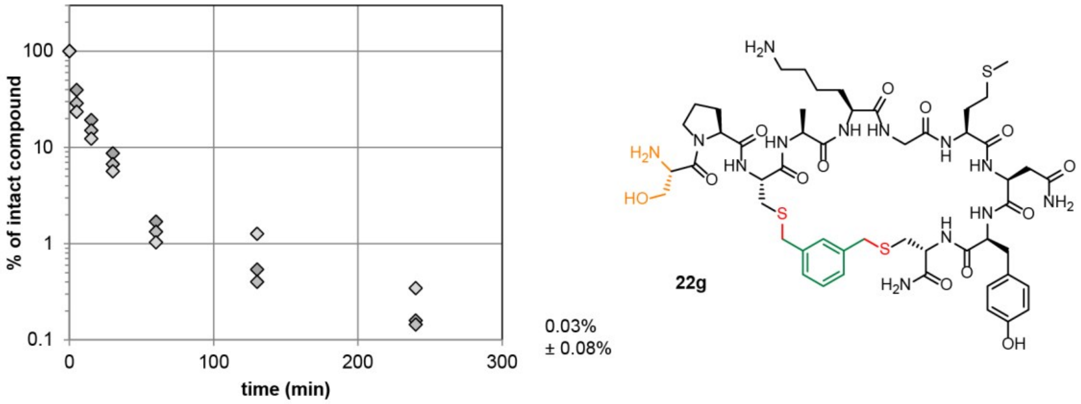

Figure S28: Proteolytic stability of **22g** in Pronase™.

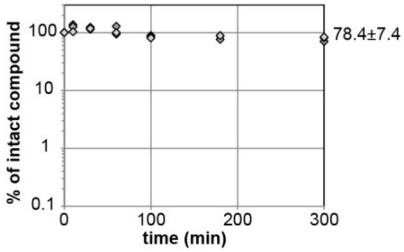

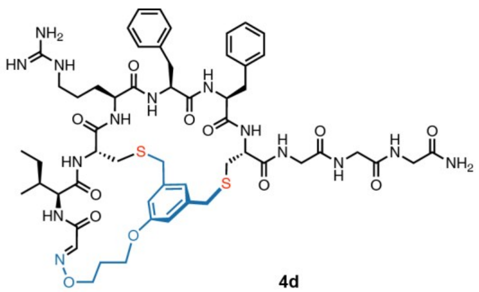

4d

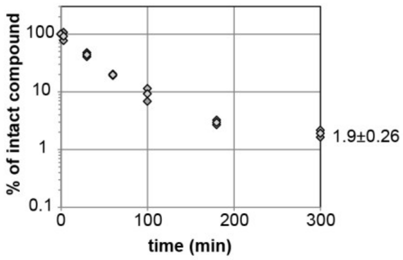

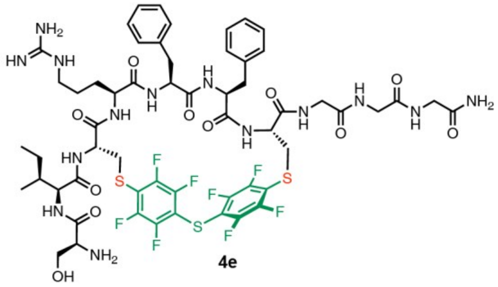

4e

Figure S29: Proteolytic stability of **4d** and **4e** in Pronase™.

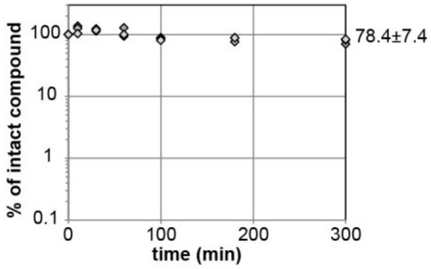

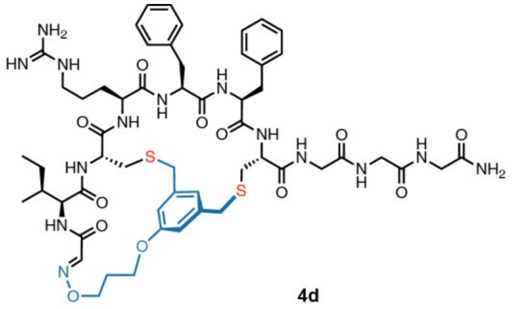

4d

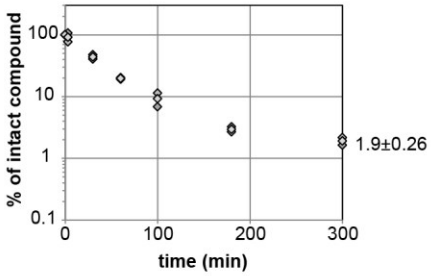

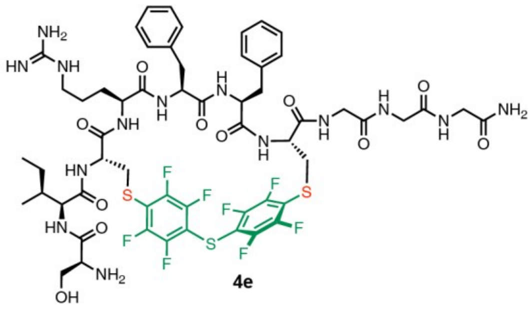

4e

Figure S30: Proteolytic stability of **5d** and **5e** in Pronase™.

5. Molecular Dynamics Simulation

Molecular dynamics (MD) simulations were performed for four bicyclic peptides (**8c**, **8b**, **7c**, and **7b**; Figure S30). The initial structure of each peptide was built using the Maestro 11.7 software of Schrödinger.[10] The topology file for each peptide was generated using the Schrödinger utility ffld_server and converted to the GROMACS format using the ffconv.py script.[11] All MD simulations in this study were performed using the GROMACS 4.6.7 suite[12] with the OPLS 2005 force field and the TIP4P water model.[13-14] The initial structure was first energy minimized for 1000 steps and then solvated in a cubic box of water molecules. The box size was chosen such that the distance between the peptide and the box wall was at least 1.5 nm. Minimal explicit counter ions were also added to neutralize the net charge of the system. With all heavy atoms of the linker restrained, the solvated system was further energy minimized for 5000 steps. Each initial structure was subjected to 1000 independent runs starting from different initial velocities. With all the heavy atoms of the linker remained restrained to their initial coordinates, a 50 ps NVT (isochoric-isothermal) equilibration at 300 K was performed for each of the 1000 runs, followed by a 50-ps NPT (isobaric-isothermal) equilibration at 300 K and 1 bar to adjust the solvent density. The equilibrated system then underwent a simulated annealing process in the NVT ensemble. The system temperature was first increased to 600 K in 500 ps and maintained at 600 K for additional 500 ps. The temperature was then decreased gradually to 300 K in 1 ns. During the simulated annealing, the position restraints for the linker were removed. In all the simulations, the temperature was regulated using the v-rescale thermostat[15] with a coupling time constant of 0.1 ps. To avoid the "hot solvent/cold solute" artifacts, [16-17] two separated thermostats were applied to the solvent (water and ions) and the peptide. Then, the system underwent a 1 ns equilibration process at 300 K and 1 bar without position restraints, followed by a 5 ns production simulation also at 300 K and 1 bar. For the NPT simulations, the pressure was maintained using the isotropic Berendsen barostat [18] with a coupling time of 2.0 ps and compressibility of 4.5×10^{-5} bar^{-1}. For all the MD simulations, bonds involving hydrogen were constrained using the LINCS algorithm. [19] A 2-fs time step was used with the leapfrog integrator.[20] The nonbonded interactions (Lennard-Jones and electrostatic) were truncated at 1.0 nm. Long-range electrostatic interactions were treated using the Particle Mesh Ewald summation method.[21-22] A long-range analytic dispersion correction was applied to both the energy and pressure to account for the truncation of Lennard-Jones interactions. The last frame of each production run was used for further analysis. The 1000 final structures for each system could be found in the Data.zip/MD_movies provided in the Supporting Information.

Cluster analysis was performed for the peptide backbone by binning the torsional angles within the ring structures; however, the ω dihedrals describing the peptide amide bonds were not included as they were all in the *trans* conformation. The bicyclic peptides had two cycles in each molecule. Cycle 1 was defined as the cycle containing the *N*-terminal residues up to the first Cys (orange circles in Figure S30). Cycle 2 was defined as the cycle containing the residues between the first and the second Cys's (blue circles in Figure S30). The cluster analysis was performed on each of the two cycles. The populations of the top 10 clusters for the two cycles for **8c**, **8b**, **7c**, and **7b** are shown in the table S4 and table S5 below. In Figure S30, the binning boundaries for each residue are shown as green lines.

In general, the bicyclic peptides linked with **TSL-1** were better structured than its counterpart linked with **TSL-6** in cycle 1 (orange circles in Figure S30). As observed in the Table S4 below,

when clustering based on the conformations of cycle 1, **8c** (bicyclized with **TSL-1**) showed two clusters that have significant populations (> 20%), while the populations of the top clusters of **8b** (bicyclized with **TSL-6**) were all relatively small (~2%). Similarly, when clustering based on the conformations of cycle 1, the top cluster of **7c** (bicyclized with **TSL-1**) had a population of 16.3%, but the population of the top cluster of **7b** (bicyclized with **TSL-6**) was only 1.2%. Overall, bicyclic peptides linked by **TSL-6** seemed to be quite flexible in cycle 1, as there were no structures with significant populations. However, the difference in cycle 2 (the cycle containing residues between the two Cys's; blue circles in Figure S30) was much smaller between bicyclic peptides linked with **TSL-1** and that linked with **TSL-6**, as shown in the Table S5 below, likely because cycle 2 of both the **TSL-1**-linked and the **TSL-6**-linked compounds shared the same molecular topology. It was also found that when comparing the Ramachandran plots between **8c** and **8b**, and similarly between **7c** and **7b**, the residue(s) near the *N*-terminus exhibited different distributions of backbone dihedral angles when the peptide was linked by **TSL-1** vs. **TSL-6**, as indicated by smaller normalized integrated products[23] between the two sets of Ramachandran plots (Figure S30). Specifically, compared to the other resides, the His residue showed a larger difference in the (ϕ, ψ) distribution between **8c** and **8b**; similarly, compared to the other resides, the Trp and Asp residues showed a larger difference in the (ϕ, ψ) distribution between **7c** and **7b**.

Table S4: Populations of the top 10 clusters of **8c**, **8b**, **7c**, and **7b** using the torsional angles in cycle 1 in the cluster analysis.

Cluster #	8c	8b	7c	7b
**1**	21.8%	2.3%	16.3%	1.2%
**2**	21.5%	2.1%	7.0%	0.4%
**3**	9.5%	1.8%	3.1%	0.4%
**4**	9.4%	1.8%	2.6%	0.3%
**5**	6.3%	1.7%	2.2%	0.3%
**6**	4.9%	1.7%	1.5%	0.3%
**7**	4.1%	1.0%	1.3%	0.3%
**8**	3.6%	1.0%	1.2%	0.3%
**9**	2.1%	1.0%	1.2%	0.3%
**10**	2.0%	0.7%	1.0%	0.3%

Table S5: Populations of the top 10 clusters of **8c**, **8b**, **7c**, and **7b** using the torsional angles in cycle 2 in the cluster analysis.

Cluster #	8c	8b	7c	7b
**1**	7.6%	12.7%	7.3%	9.9%
**2**	5.4%	3.7%	5.4%	3.9%
**3**	4.4%	2.2%	4.7%	2.6%
**4**	4.1%	2.2%	3.9%	2.2%
**5**	2.6%	2.1%	3.9%	2.2%
**6**	2.5%	2.0%	3.6%	2.0%
**7**	2.5%	1.9%	3.0%	1.9%
**8**	2.1%	1.7%	3.0%	1.8%
**9**	2.0%	1.7%	2.8%	1.6%
**10**	1.6%	1.5%	2.0%	1.3%

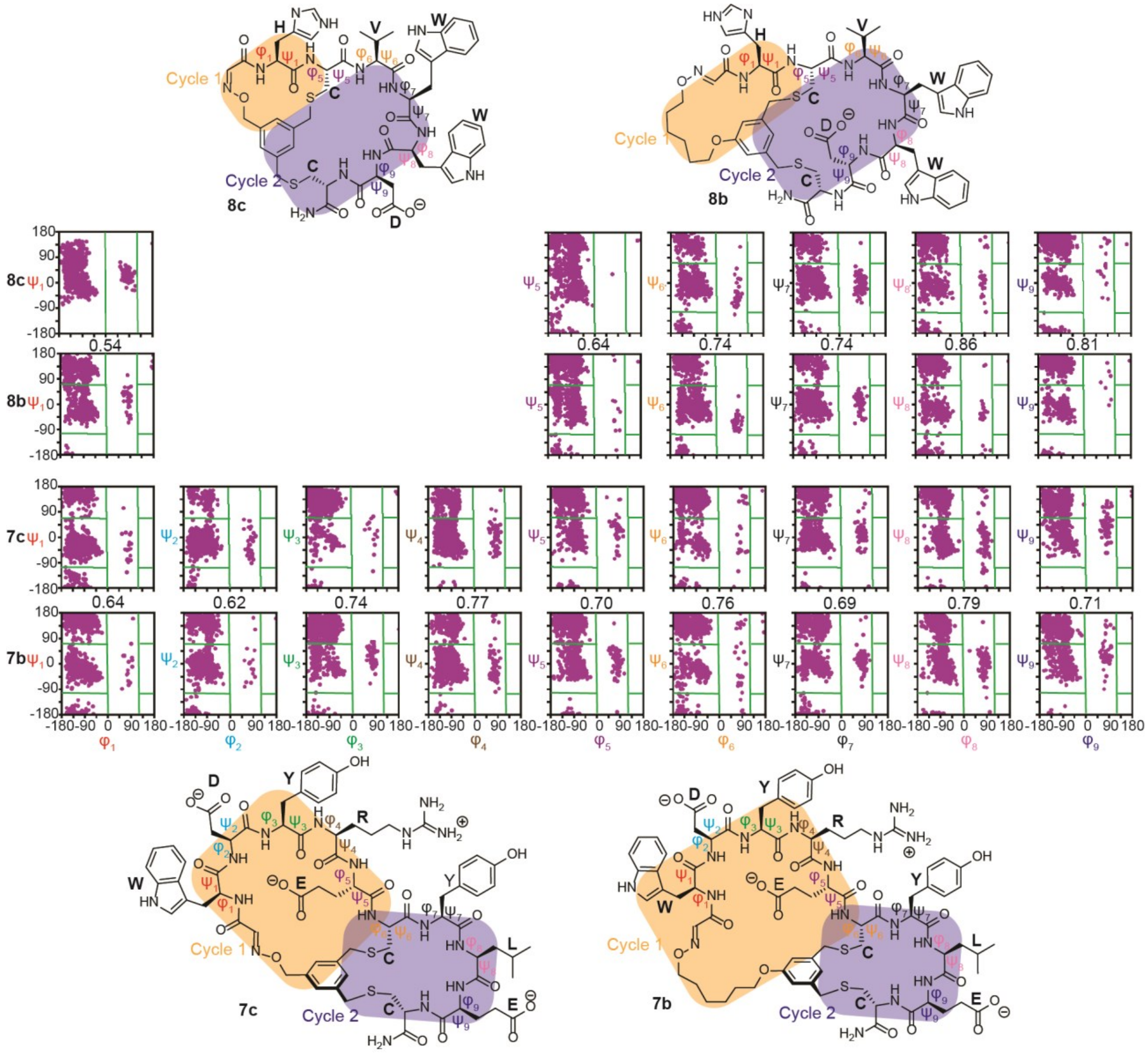

Figure S31: Ramachandran plot of the cyclic peptide backbone for **8c**, **8b**, **7c** and **7b**.: Green lines indicate the binning boundaries used in the cluster analysis. The numbers shown between the Ramachandran plots of **8c** and **8b,** and between those of **7c** and **7b** are the normalized integrated product (NIP) calculated as $\mathrm{NIP} = \frac{2\sum_i \rho_{i,\mathrm{peptide1}}\rho_{i,\mathrm{peptide2}}}{\sum_i \rho_{i,\mathrm{peptide1}}{}^2 + \sum_i \rho_{i,\mathrm{peptide2}}{}^2}$.[23] NIP takes a value between 0 and 1, with 0 indicating no overlap between the two distributions and 1 indicating the two distributions are identical.

6. Summary of synthesis

6.1. *Summary of* ***TSL****s Peptides Synthesis*

SHCDYYC-TSL1

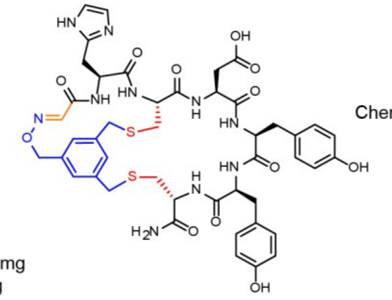

Chemical Formula: $C_{45}H_{50}N_{10}O_{12}S_2$
Exact Mass: 986.31

Starting material mass = 5.6 mg
Final product mass = 1.4 mg

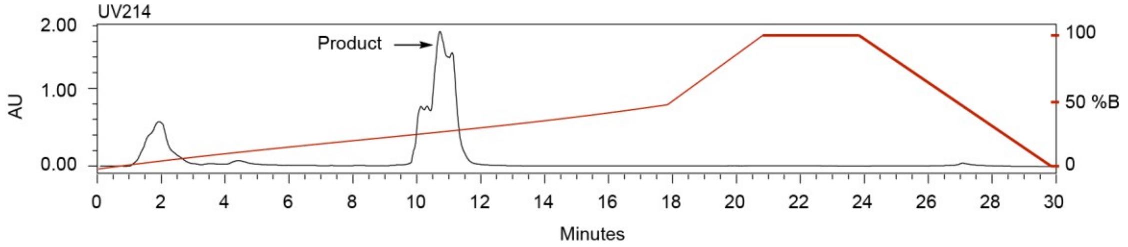

Gradient Table

Time (min)	Solvent B (%)
0	2
2	2
18	50
21	100
24	100
26	2
30	2

Flow rate: 8 mL/min
Symmetry C18 prep column
(100 A, 5 μm, 19 mm X 50 mm)

Solvent A: H_2O + 0.1% (v/v) TFA
Solvent B: CH_3CN + 0.1% (v/v) TFA

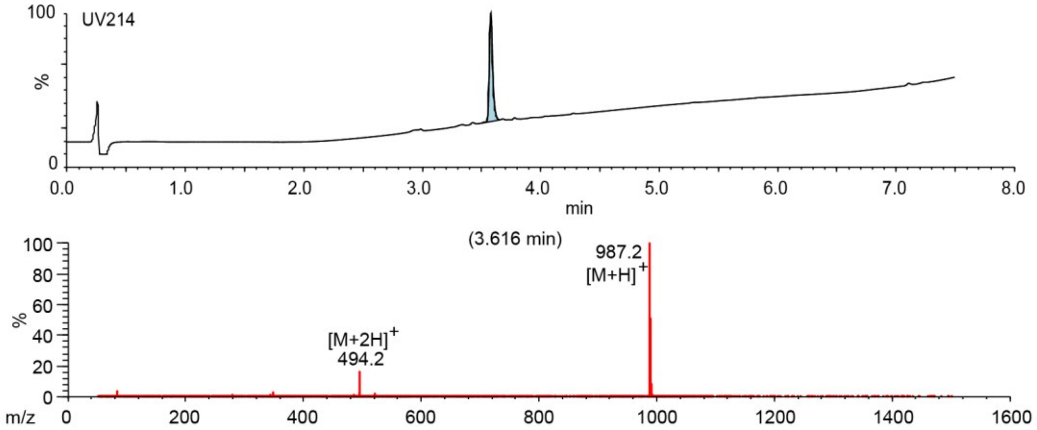

Figure S32: Summary for **1c** synthesis

SYCKADC-TSL1

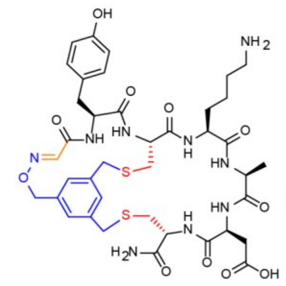

Chemical Formula: $C_{39}H_{51}N_9O_{11}S_2$
Exact Mass: 885.3149

Starting material mass = 9.0 mg
Final product mass = 3.7mg

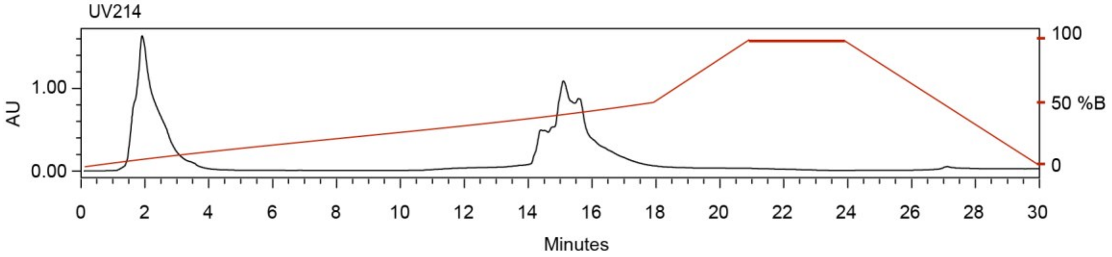

Gradient Table

Time (min)	Solvent B (%)
0	2
2	2
18	50
21	100
24	100
26	2
30	2

Flow rate: 8 mL/min
Symmetry C18 prep column
(100 A, 5 μm, 19 mm X 50 mm)

Solvent A: H_2O + 0.1% (v/v) TFA
Solvent B: CH_3CN + 0.1% (v/v) TFA

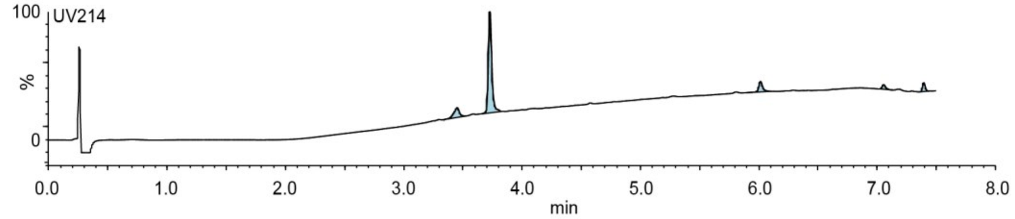

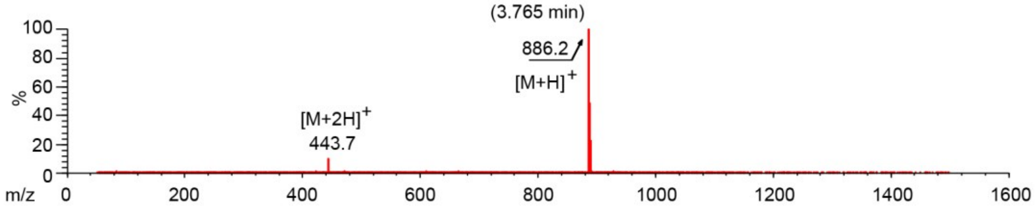

Figure S33: Summary for **2c** synthesis

SYCKPFC-TSL1

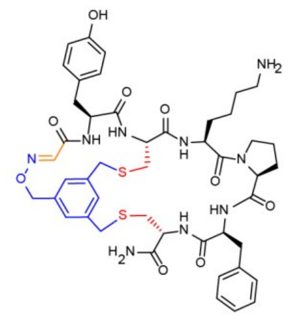

Chemical Formula: $C_{46}H_{57}N_9O_9S_2$
Exact Mass: 943.3721

Starting material mass = 10 mg
Final product mass = 4.6 mg

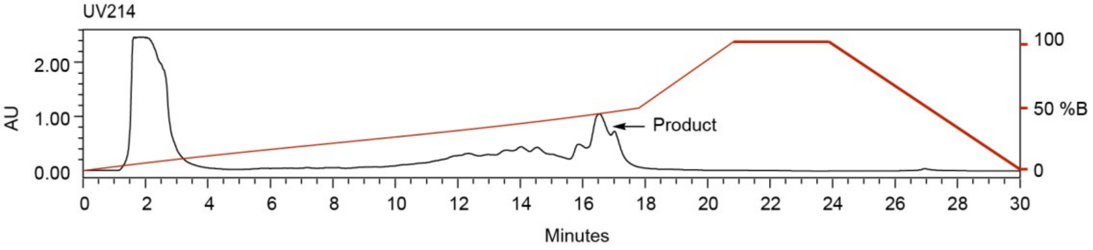

Gradient Table

Time (min)	Solvent B (%)
0	2
2	2
18	50
21	100
24	100
26	2
30	2

Flow rate: 8 mL/min
Symmetry C18 prep column
(100 A, 5 μm, 19 mm X 50 mm)

Solvent A: H_2O + 0.1% (v/v) TFA
Solvent B: CH_3CN + 0.1% (v/v) TFA

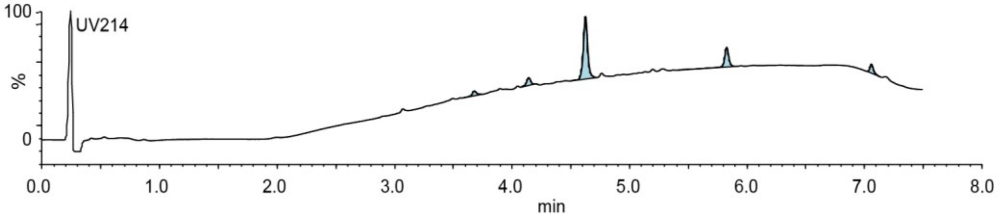

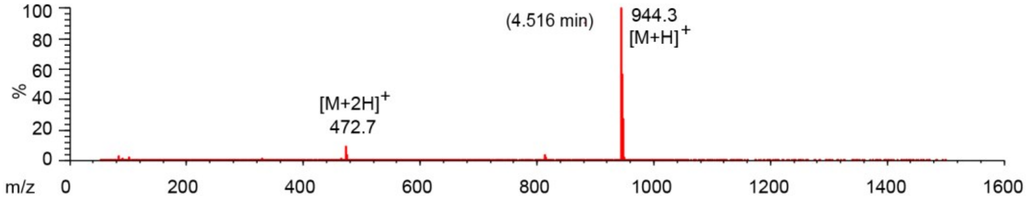

Figure S34: Summary for **3c** synthesis

STCQGECGGG-TSL3

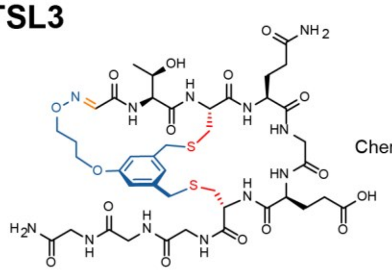

Chemical Formula: $C_{41}H_{58}N_{12}O_{16}S_2$
Exact Mass: 1038.35

Starting material mass = 5.8 mg
Final product mass = 3 mg

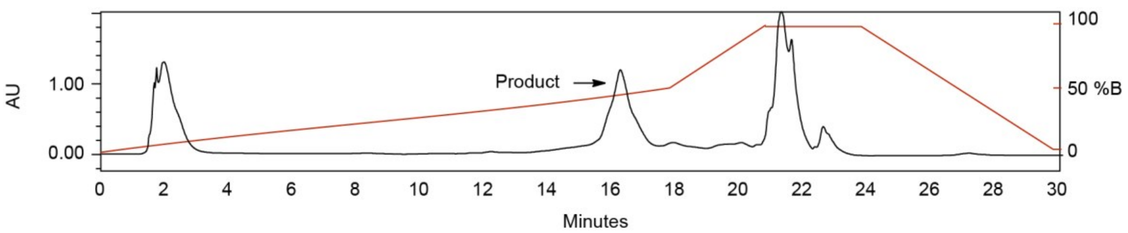

Gradient Table

Time (min)	Solvent B (%)
0	2
2	2
18	50
21	100
24	100
26	2
30	2

Flow rate: 8 mL/min
Symmetry C18 prep column
(100 A, 5 μm, 19 mm X 50 mm)

Solvent A: H_2O + 0.1% (v/v) TFA
Solvent B: CH_3CN + 0.1% (v/v) TFA

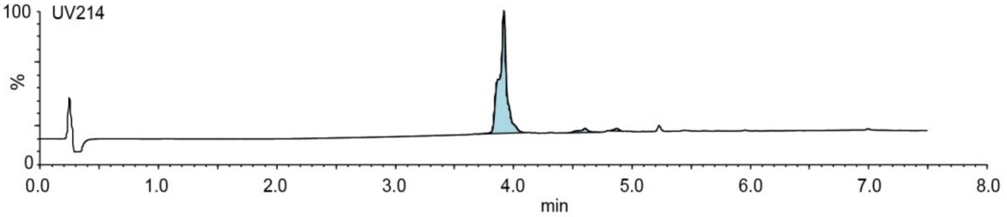

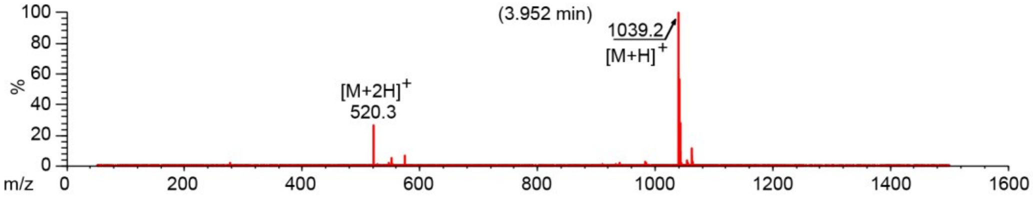

Figure S35: Summary for **4d** synthesis

SICRFFCGGG-TSL3

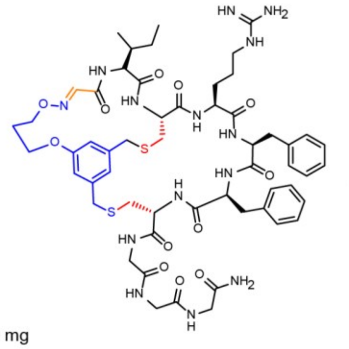

Chemical Formula: $C_{55}H_{74}N_{14}O_{12}S_2$
Exact Mass: 1186.5052

Starting material mass = 5.9 mg
Final product mass = 3.7 mg

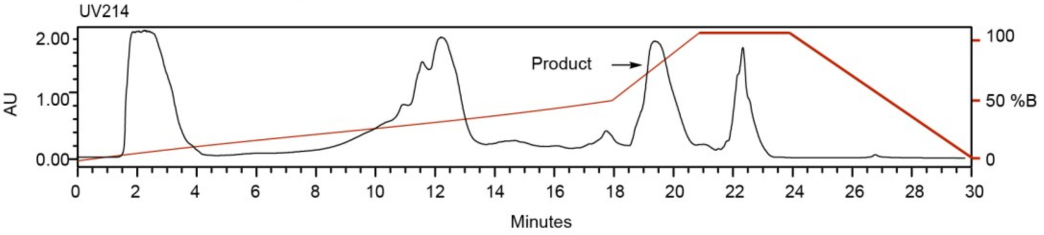

Gradient Table

Time (min)	Solvent B (%)
0	2
2	2
18	50
21	100
24	100
26	2
30	2

Flow rate: 8 mL/min
Symmetry C18 prep column
(100 A, 5 µm, 19 mm X 50 mm)

Solvent A: H_2O + 0.1% (v/v) TFA
Solvent B: CH_3CN + 0.1% (v/v) TFA

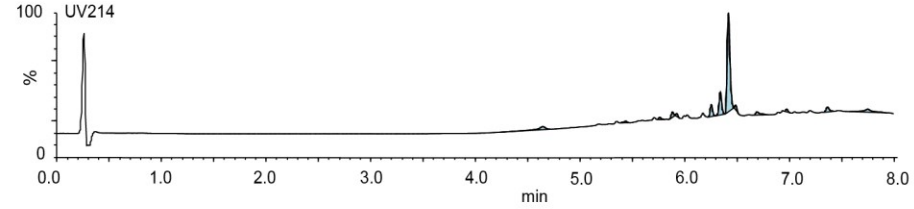

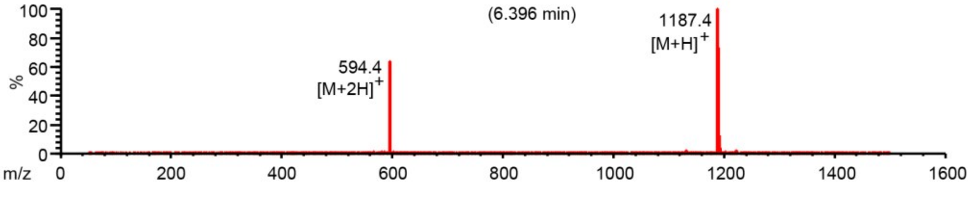

Figure S36: Summary for **5d** synthesis

SWDYRECYLEC-TSL1

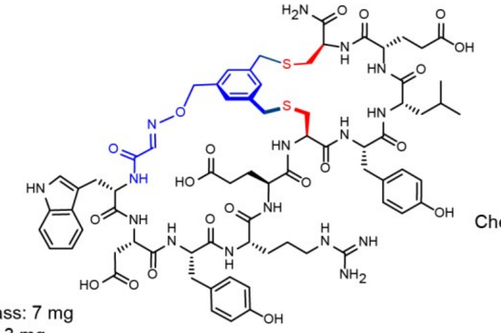

Chemical Formula: $C_{72}H_{90}N_{16}O_{20}S_2$
Exact Mass: 1562.60

Starting material mass: 7 mg
Final product mass: 3 mg

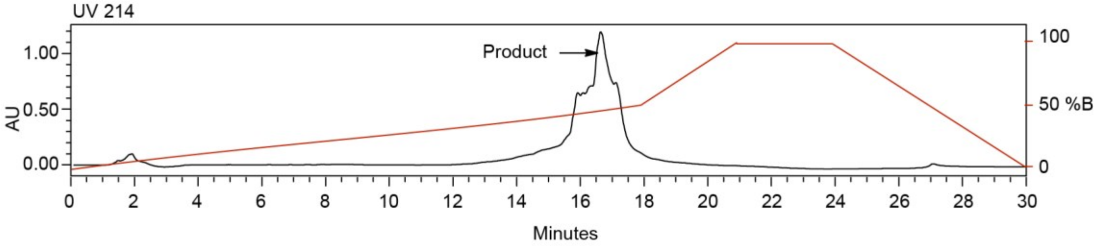

Gradient Table

Time (min)	Solvent B (%)
0	2
2	2
18	50
21	100
24	100
26	2
30	2

Flow rate: 8 mL/min
Symmetry C18 prep column
(100 A, 5 μm, 19 mm X 50 mm)

Solvent A: H_2O + 0.1% (v/v) TFA
Solvent B: CH_3CN + 0.1% (v/v) TFA

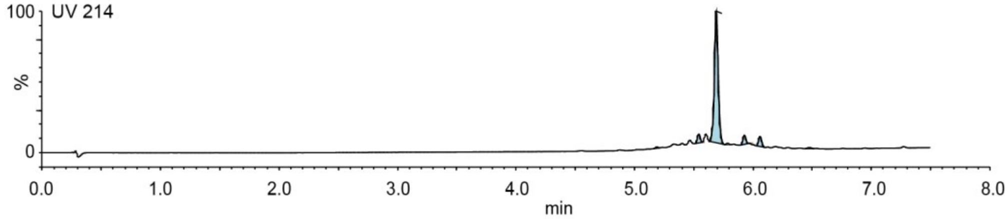

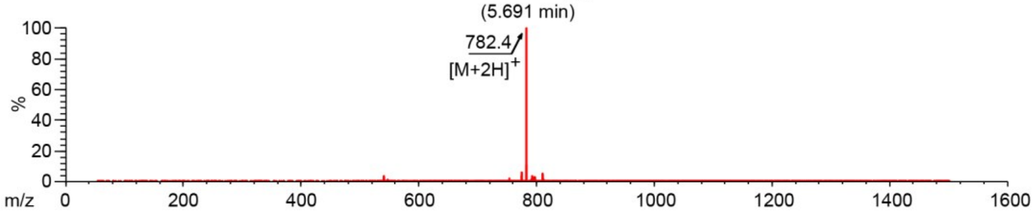

Figure S37: Summary for **7c** synthesis

SWDYRECYLEC-TSL6

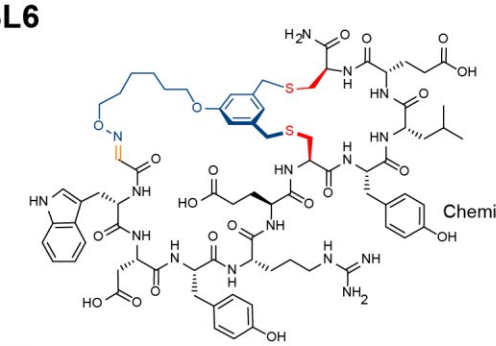

Chemical Formula: $C_{77}H_{100}N_{16}O_{21}S_2$
Exact Mass: 1648.6690

Starting material mass: 19 mg
Final product mass: 8 mg

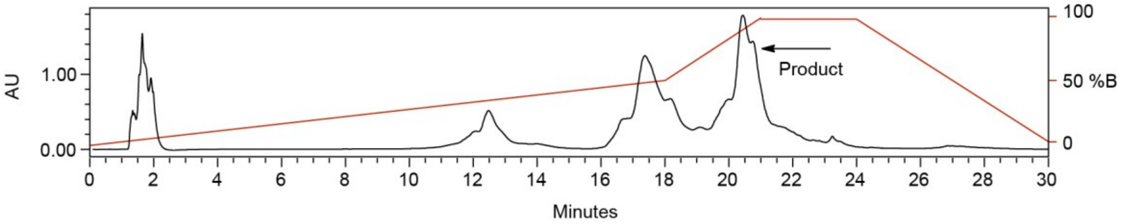

Gradient Table

Time (min)	Solvent B (%)
0	2
2	2
18	50
21	100
24	100
26	2
30	2

Flow rate: 8 mL/min
Symmetry C18 prep column
(100 A, 5 μm, 19 mm X 50 mm)

Solvent A: H_2O + 0.1% (v/v) TFA
Solvent B: CH_3CN + 0.1% (v/v) TFA

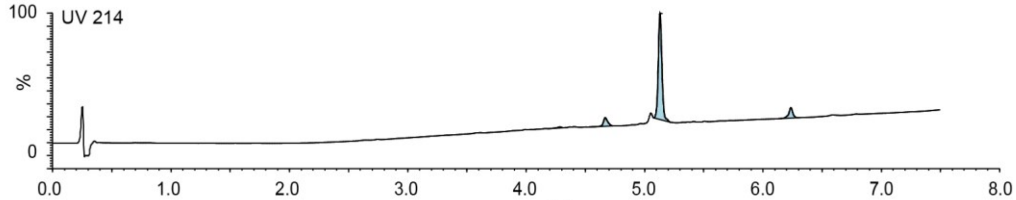

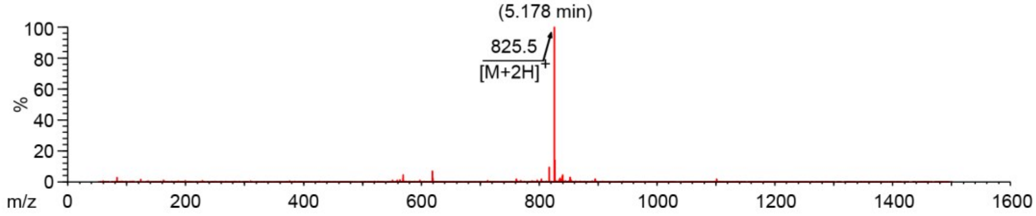

Figure S38: Summary for **7b** synthesis

SHCVWWDC-TSL1

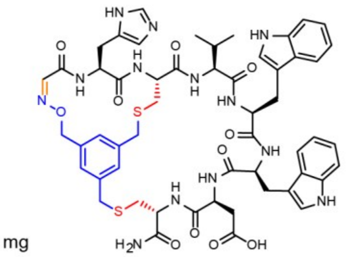

Chemical Formula: $C_{54}H_{61}N_{13}O_{11}S_2$
Exact Mass: 1131.41

Starting material mass = 9.1 mg
Final product mass = 4.2 mg

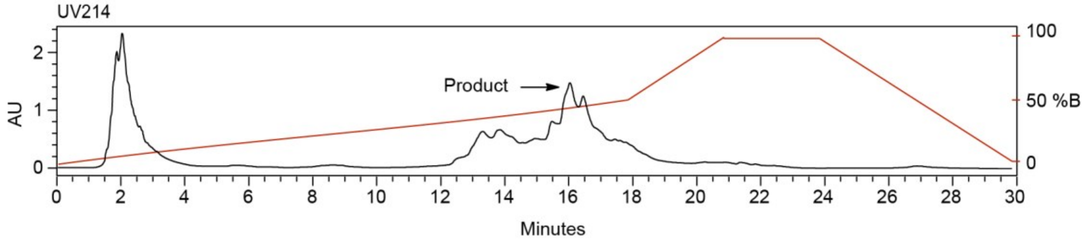

Gradient Table

Time (min)	Solvent B (%)
0	2
2	2
18	50
21	100
24	100
26	2
30	2

Flow rate: 8 mL/min
Symmetry C18 prep column
(100 A, 5 μm, 19 mm X 50 mm)

Solvent A: H_2O + 0.1% (v/v) TFA
Solvent B: CH_3CN + 0.1% (v/v) TFA

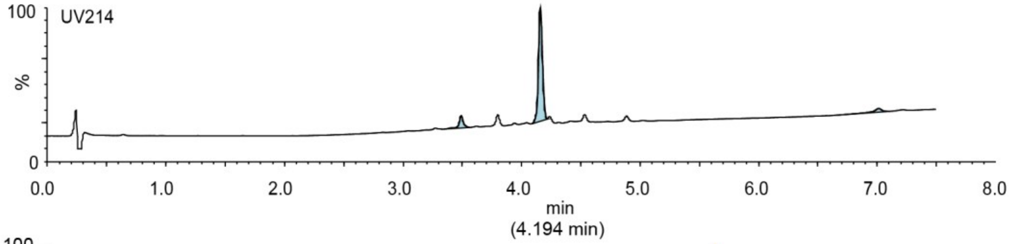

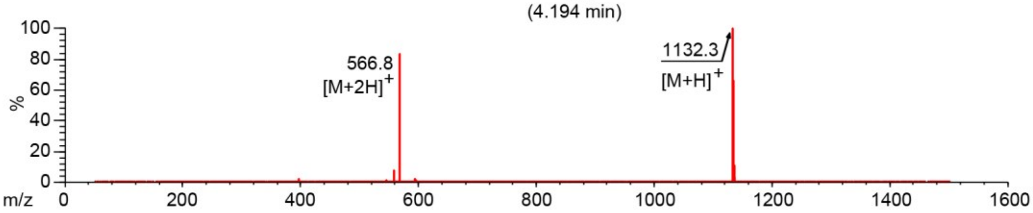

Figure S39: Summary for **8c** synthesis

SFCDWYGC-TSL6

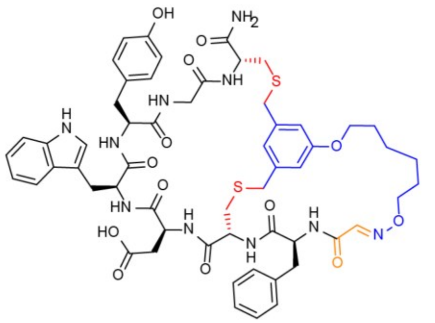

Chemical Formula: $C_{57}H_{66}N_{10}O_{13}S_2$
Exact Mass: 1162.4252

Starting material mass = 20 mg
Final product mass = 5 mg

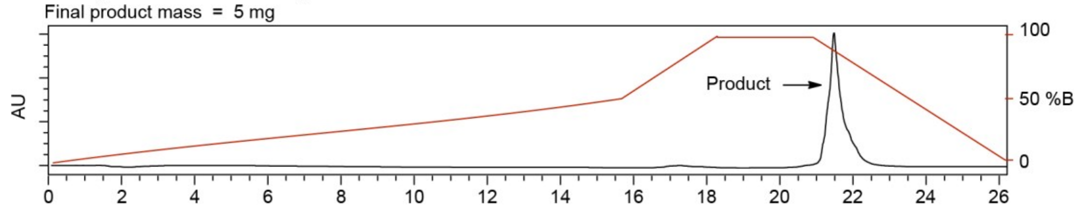

Gradient Table

Time (min)	Solvent B (%)
0	2
2	2
18	50
21	100
24	100
26	2
30	2

Flow rate: 8 mL/min
Symmetry C18 prep column
(100 A, 5 μm, 19 mm X 50 mm)

Solvent A: H_2O + 0.1% (v/v) TFA
Solvent B: CH_3CN + 0.1% (v/v) TFA

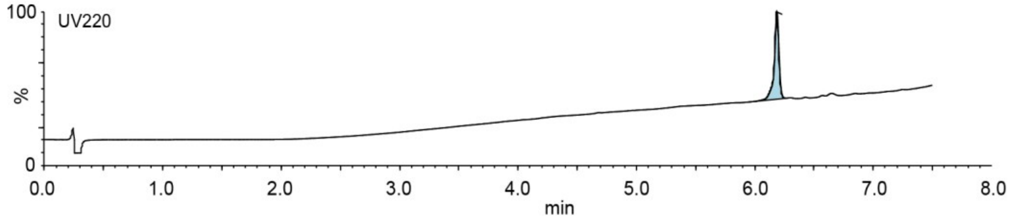

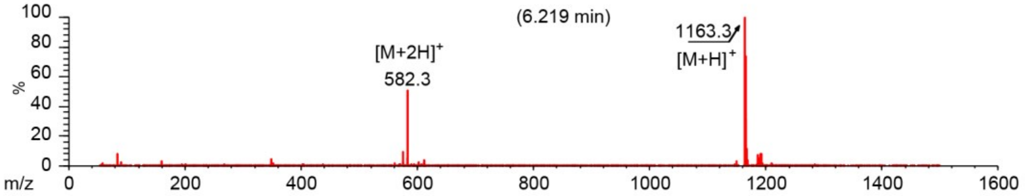

Figure S40: Summary for **9b** synthesis

SSWPARCLHQDLC-TSL1

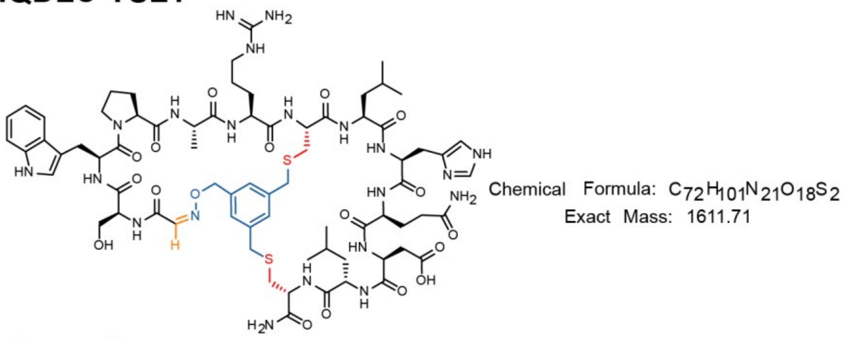

Chemical Formula: $C_{72}H_{101}N_{21}O_{18}S_2$
Exact Mass: 1611.71

Starting material mass = 15 mg
Final product mass = 4.6 mg

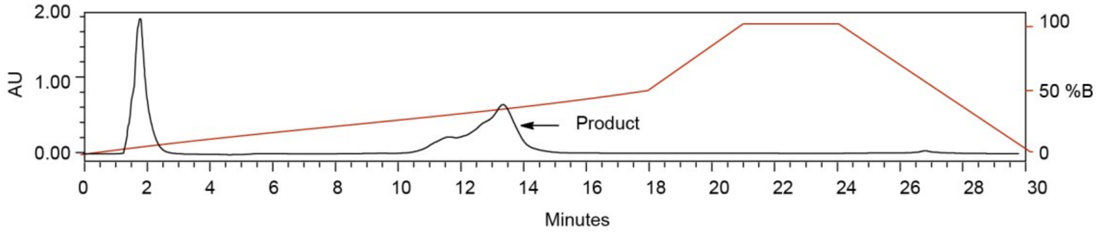

Gradient Table

Time (min)	Solvent B (%)
0	2
2	2
18	50
21	100
24	100
26	2
30	2

Flow rate: 8 mL/min
Symmetry C18 prep column
(100 A, 5 μm, 19 mm X 50 mm)

Solvent A: H_2O + 0.1% (v/v) TFA
Solvent B: CH_3CN + 0.1% (v/v) TFA

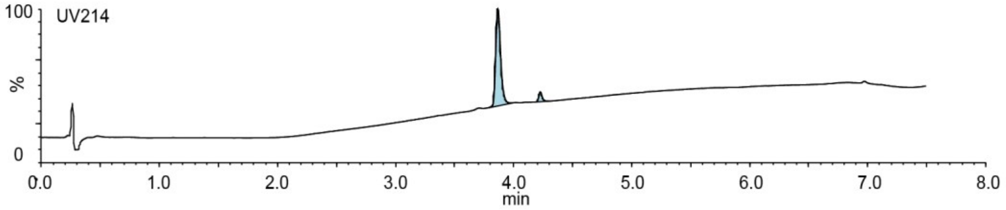

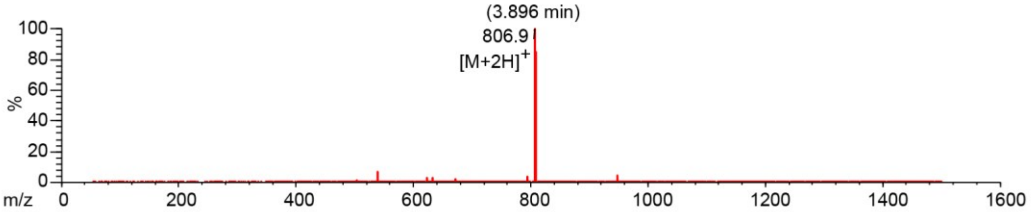

Figure S41: Summary for **13c** synthesis

SNTWNPWCPWDAPLCam-TSL1

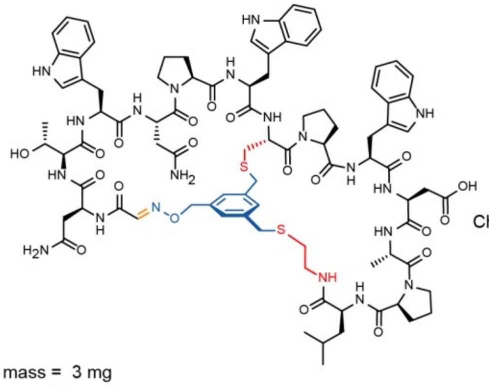

Chemical Formula: $C_{89}H_{110}N_{20}O_{20}S_2$
Exact Mass: 1842.76

Starting material mass = 3 mg
Final product mass = 1.3 mg

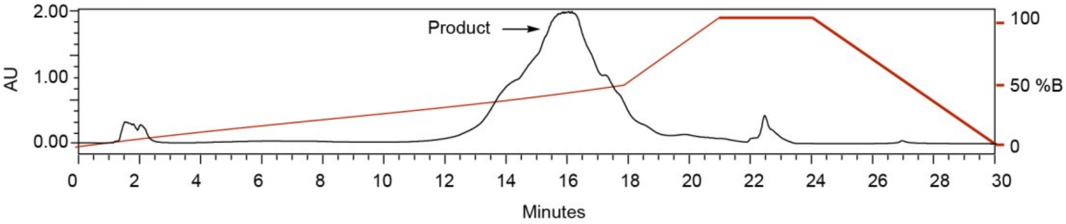

Gradient Table

Time (min)	Solvent B (%)
0	2
2	2
18	50
21	100
24	100
26	2
30	2

Flow rate: 8 mL/min
Symmetry C18 prep column
(100 A, 5 μm, 19 mm X 50 mm)

Solvent A: H_2O + 0.1% (v/v) TFA
Solvent B: CH_3CN + 0.1% (v/v) TFA

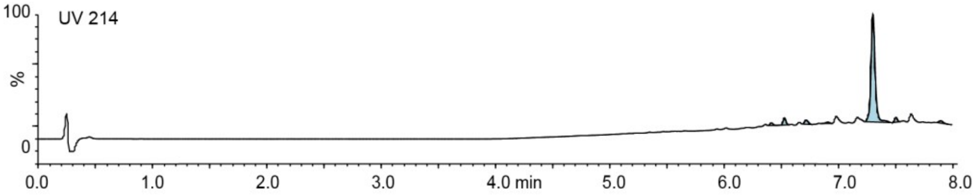

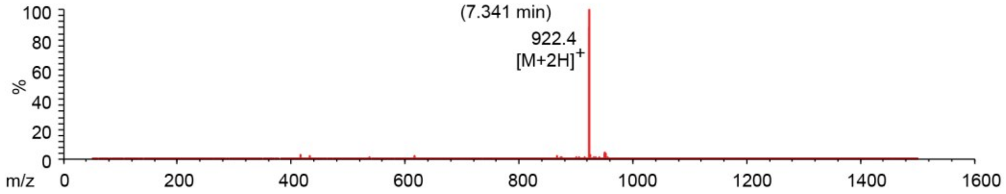

Figure S42: Summary for **12c** synthesis

SPCKGPSATC-TSL6

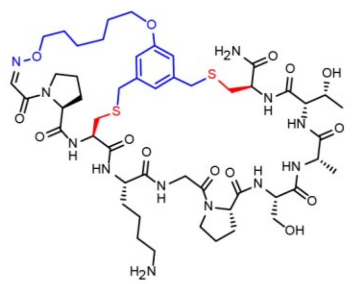

Chemical Formula: $C_{50}H_{76}N_{12}O_{14}S_2$
Exact Mass: 1132.5045

Starting material mass: 10 mg
Final product mass: 1 mg

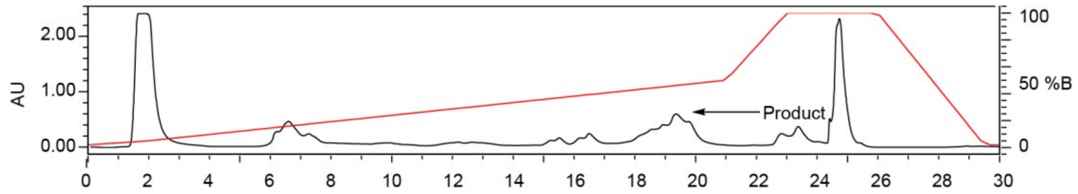

Time (min)	Solvent B (%)
0	2
2	5
21	50
23	100
26	100
29.5	2
30	2

Flow Rate: 8 mL/min
Symmetry C18 Prep Column
(100 Å, 5 µm, 10 mm X 50 mm)

Slovent A: H_2O + 0.1% (v/v) TFA
Slovent B: MeCN + 0.1% (v/v) TFA

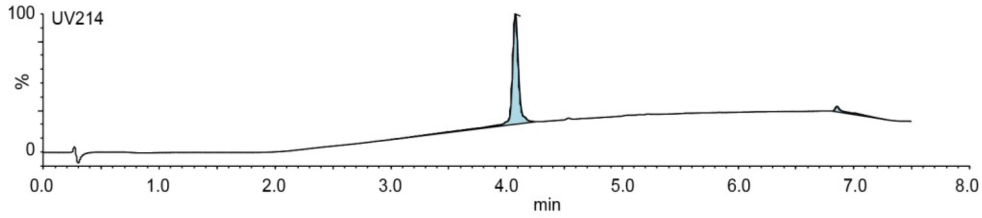

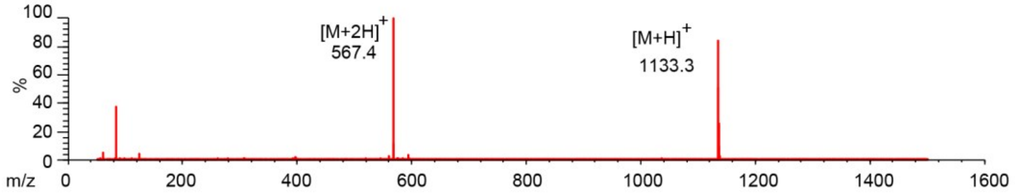

Figure S43: Summary for **15d** synthesis

SPCKGRHHNC-TSL6

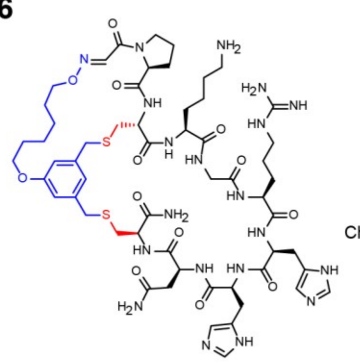

Chemical Formula: $C_{57}H_{84}N_{20}O_{13}S_2$
Exact Mass: 1320.5968

Starting material mass: 6.0 mg
Final product mass: 10.0 mg

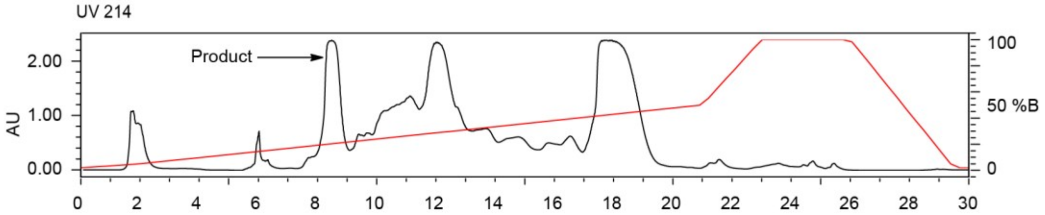

Time (min)	Solvent B (%)
0	2
2	5
21	50
23	100
26	100
29.5	2
30	2

Flow Rate: 8 mL/min
Symmetry C18 Prep Column
(100 Å, 5 µm, 10 mm X 50 mm)

Slovent A: H_2O + 0.1% (v/v) TFA
Slovent B: MeCN + 0.1% (v/v) TFA

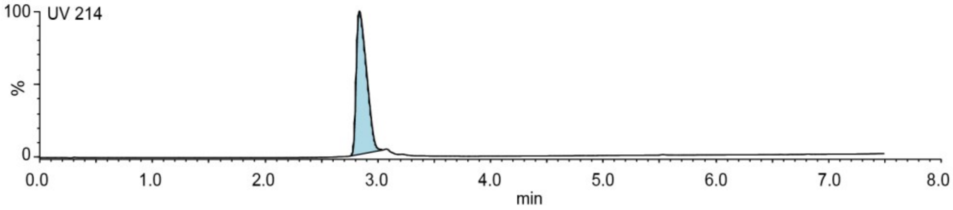

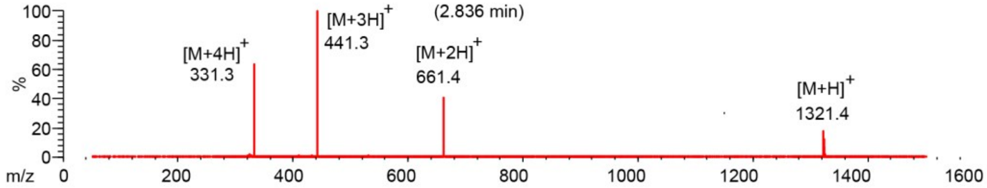

Figure S44: Summary for **16b** synthesis

SPCQRGHMFC-TSL6

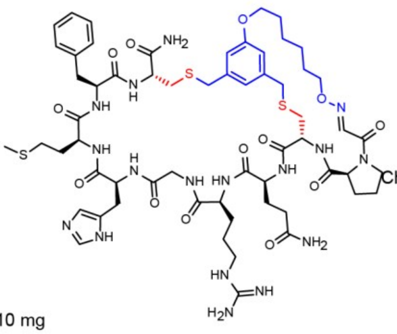

Chemical Formula: $C_{60}H_{85}N_{17}O_{13}S_3$
Exact Mass: 1347.57

Starting material mass: 10 mg
Final product mass: 1 mg

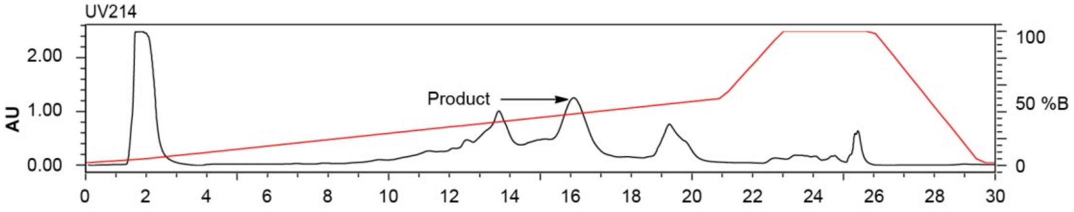

Time (min)	Solvent B (%)
0	2
2	5
21	50
23	100
26	100
29.5	2
30	2

Flow Rate: 8 mL/min
Symmetry C18 Prep Column
(100 Å, 5 µm, 10 mm X 50 mm)

Slovent A: H_2O + 0.1% (v/v) TFA
Slovent B: MeCN + 0.1% (v/v) TFA

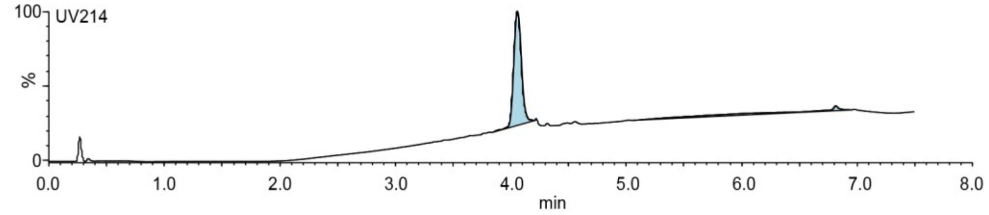

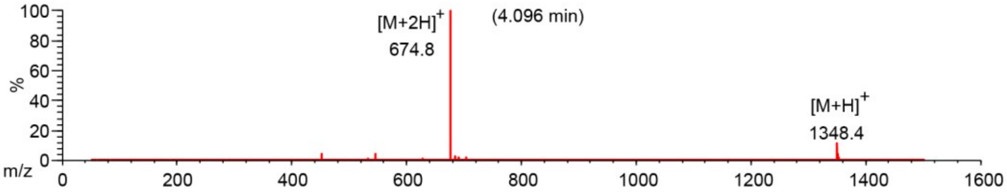

Figure S45: Summary for **18b** synthesis

SYCKRAHKNC-TSL6

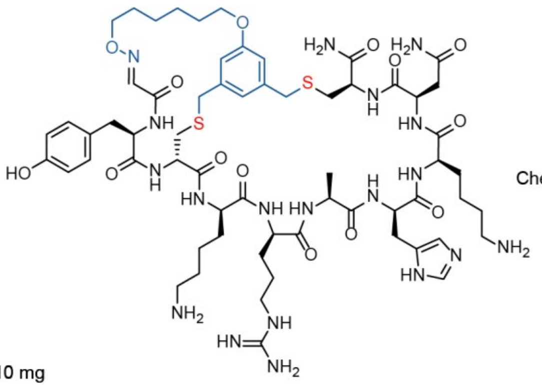

Chemical Formula: $C_{62}H_{93}N_{19}O_{14}S_2$
Exact Mass: 1391.66
Molecular Weight: 1392.67

Starting material mass :10 mg
Final product mass: 1.6 mg

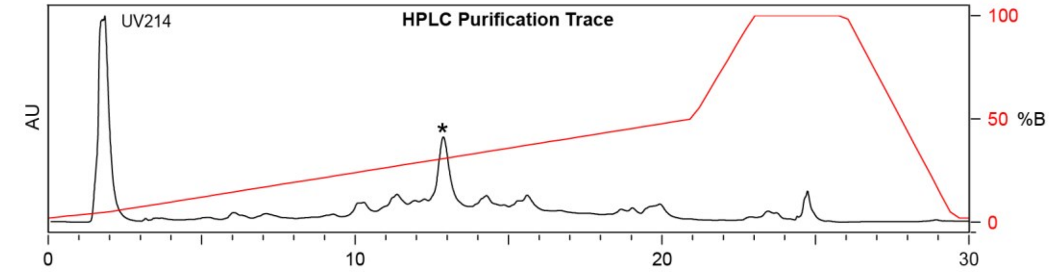

Time (min)	Solvent B (%)
0	2
2	5
21	50
23	100
26	100
29.5	2
30	2

Flow Rate: 8 mL/min
Symmetry C18 Prep Column
(100 Å, 5 μm, 10 mm X 50 mm)

Slovent A: H_2O + 0.1% (v/v) TFA
Slovent B: MeCN + 0.1% (v/v) TFA

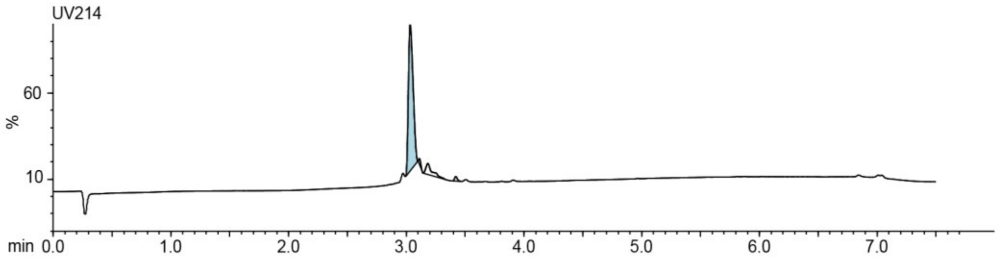

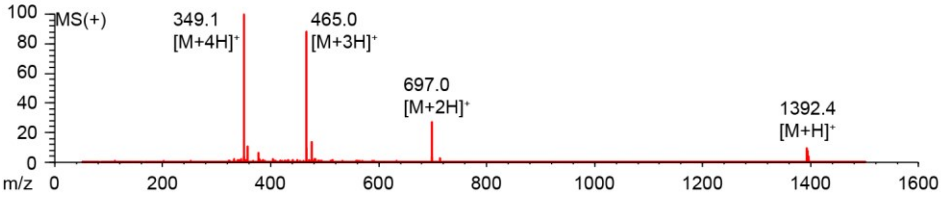

Figure S46: Summary for **19b** synthesis

SWCRGHDRTC-TSL6

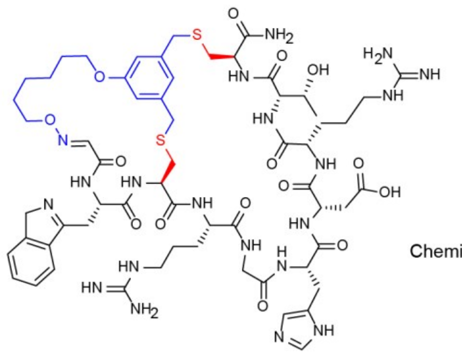

Starting material mass: 10 mg
Final product mass: 0.7 mg

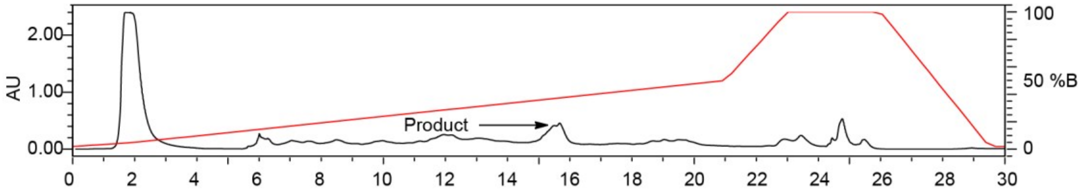

Time (min)	Solvent B (%)
0	2
2	5
21	50
23	100
26	100
29.5	2
30	2

Flow Rate: 8 mL/min
Symmetry C18 Prep Column
(100 Å, 5 µm, 10 mm X 50 mm)

Slovent A: H_2O + 0.1% (v/v) TFA
Slovent B: MeCN + 0.1% (v/v) TFA

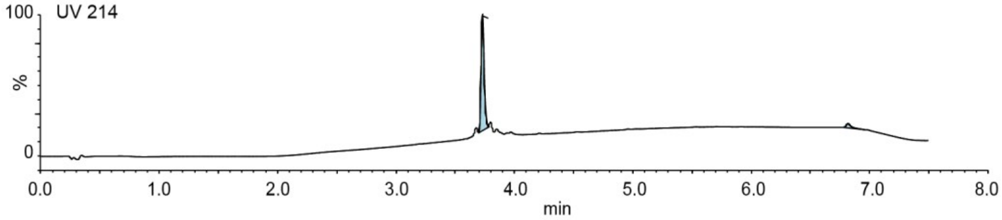

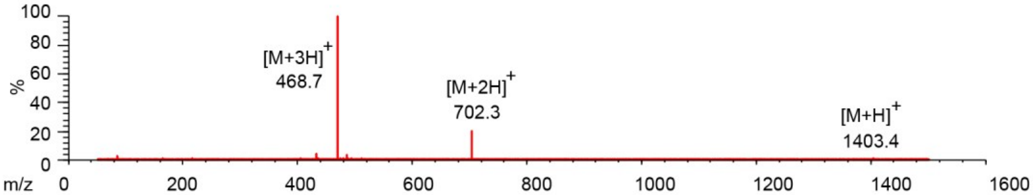

Figure S47: Summary for **21b** synthesis

SPCAKGMNYC-TSL6

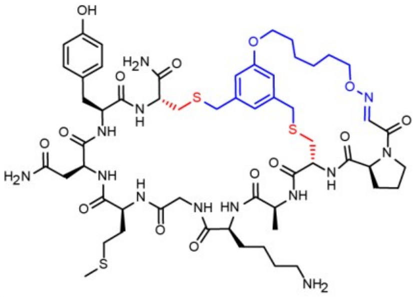

Chemical Formula: $C_{56}H_{81}N_{13}O_{14}S_3$
Exact Mass: 1255.5188

Starting material mass: 10 mg
Final product mass: 0.7 mg

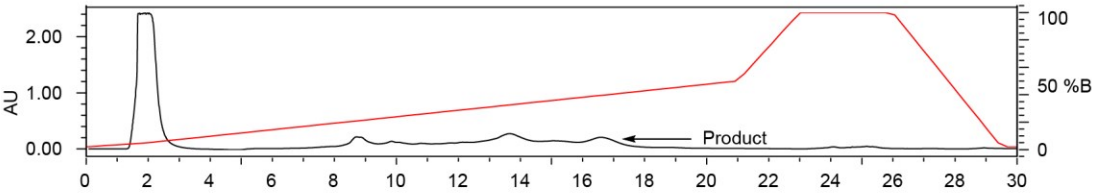

Time (min)	Solvent B (%)
0	2
2	5
21	50
23	100
26	100
29.5	2
30	2

Flow Rate: 8 mL/min
Symmetry C18 Prep Column
(100 Å, 5 µm, 10 mm X 50 mm)

Slovent A: H_2O + 0.1% (v/v) TFA
Slovent B: MeCN + 0.1% (v/v) TFA

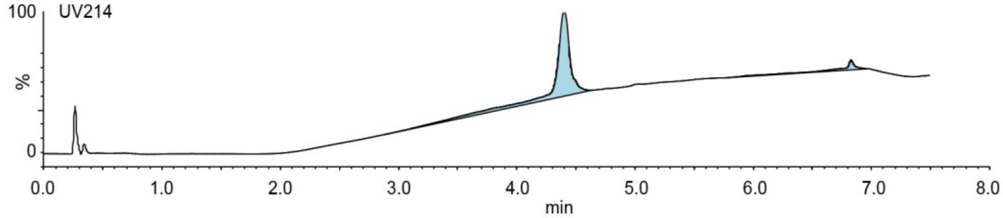

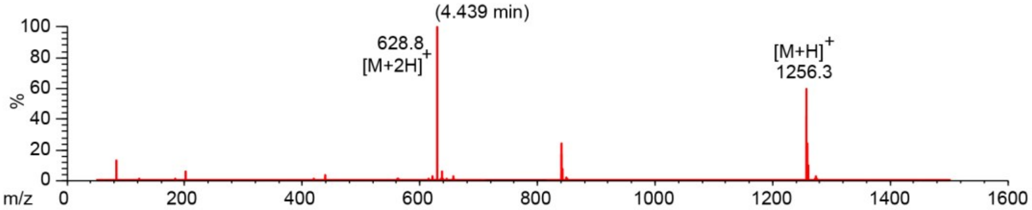

Figure S48: Summary for **22b** synthesis

6.2. *Summary of* ***PFS*** *Peptides Synthesis*

STCQGECGGG-PFS

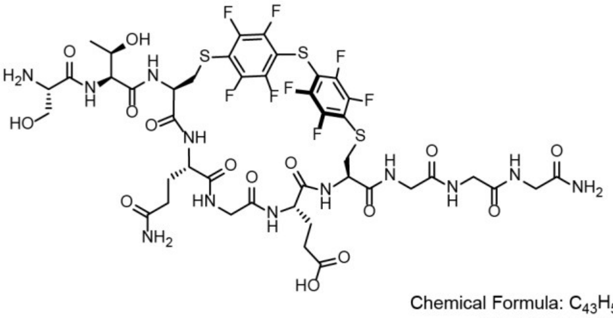

Chemical Formula: $C_{43}H_{50}F_8N_{12}O_{15}S_3$
Exact Mass: 1222.26
Molecular Weight: 1223.11

Starting material mass : 9.7 mg
Final product mass: 5.0 mg

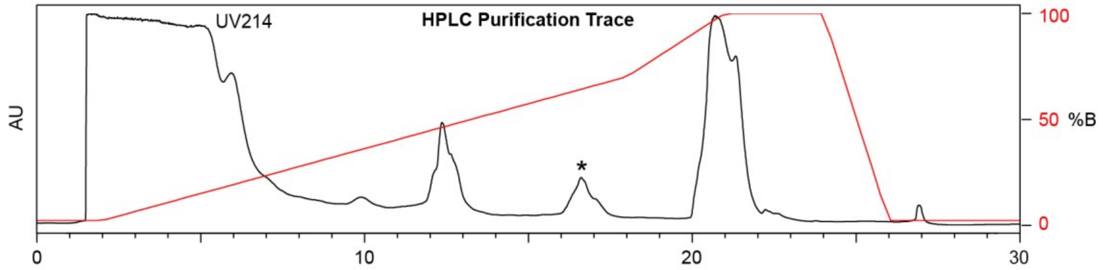

Time (min)	Solvent B %
0	2
2	2
18	70
21	100
24	100
26	0
30	0

Flow Rate: 8 mL/min
Symmetry C18 Prep Column
(100 Å, 5 μm, 10 mm X 50 mm)

Slovent A: H_2O + 0.1% (v/v) TFA
Slovent B: MeCN + 0.1% (v/v) TFA

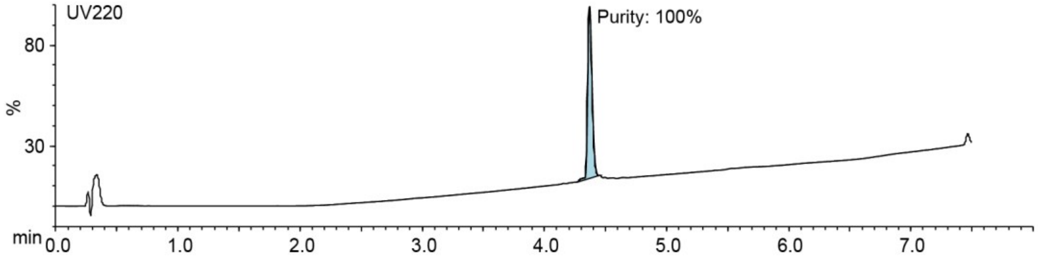

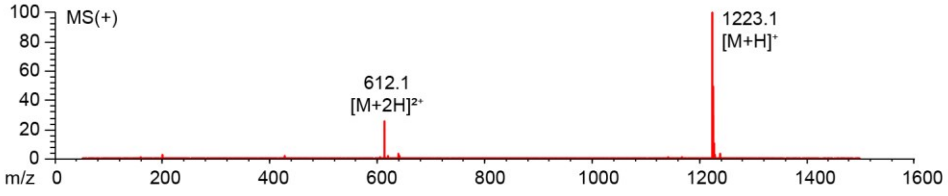

Figure S49: Summary for **4e** synthesis

SICRFFCGGG-PFS

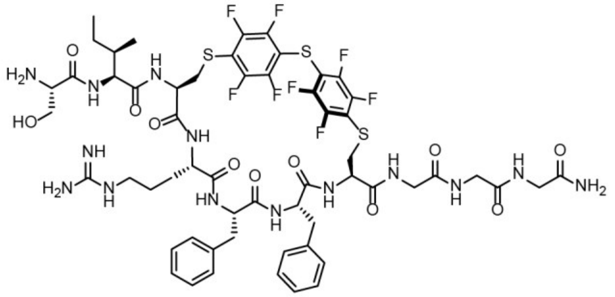

Starting material mass : 5.5 mg
Final product mass: 3.2 mg

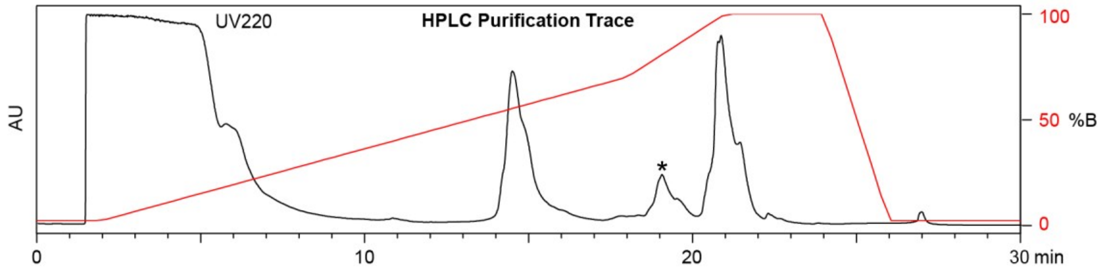

Time (min)	Solvent B (%)
0	2
2	2
18	70
21	100
24	100
26	0
30	0

Flow Rate: 8 mL/min
Symmetry C18 Prep Column
(100 Å, 5 μm, 10 mm X 50 mm)

Slovent A: H_2O + 0.1% (v/v) TFA
Slovent B: MeCN + 0.1% (v/v) TFA

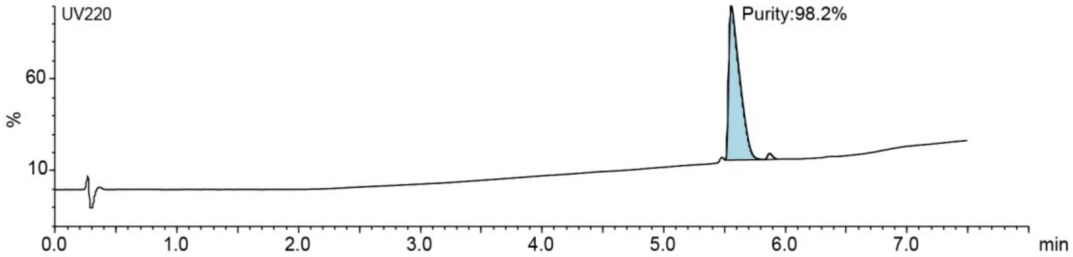

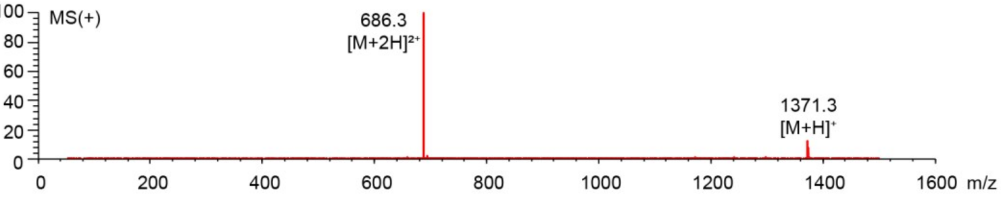

Figure S50: Summary for **5e** synthesis

6.3. Summary of **DBMB** Peptides Synthesis

SHCDYYC-DBMB

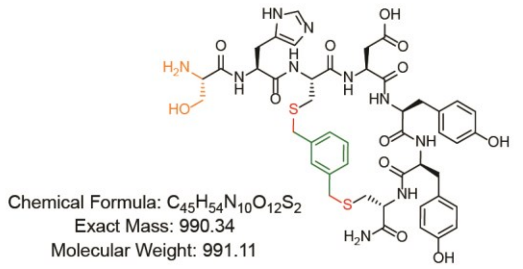

Starting material mass :10 mg
Final product mass: 5.2 mg

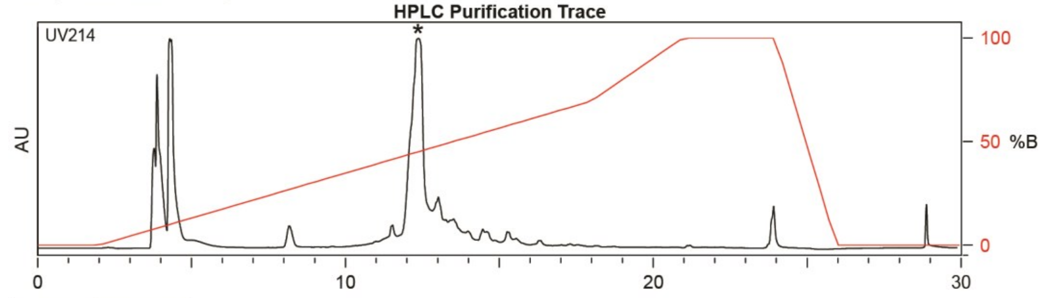

Time (min)	Solvent B (%)
0	2
2	2
18	70
21	100
24	100
26	2
29.5	2
30	2

Flow Rate: 13 mL/min
Phenomenex Kinetex EVO C18 Prep Column
(100 Å, 5 μm, 21.5 mm X 250 mm)

Slovent A: H_2O + 0.1% (v/v) TFA
Slovent B: MeCN + 0.1% (v/v) TFA

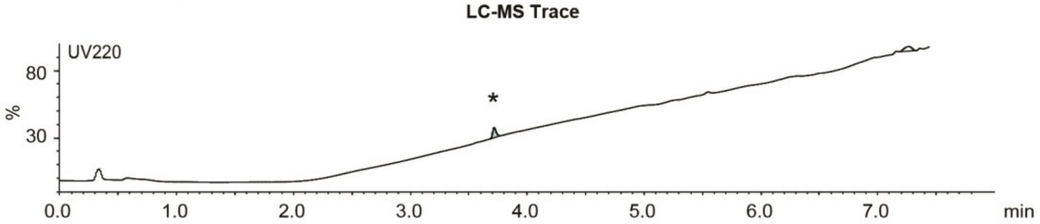

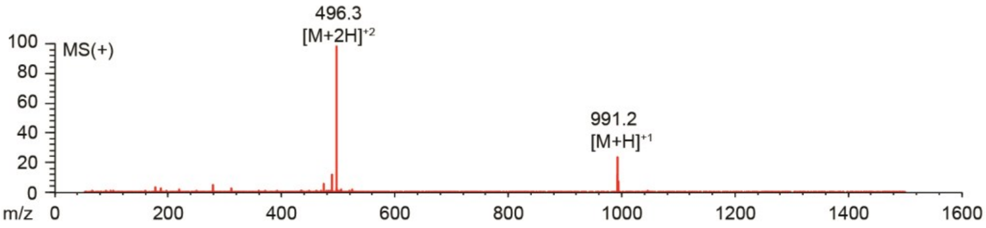

Figure S51: Summary for **1g** synthesis

SYCKADC-DBMB

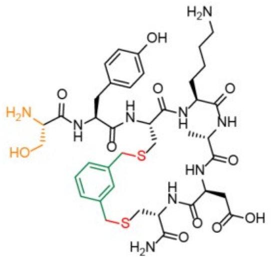

Chemical Formula: $C_{39}H_{55}N_9O_{11}S_2$
Exact Mass: 889.35
Molecular Weight: 890.04

Starting material mass: 10 mg
Final product mass: 5.3 mg

HPLC Purification Trace

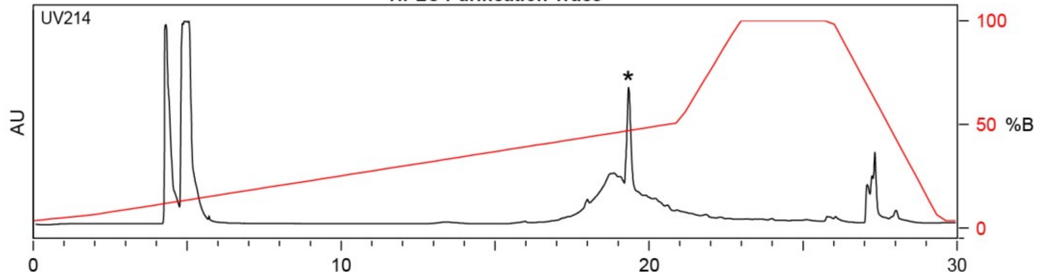

Time (min)	Solvent B (%)
0	2
2	2
21	50
23	100
26	100
29.5	2
30	2

Flow Rate: 13 mL / min
Phenomenex Kinetex EVO C18 Prep Column
(100 Å, 5 µm, 21.5 mm X 250 mm)

Slovent A: H_2O + 0.1% (v/v) TFA
Slovent B: MeCN + 0.1% (v/v) TFA

LCMS Trace

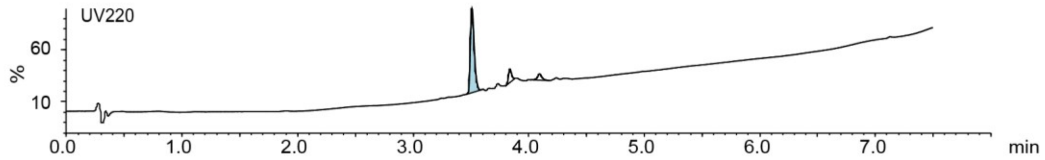

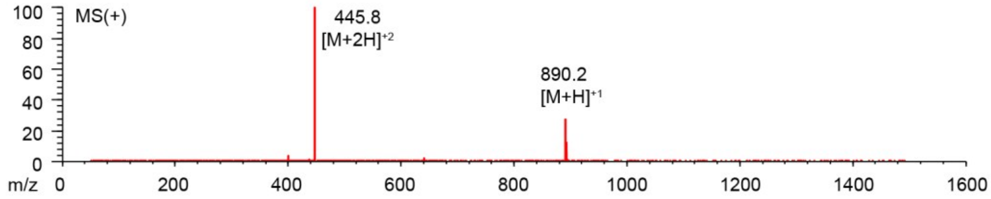

Figure S52: Summary for **2g** synthesis

SYCKRFC-DBMB

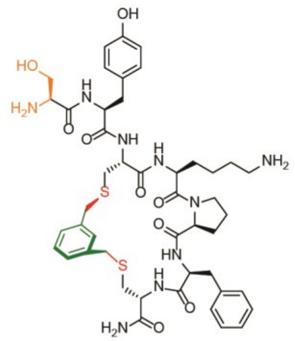

Chemical Formula: $C_{46}H_{61}N_9O_9S_2$
Exact Mass: 947.40
Molecular Weight: 948.17

Starting material mass :10 mg
Final product mass: 7.0 mg

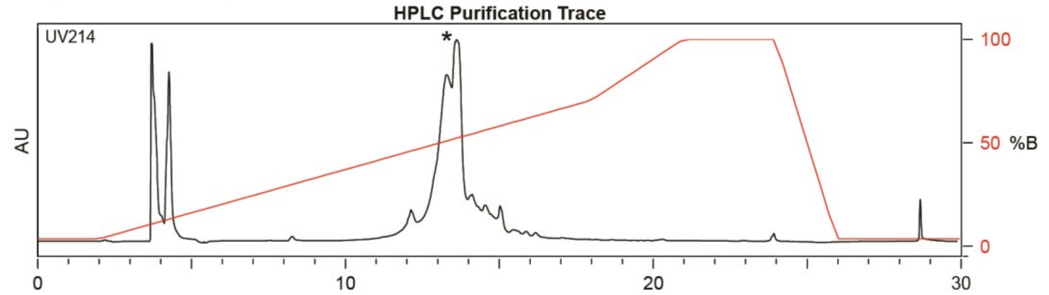

Time (min)	Solvent B (%)
0	2
2	2
18	70
21	100
24	100
26	2
29.5	2
30	2

Flow Rate: 13 mL/min
Phenomenex Kinetex EVO C18 Prep Column
(100 Å, 5 μm, 21.5 mm X 250 mm)

Slovent A: H_2O + 0.1% (v/v) TFA
Slovent B: MeCN + 0.1% (v/v) TFA

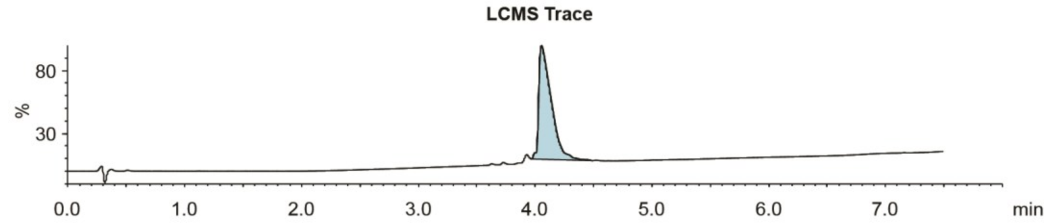

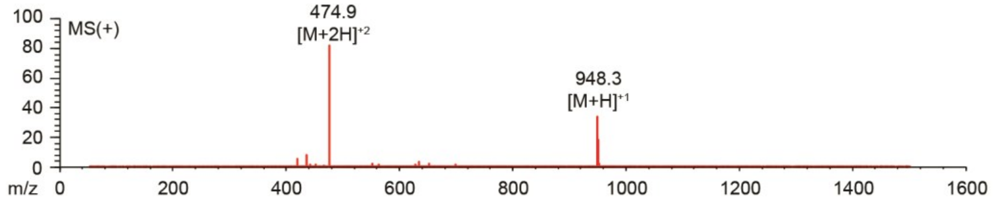

Figure S53: Summary for **3g** synthesis

STCQGECGGG-DBMB

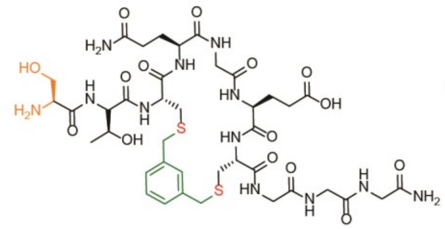

Chemical Formula: $C_{39}H_{58}N_{12}O_{15}S_2$
Exact Mass: 998.36
Molecular Weight: 999.08

Starting material mass :10 mg
Final product mass: 1.7 mg

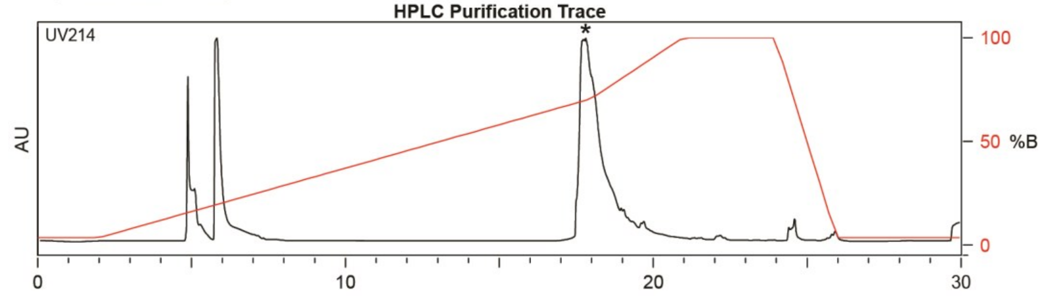

Time (min)	Solvent B (%)
0	2
2	2
18	70
21	100
24	100
26	2
29.5	2
30	2

Flow Rate: 13 mL / min
Phenomenex Kinetex EVO C18 Prep Column
(100 Å, 5 μm, 21.5 mm X 250 mm)

Slovent A: H_2O + 0.1% (v/v) TFA
Slovent B: MeCN + 0.1% (v/v) TFA

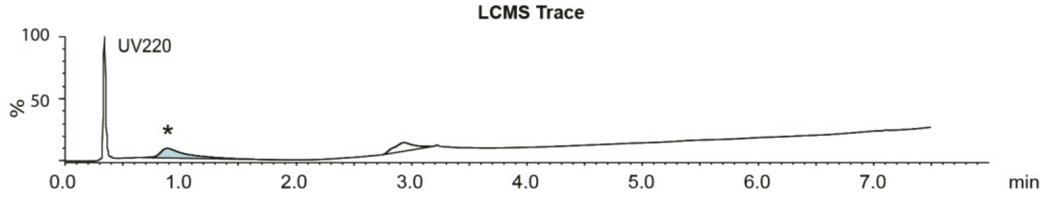

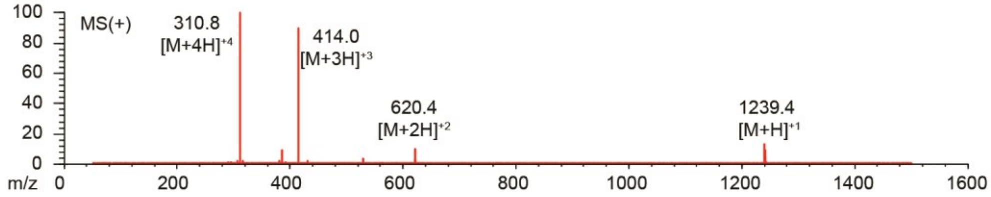

Figure S54: Summary for **4g** synthesis

SICRFFCGGG-DBMB

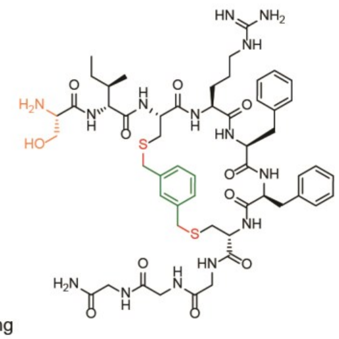

Chemical Formula: $C_{53}H_{74}N_{14}O_{11}S_2$
Exact Mass: 1146.51
Molecular Weight: 1147.38

Starting material mass: 10 mg
Final product mass: 5.1 mg

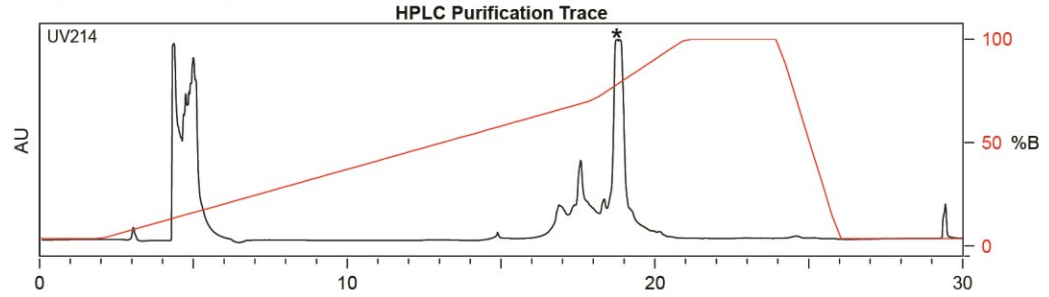

Time (min)	Solvent B (%)
0	2
2	2
18	70
21	100
24	100
26	2
29.5	2
30	2

Flow Rate: 13 mL/min
Phenomenex Kinetex EVO C18 Prep Column
(100 Å, 5 µm, 21.5 mm X 250 mm)

Slovent A: H_2O + 0.1% (v/v) TFA
Slovent B: MeCN + 0.1% (v/v) TFA

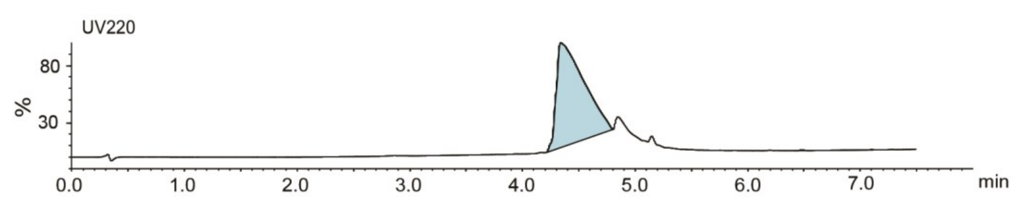

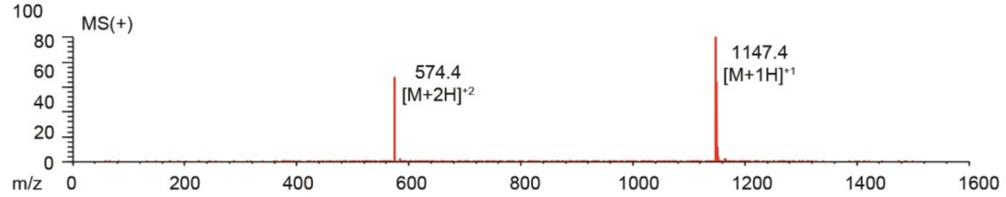

Figure S55: Summary for **5g** synthesis

SHDCYLEC-DBMB

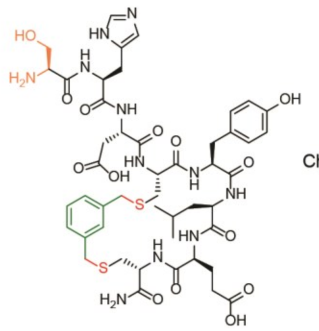

Chemical Formula: $C_{47}H_{63}N_{11}O_{14}S_2$
Exact Mass: 1069.40
Molecular Weight: 1070.20

Starting material mass :10 mg
Final product mass: 3.0 mg

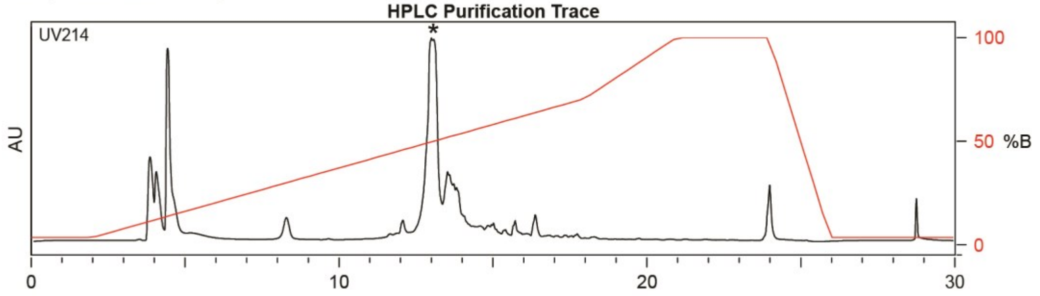

Time (min)	Solvent B (%)
0	2
2	2
18	70
21	100
24	100
26	2
29.5	2
30	2

Flow Rate: 13 mL/min
Phenomenex Kinetex EVO C18 Prep Column
(100 Å, 5 μm, 21.5 mm X 250 mm)

Slovent A: H_2O + 0.1% (v/v) TFA
Slovent B: MeCN + 0.1% (v/v) TFA

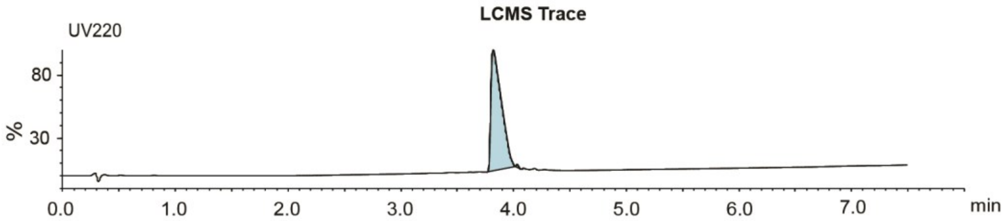

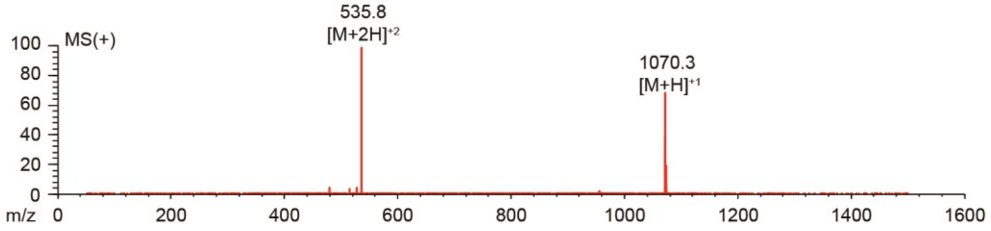

Figure S56: Summary for **6g** synthesis

SWDYRECYLEC-DBMB

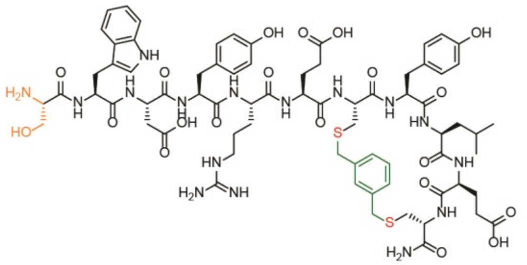

Chemical Formula: $C_{72}H_{94}N_{16}O_{20}S_2$
Exact Mass: 1566.63
Molecular Weight: 1567.76

Starting material mass:10 mg
Final product mass: 1.3 mg

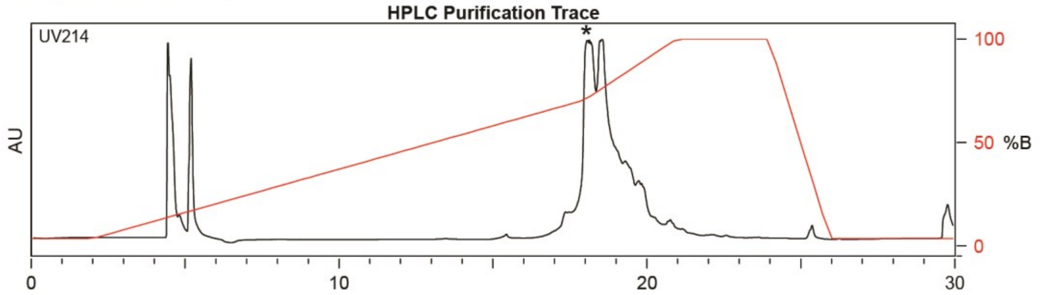

Time (min)	Solvent B (%)
0	2
2	2
18	70
21	100
24	100
26	2
29.5	2
30	2

Flow Rate: 13 mL/min
Phenomenex Kinetex EVO C18 Prep Column
(100 Å, 5 µm, 21.5 mm X 250 mm)

Slovent A: H_2O + 0.1% (v/v) TFA
Slovent B: MeCN + 0.1% (v/v) TFA

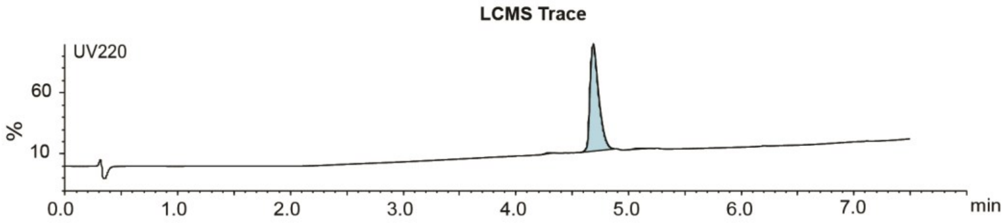

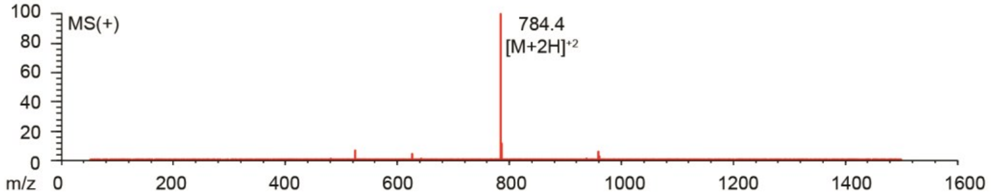

Figure S57: Summary for **7g** synthesis

SHCVWWDC-DBMB

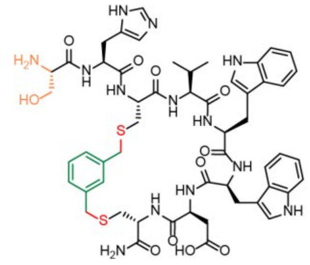

Chemical Formula: $C_{54}H_{65}N_{13}O_{11}S_2$
Exact Mass: 1135.44
Molecular Weight: 1136.31

Starting material mass: 20.0 mg
Final product mass: 3.6 mg

HPLC Purification Trace

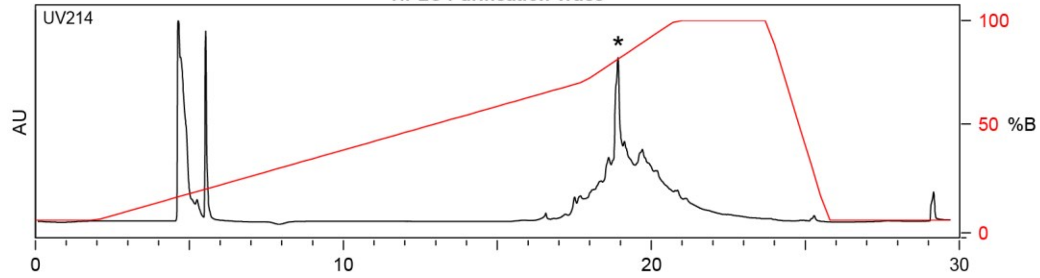

Time (min)	Solvent B (%)
0	2
2	2
18	70
21	100
24	100
26	2
29.5	2
30	2

Flow Rate: 13 mL/min
Phenomenex Kinetex EVO C18 Prep Column
(100 Å, 5 µm, 21.5 mm X 250 mm)

Slovent A: H_2O + 0.1% (v/v) TFA
Slovent B: MeCN + 0.1% (v/v) TFA

LCMS Trace

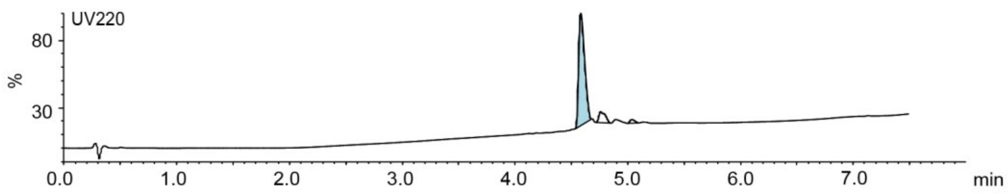

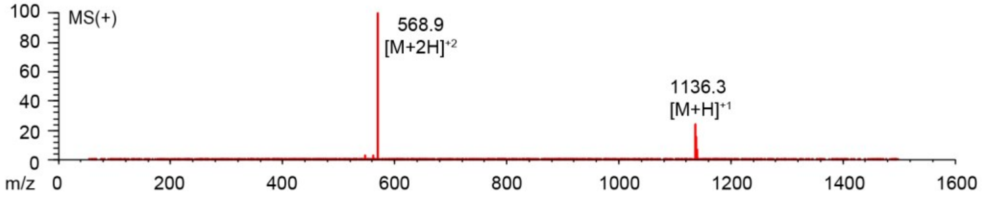

Figure S58: Summary for **8g** synthesis

SFCDWYGC-DBMB

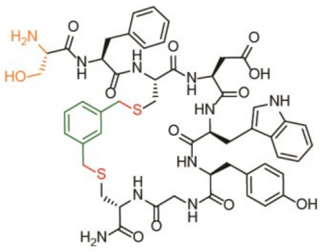

Chemical Formula: $C_{52}H_{60}N_{10}O_{12}S_2$
Exact Mass: 1080.38
Molecular Weight: 1081.23

Starting material mass :10 mg
Final product mass: 1.1 mg

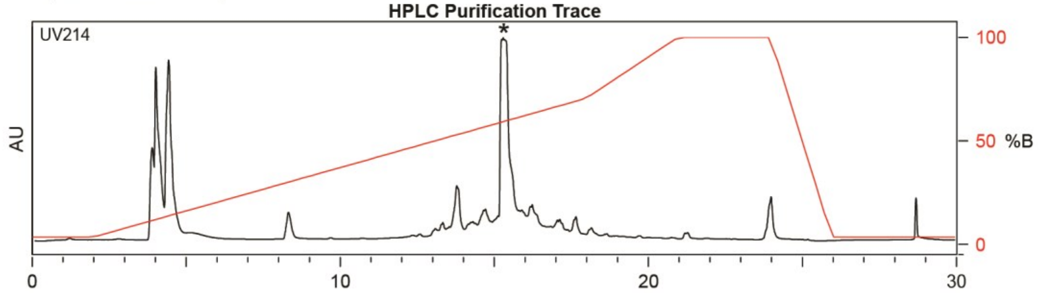

Time (min)	Solvent B (%)
0	2
2	2
18	70
21	100
24	100
26	2
29.5	2
30	2

Flow Rate: 13 mL/min
Phenomenex Kinetex EVO C18 Prep Column
(100 Å, 5 µm, 21.5 mm X 250 mm)

Slovent A: H_2O + 0.1% (v/v) TFA
Slovent B: MeCN + 0.1% (v/v) TFA

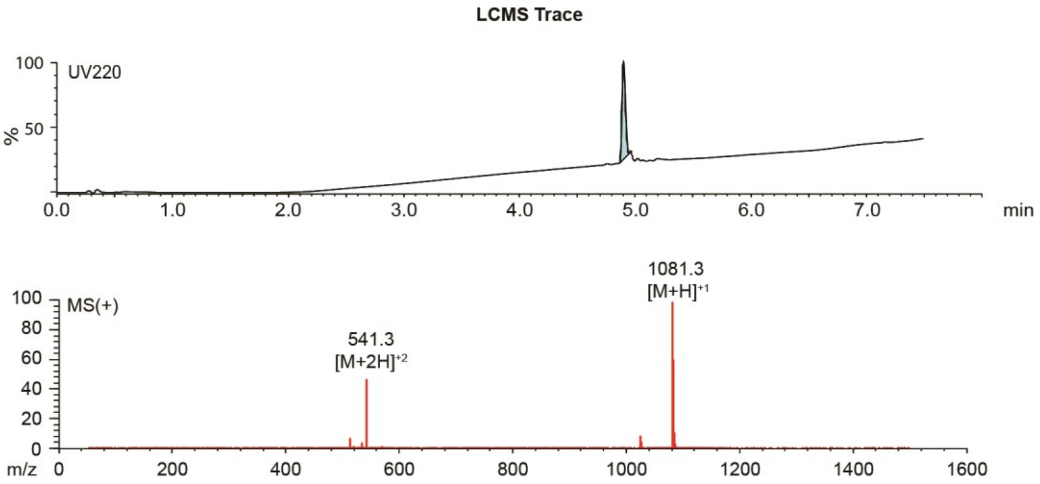

Figure S59: Summary for **9g** synthesis

SSWPARCLHQDLC-DBMB

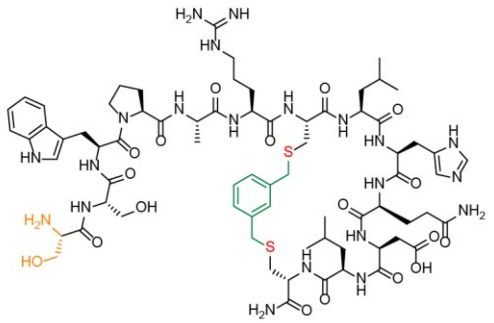

Chemical Formula: $C_{72}H_{105}N_{21}O_{18}S_2$
Exact Mass: 1615.74
Molecular Weight: 1616.88

Starting material mass :10 mg
Final product mass: 4.9 mg

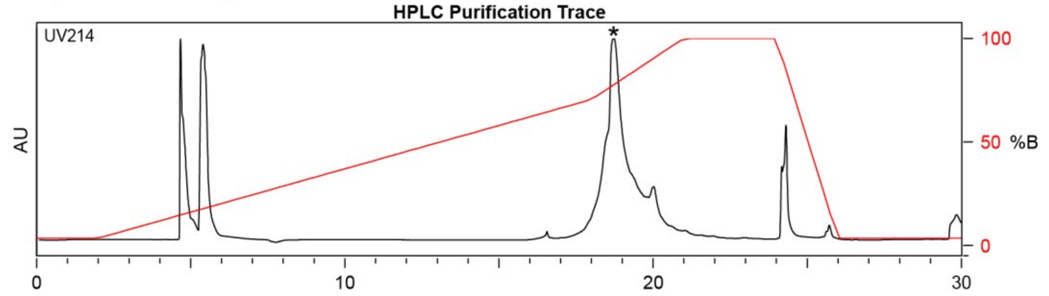

Time (min)	Solvent B (%)
0	2
2	2
18	70
21	100
24	100
26	2
29.5	2
30	2

Flow Rate: 13 mL / min
Phenomenex Kinetex EVO C18 Prep Column
(100 Å, 5 µm, 21.5 mm X 250 mm)

Slovent A: H_2O + 0.1% (v/v) TFA
Slovent B: MeCN + 0.1% (v/v) TFA

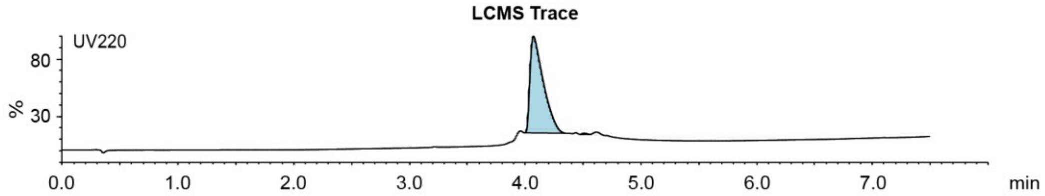

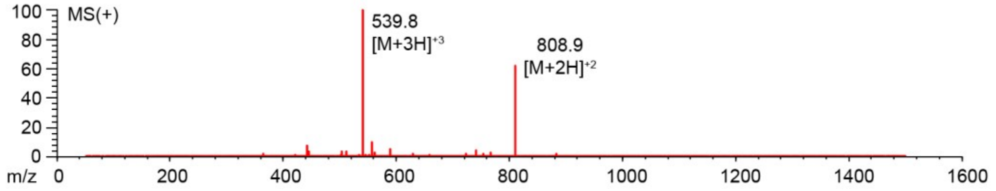

Figure S60: Summary for **12g** synthesis

SPCKAGTGQC-DBMB

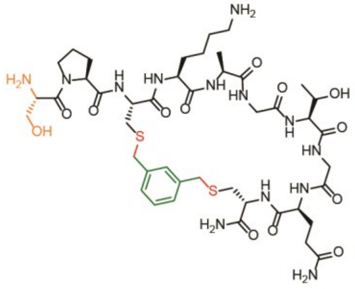

Chemical Formula: $C_{44}H_{69}N_{13}O_{13}S_2$
Exact Mass: 1051.46
Molecular Weight: 1052.23

Starting material mass: 10.0 mg
Final product mass: 1.3 mg

HPLC Purification Trace

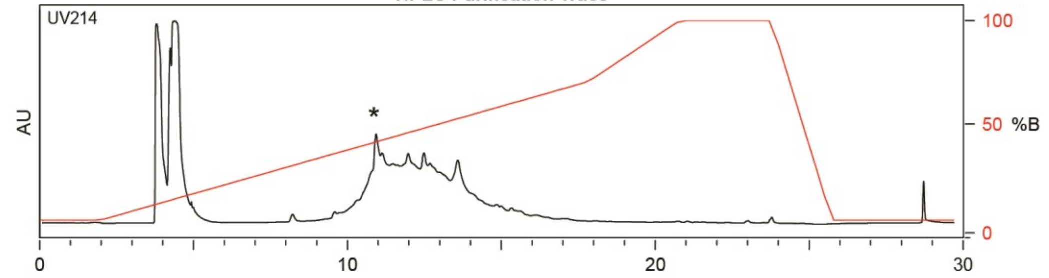

Time (min)	Solvent B (%)
0	2
2	2
18	70
21	100
24	100
26	2
29.5	2
30	2

Flow Rate: 13 mL/min
Phenomenex Kinetex EVO C18 Prep Column
(100 Å, 5 µm, 21.5 mm X 250 mm)

Slovent A: H_2O + 0.1% (v/v) TFA
Slovent B: MeCN + 0.1% (v/v) TFA

LCMS Trace

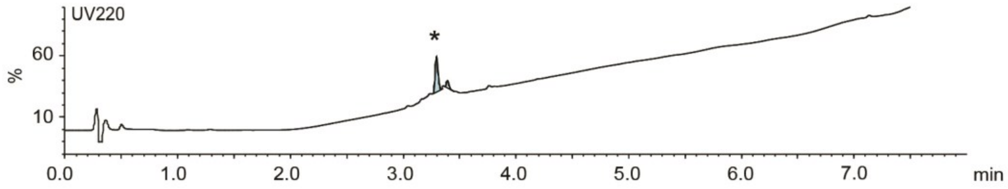

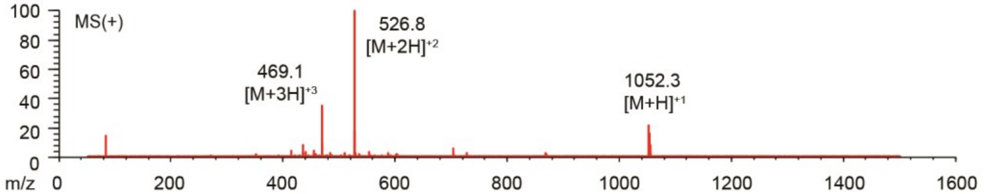

Figure S61: Summary for **14g** synthesis

SPCKGPSATC-DBMB

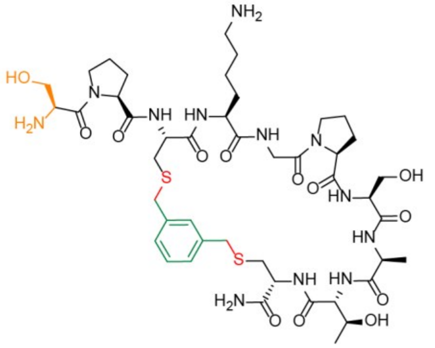

Chemical Formula: $C_{45}H_{70}N_{12}O_{13}S_2$
Exact Mass: 1050.46
Molecular Weight: 1051.25

Starting material mass: 5 mg
Final product mass: 0.5 mg

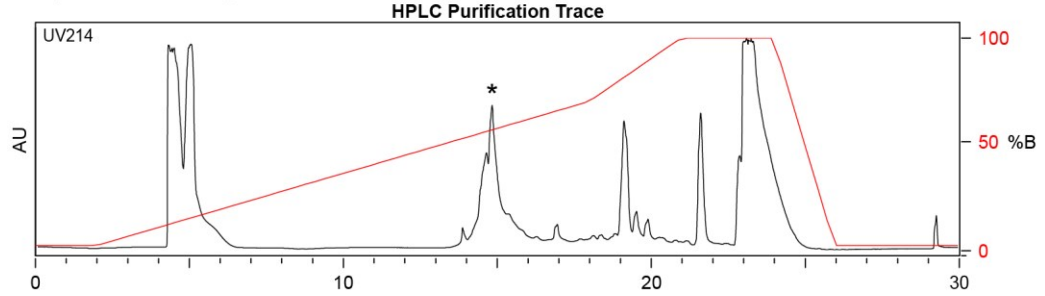

Time (min)	Solvent B (%)
0	2
2	2
18	70
21	100
24	100
26	2
29.5	2
30	2

Flow Rate: 13 mL/min
Phenomenex Kinetex EVO C18 Prep Column
(100 Å, 5 µm, 21.5 mm X 250 mm)

Slovent A: H_2O + 0.1% (v/v) TFA
Slovent B: MeCN + 0.1% (v/v) TFA

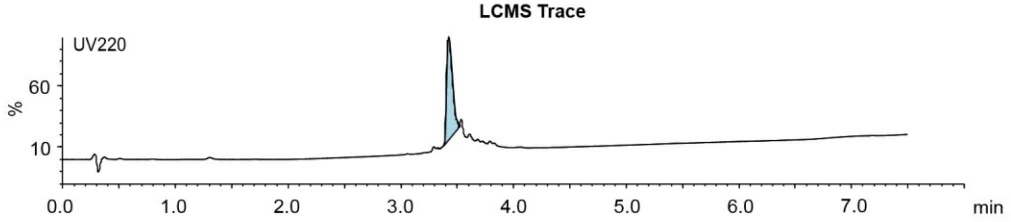

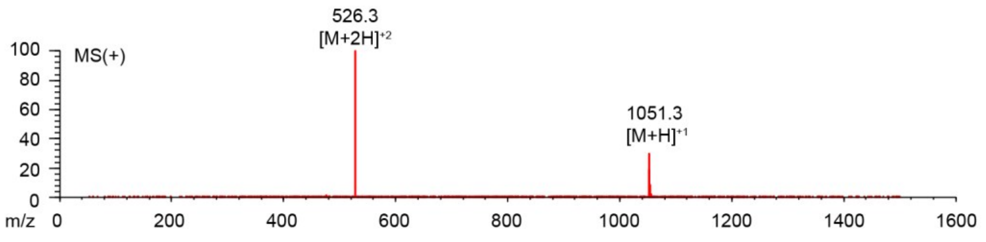

Figure S62: Summary for **15g** synthesis

SPCKGRHHNC-DBMB

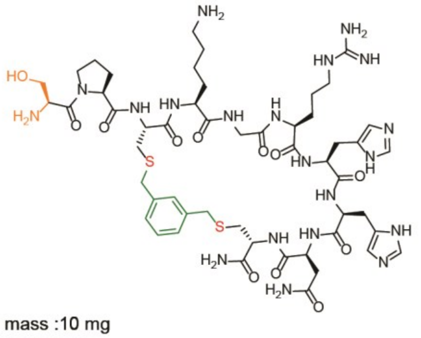

Chemical Formula: $C_{52}H_{78}N_{20}O_{12}S_2$
Exact Mass: 1238.55
Molecular Weight: 1239.44

Starting material mass :10 mg
Final product mass: 6.8 mg

HPLC Purification Trace

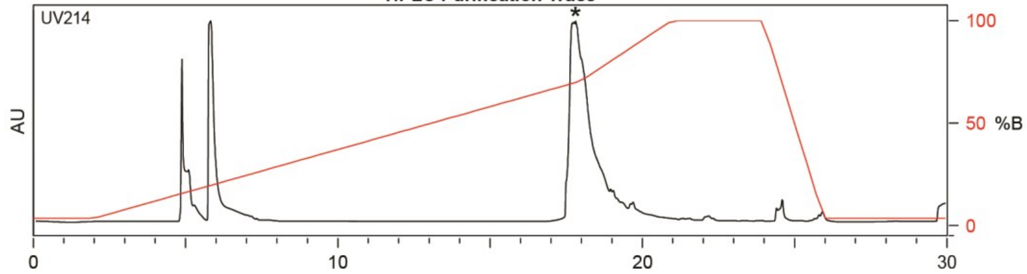

Time (min)	Solvent B (%)
0	2
2	2
18	70
21	100
24	100
26	2
29.5	2
30	2

Flow Rate: 13 mL / min
Phenomenex Kinetex EVO C18 Prep Column
(100 Å, 5 μm, 21.5 mm X 250 mm)

Slovent A: H_2O + 0.1% (v/v) TFA
Slovent B: MeCN + 0.1% (v/v) TFA

LCMS Trace

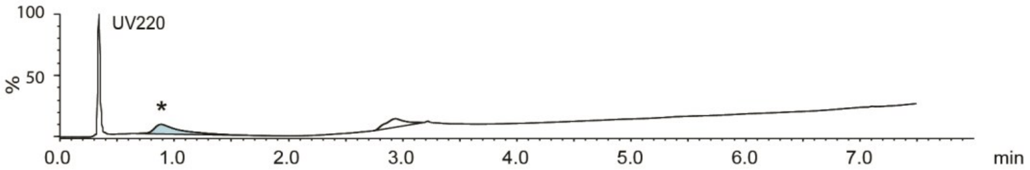

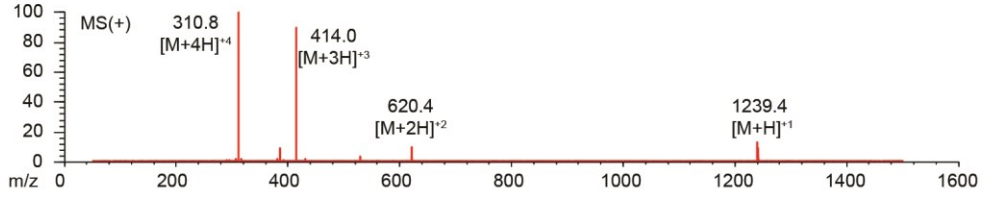

Figure S63: Summary for **16g** synthesis

SYCKRAHKNC-DBMB

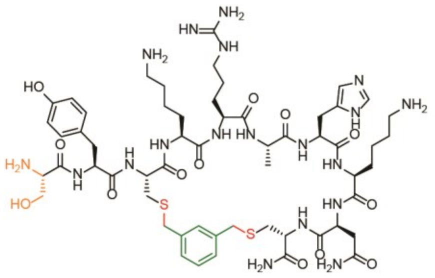

Chemical Formula: $C_{57}H_{87}N_{19}O_{13}S_2$
Exact Mass: 1309.62
Molecular Weight: 1310.56

Starting material mass : 4 mg
Final product mass: 2.3 mg

HPLC Purification Trace

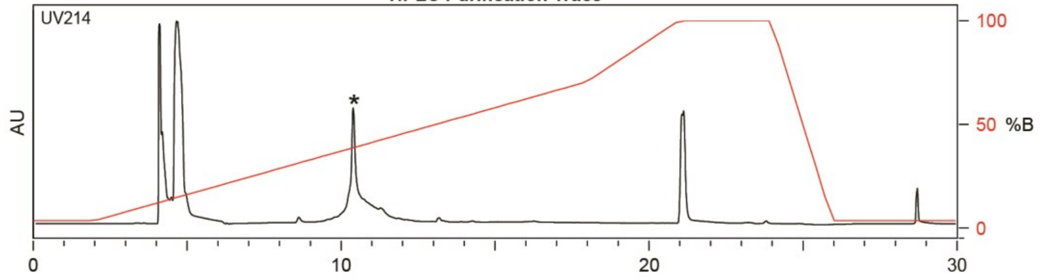

Time (min)	Solvent B (%)
0	2
2	2
18	70
21	100
24	100
26	2
29.5	2
30	2

Flow Rate: 13 mL / min
Phenomenex Kinetex EVO C18 Prep Column
(100 Å, 5 µm, 21.5 mm X 250 mm)

Slovent A: H_2O + 0.1% (v/v) TFA
Slovent B: MeCN + 0.1% (v/v) TFA

LCMS Trace

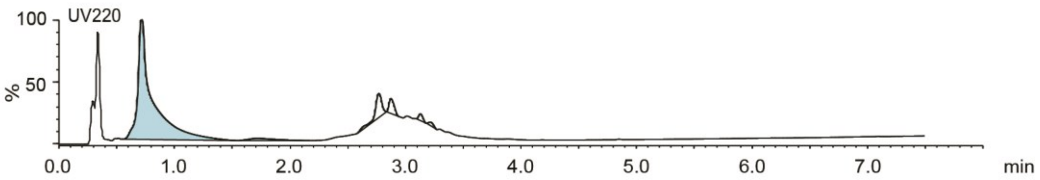

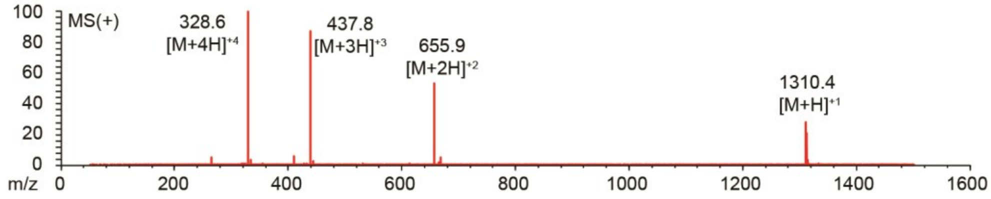

Figure S64: Summary for **19g** synthesis

SQCKRAHAEC-DBMB

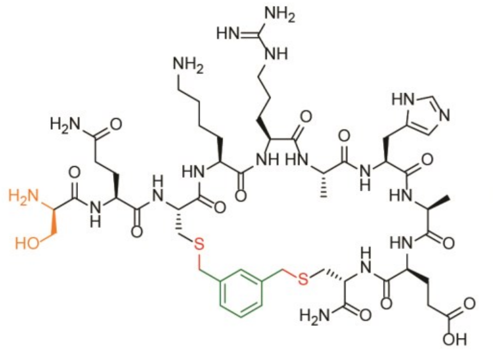

Chemical Formula: $C_{51}H_{80}N_{18}O_{14}S_2$
Exact Mass: 1232.55
Molecular Weight: 1233.43

Starting material mass: 5.0 mg
Final product mass: 0.5 mg

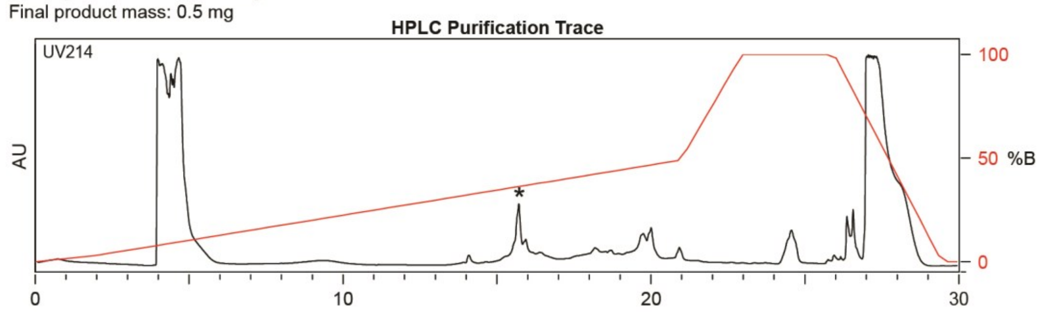

Time (min)	Solvent B (%)
0	2
2	2
18	50
21	100
24	100
26	2
29.5	2
30	2

Flow Rate: 13 mL/min
Phenomenex Kinetex EVO C18 Prep Column
(100 Å, 5 µm, 21.5 mm X 250 mm)

Slovent A: H_2O + 0.1% (v/v) TFA
Slovent B: MeCN + 0.1% (v/v) TFA

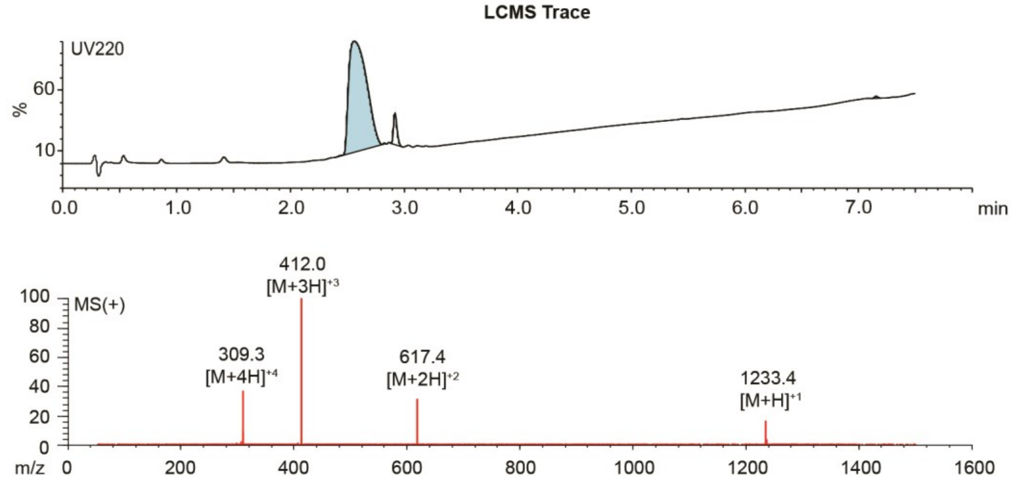

Figure S65: Summary for **20g** synthesis

SPCAKGMNYC-MBX

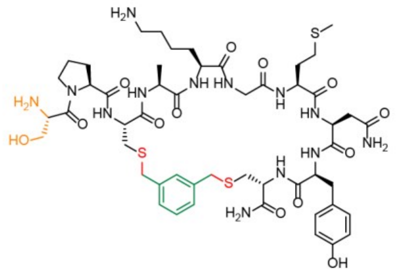

Chemical Formula: $C_{51}H_{75}N_{13}O_{13}S_3$
Exact Mass: 1173.48
Molecular Weight: 1174.42

Starting material mass: 5 mg
Final product mass: 1.4 mg

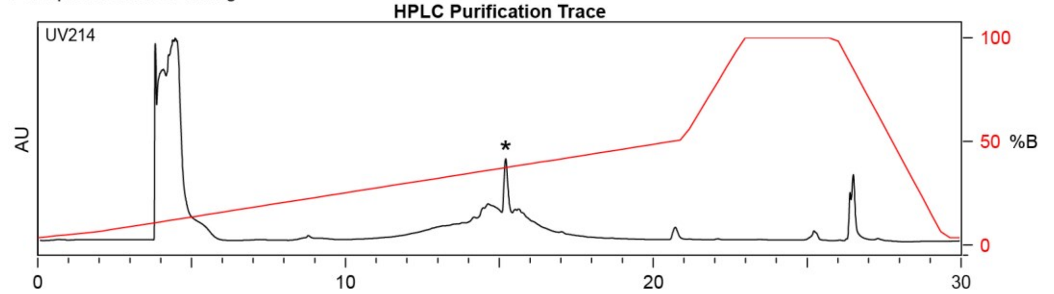

Time (min)	Solvent B (%)
0	2
2	2
21	50
23	100
26	100
29.5	2
30	2

Flow Rate: 13 mL / min
Phenomenex Kinetex EVO C18 Prep Column
(100 Å, 5 µm, 21.5 mm X 250 mm)

Slovent A: H_2O + 0.1% (v/v) TFA
Slovent B: MeCN + 0.1% (v/v) TFA

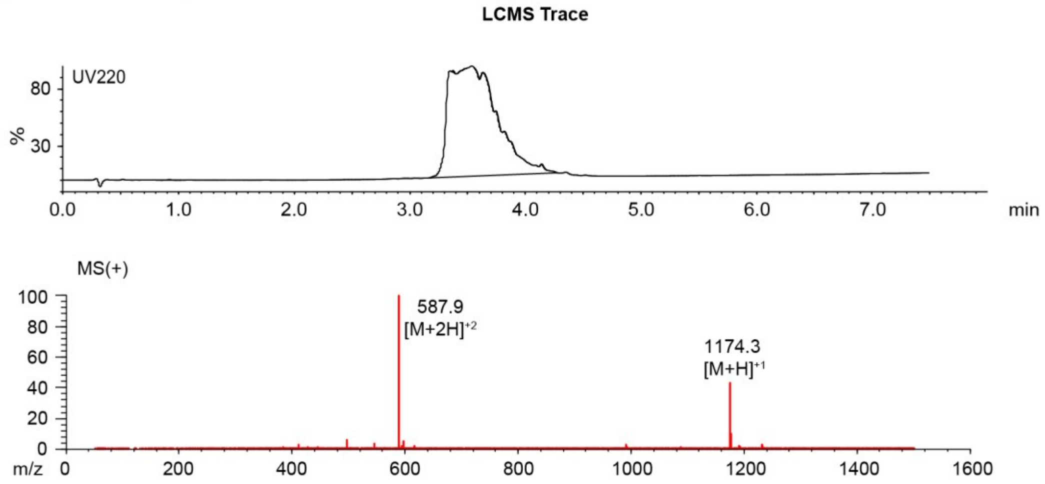

Figure S66: Summary for **22g** synthesis

7. NMR spectra

7.1. *NMR spectra for **TSL-1**, **TSL-3** and **TSL-6***

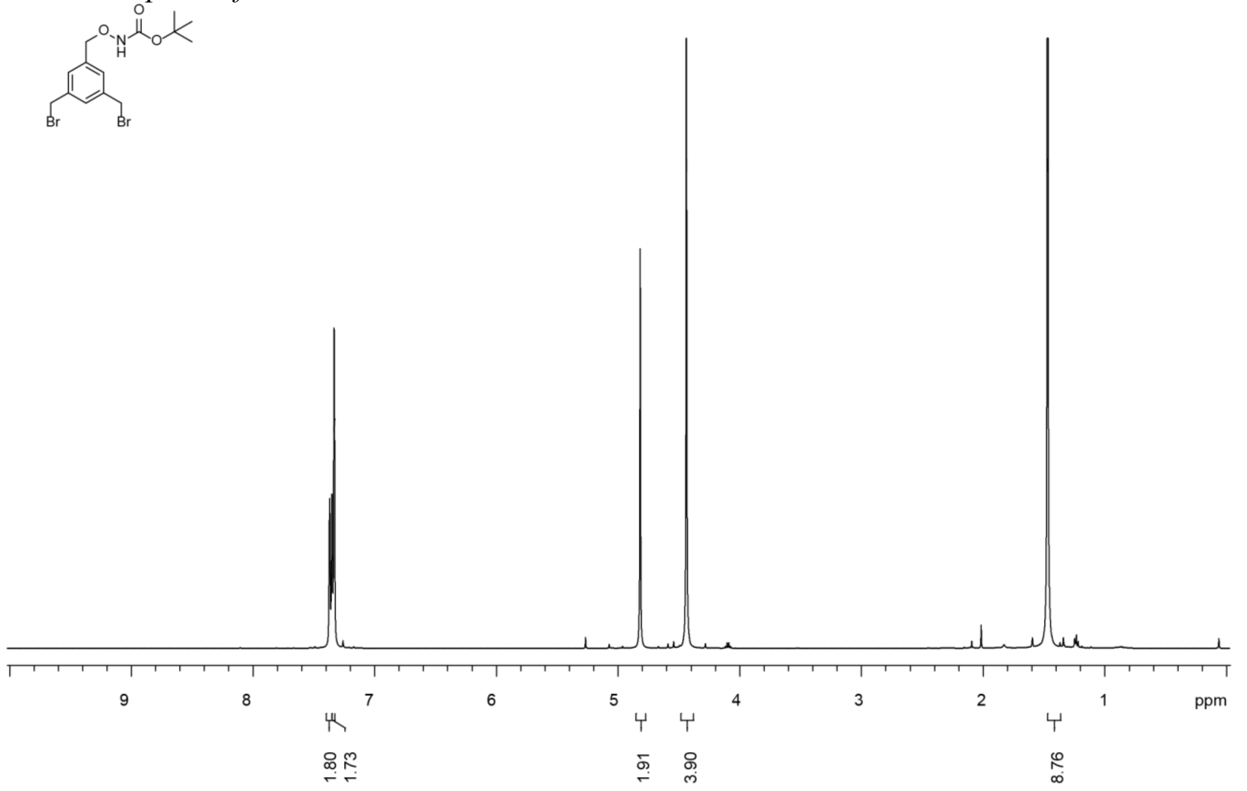

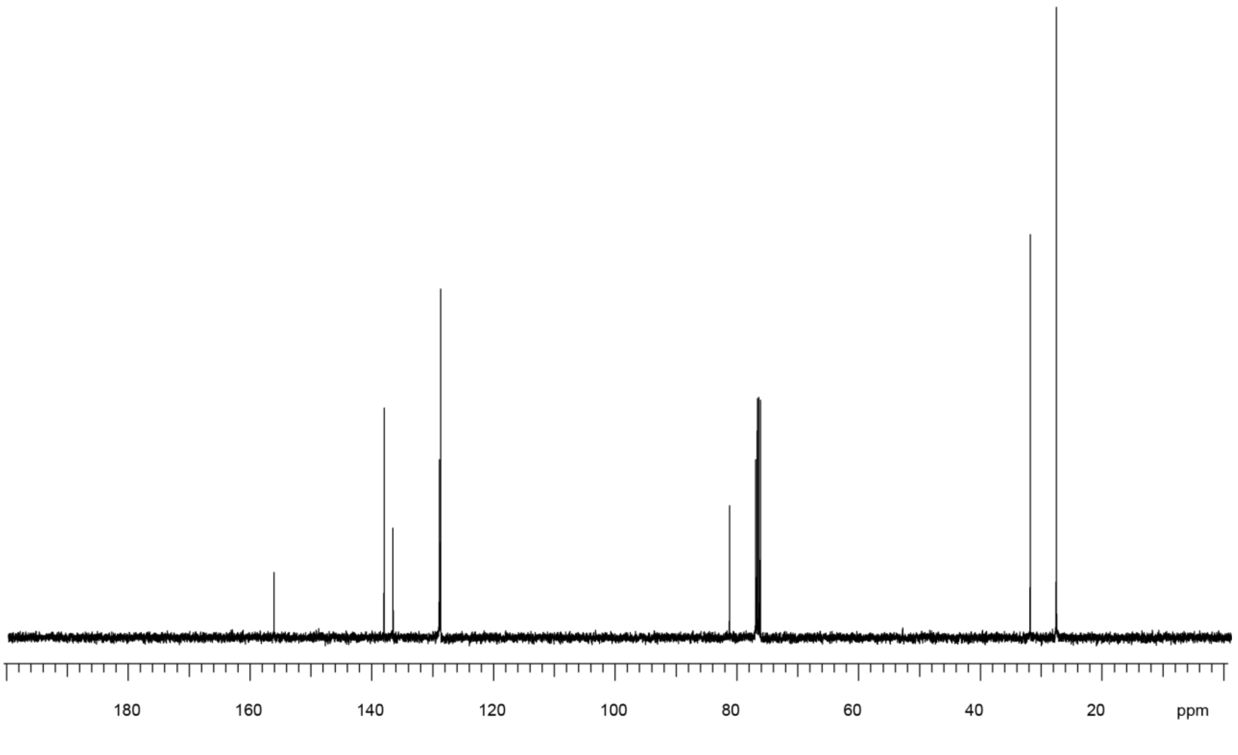

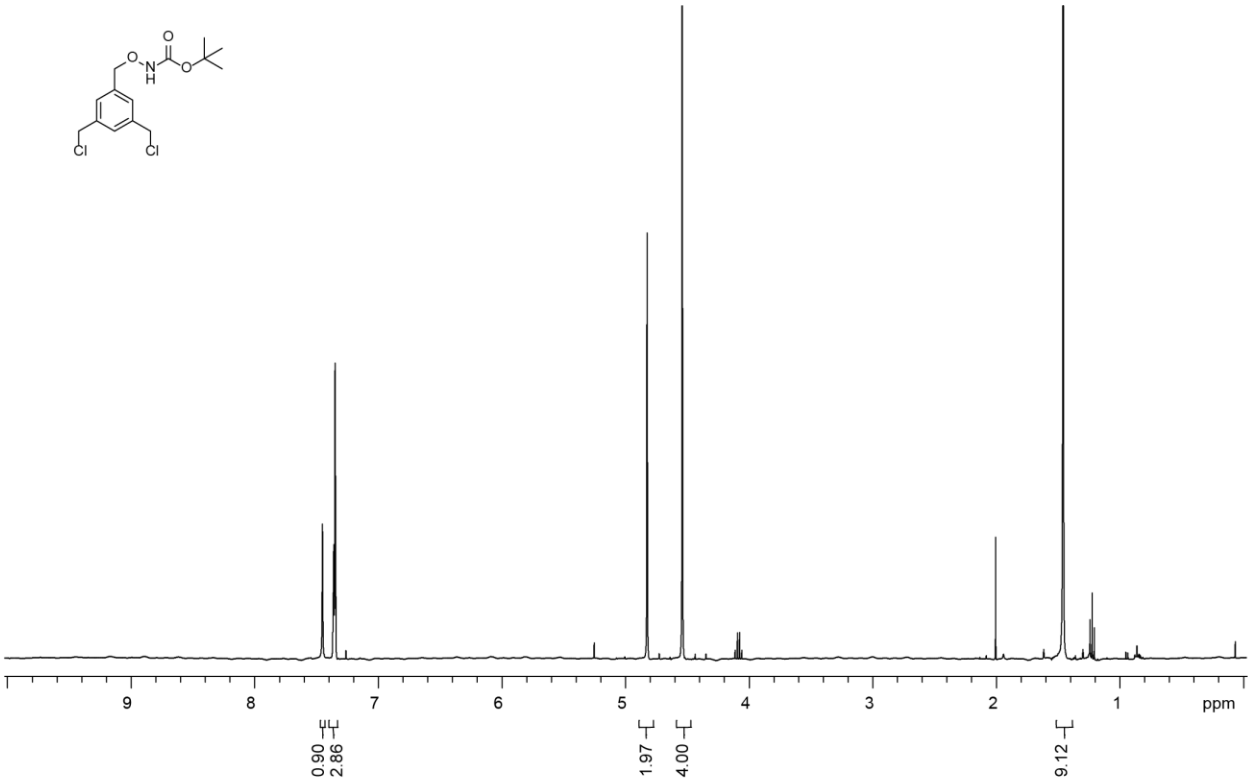
O
O
N
H
O
Cl
Cl
9
8
7
6
5
4
3
2
1
ppm
0.90
2.86
1.97
4.00
9.12

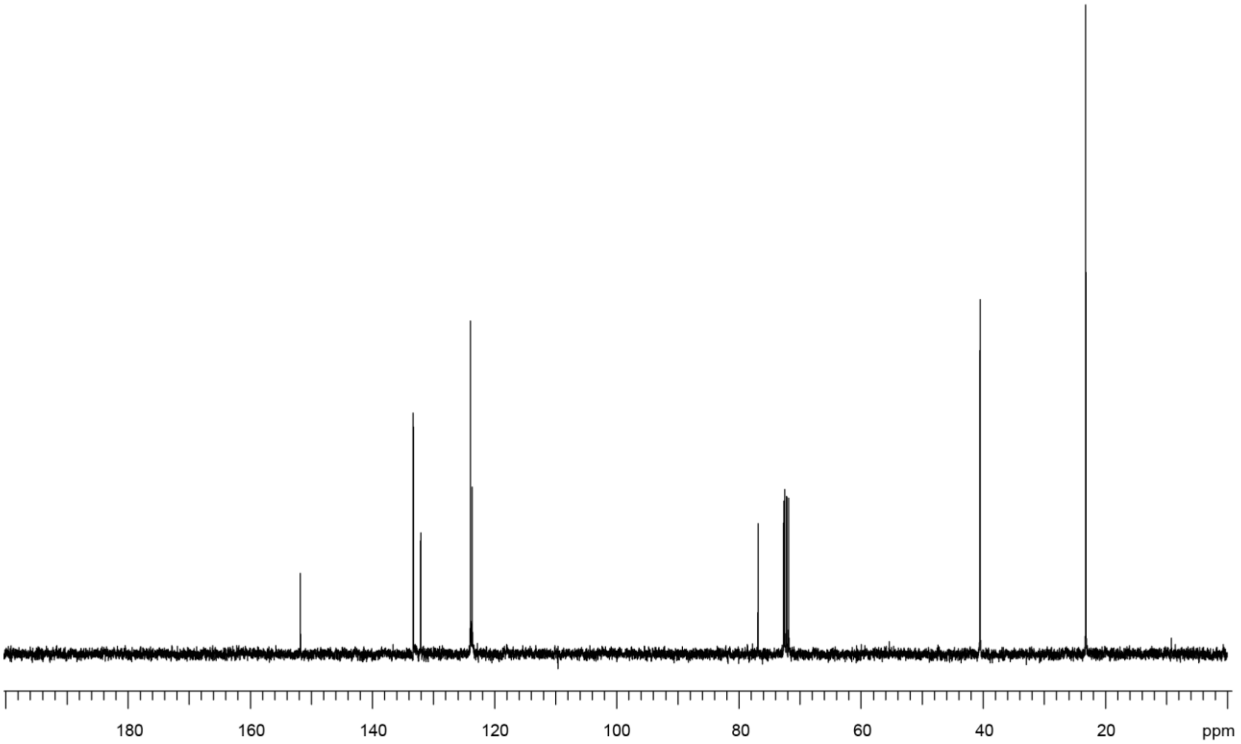
180
160
140
120
100
80
60
40
20
ppm

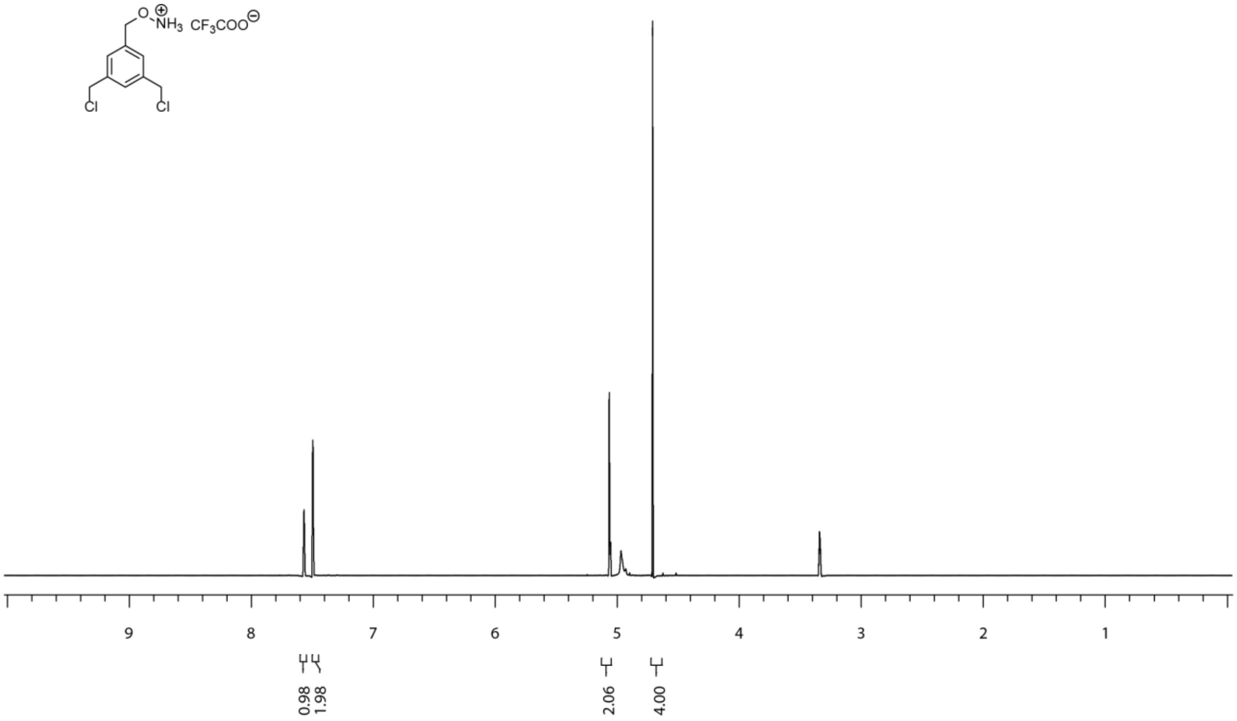
NH3 CF3COO
Cl
Cl
9
8
7
6
5
4
3
2
1
0.98
1.98
2.06
4.00

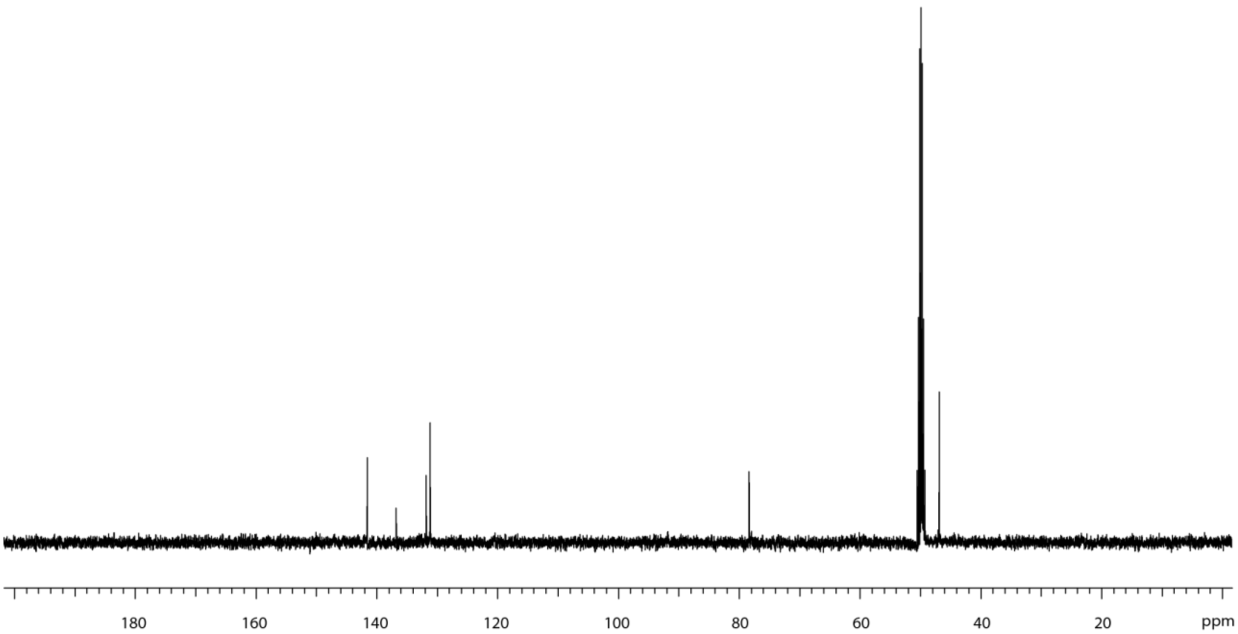
180
160
140
120
100
80
60
40
20
ppm

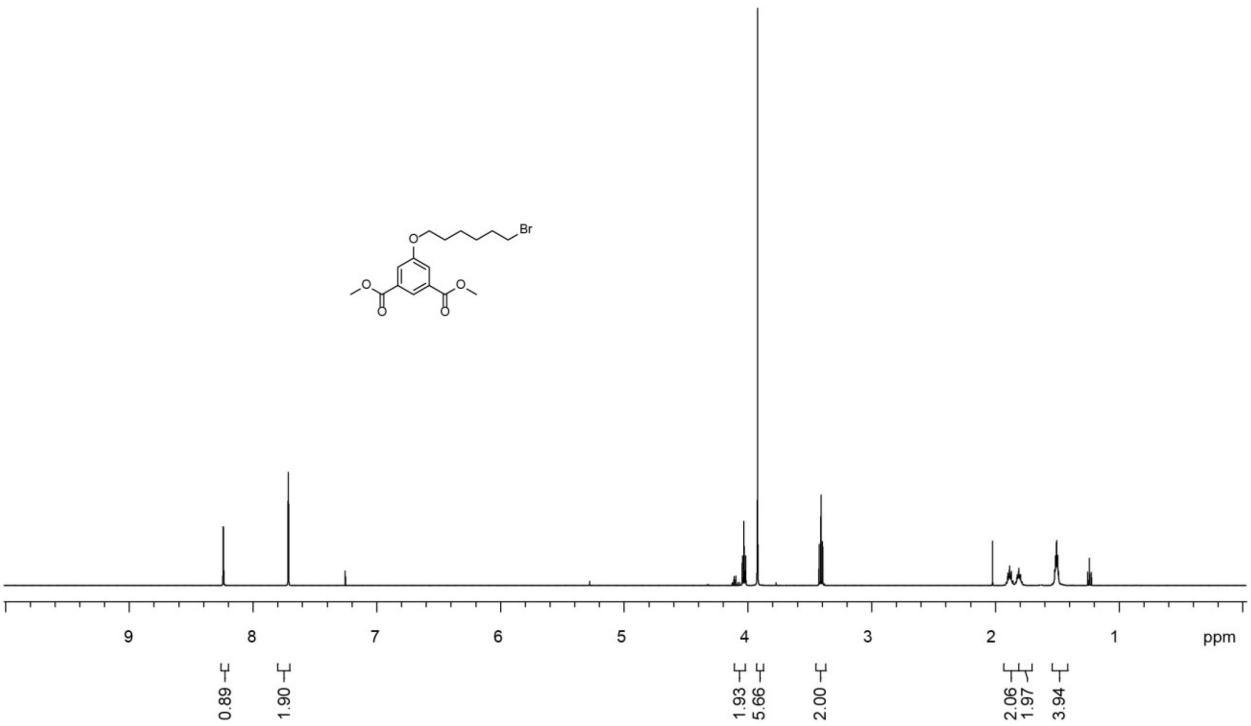
Br
9
8
7
6
5
4
3
2
1
ppm
0.89
1.90
1.93
5.66
2.00
2.06
1.97
3.94

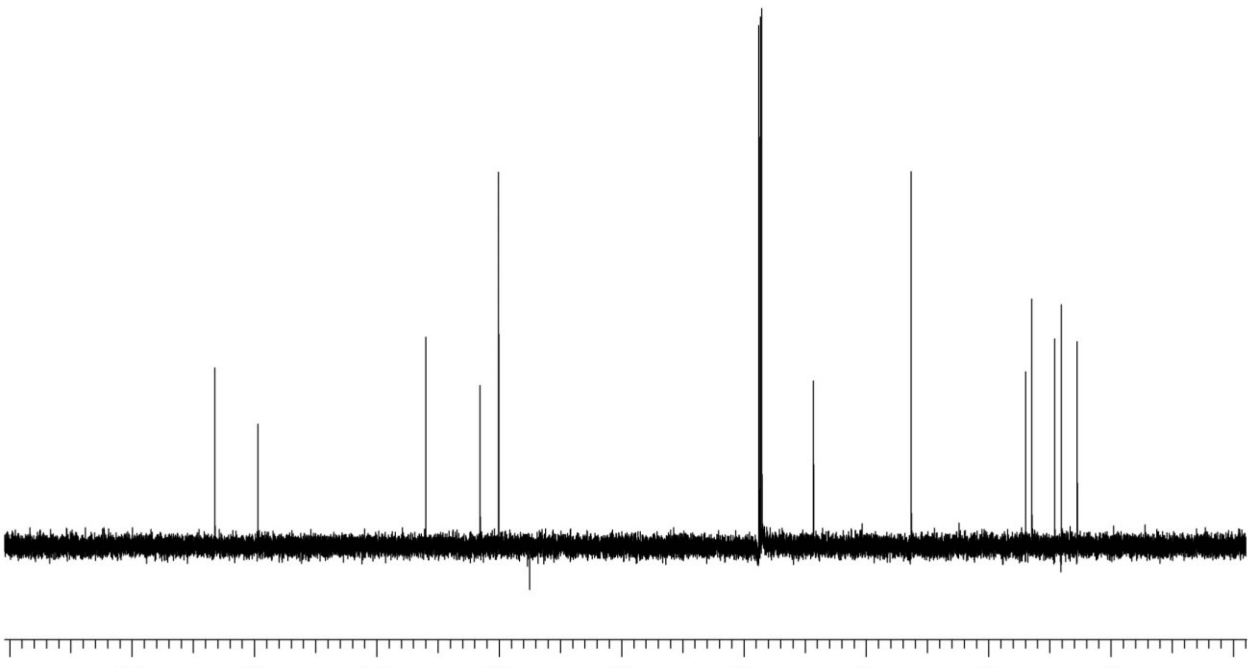
180
160
140
120
100
80
60
40
20
ppm

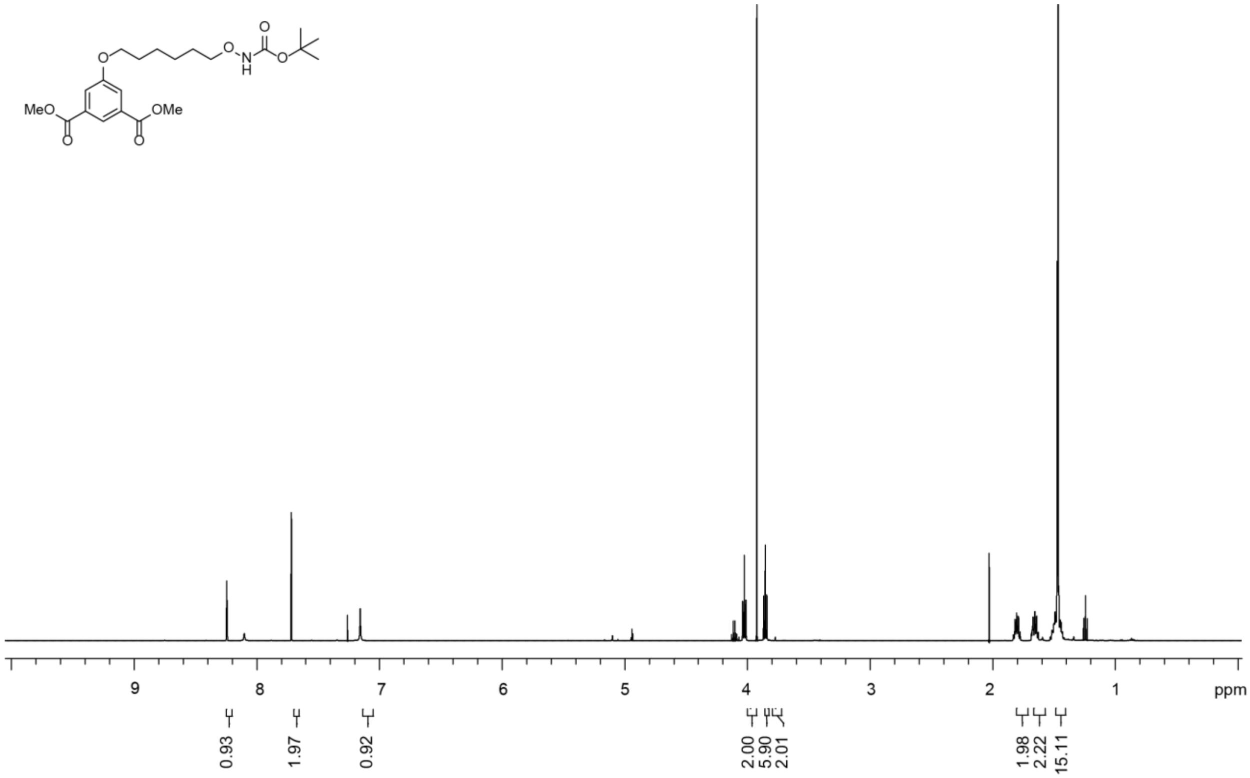

MeO
OMe
O
N
H
9
8
7
6
5
4
3
2
1
ppm
0.93
1.97
0.92
2.00
5.90
2.01
1.98
2.22
15.11

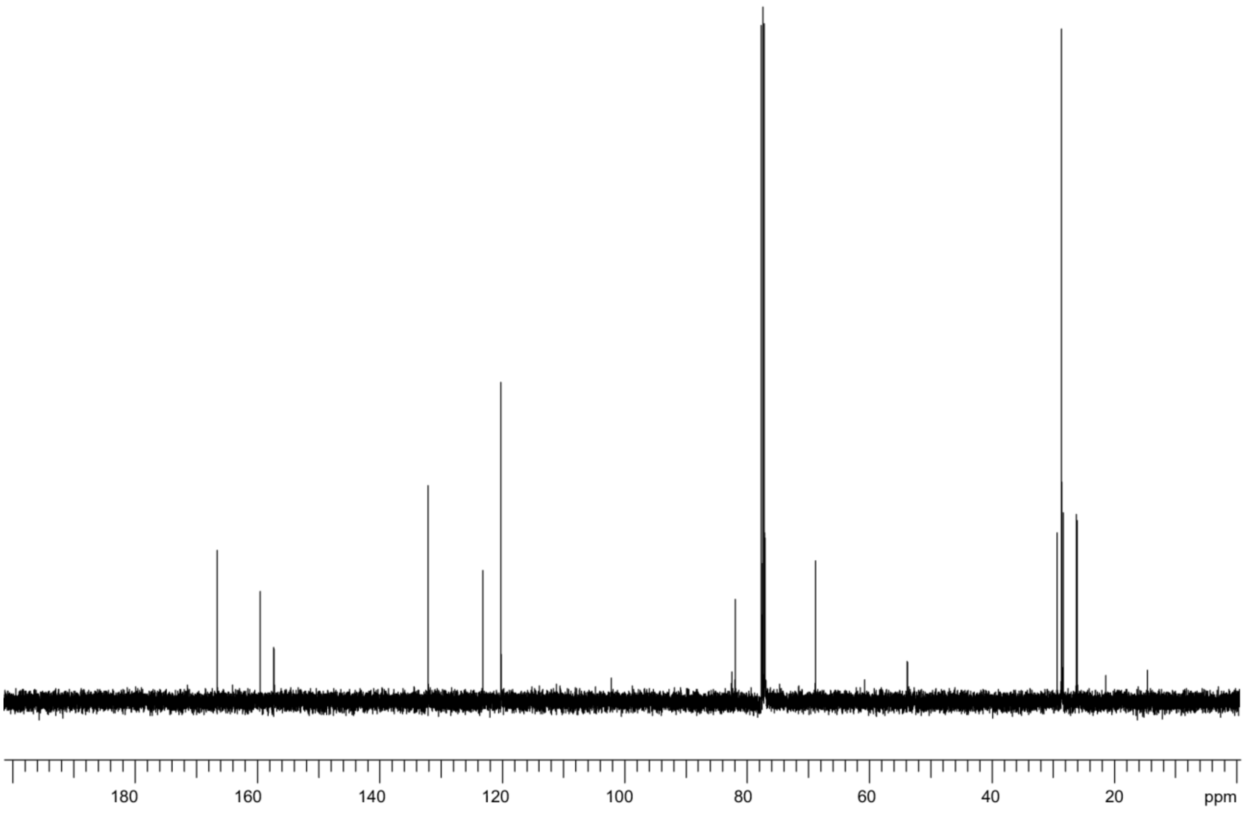

180
160
140
120
100
80
60
40
20
ppm

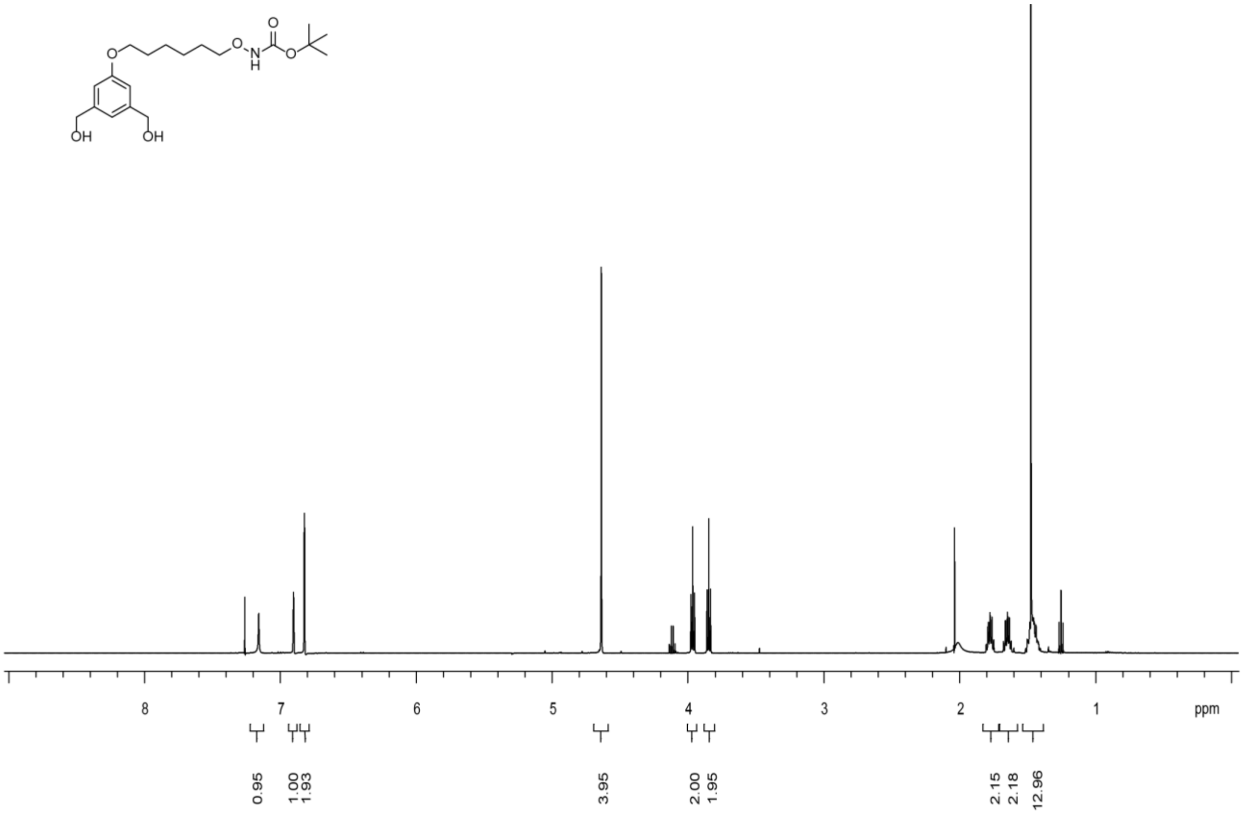
O
N
H
OH
OH
8
7
6
5
4
3
2
1
ppm
0.95
1.00
1.93
3.95
2.00
1.95
2.15
2.18
12.96

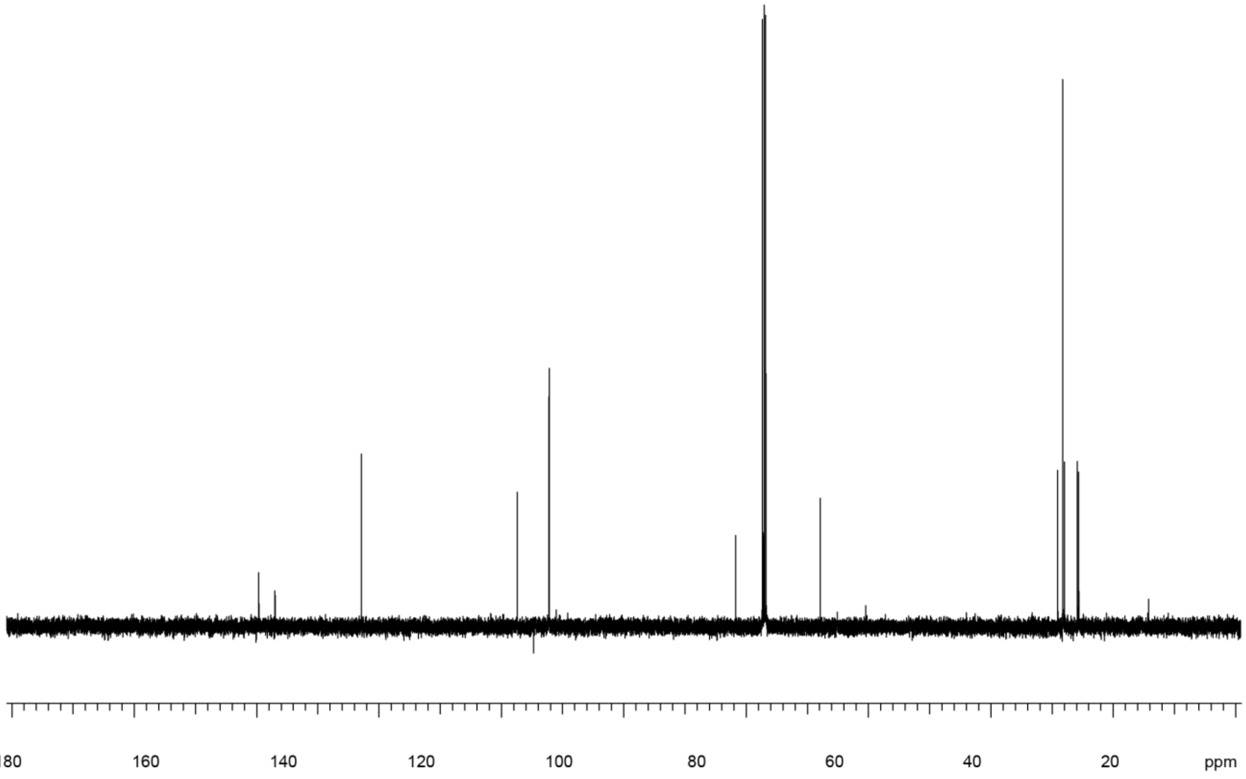
180
160
140
120
100
80
60
40
20
ppm

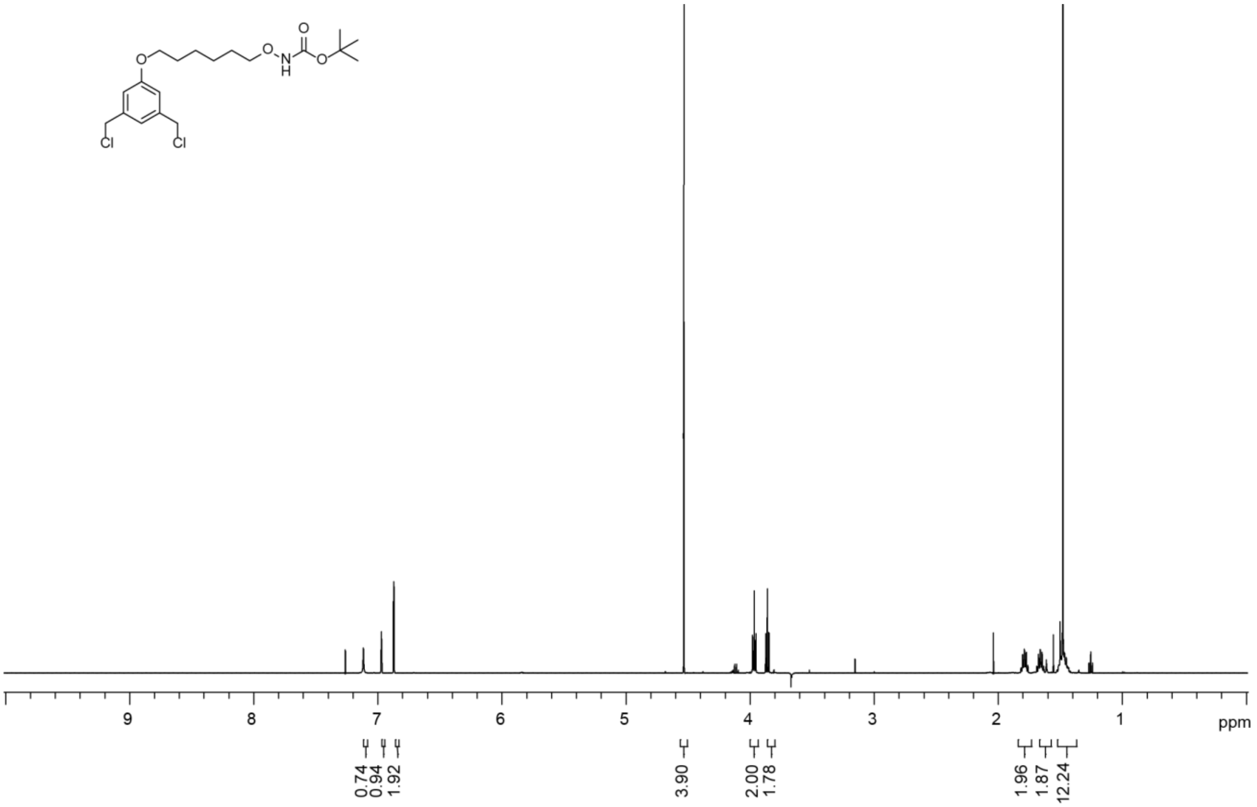
O
O
N
H
O
O
Cl
Cl
9 8 7 6 5 4 3 2 1 ppm
0.74
0.94
1.92
3.90
2.00
1.78
1.96
1.87
12.24

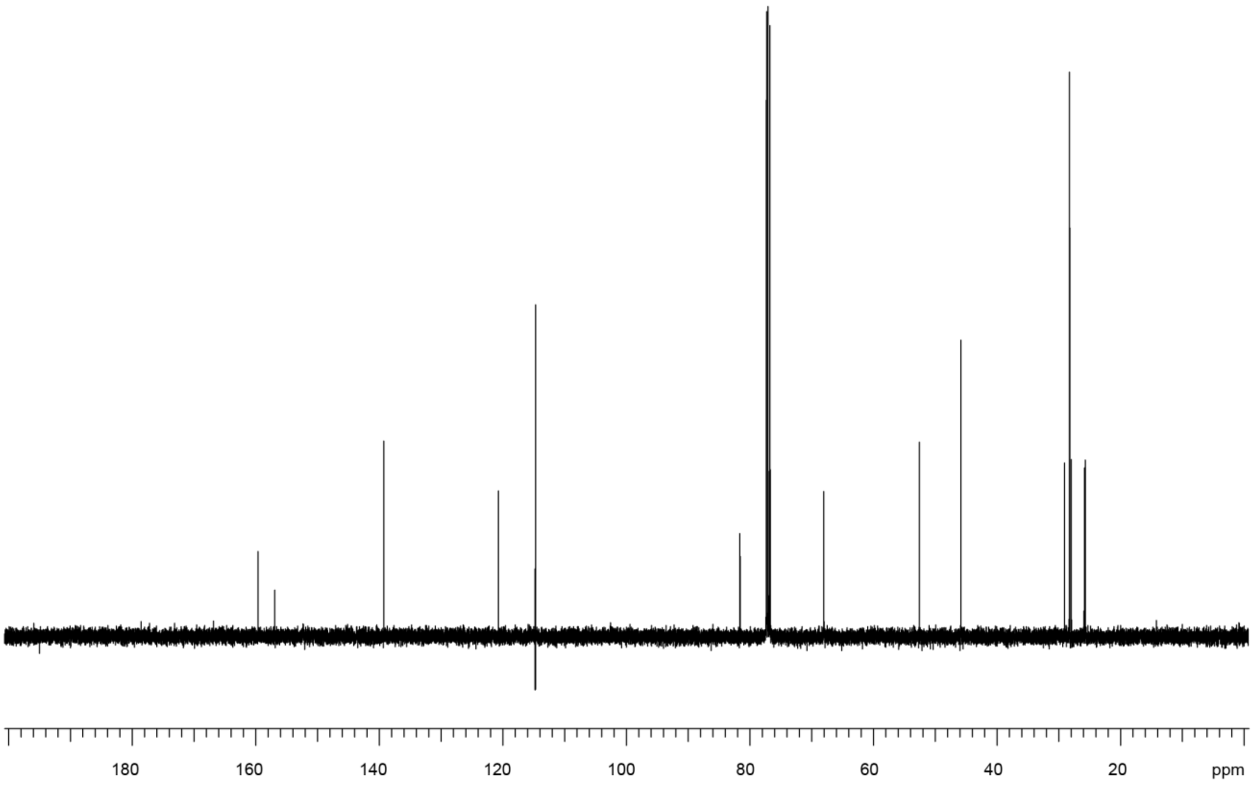
180 160 140 120 100 80 60 40 20 ppm

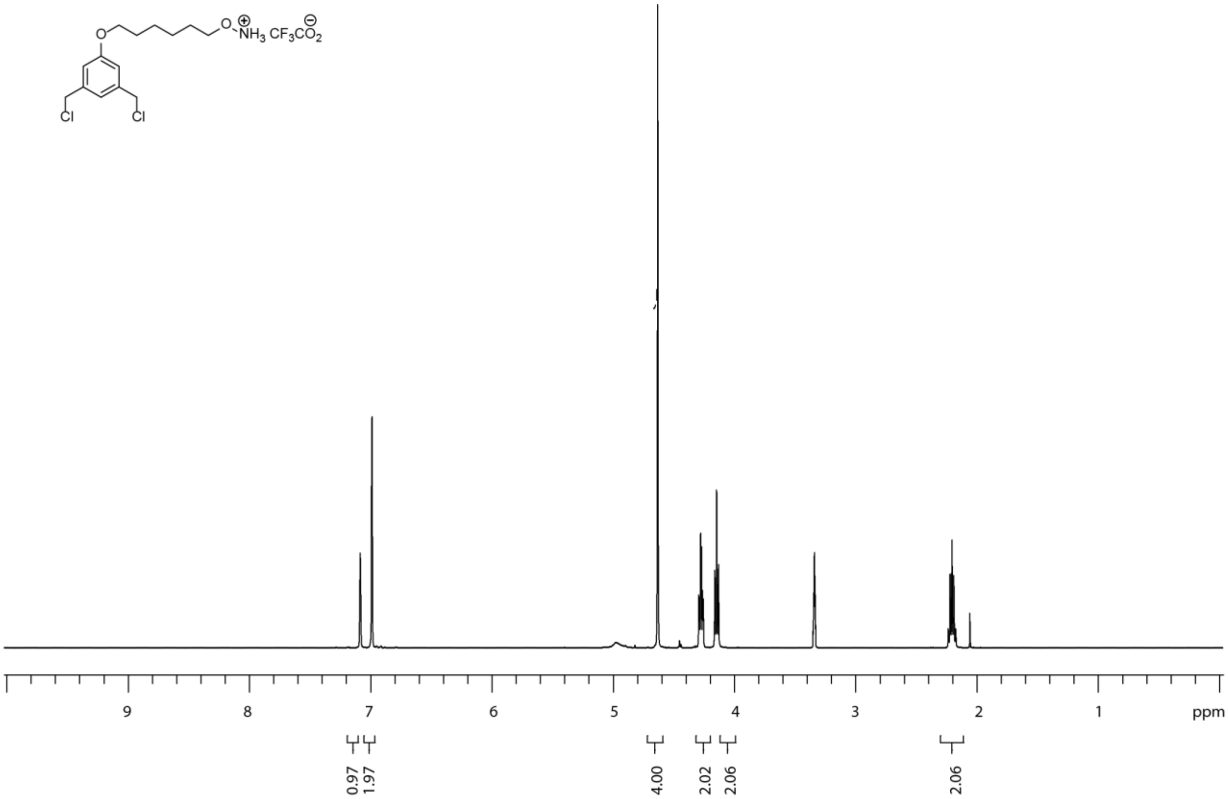
O
NH3 CF3CO2
Cl
Cl
9 8 7 6 5 4 3 2 1 ppm
0.97
1.97
4.00
2.02
2.06
2.06

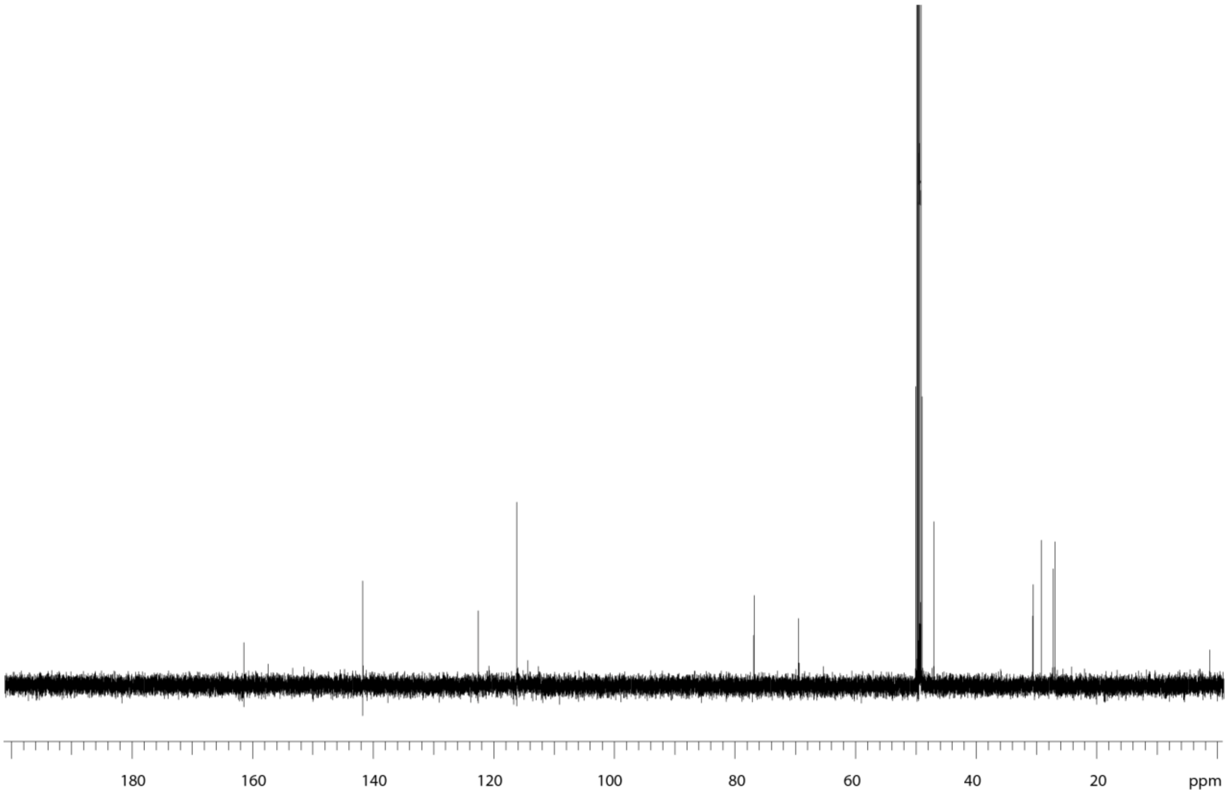
180 160 140 120 100 80 60 40 20 ppm

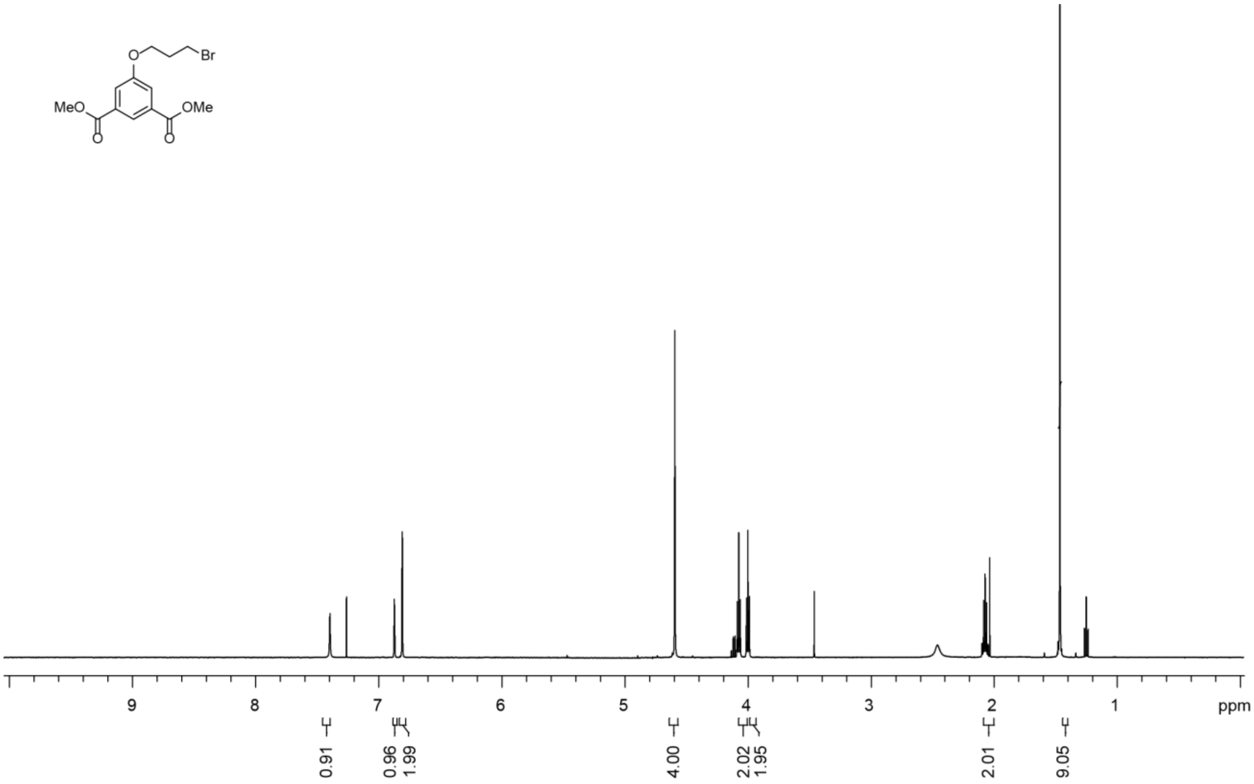
O
Br
MeO
OMe
O
O
9
8
7
6
5
4
3
2
1
ppm
0.91
0.96
1.99
4.00
2.02
1.95
2.01
9.05

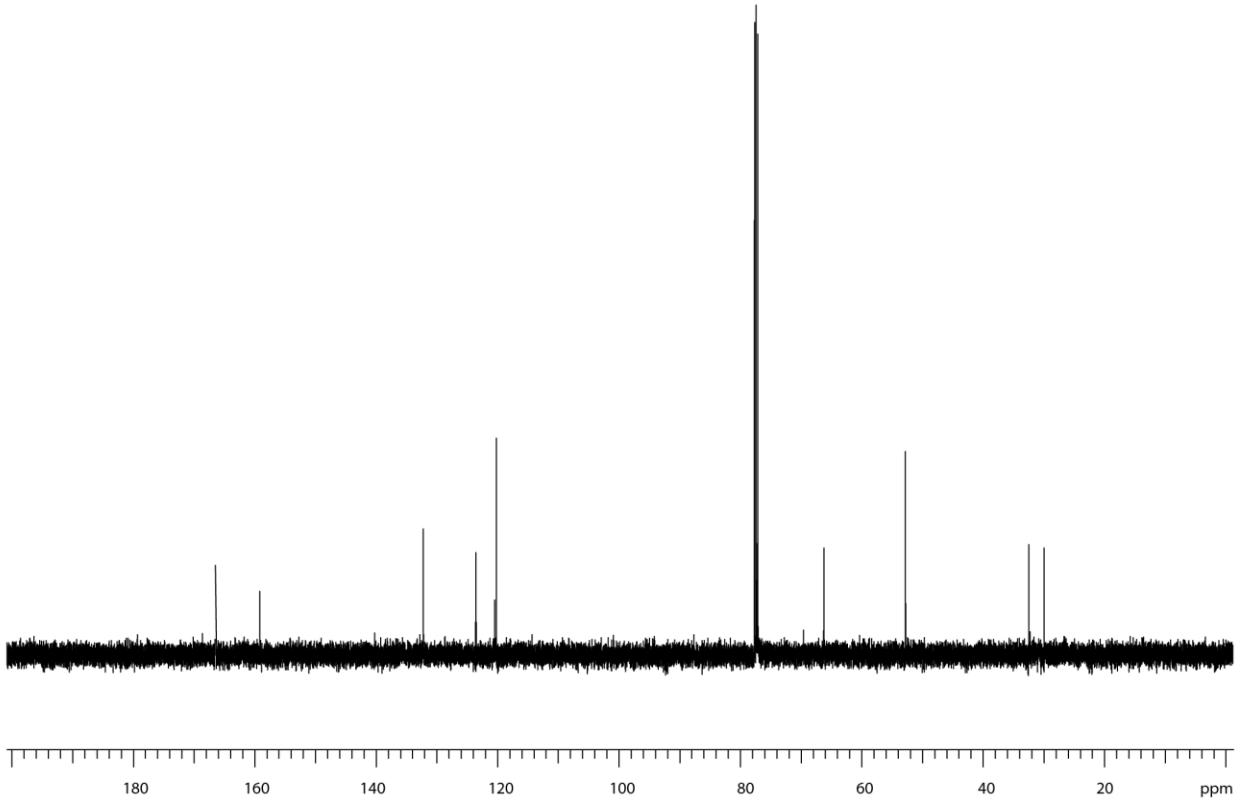
180
160
140
120
100
80
60
40
20
ppm

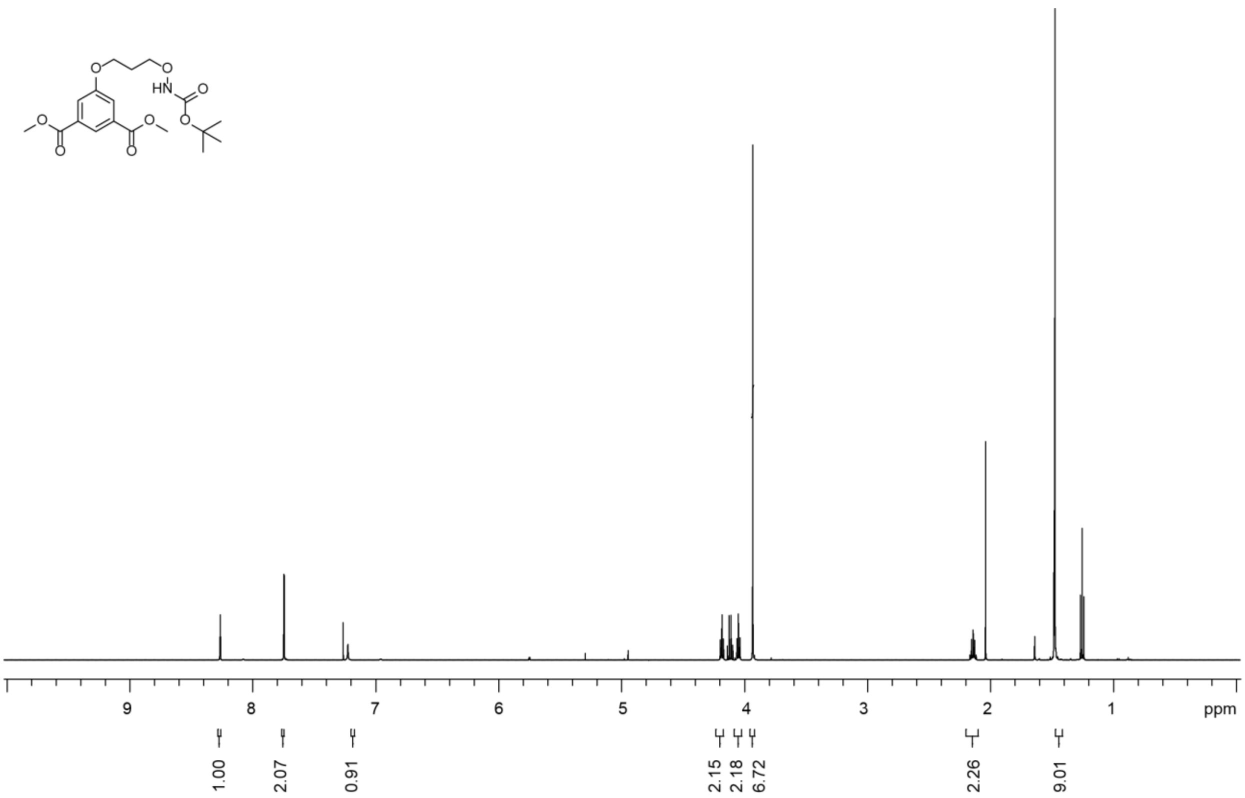

9
8
7
6
5
4
3
2
1
ppm
1.00
2.07
0.91
2.15
2.18
6.72
2.26
9.01

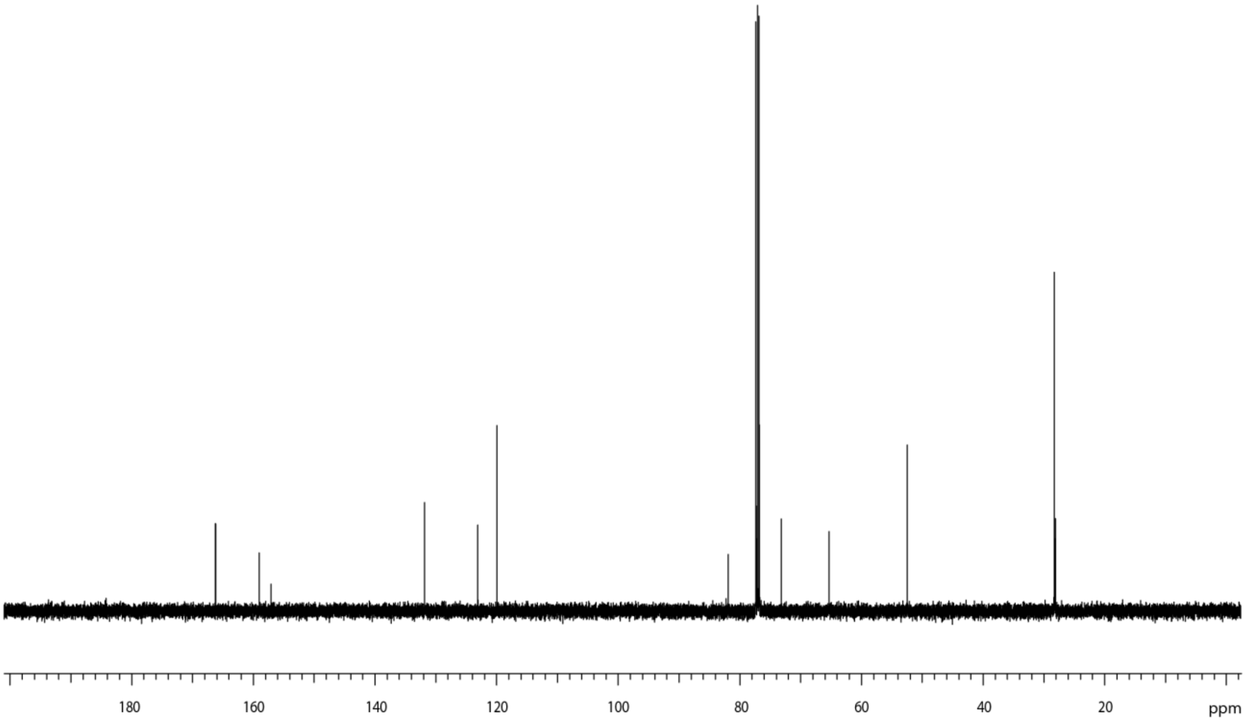

180
160
140
120
100
80
60
40
20
ppm

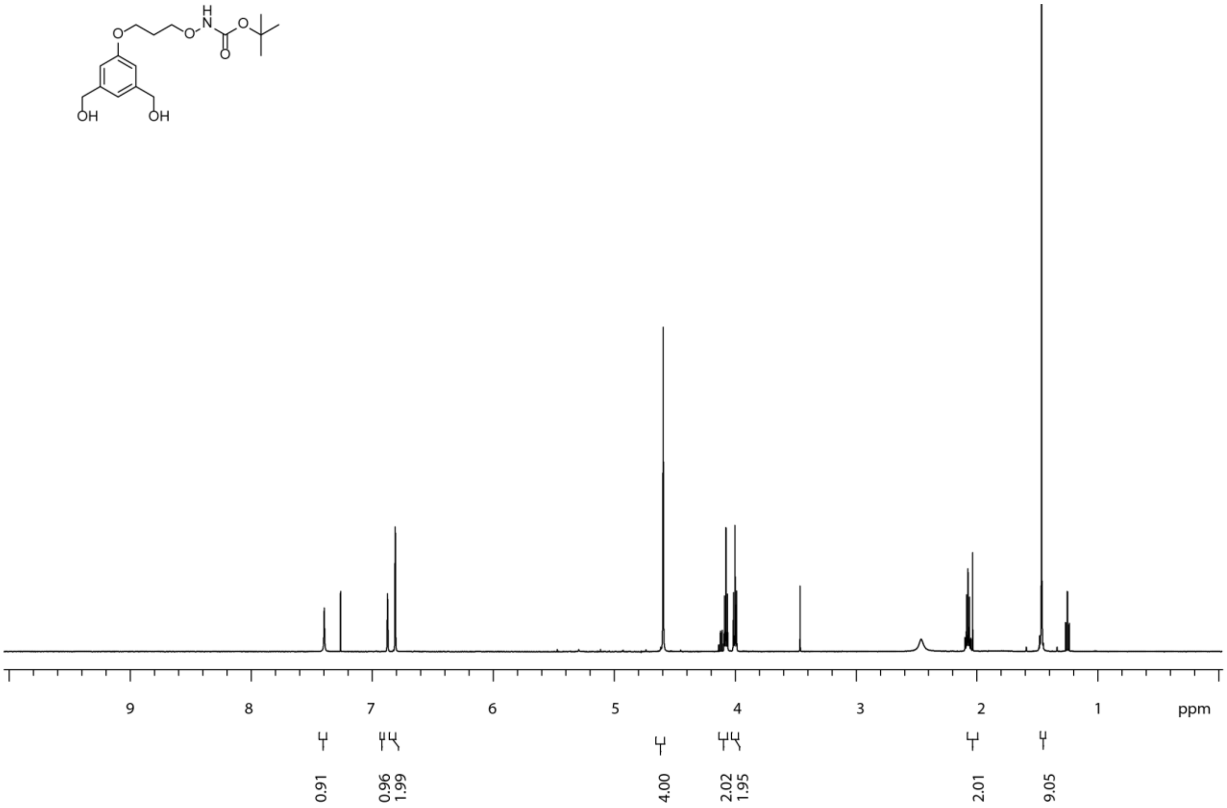

O
H
N
O
O
O
OH
OH
9
8
7
6
5
4
3
2
1
ppm
0.91
0.96
1.99
4.00
2.02
1.95
2.01
9.05

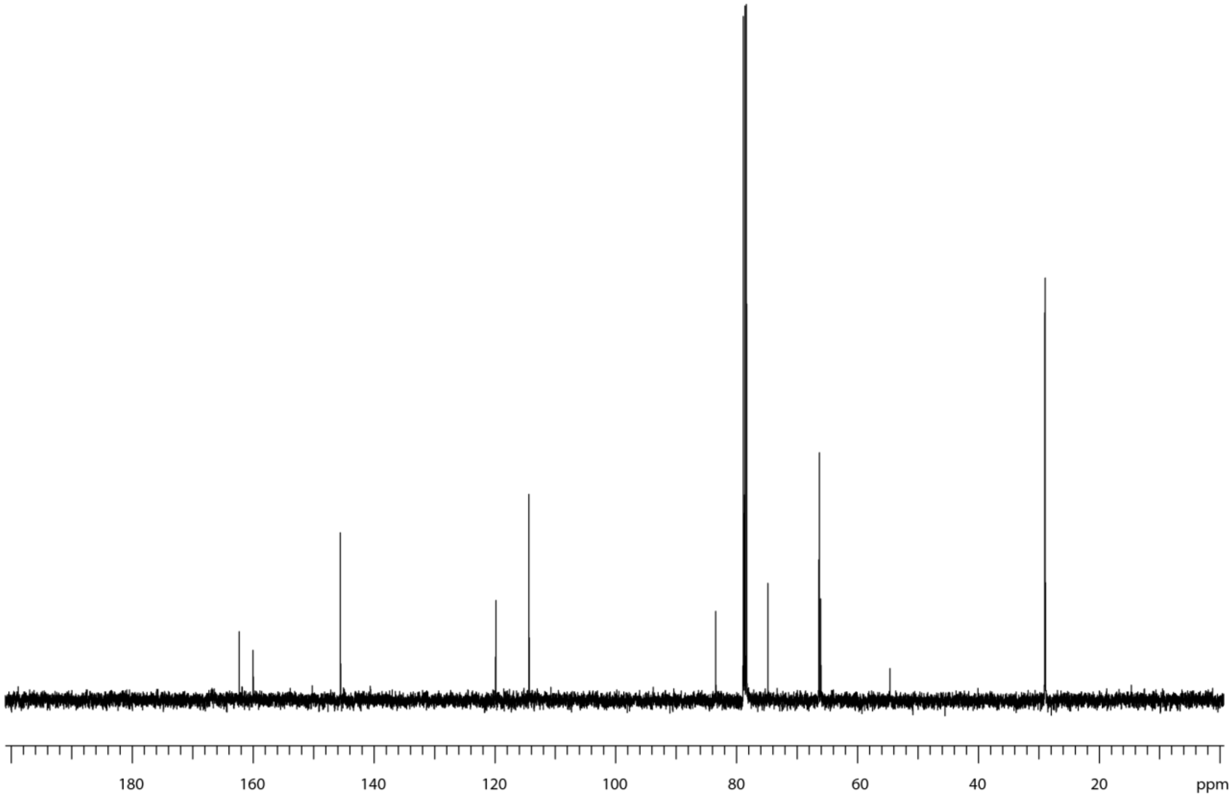

180
160
140
120
100
80
60
40
20
ppm

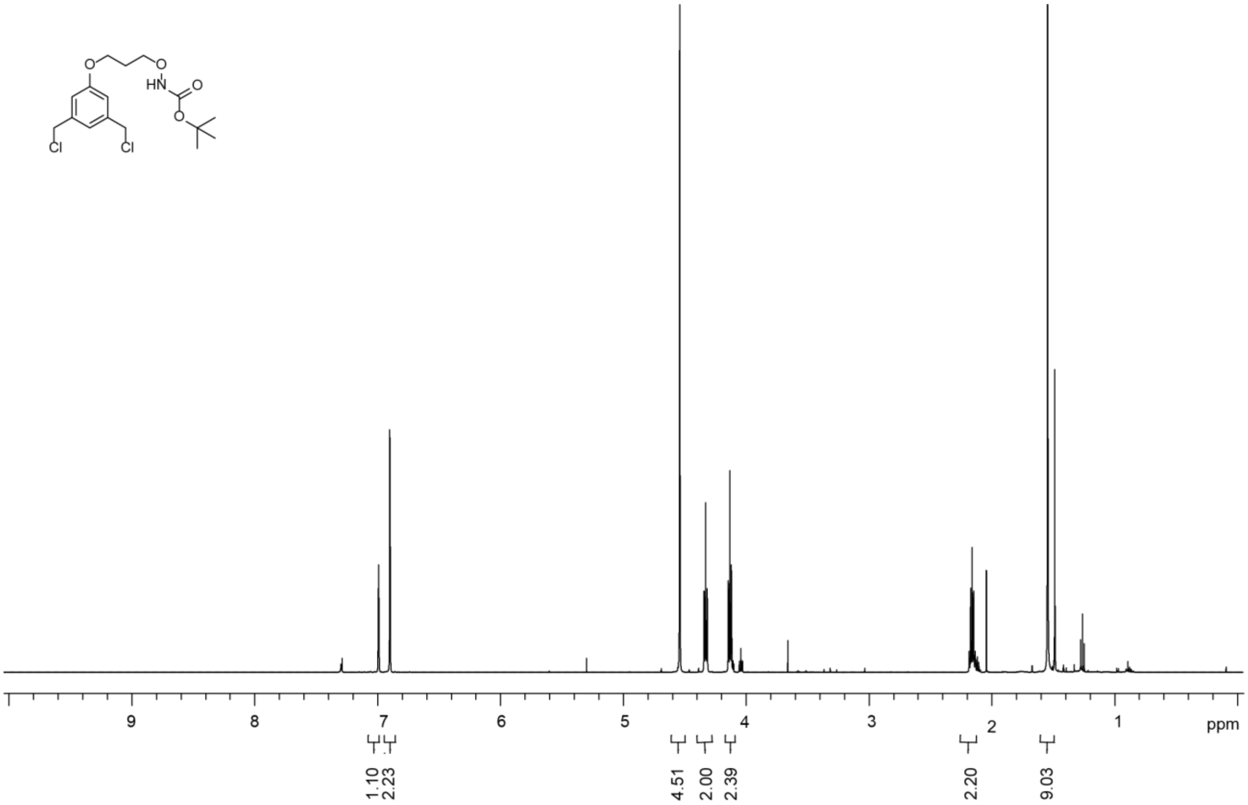
HN
O
O
O
O
Cl
Cl
9
8
7
6
5
4
3
2
1
ppm
1.10
2.23
4.51
2.00
2.39
2.20
9.03

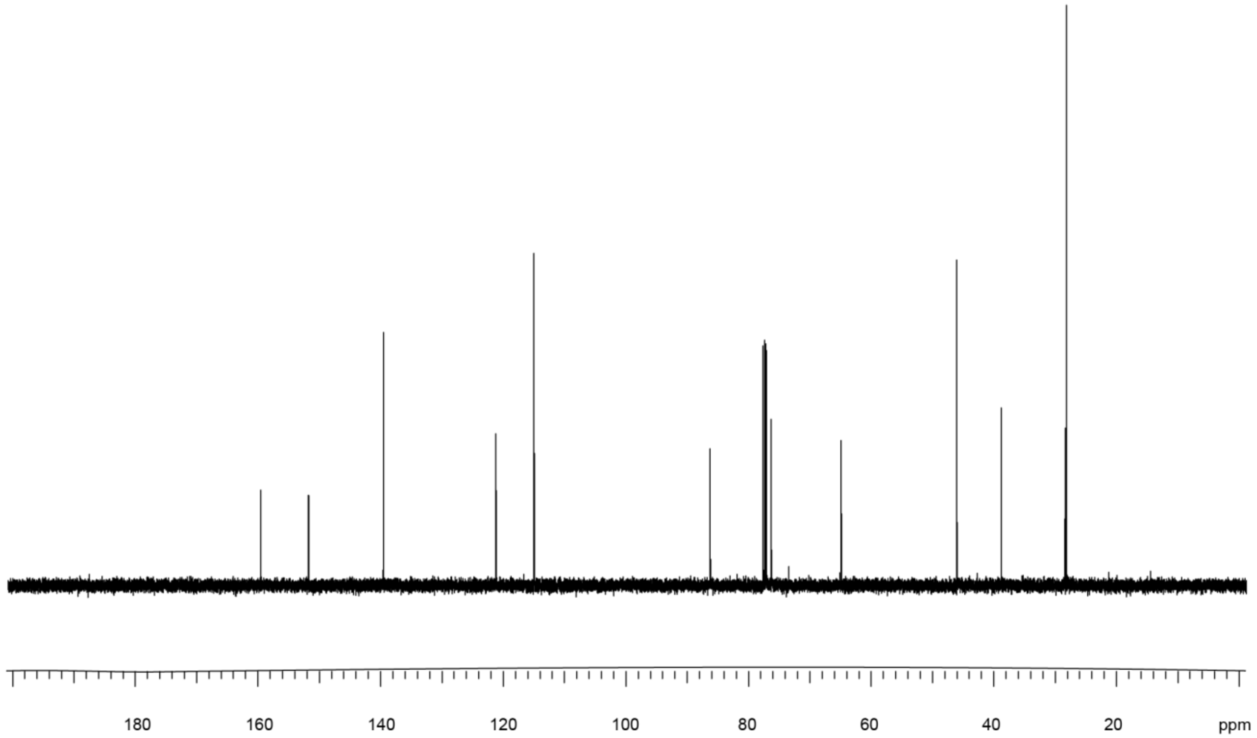
180
160
140
120
100
80
60
40
20
ppm

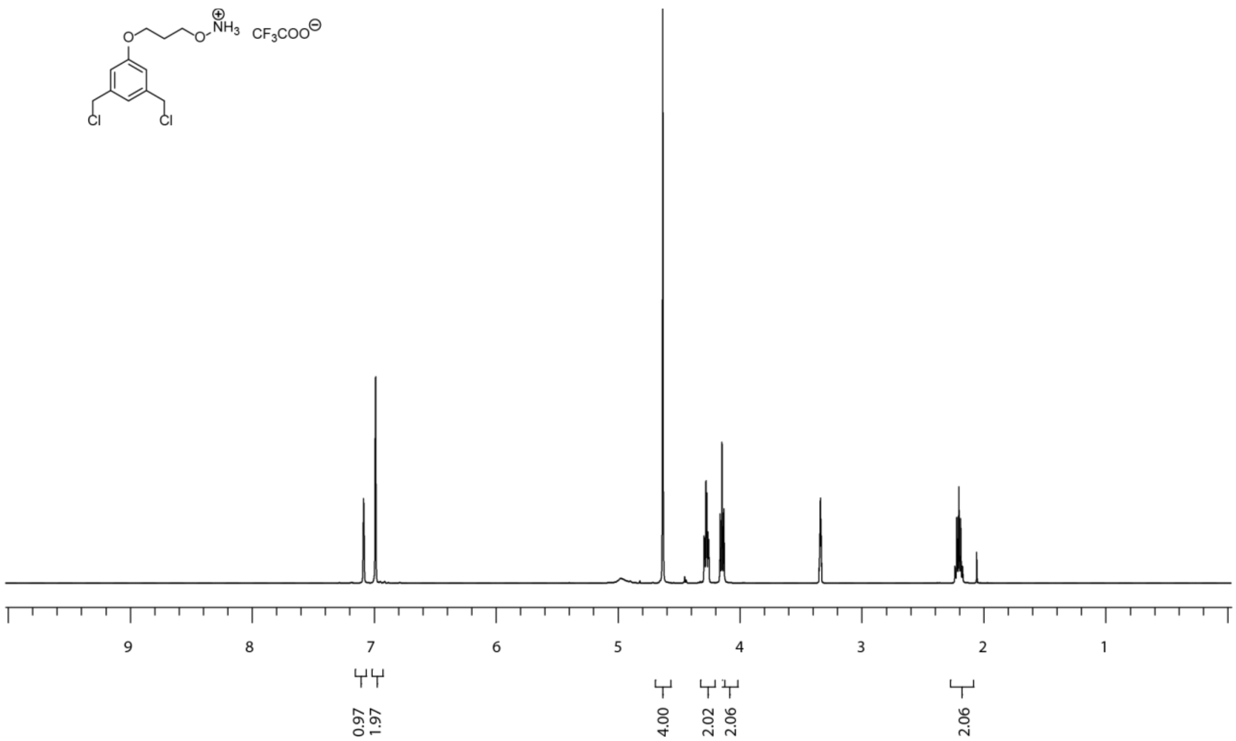
NH3 CF3COO
Cl
Cl
9 8 7 6 5 4 3 2 1
0.97 1.97 4.00 2.02 2.06 2.06

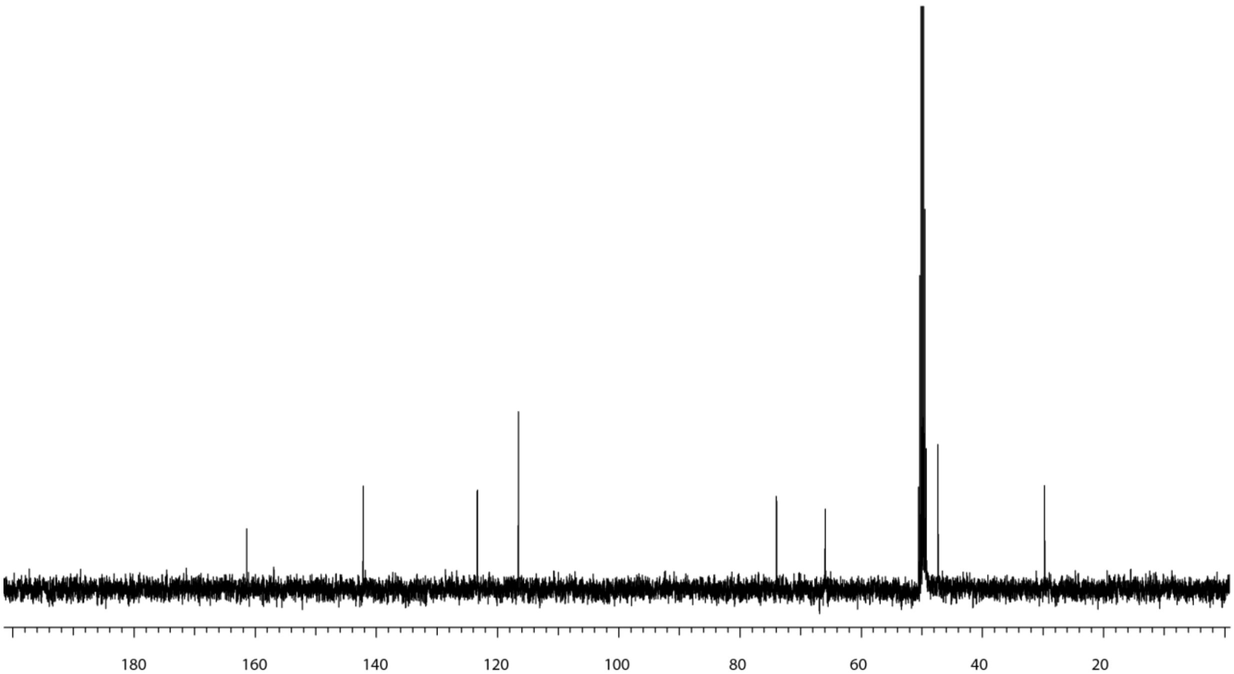
180 160 140 120 100 80 60 40 20

7.2. *Proton NMR assignment and corresponding NMR spectra of **7c*** (1H, COSY, TOCSY, NOESY and ROESY)

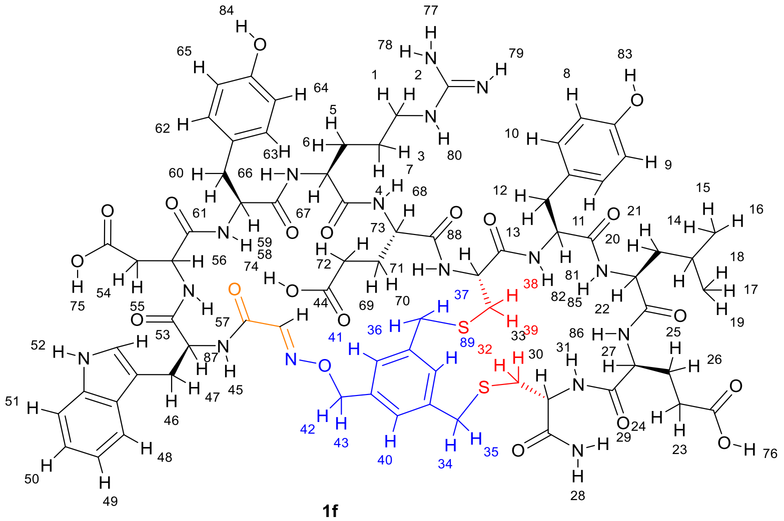

1f

Residue	NH	ppm	Hα	ppm	Hβ'	ppm	Hγ'	ppm	Hδ'	ppm	Hε'	ppm
Trp	45	7.54	87	4.52	46, 47	3.16						
Asp	57	7.50	56	4.54	54, 55	2.66, 2.48						
Tyr	58	7.79	59	4.32	60, 61	2.90						
Arg	66	8.12	67	3.83	5, 6	1.69	1, 2	1.47	80	3.05	77-80	7.15
Glu	7	7.58	68	4.12	69, 70	2.00, 1.87	72, 73	2.36	-			
Cys	71	7.53	88	4.06	38, 39	2.64, 2.40						
Tyr	82	7.59	81	4.02	12, 13	2.77						
Leu	85	7.36	22	3.96	20, 21	1.55, 1.39	90	1.34				
Glu	86	7.63	27	4.19	25, 26	2.09, 1.99	23, 24	2.43, 2.38	-			
Cys	31	7.63	30	4.46	32, 33	3.00, 2.72		-	-			
Other signals												
Cys	28	7.04	29	6.71								
Trp (Ar)	52	9.96	53	7.02	50	7.13	51	7.56	48	7.36	49	7.05
Tyr (Ar)	62, 63	6.92	64, 65	6.60								
Tyr (Ar)	11, 10	6.92	8, 9	6.64								
TSL-1	89	7.12	40	7.04	41	6.95	42, 43	4.95	34, 35	3.66	36, 37	3.51

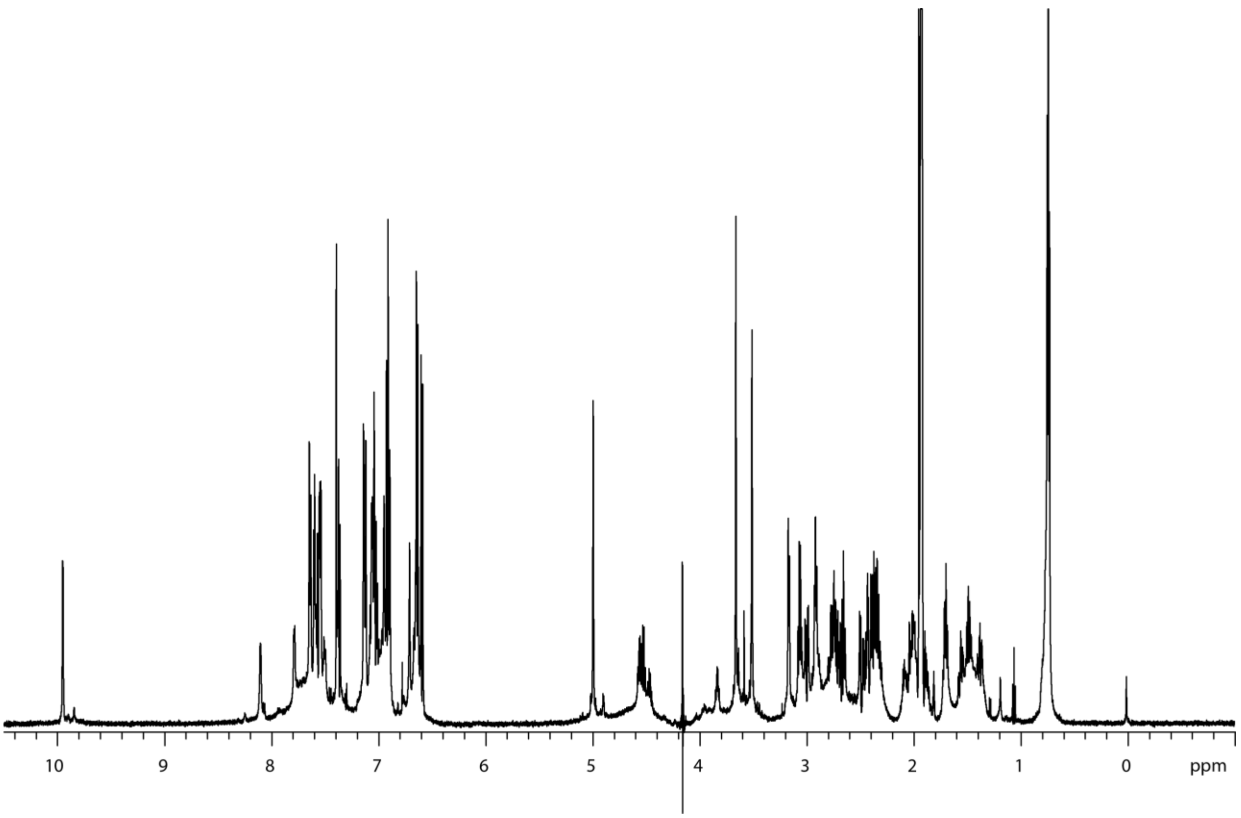

1H NMR of **7c**

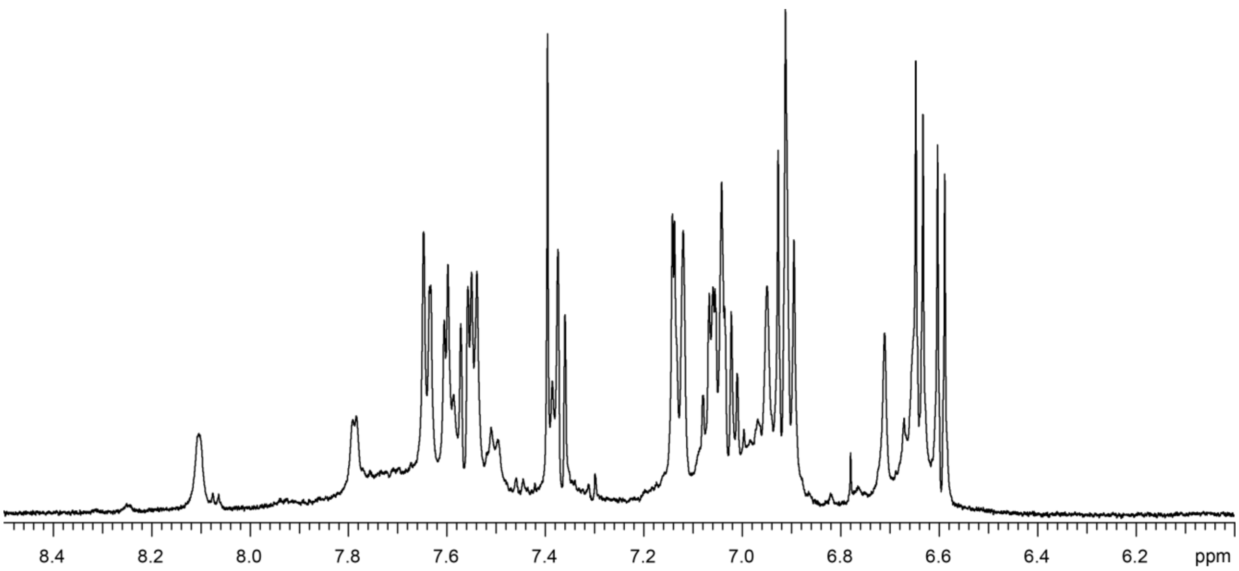

1H NMR of **7c** (expanded)

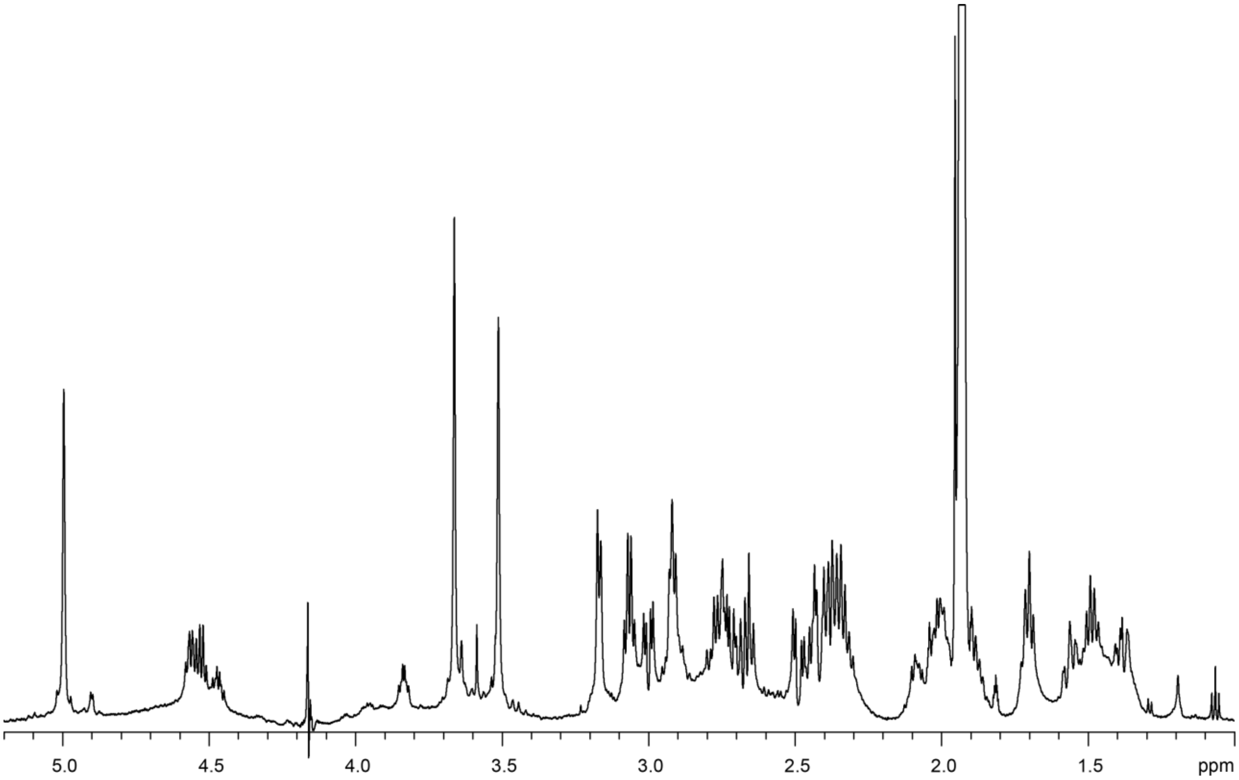

1H NMR of **7c** (expanded)

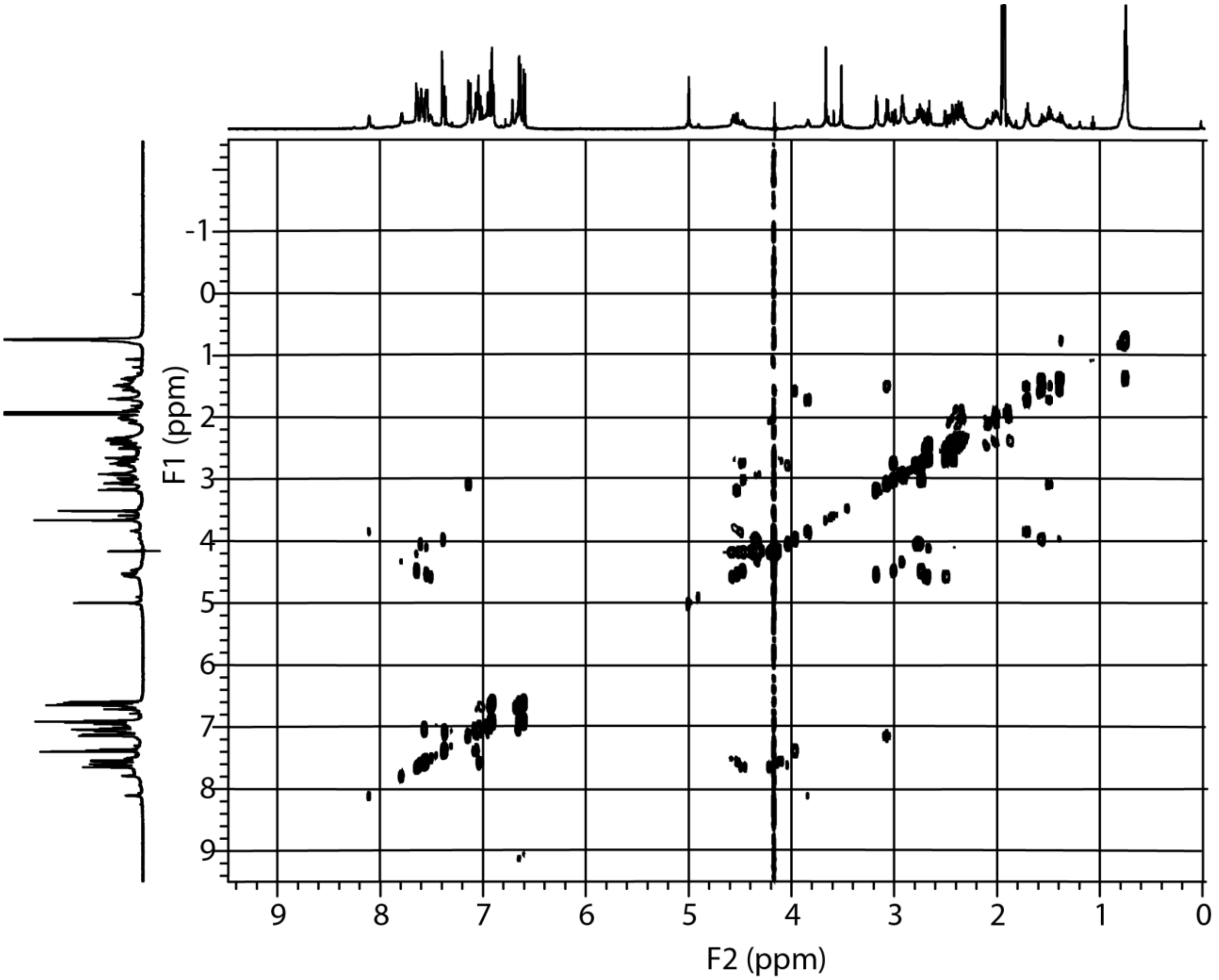

COSY NMR of **7c**

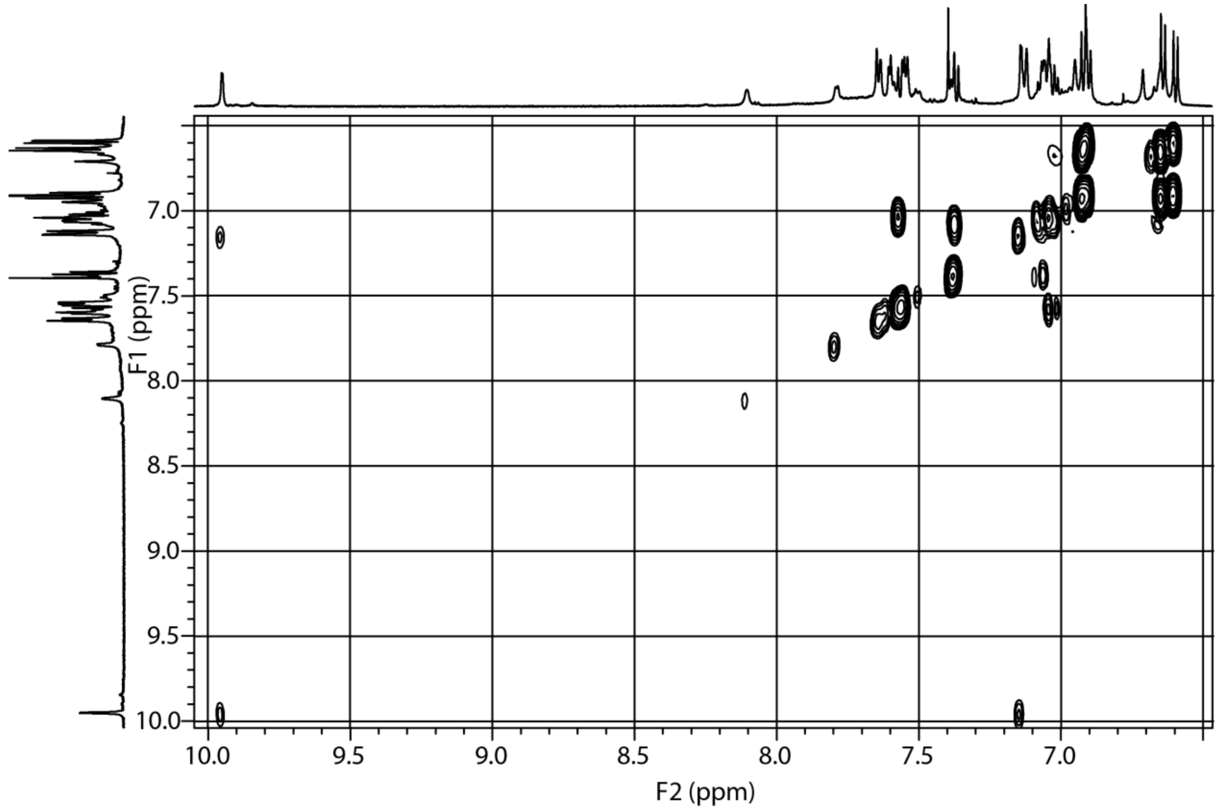

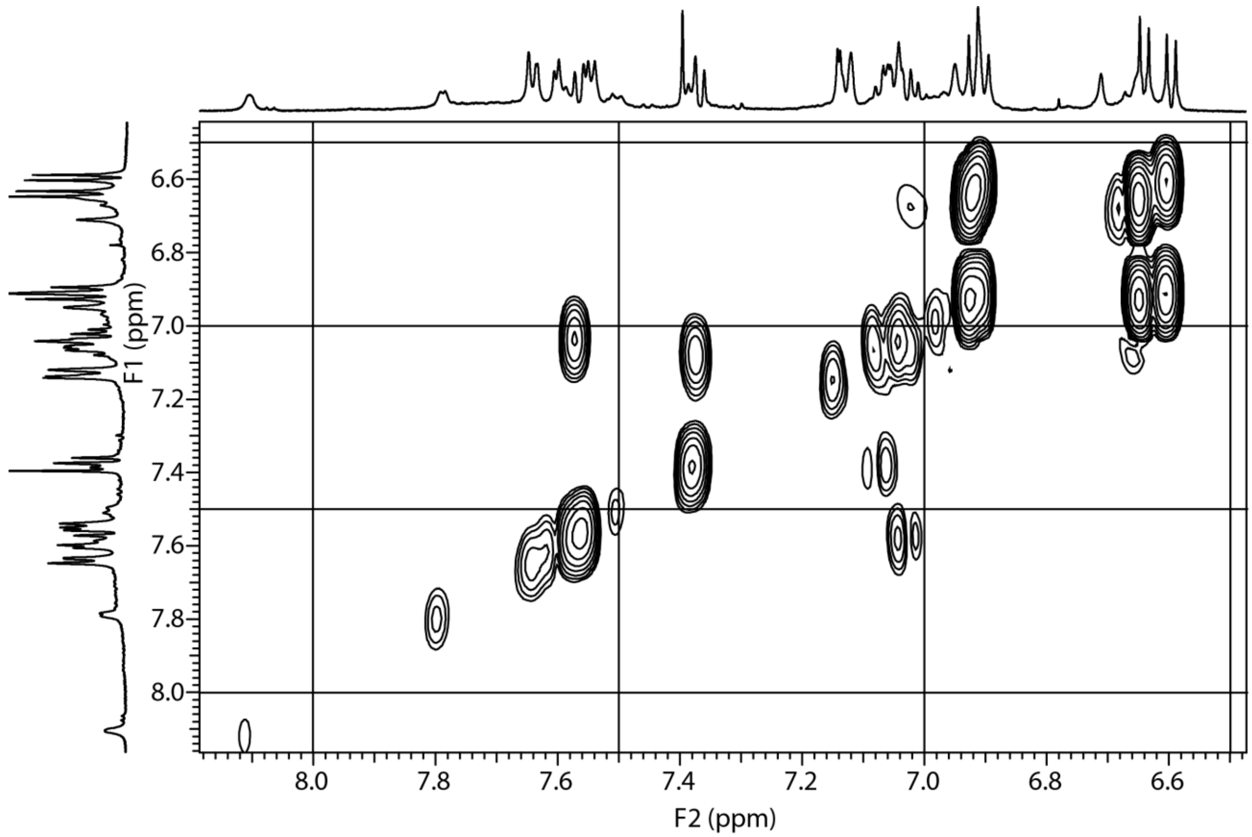

COSY NMR (expanded) of **7c**

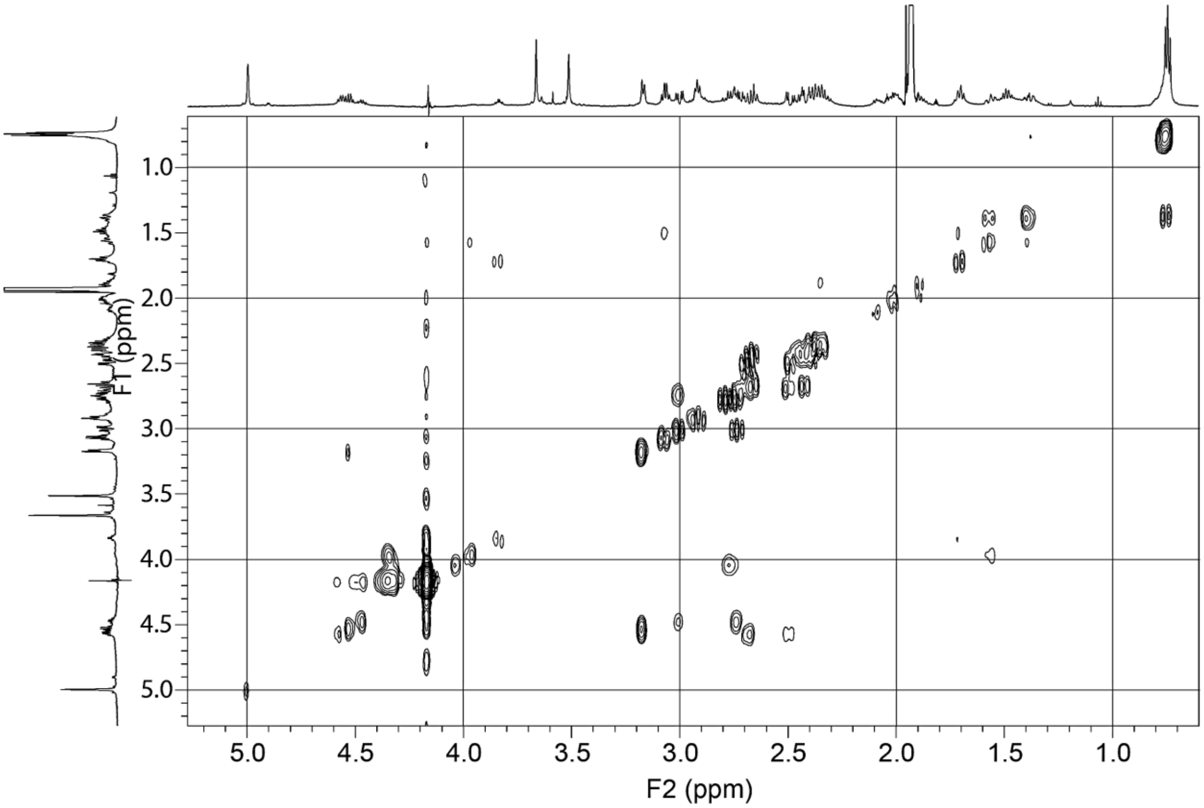

COSY NMR (expanded) of **7c**

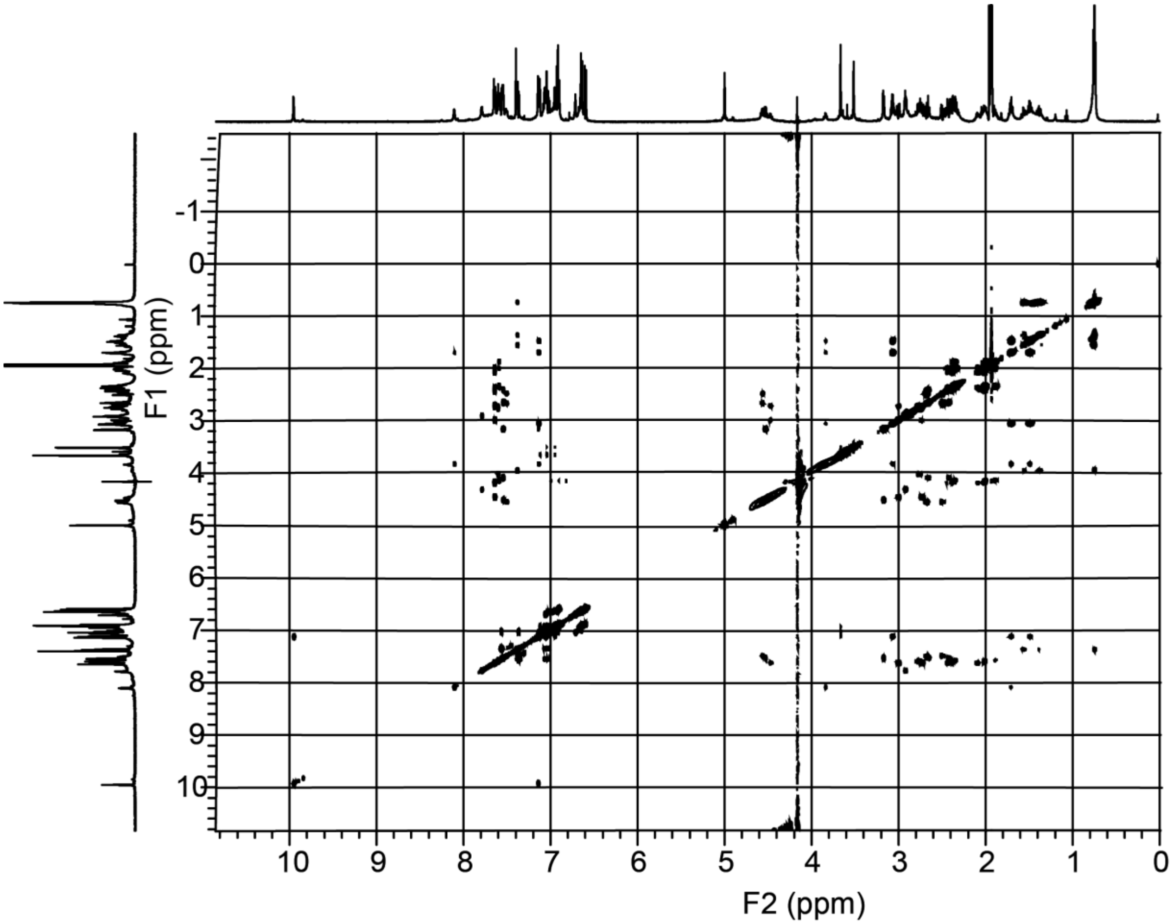

TOCSY NMR of **7c**

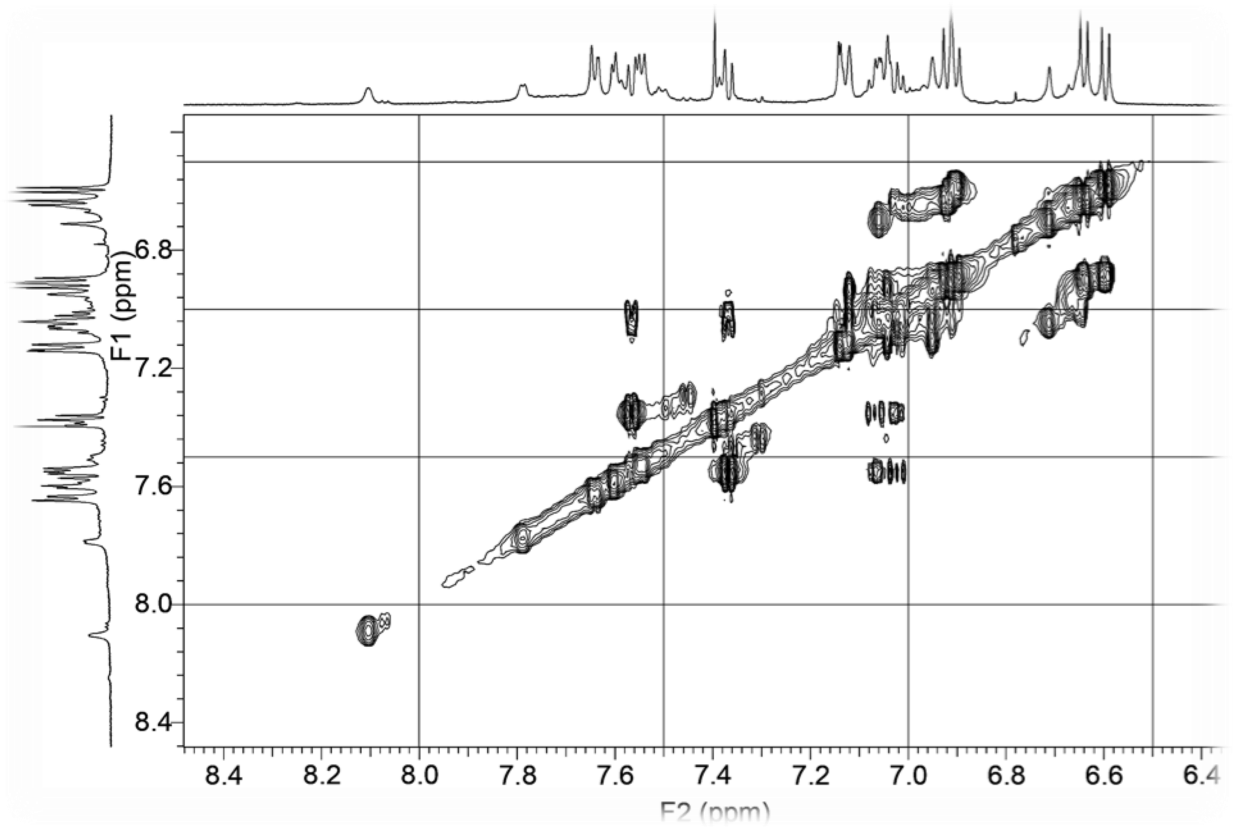
6.8
7.2
7.6
8.0
8.4
F1 (ppm)
8.4
8.2
8.0
7.8
7.6
7.4
7.2
7.0
6.8
6.6
6.4
F2 (ppm)

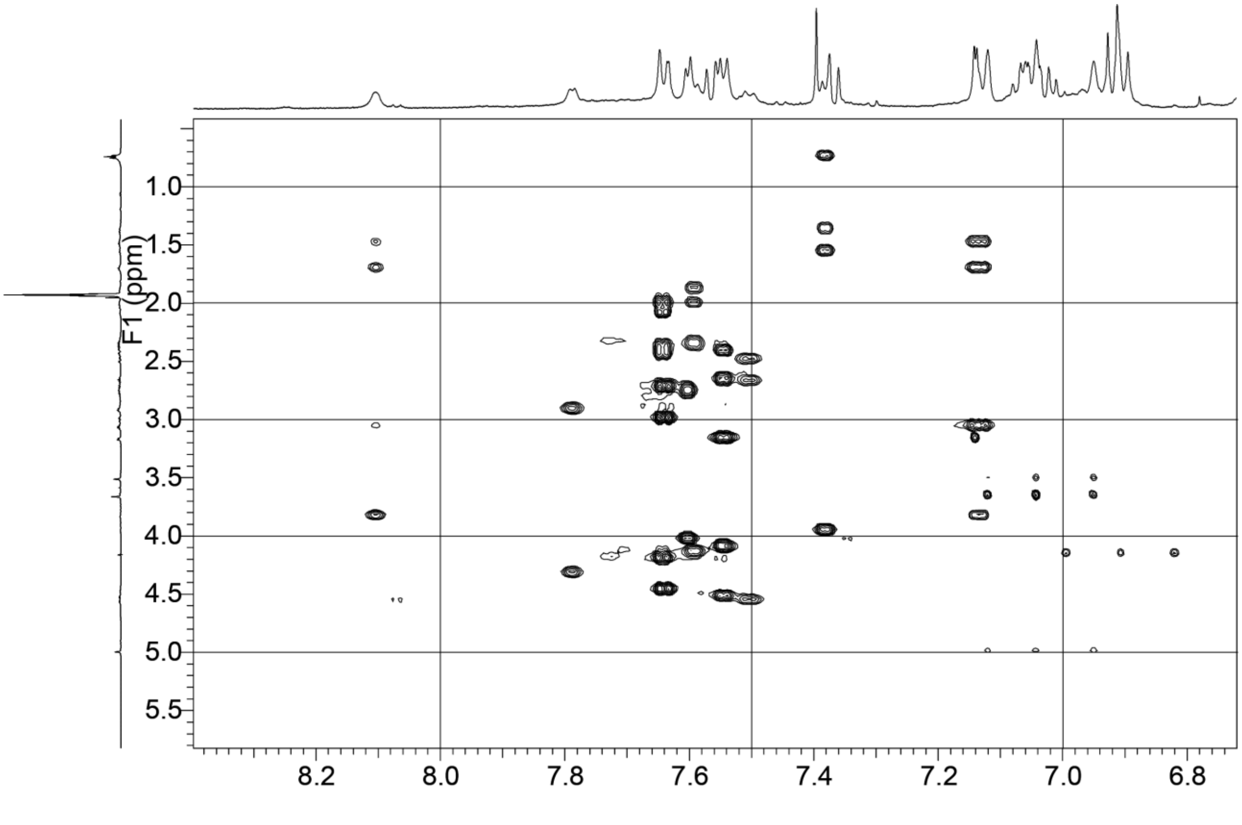
1.0
1.5
2.0
2.5
3.0
3.5
4.0
4.5
5.0
5.5
F1 (ppm)
8.2
8.0
7.8
7.6
7.4
7.2
7.0
6.8

TOCSY NMR (expanded) of **7c**

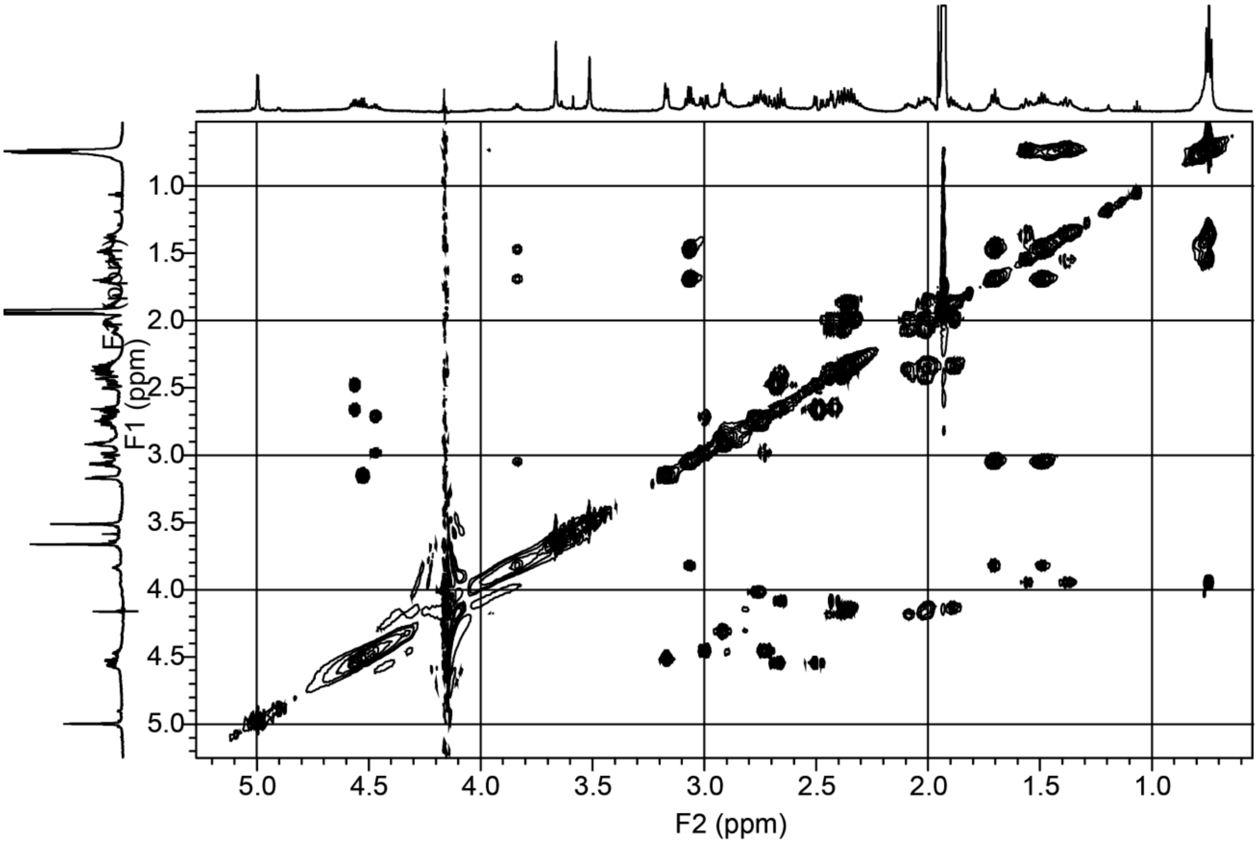

TOCSY NMR (expanded) of **7c**

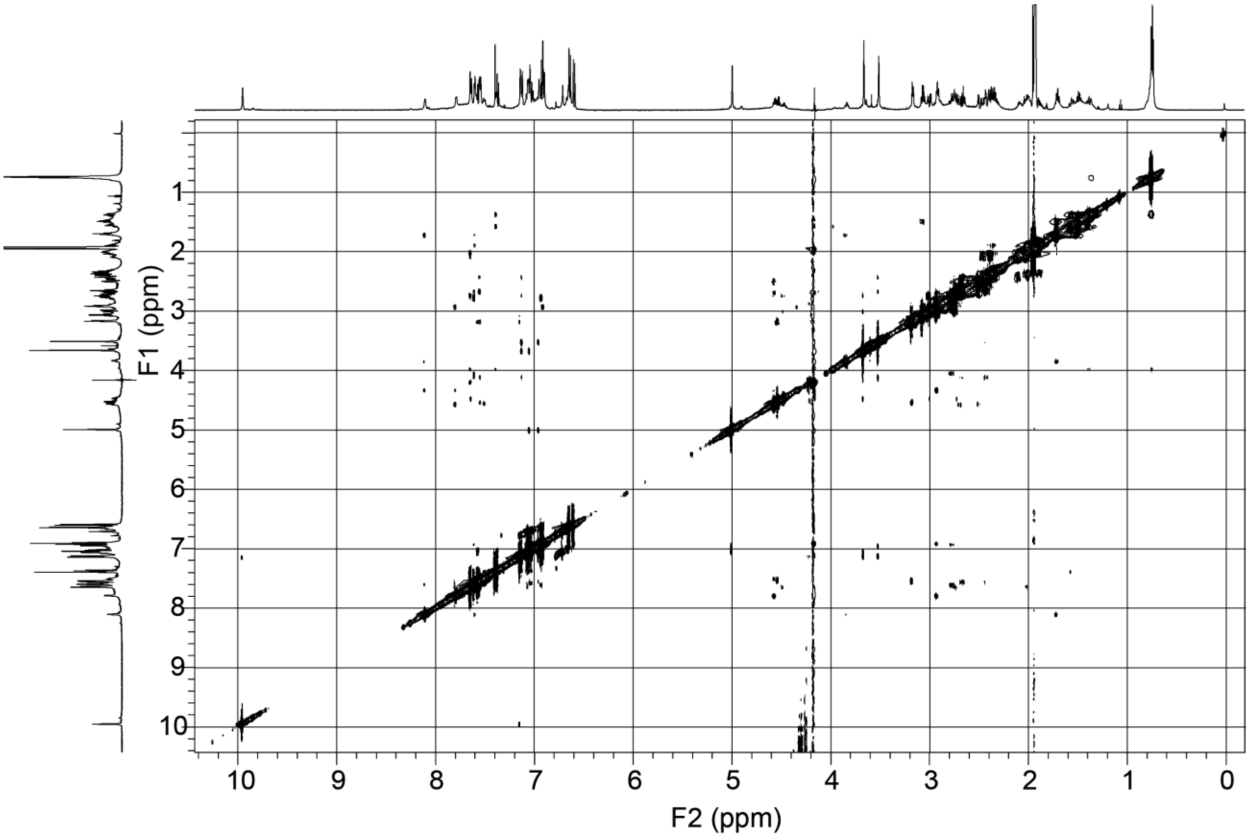

NOESY NMR of **7c**

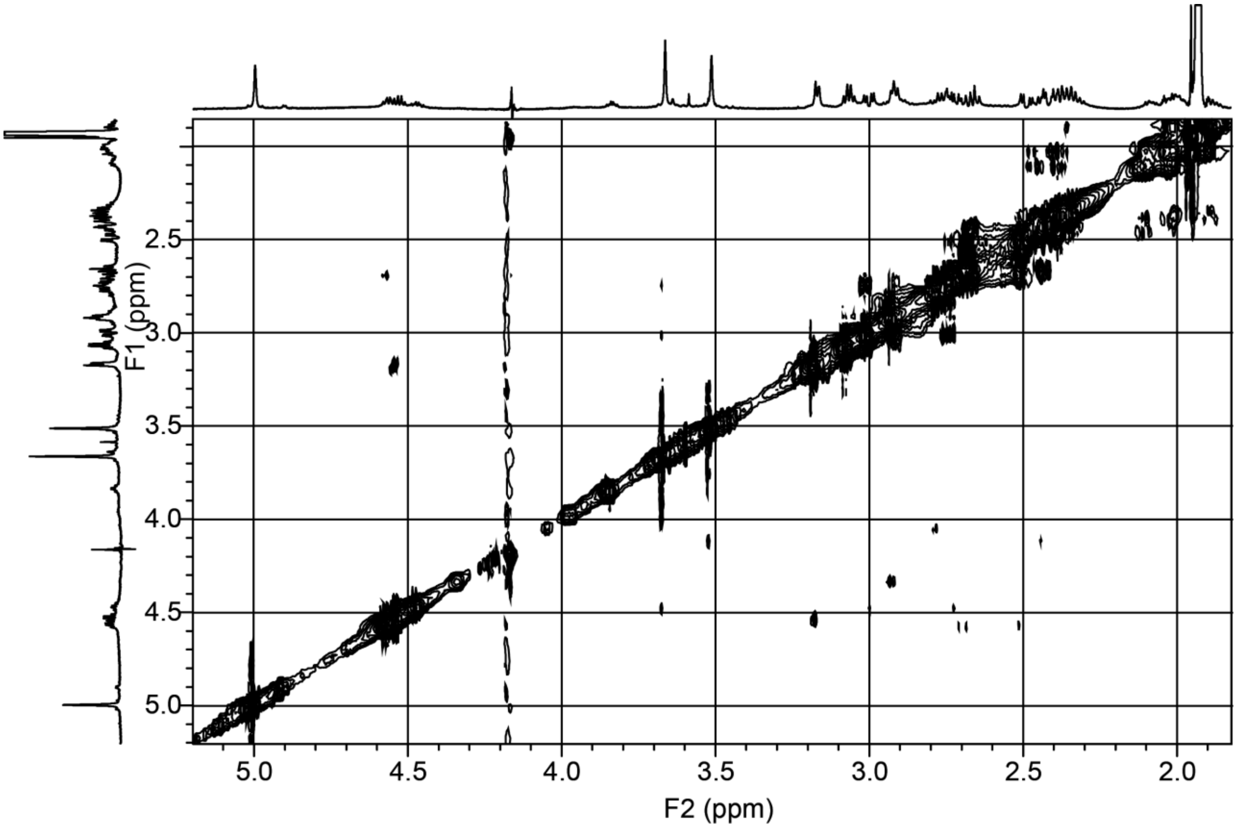

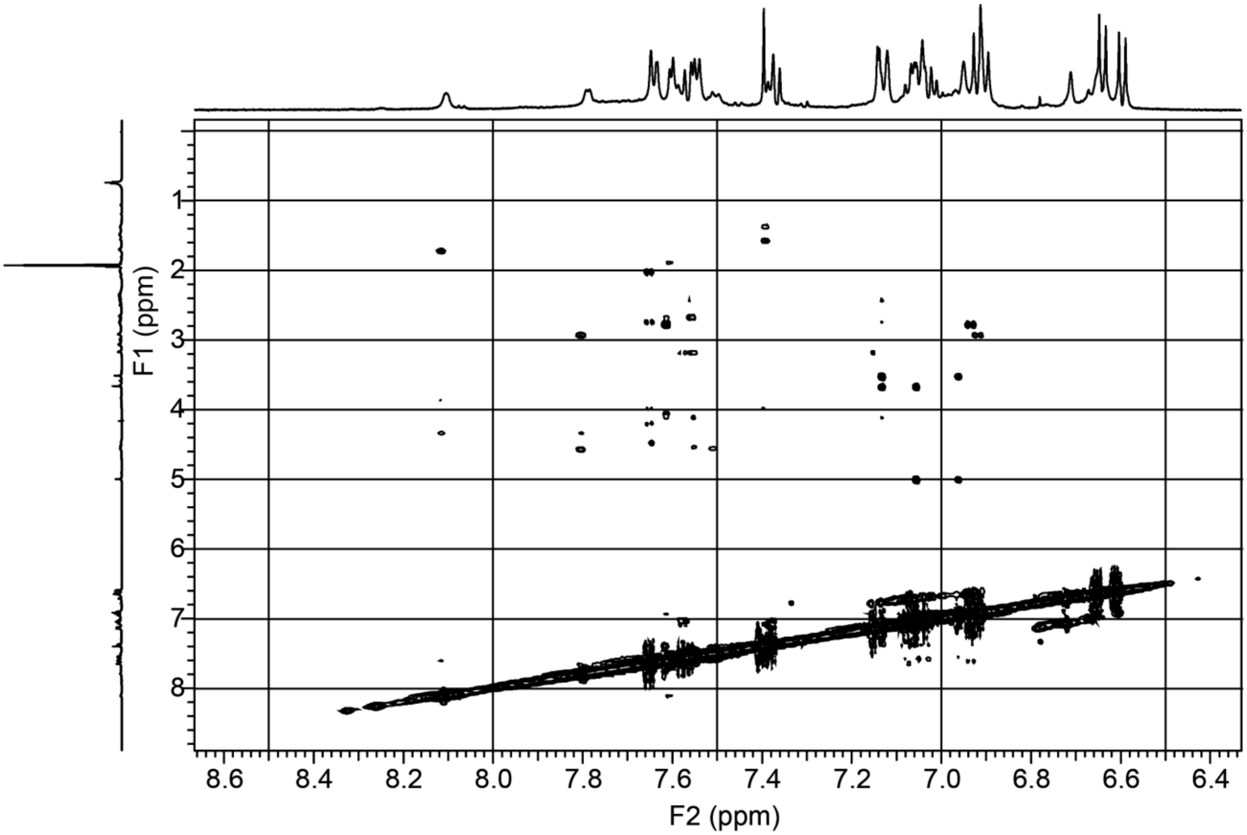

NOESY NMR (expanded) of **7c**

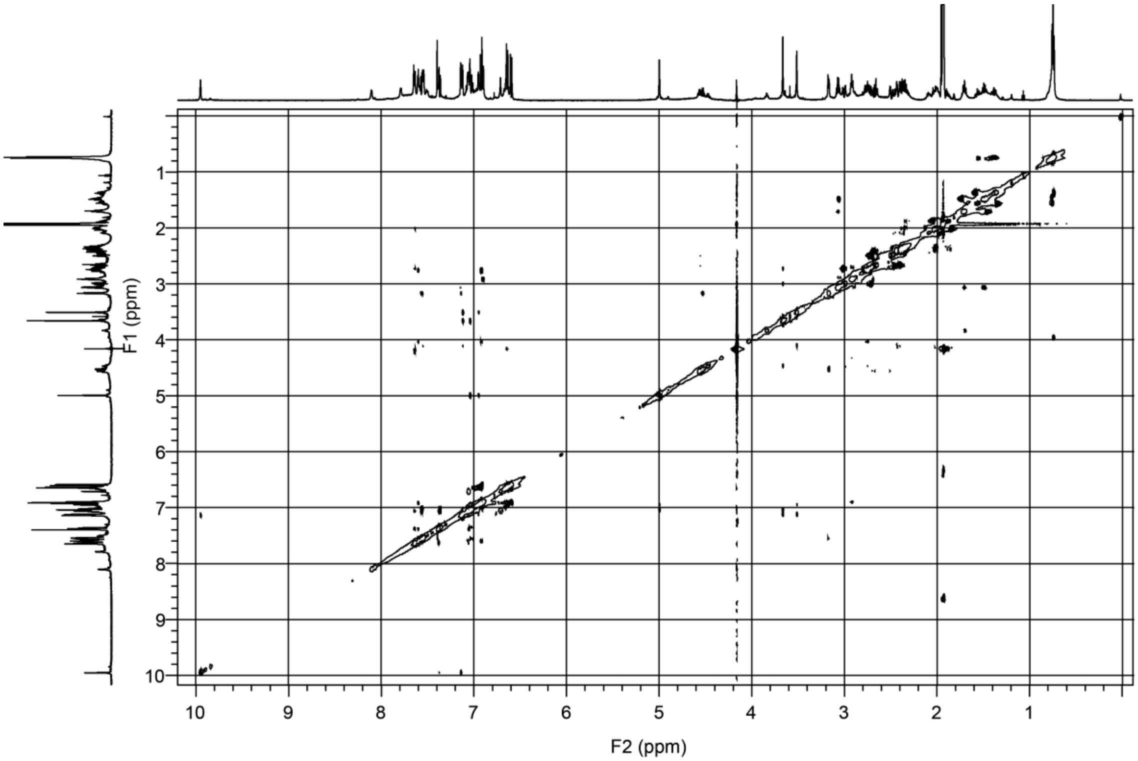

ROESY NMR of **7c**

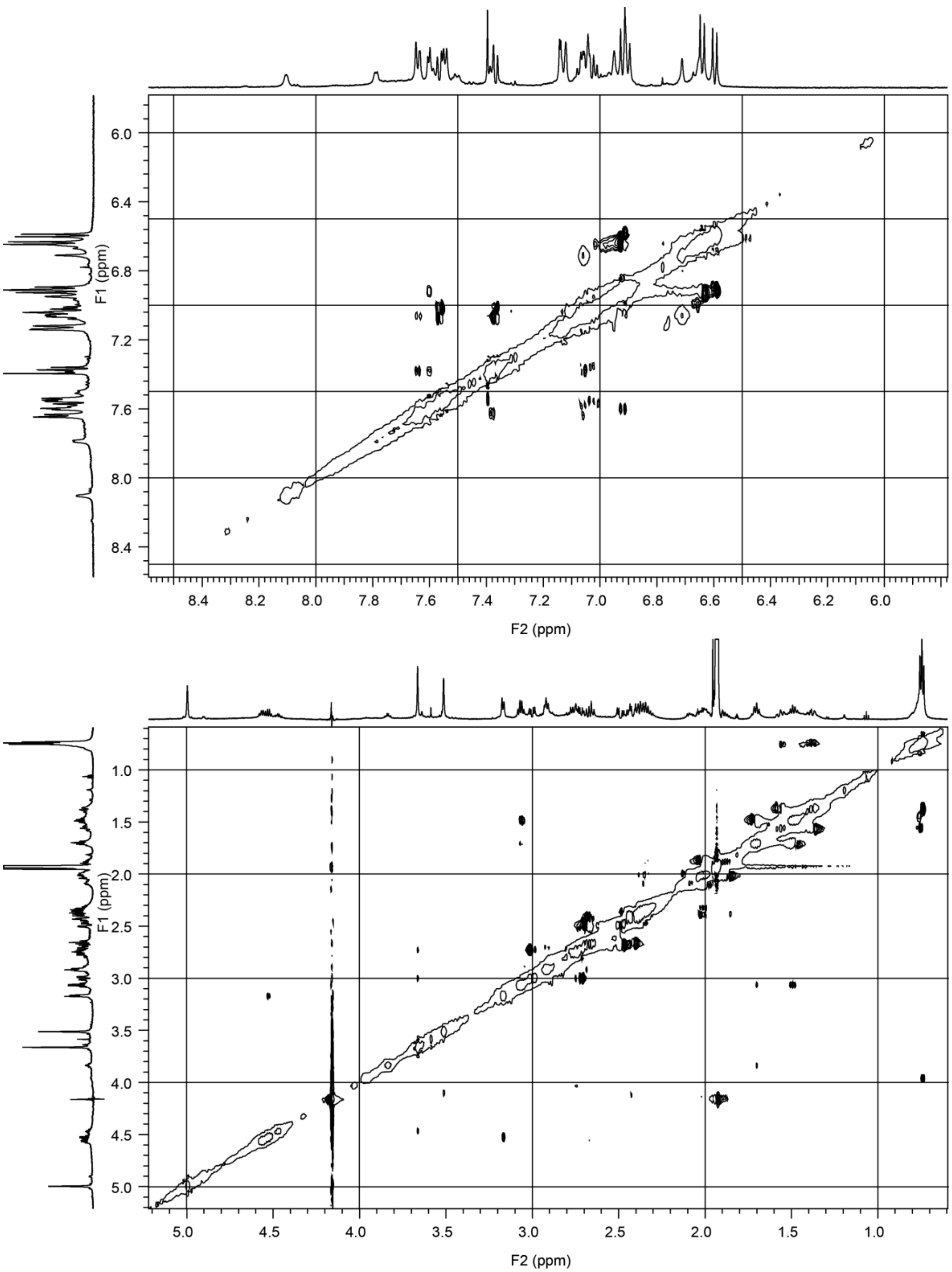

ROESY NMR (expanded) of **7c**

7.3. *Proton NMR assignment and corresponding NMR spectra of* ***3c***(1H, COSY, TOCSY, NOESY and ROESY)

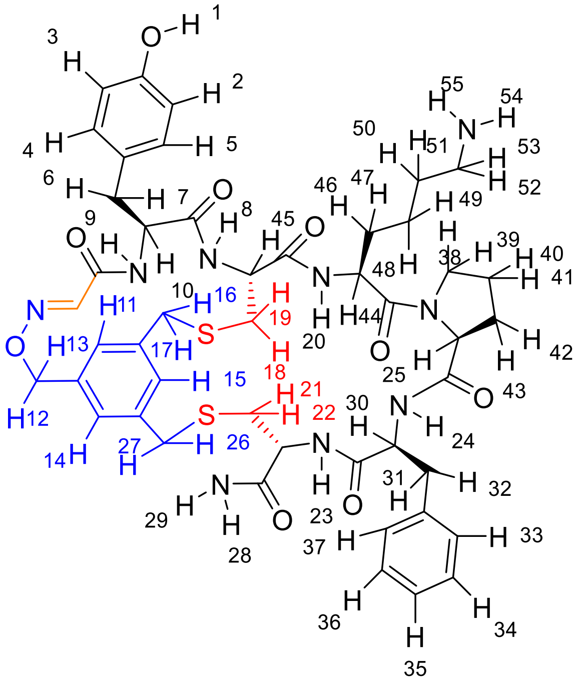

Residue	NH	ppm	Hα	ppm	Hβ'	ppm	Hγ'	ppm	Hδ'	ppm	Hε'	ppm
Tyr	9	7.27	10	4.86	6, 7	3.05						
Cys	8	7.07	45	4.50	18, 19	3.14, 2.02						
Lys	20	7.47	44	3.88	46, 47	1.54, 1.49	48, 49	1.28, 1.16	50, 51	1.45	52, 53	2.75
Pro			25	4.26	42, 43	2.03, 1.84	40, 41	1.53, 0.70	18, 39	3.12, 2.84		
Phe	24	7.95	30	4.25	31, 32	3.20, 3.00						
Cys	23	7.36	66	4.44	21, 22	2.29						
Other signals												
Cys	28	7.32	29	6.64								
Lys	54,55	7.29										
Phe (Ar)	33, 37	7.28	34-36	7.21 1								
Tyr (Ar)	2, 3	7.04	4, 5	6.77								
TSL-1	11	7.18	14	7.17	15	6.92	12, 13	5.08, 4.97	21, 22	3.64	18, 19	3.55

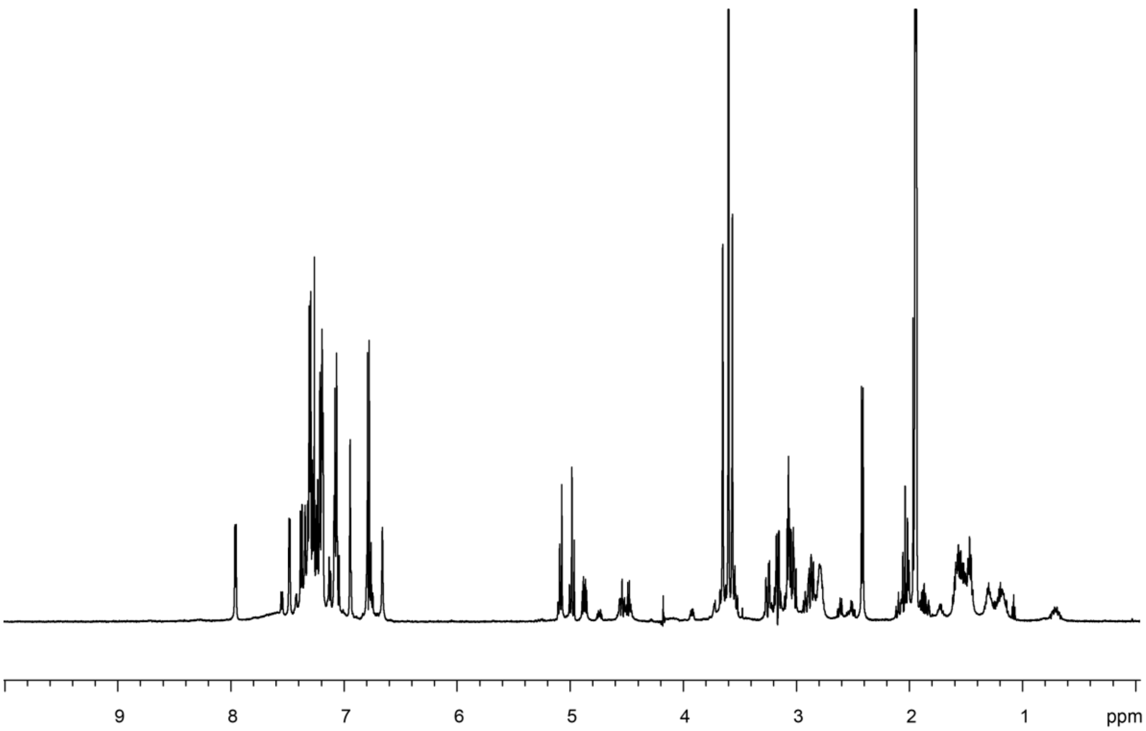

1H NMR of **3c**

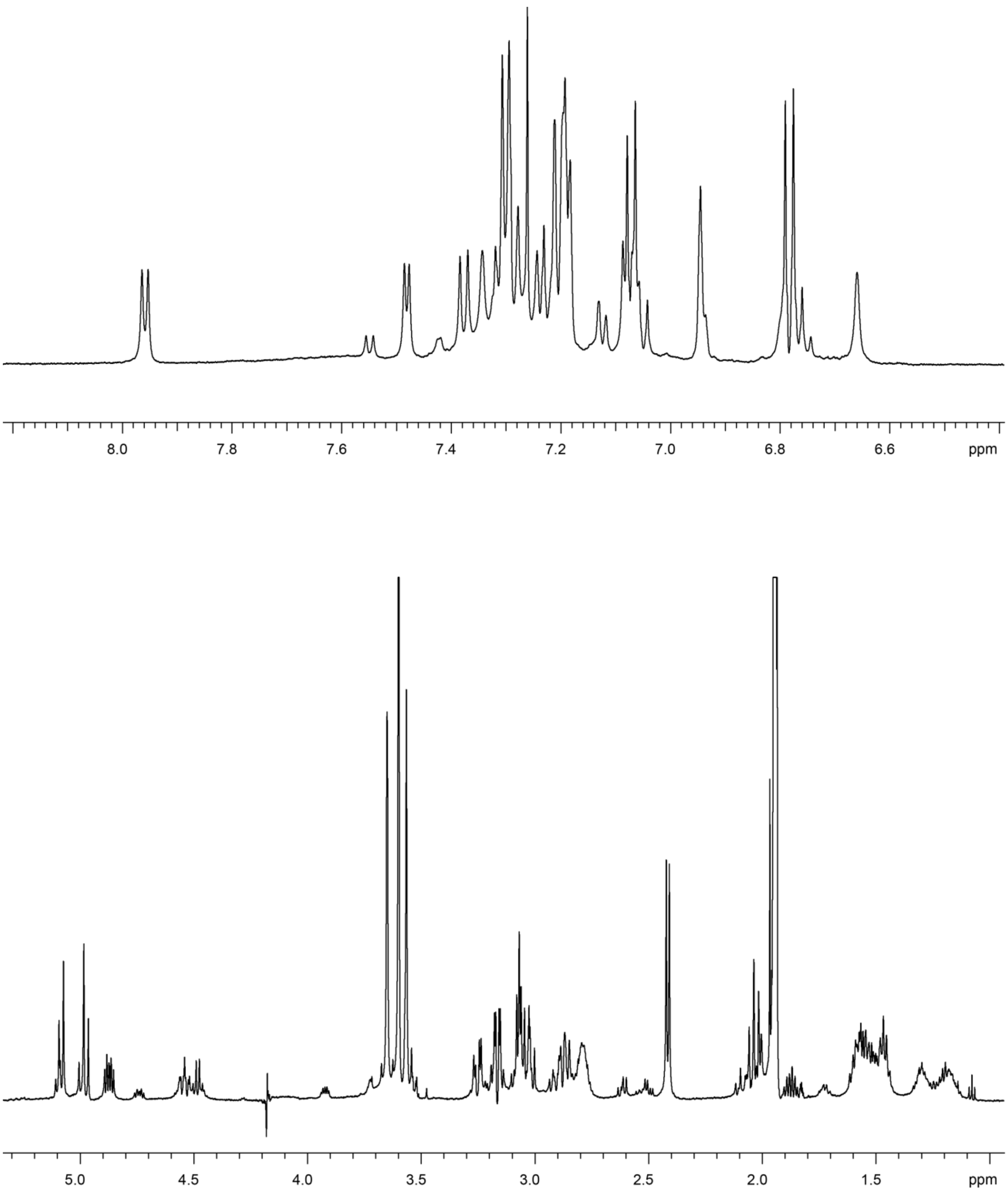

1H NMR (expanded) of **3c**

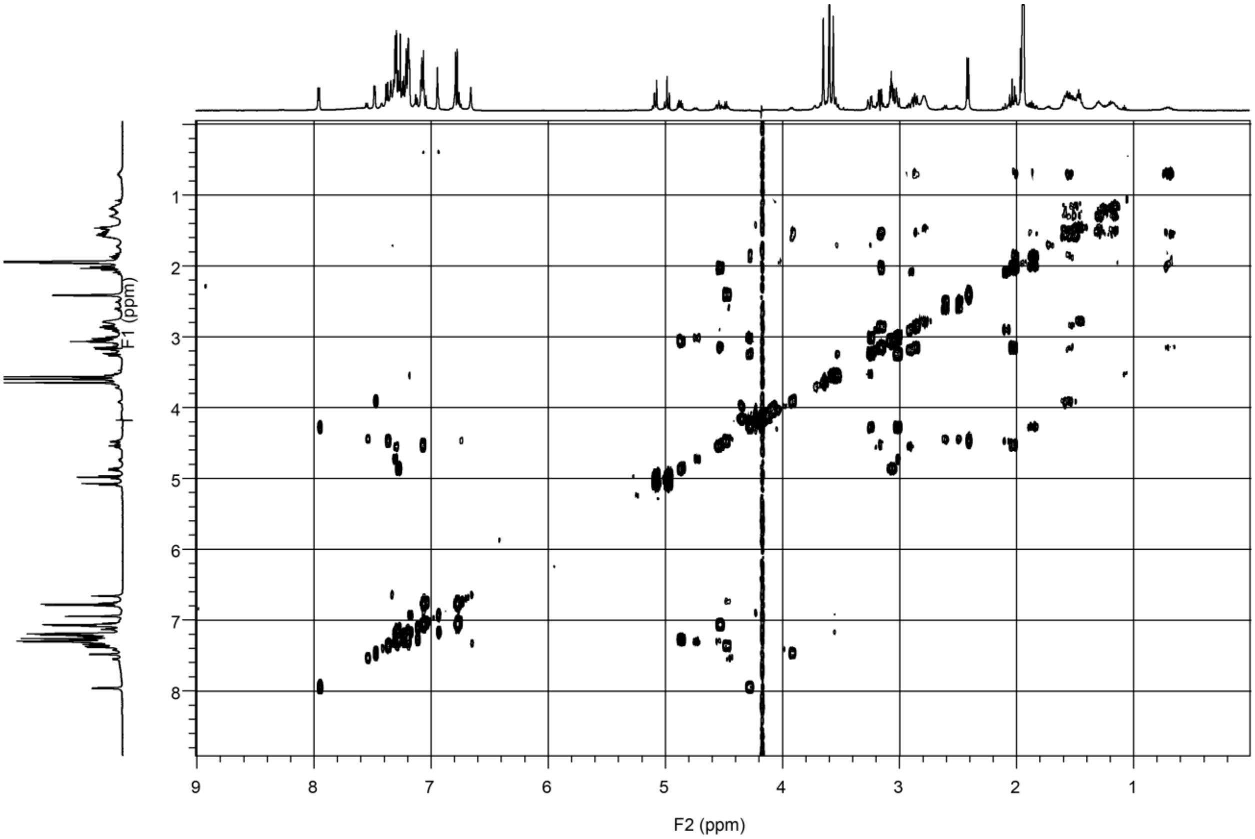

COSY NMR of **3c**

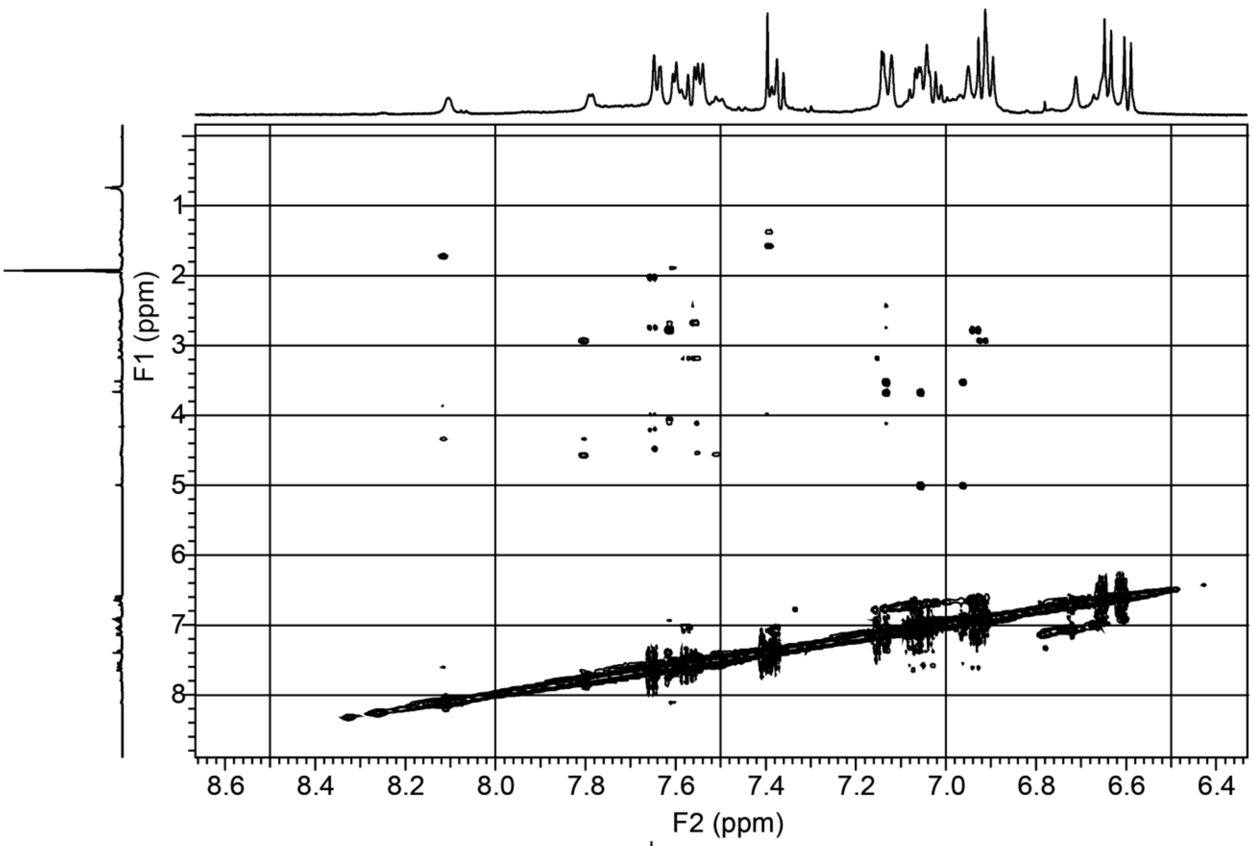

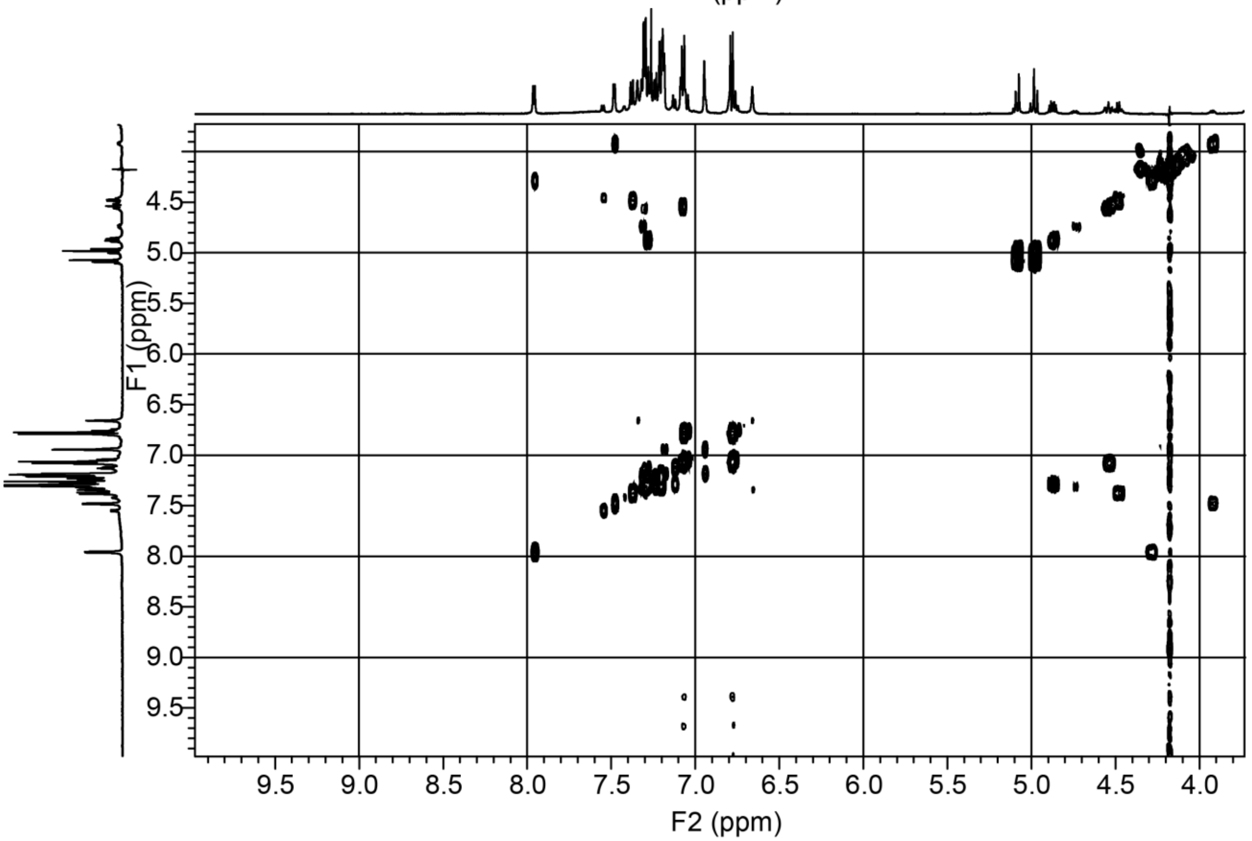

COSY NMR (expanded) of **3c**

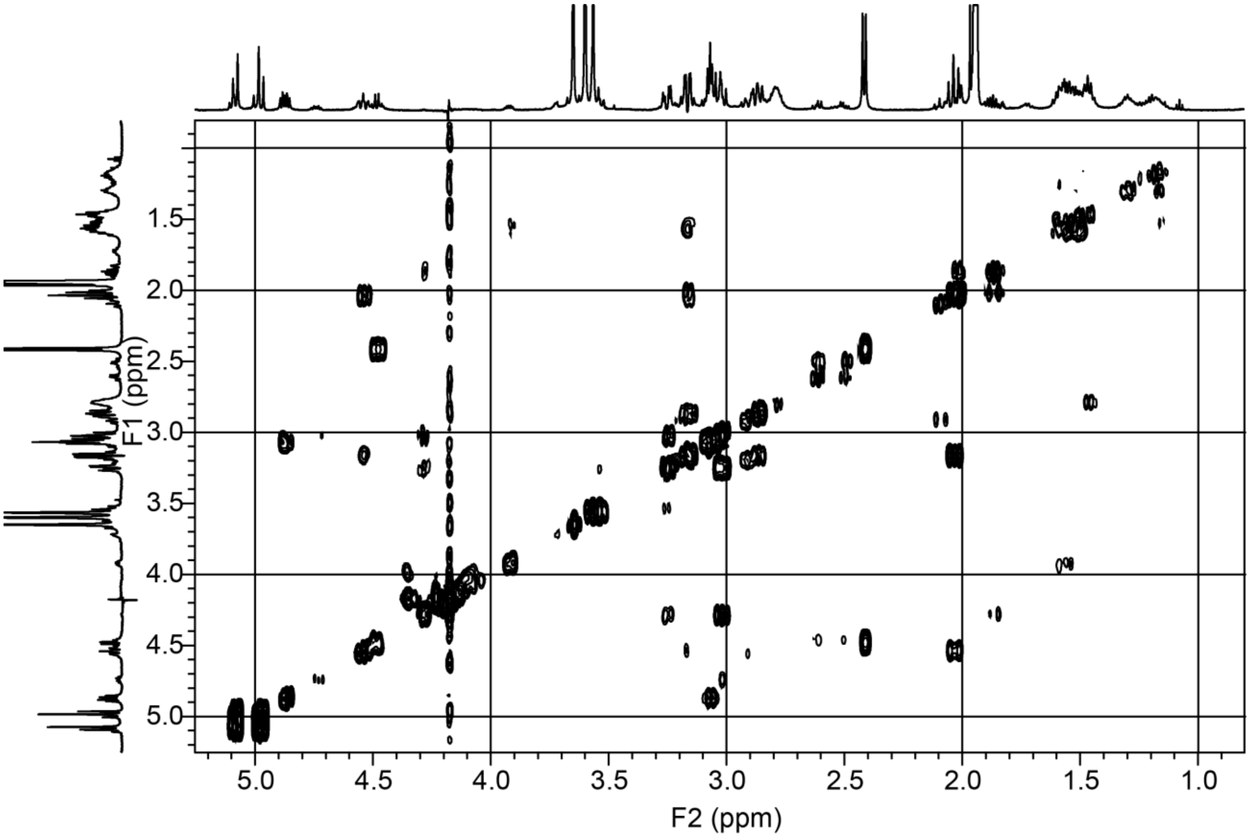

COSY NMR (expanded) of **3c**

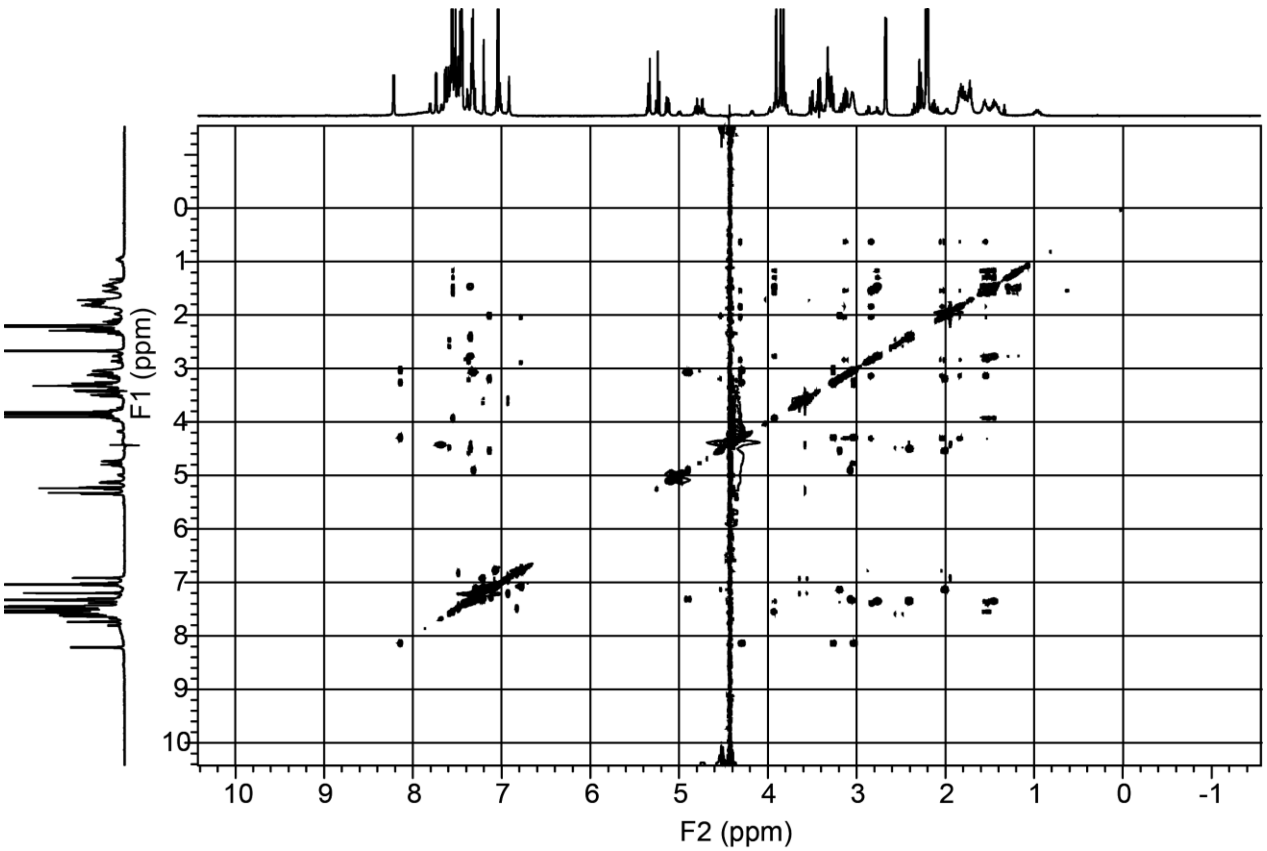

TOCSY NMR of **3c**

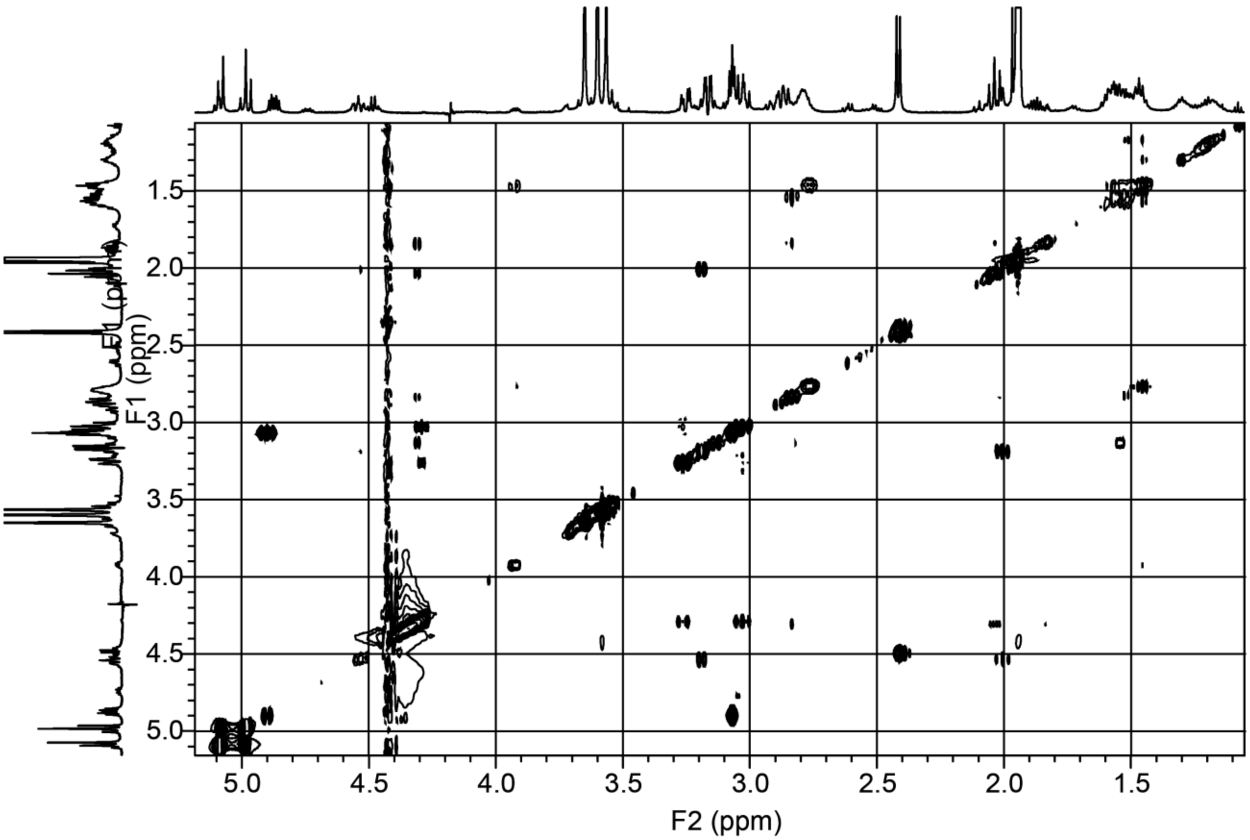

TOCSY NMR (expanded) of **3c**

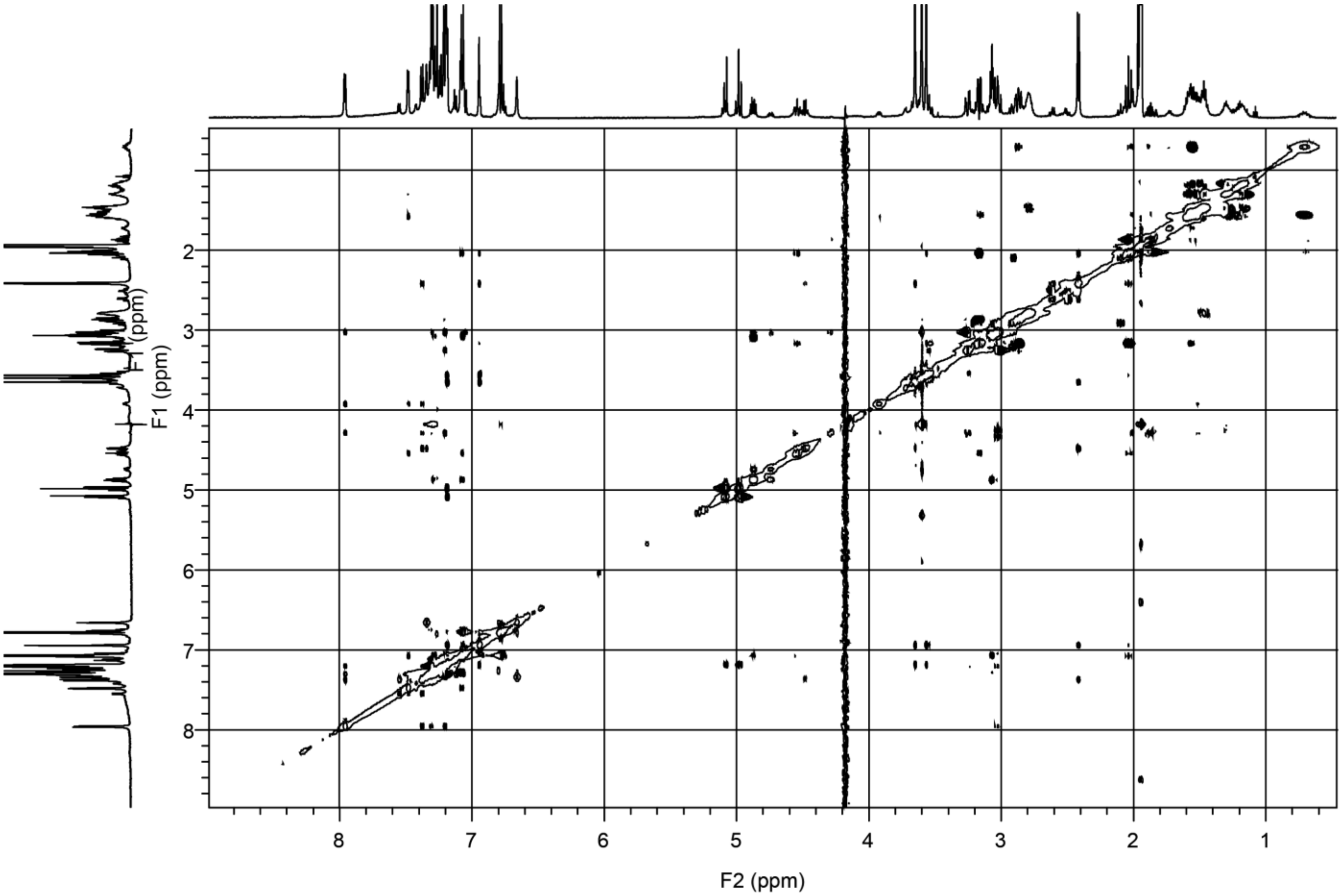

ROESY NMR of **3c**

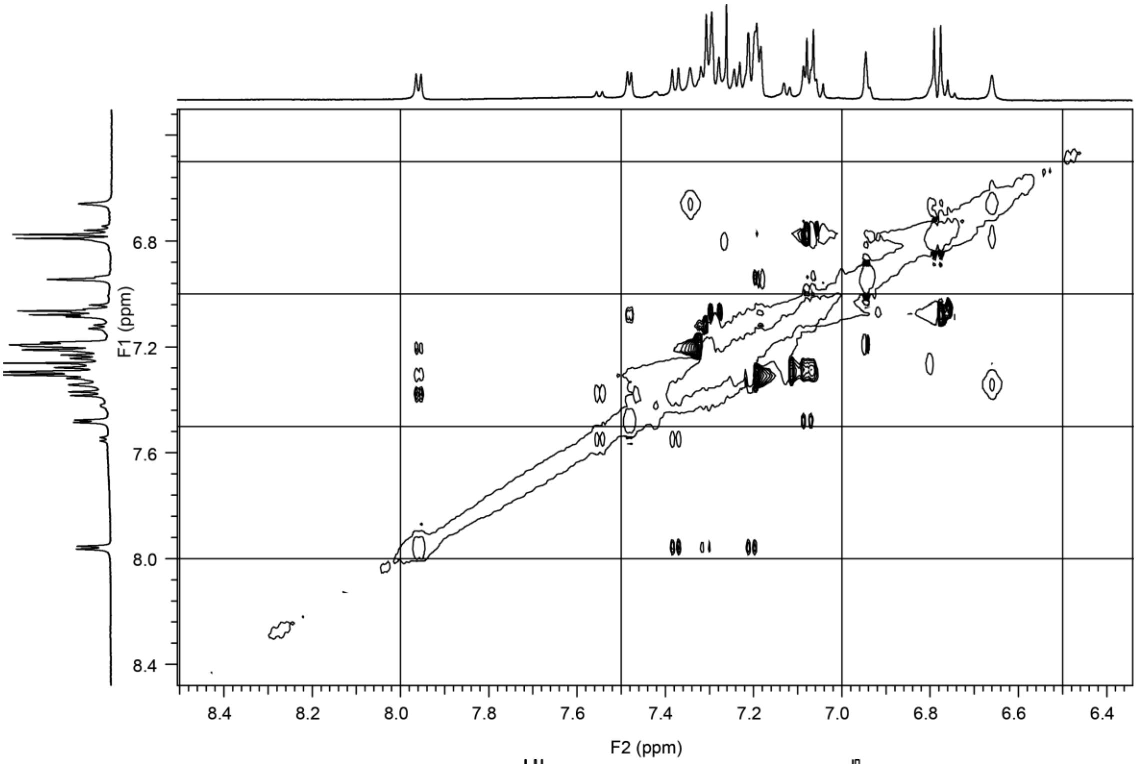

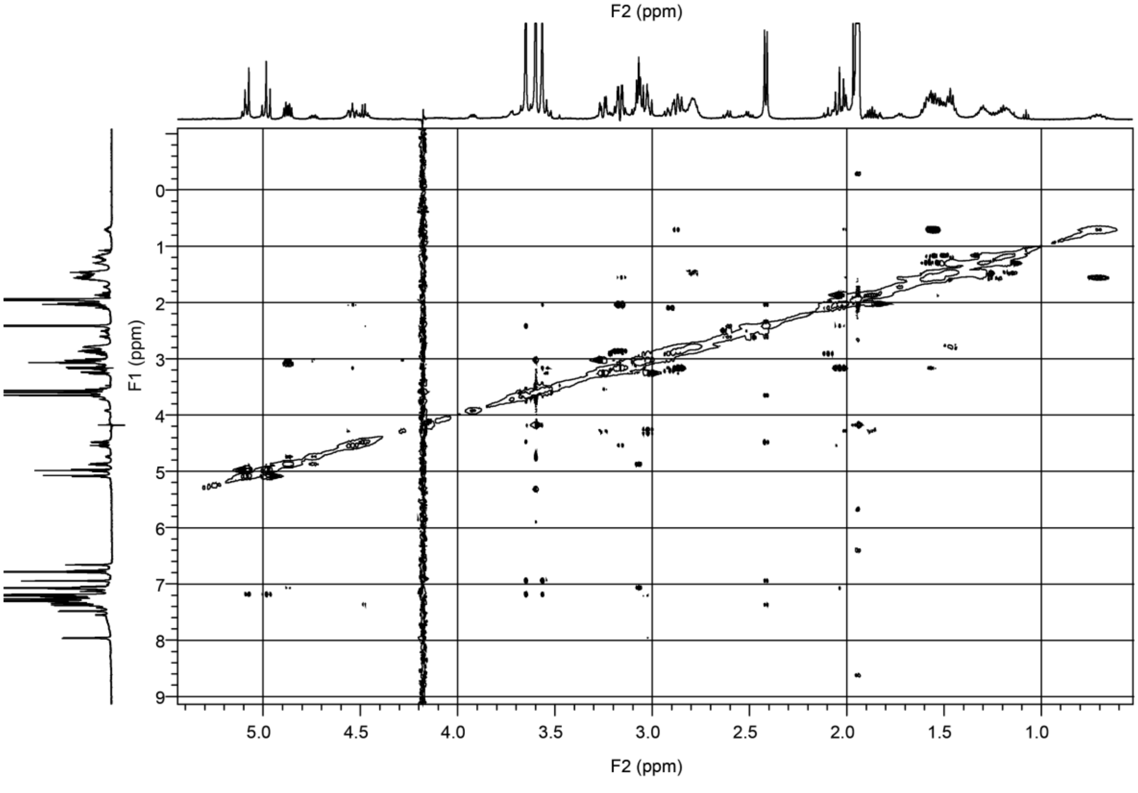

ROESY NMR (expanded) of **3c**

References:

1. Kalhor-Monfared, S.; Jafari, M. R.; Patterson, J. T.; Kitov, P. I.; Dwyer, J. J.; Nuss, J. M.; Derda, R., Rapid biocompatible macrocyclization of peptides with decafluoro-diphenylsulfone. *Chem. Sci.* **2016,** *7* (6), 3785-3790.
2. Wang, G.; Li, X.; Wang, Z., APD3: the antimicrobial peptide database as a tool for research and education. *Nucleic Acids Res.* **2016,** *44* (D1), D1087-93.
3. Thevenet, P.; Shen, Y.; Maupetit, J.; Guyon, F.; Derreumaux, P.; Tuffery, P., PEP-FOLD: an updated de novo structure prediction server for both linear and disulfide bonded cyclic peptides. *Nucleic Acids Res.* **2012,** *40* (Web Server issue), W288-93.
4. Shen, Y.; Maupetit, J.; Derreumaux, P.; Tuffery, P., Improved PEP-FOLD Approach for Peptide and Miniprotein Structure Prediction. *J Chem Theory Comput* **2014,** *10* (10), 4745-58.
5. Lamiable, A.; Thevenet, P.; Rey, J.; Vavrusa, M.; Derreumaux, P.; Tuffery, P., PEP-FOLD3: faster de novo structure prediction for linear peptides in solution and in complex. *Nucleic Acids Res.* **2016,** *44* (W1), W449-54.
6. He, B.; Tjhung, K. F.; Bennett, N. J.; Chou, Y.; Rau, A.; Huang, J.; Derda, R., Compositional Bias in Naive and Chemically-modified Phage-Displayed Libraries uncovered by Paired-end Deep Sequencing. *Sci. Rep.* **2018,** *8* (1), 1214.
7. Sojitra, M.; Sarkar, S.; Maghera, J.; Rodrigues, E.; Carpenter, E.; Seth, S.; Vinals, D. F.; Bennett, N.; Reddy, R.; Khalil, A.; Xue, X.; Bell, M.; Zheng, R. B.; Ling, C.-C.; Lowary, T. L.; Paulson, J. C.; Macauley, M. S.; Derda, R., Genetically Encoded, Multivalent Liquid Glycan Array (LiGA). *bioRxiv* **2020**, 2020.03.24.997536.
8. Tjhung, K. F.; Deiss, F.; Tran, J.; Chou, Y.; Derda, R., Intra-domain phage display (ID-PhD) of peptides and protein mini-domains censored from canonical pIII phage display. *Front Microbiol* **2015,** *6*, 340.
9. Ng, S.; Jafari, M. R.; Matochko, W. L.; Derda, R., Quantitative Synthesis of Genetically Encoded Glycopeptide Libraries Displayed on M13 Phage. *ACS Chem. Biol.* **2012,** *7* (9), 1482-1487.
10. Maestro. 11.7 ed.; Schrödinger, LLC: New York, NY, 2018.
11. Frolov, A. I.; Kiselev, M. G., Prediction of Cosolvent Effect on Solvation Free Energies and Solubilities of Organic Compounds in Supercritical Carbon Dioxide Based on Fully Atomistic Molecular Simulations. *J. Phys. Chem. B* **2014,** *118* (40), 11769-11780.
12. Hess, B.; Kutzner, C.; van der Spoel, D.; Lindahl, E., GROMACS 4: Algorithms for Highly Efficient, Load-Balanced, and Scalable Molecular Simulation. *J. Chem. Theory Comput.* **2008,** *4* (3), 435-447.
13. Kaminski, G. A.; Friesner, R. A.; Tirado-Rives, J.; Jorgensen, W. L., Evaluation and Reparametrization of the OPLS-AA Force Field for Proteins via Comparison with Accurate Quantum Chemical Calculations on Peptides. *J. Phys. Chem. B* **2001,** *105* (28), 6474-6487.
14. Jorgensen, W. L.; Chandrasekhar, J.; Madura, J. D.; Impey, R. W.; Klein, M. L., Comparison of simple potential functions for simulating liquid water. *J. Chem. Phys.* **1983,** *79* (2), 926-935.
15. Bussi, G.; Donadio, D.; Parrinello, M., Canonical sampling through velocity rescaling. *J. Chem. Phys.* **2007,** *126* (1), 014101.
16. Cheng, A.; Merz, K. M., Application of the Nosé−Hoover Chain Algorithm to the Study of Protein Dynamics. *J. Chem. Phys* **1996,** *100* (5), 1927-1937.
17. Lingenheil, M.; Denschlag, R.; Reichold, R.; Tavan, P., The "Hot-Solvent/Cold-Solute" Problem Revisited. *J. Chem. Theory Comput.* **2008,** *4* (8), 1293-1306.
18. Berendsen, H. J. C.; Postma, J. P. M.; Gunsteren, W. F. v.; DiNola, A.; Haak, J. R., Molecular dynamics with coupling to an external bath. *J. Chem. Phys.* **1984,** *81* (8), 3684-3690.
19. Hess, B.; Bekker, H.; Berendsen, H. J. C.; Fraaije, J. G. E. M., LINCS: A linear constraint solver for molecular simulations. *J. Comput. Chem.* **1997,** *18* (12), 1463-1472.
20. Hockney, R. W.; Eastwood, J. W., *Computer simulation using particles*. Taylor & Francis Group: New York, 1988.
21. Darden, T.; York, D.; Pedersen, L., Particle mesh Ewald: An N· log (N) method for Ewald sums in large systems. *J. Chem. Phys.* **1993,** *98* (12), 10089-10092.
22. Essmann, U.; Perera, L.; Berkowitz, M. L.; Darden, T.; Lee, H.; Pedersen, L. G., A smooth particle mesh Ewald method. *J. Chem. Phys.* **1995,** *103* (19), 8577-8593.
23. Damas, J. o. M.; Filipe, L. C.; Campos, S. R.; Lousa, D.; Victor, B. L.; Baptista, A. n. M.; Soares, C. u. M., Predicting the thermodynamics and kinetics of helix formation in a cyclic peptide model. *J. Chem. Theory Comput.* **2013,** *9* (11), 5148-5157.
